# Comparative Genome Analysis and Phylogenetic Relationship of Order Liliales Insight from the Complete Plastid Genome Sequences of Two Lilies (*Lilium longiflorum* and *Alstroemeria aurea*)

**DOI:** 10.1371/journal.pone.0068180

**Published:** 2013-06-18

**Authors:** Jung Sung Kim, Joo-Hwan Kim

**Affiliations:** Department of Life Science, Gacheon University, Seongnam, Gyeonggi-do, South Korea; University of Lausanne, Switzerland

## Abstract

Monocots are one of the most diverse, successful and economically important clades of angiosperms. We attempt to analyse the complete plastid genome sequences of two lilies and their lengths were 152,793bp in *Lilium* *longiflorum* (Liliaceae) and 155,510bp in *Alstroemeria* *aurea* (Alstroemeriaceae). Phylogenetic analyses were performed for 28 taxa including major lineages of monocots using the sequences of 79 plastid genes for clarifying the phylogenetic relationship of the order Liliales. The sister relationship of Liliales and Asparagales-commelinids was improved with high resolution. Comparative analyses of inter-familial and inter-specific sequence variation were also carried out among three families of Liliaceae, Smilacaceae, and Alstroemeriaceae, and between two *Lilium* species of *L* *. longflorum* and *L* *. superbum* . Gene content and order were conserved in the order Liliales except *inf*A loss in *Smilax* and *Alstroemeria* . IR boundaries were similar in IRa, however, IRb showed different extension patterns as JLB of *Smilax* and JSB in *Alstroemeria* . Ka/Ks ratio was high in *mat*K among the pair-wise comparison of three families and the most variable genes were *psa*J, *ycf*1, *rpl*32, *rpl*22, *mat*K, and *ccs*A among the three families and *rps*15, *rpo*A, *mat*K, and *ndh*F between *Lilium*.

## Introduction

The plastid genome generally contains 30–50 different RNA genes and about 100 protein-coding genes in land plants, which are roughly classified into two main groups: genes involved in the expression and translation machinery of the chloroplast, and genes related to bioenergetics and photosynthetic function [1]. It is highly conserved in organization, gene order and content with a typical circular form [2]. In the past several years, numerous data for genomic sequences have been pouring in and are being applied in the plant phylogeny field due to the development and advancement of next-generation sequencing (NGS) technologies. This method has contributed to improve many studies in plant biology: molecular marker development, hybridisation and introgression, transcriptome investigation, phylogenetic and ecological studies and polyploidy genetics [3].

Among the monocots, sequence data for the plastid genome have been rapidly accumulating for the Poaceae members in particular, focusing on the economically important plant species [4–14] since *Oryza sativa* was first analyzed [15]. This moved the interest to non-model plants and gave rise to an expansion of the database for monocot plastid genome sequences [12,14,16–21], giving us the opportunity to resolve their phylogenetic relationships. Jansen and colleagues [22] examined the phylogenetic relationships of angiosperms using 81 genes and reported specific gene and intron losses in the main lineage of monocots, including *accD* in *Acorus* (Acorales), *rps16* in *Dioscorea* (Dioscoreales), the *ndh* gene cluster in *Phalaenopsis* (Asparagales), *rpl32* in *Yucca* (Asparagales) and *accD*, the *clpP* intron, the *rpoC1* intron and *ycf1* and *ycf2* in Poales.

Monocots are one of the major radiations of angiosperms. They have relatively uniform characters: a single cotyledon, parallel leaf venation, floral parts in threes, sieve-tube plastids with several cuneate protein crystals, scattered vascular bundles in their stems, no vascular cambium-producing secondary phloem and secondary xylem with some exceptions [23]. They comprise *ca* 61,000 species in 78–80 families and 11 orders [24,25] and are one of the most diverse, morphologically varied, ecologically successful and economically important clades of angiosperms [12].

Although many molecular phylogenetic studies have led to a remarkable reclassification and understanding of relationships within and among the orders, many relationships remain weakly supported among the orders and families. In contrast to the morphological data, molecular data placed *Acorus* on the root node within monocots [26,27]. This was also revealed by recent plastome sequence analyses [12,22,28]. Chase and colleagues [26] suggested a well-defined ordinal relationship in monocots using combined data from seven genes: four plastid genes, one mitochondrial gene and two nuclear ribosomal genes. Twelve orders were positioned subsequently from the root: Acorales, Alismatales, Petrosaviales, Dioscoreales and Pandanales, Liliales, Asparagales and commelinids, which includes the five orders of Arecales, Commelinales, Zingiberales, Dasypogonales and Poales. Although all orders were strongly supported, the sister relationship between Liliales and Asparagales remains uncertain as does the ordinal relationship within commelinids. Givnish and colleagues [12] tried to resolve these uncertain relationships using 81 plastome-encoded gene sequences and an increase in taxon sampling, including 32 families of monocots, and the results showed an improved support value for those relationships in the maximum likelihood (ML) tree. However, their positions and relationships are still ambiguous, and in addition, the position of Liliales and Dioscoreales+Pandanales is reversed in the most parsimonious (MP) tree. These problematic relationships were also described in the phylogenetic study of Moore and colleagues [28] using plastid inverted repeat sequences. Recently Liu and colleagues [29] reported a complete plastid genome of *Smilax* *china* (Smilaceae), a member of Liliales, and tried to analyse the phylogenetic relationship of monocots using three different combined data matrix of 63 chloroplast genes (excluding *ndhA-K*, *infA*, *rps16*, and *ycf2* from 77 genes data matrix), 77 protein coding genes, and 81 genes (including rRNA genes from 77 genes data matrix) focused on the position of Liliales. It showed a sister relationship of Liliales and Dioscoreales-Pandanales that was strongly supported by 63 genes, while a sister relationship of Liliales with the commelinids-Asparagales clade was supported moderately on ML analysis by the 81 genes. They suggested a rapid divergence among Liliales, Dioscoreales-Pandanales, and commelinids-Asparagales.

We analyzed the complete plastid genomes of two lilies from different families, the Easter lily ( *Lilium* *longiflorum* , Liliaceae) and Peruvian lily ( *Alstroemeria* *aurea* , Alstroemeriaceae), which are famous ornamental flowers in the order Liliales. The former is recognized as most distinctive genera being closer to Smilacaceae and the latter is near to the basal clade in the order Liliaes [30]. Although they have not generated the complete sequence and structure, the partial protein coding gene sequences of *Lilium* *superbum* was repeatedly referred as a representative of Liliales in many studies. We also attempted to compare the organisation of the whole plastid genome among 3 families of Liliales and to clarify the phylogenetic position of the order Liliales within monocots using 79 protein-coding gene sequences. In addition, we confirmed the sequence variation in species level of the order Liliales using the 78 plastid genes of two *Lilium* species, newly analyzed *L* *. longiflorum* and *L* *. superbum* downloaded from the NCBI database. We also described here a new method for extracting plastid DNA with a simple buffer composition compared topreviously applied methodologies [31–33], in the hope that it will be applied in monocot plastome research without the centrifuge equipped swinging bucket rotor.

## Materials and Methods

### Plastid isolation from two lilies

We established a simple method for isolating plastids of *Lilium* *longiflorum* and it was successfully applied to *Alstroemeria* *aurea* . We prepared 50 g of fresh young leaves and deposited in a refrigerator for 2 days. They were then cut in 1–2-cm^2^ pieces and blended with isolation buffer [CIB; 0.35 M sorbitol, 50 mM Tris-HCl, 5 mM EDTA, 0.1% bovine serum albumin (BSA; w/v, Sigma A4503), 0.01% DL-DTT]. The slush was filtered through a 4-fold gauze by squeezing and a 3-fold miracloth (Calbiochem, cat. no. 475855) without squeezing. The filtrate was centrifuged at 200 *g* for 3 min and the supernatant was centrifuged again at 1,000 *g* for 7 min. After the pellet was completely diluted with CIB buffer without BSA, the plastid band was extracted using a 40/80% Percoll gradient by centrifuging at 3,200 *g* for 15 min. To purify the plastid solution, 3 volumes of CIB buffer without BSA, were added to the isolated solution and centrifuged at 1,700 *g* for 1 min. The pellet was dissolved again with CIB buffer without BSA, and plastid DNA was used as a template for 454 pyrosequencing and finally extracted using a DNeasy Plant Mini Kit (Qiagen, cat. no. 69104). Until the plastid DNA was isolated, the high-speed centrifuge was maintained at 4^°^C.

### Genome sequencing, assembling and annotation

The genome sequencing was performed at SolGent Co. (Daejeon, Korea). The quality of the DNA was assessed by gel electrophoresis (Fig. S1) and the quantity was estimated by a fluorescence-based method using the Quant-iT PicoGreen dsDNA Kit (Invitrogen). A whole-genome shotgun library was generated from 5 µg of the plastid DNA with the GS DNA Library Preparation Kit (Roche Applied Science) according to the manufacturer’s protocol. The DNA library was titrated by means of sequencing on the Genome Sequencer FLX system (Roche Applied Science). Based on the results of the titration sequencing run, an appropriate amount of the DNA library was used for the emulsion PCR setup. Subsequently, the clonally amplified DNA fragments bound to capture beads were enriched and sequenced on four medium regions of a PicoTiter Plate using standard sequencing chemistry (Roche Applied Science). Upon sequencing and processing of the raw data, a de novo assembly was performed using the GS de novo Assembler software version 2.5.3 with default settings. Gaps between the contigs were filled using designed primer sets and whole plastid genome sequence was obtained. The plastid genome of *Lilium* *longiflorum* and *Alstroemeria* *aurea* were annotated using DOGMA (Dual Organellar GenoMe Annotator, http://dogma.ccbb.utexas.edu/, [34]). Annotation of the transfer RNA gene was performed using DOGMA and the tRNAscan-SE programme (ver. 1.23 [35]). Intron and exon boundaries for intron containing genes were determined by comparison of reference sequences of monocots.

### Taxon and gene sampling, sequence alignment and phylogenetic analysis

The 28 taxa selected here represent eight orders of monocots with two ancestral angiosperms and three gymnosperms as outgroups (Table 1). All of the plastid genome sequences were available in GenBank including the partial coding gene sequences of three species, *Pandanus* *utilis* , *Yucca* *schidigera* , and *Lilium* *superbum* . The character matrix for phylogenetic analysis consisted of the nucleotide sequences of 79 protein-coding genes and all of the pseudogenes were also included in the data matrix (Data File S1), although several gene and intron losses were found. We excluded four ribosomal RNA genes because they affected an obviously incongruent phylogenetic tree of monocots based on the data matrix of gene partitions [29]. They were aligned by MUSCLE [36] and manually adjusted. Both RAxML, BI, and MP analyses were performed on the concatenated 79-gene data set which generated by Geneious R6 (ver. 6.0.5 available from http://www.geneious.com/ [37]). The length of the 79 aligned genes used for phylogenetic analysis was 96,692 bp and the aligned matrix is available from the authors on request. Akaike Information Criterion (AIC) via jModelTest (ver. 0.1.1 [38]) was used to determine the most appropriate substitution model for the full data matrix in addition to 79 separated gene data matrix (Table S1). The RAxML tree was generated with the RAxML BlackBox web-server (ver. 7.2.8 [39]) which performs under the GTR+G model of nucleotide substitution, with gamma distributed rate heterogeneity and a proportion of invariant sites. And also rapid bootstrap was performed with 100 replications. Bayesian inference analysis was performed using MrBayes plug-in [40] in Geneious 6.0.5 [37] with default setting upon the GTR model and gamma rate variations. The MP tree was also constructed by heuristic search and bootstrap was performed with 1000 replicates using PAUP* 4.0b10 [41]. For the heuristic analyses, tree searches were performed with 1000 random sequence additions and tree-bisection-reconnection (TBR) branch swapping, permitting 10 trees to be held at each step to reduce time searching suboptimal ‘islands’ of trees (e.g. [26,42]). Bootstrap analysis [43] used the same settings as above.

**Table 1:** List of taxa used for the phylogenetic analysis of major lineage of monocots.

**Ref. no.**	**Species**	**Family**	**Order**	**Length**
NC010093	*Acorus* *americanus*	Acoraceae	Acorales	153,819
NC007407	*Acorus* *calamus*	Acoraceae	Acorales	153,821
NC010109	*Lemna* *minor*	Araceae	Alismatales	165,955
NC009601	*Dioscorea* *elephantipes*	Dioscoreaceae	Dioscoreales	152,609
HQ180687-HQ183091	*Pandanus* *utilis*	Pandanaceae	Pandanales	unknown
NC014056	*Oncidium Gower*	Orchidaceae	Asparagales	146,484
NC007499	*Phalaenopsis* *aphrodite* subsp. *formosana*	Orchidaceae	Asparagales	148,964
DQ069347-DQ069702	*Yucca* *schidigera*	Asparagaceae	Asparagales	unknown
HM536959	*Smilax* *china*	Smilacaceae	Liliales	157,878
HQ180423-HQ183692	*Lilium* *superbum*	Liliaceae	Liliales	unknown
This study	*Lilium* *longiflorum*	Liliaceae	Liliales	152,789
This study	*Alstroemeria* *aurea*	Alstroemeriaceae	Liliales	155,506
NC013991	*Phoenix* *dactylifera*	Arecaceae	Arecales	158,462
NC015830	*Bambusa* *emeiensis*	Poaceae	Poales	139,493
NC015831	*Ferrocalamus* *rimosivaginus*	Poaceae	Poales	139,467
NC011713	*Festuca* *arundinacea*	Poaceae	Poales	136,048
NC009950	*Lolium* *perenne*	Poaceae	Poales	135,282
NC005973	*Oryza* *nivara*	Poaceae	Poales	134,494
NC008155	*Oryza sativa* (japonica group)	Poaceae	Poales	134,496
NC015826	*Phyllostachys* *nigra* var. *henonis*	Poaceae	Poales	139,839
NC002762	*Triticum aestivum*	Poaceae	Poales	134,545
NC001666	*Zea mays*	Poaceae	Poales	140,384
NC013823	*Typha* *latifolia*	Typaceae	Poales	161,572
NC005086	*Amborella* *trichopoda*	Amborellaceae	Amborellales	162,686
NC006050	*Nymphaea* *alba*	Nymphaeaceae	Nymphaeales	159,930
NC009618	*Cycas* *taitungensis*	Cycadaceae	Cycadales	163,403
NC004677	*Pinus* *koraiensis*	Pinaceae	Pinales	117,190
NC010654	*Welwitschia* *mirabilis*	Welwitschiaceae	Welwitschiales	119,726

### Comparative analyses of plastid genome sequences among the familial and species level in the order Liliales

We compared the plastid genome structures of three families from the order Liliales using the data of *Lilium* *longiflorum* , *Alstroemeria* *aurea* , and *Smilax* *china* [29]. The structural changes in the plastid genome were confirmed by gene content and order comparisons performed using MultiPip-maker [44]. IR boundaries were also described among three families and the substitution rates of 78 genes, which excluded *inf*A gene because of their loss in *Smilax* and *Alstroemeria* , were calculated using DnaSP [45]. We also analysed the tandem repeat sequences distribution in the plastid genome of three families using Tandom Repeat Finder [46]. The sequence variation of 78 plastid genes between two *Lilium* species, *L* *. longiflorum* and *L* *. superbum* , were also confirmed.

## Results

### Plastid genome features of the Easter and Peruvian lily

**Figure 1:**
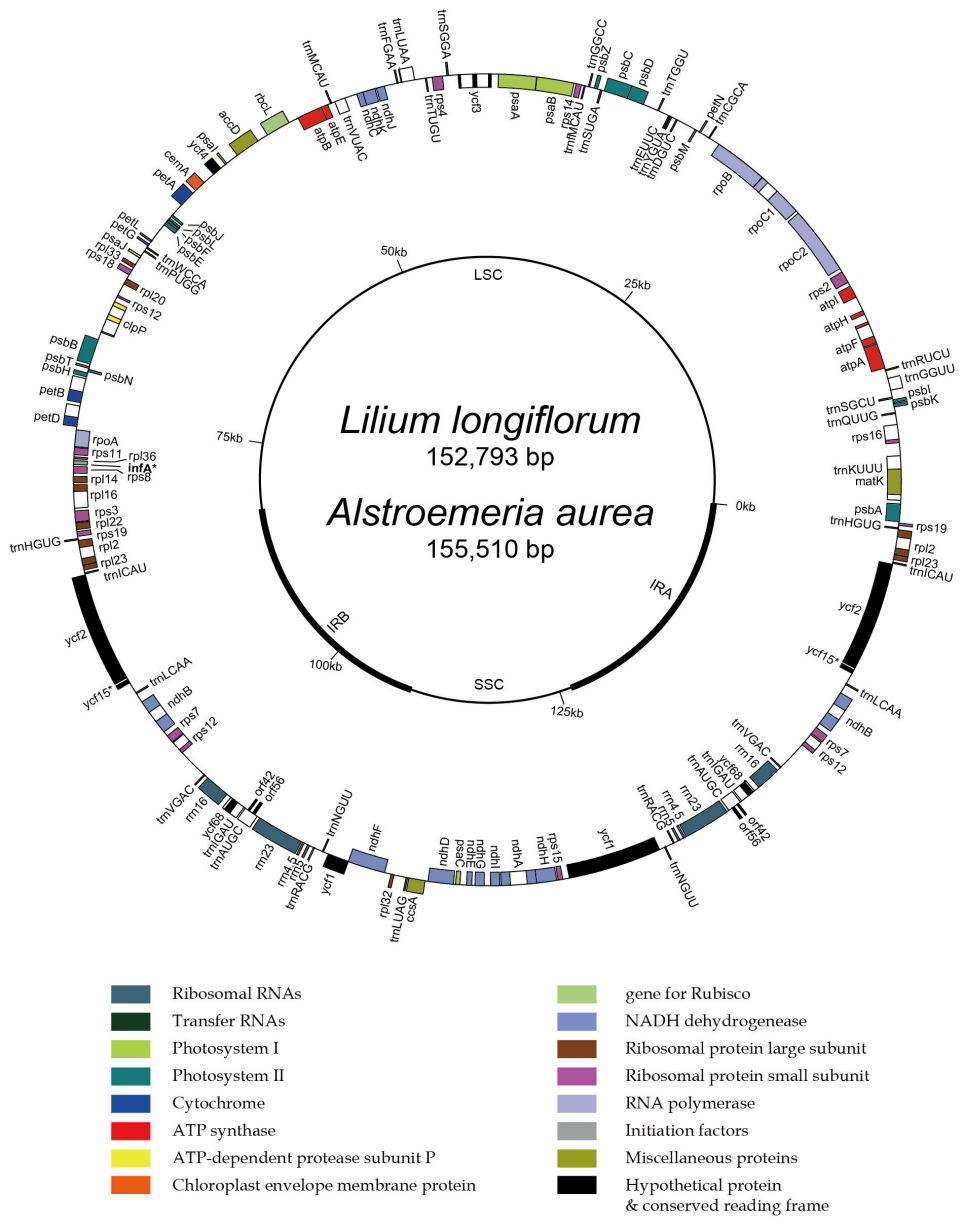
Map of the complete plastid genome of *Lilium* *longiflorum* and *Alstroemeria* *aurea* represented as a circular molecule.

We sequenced the complete plastid genome of the Easter lily ( *Lilium* *longiflorum* , Liliaceae, KC968977) and the Peruvian lily ( *Alstroemeria* *aurea* , Alstroemeriaceae, KC968976). Plastid genome of *L* *. longiflorum* is 152,793 bp in length and composed of LSC region of 82,230 bp, two IR copies of 26,520 bp and SSC region of 17,523 bp (Figure 1). A total of 136 predicted coding regions were detected, 92 of which were different and 22 of which were duplicated in the IR. The coding regions included 47 protein coding genes, 37 transfer RNAs, 4 duplicated ribosomal RNAs, and 26 ribosomal proteins (Table 2). Sixteen genes containing introns are *trn*A-UGC, *trn*I-GAU, *trn*K-UUU, *trn*L-UAA, *pet*B, *pet*D, *atp*F, *ndh*A, *ndh*B, *clp*P, *rpl*2, *rpl*16, *rps*12, *rps*16, *ycf*1 and *ycf*3. Both *clp*P and *ycf*3 were composed of two introns but the others had a single intron. Three genes, *psb*T, *rpl*2 and *ndh*D, possess ACG and *rps*19 has GUG as start codons. *infA* may be a pseudogene consisting of 231 bp modified at the 5’ end.

**Table 2:** List of genes found in *Lilium* *longiflorum* chloroplast genome.

Group of gene			Name of gene*		No.
**RNA genes**	Ribosomal RNAs		rrn4.5(x2), rrn5(x2), rrn16(x2), rrn23(x2)		8
	Transfer RNAs		trnA-UGC^a^(x2), trnC-GCA, trnD-GUC, trnE-UUC, trnF-GAA, trnfM-CAU, trnG-UCC, trnH-GUG(x2), trnI-CAU(x2), trnI-GAU^a^(x2), trnK-UUU^a^, trnL-CAA(x2), trnL-UAA^a^, trnL-UAG, trnM-CAU, trnN-GUU(x2), trnP-UGG, trnQ-UUG, trnR-ACG(x2), trnR-UCU, trnS-GCU, trnS-GGA, trnS-UGA, trnT-GGU, trnT-UGU, trnV-GAC(x2), trnV-UAC^a^, trnW-CCA, trnY-GUA		37
**Protein genes**	Photosynthesis				
	Photosystem I		psaA, psaB, psaC, psaI, psaJ		5
	Photosystem II		psbA, psbB, psbC, psbD, psbE, psbF, psbH, psbI, psbJ, psbK, psbL, psbM, psbN, psbT, psbZ		15
	Cytochrome		petA, petB^a^, petD^a^, petG, petL, petN		6
	ATP synthase		atpA, atpB, atpE, atpF^a^, atpH, atpI		6
	Rubisco		rbcL		1
	NADH dehydrogenease		ndhA^a^, ndhB^a^(x2), ndhC, ndhD, ndhE, ndhF, ndhG, ndhH, ndhI, ndhJ, ndhK		12
	ATP-dependent protease subunit P		clpP^a^		1
	Chloroplast envelope membrane protein		cemA		1
**Ribosomal proteins**	large units		rpl2^a^(x2), rpl14, rpl16^a^, rpl20, rpl22, rpl23(x2), rpl32, rpl33, rpl36		11
	small units		rps2, rps3, rps 4, rps7(x2), rps8, rps11, rps12^a^(x2), rps14, rps15, rps16^a^,rps18, rps19(x2)		15
**Transcription**	RNA polymerase		rpoA, rpoB, rpoC1^a^, rpoC2		4
**/trnslation**	Initiation factor		infA		1
	Miscellaneous proteins		accD, ccsA, matK		3
	Hypothetical proteins & Conserved reading frame		ycf1(x2), ycf2(x2), ycf3^a^, ycf4, ycf15(x2), ycf68(x2)		10
Total					136

* (x2): duplicated genes, ^a^ genes having introns

*A* *. aurea* possess a structurally similar plastid genome to *L* *. longiflorum* and it is consisted of 155,510 bp. It includes LSC region of 84,241 bp, two IRs of 26,701 bp and SSC region of 17,867 bp (Figure 1). It shows the same gene contents and orders excluding the loss of *inf*A and *ycf15*.

### Phylogenetic analyses

**Figure 2:**
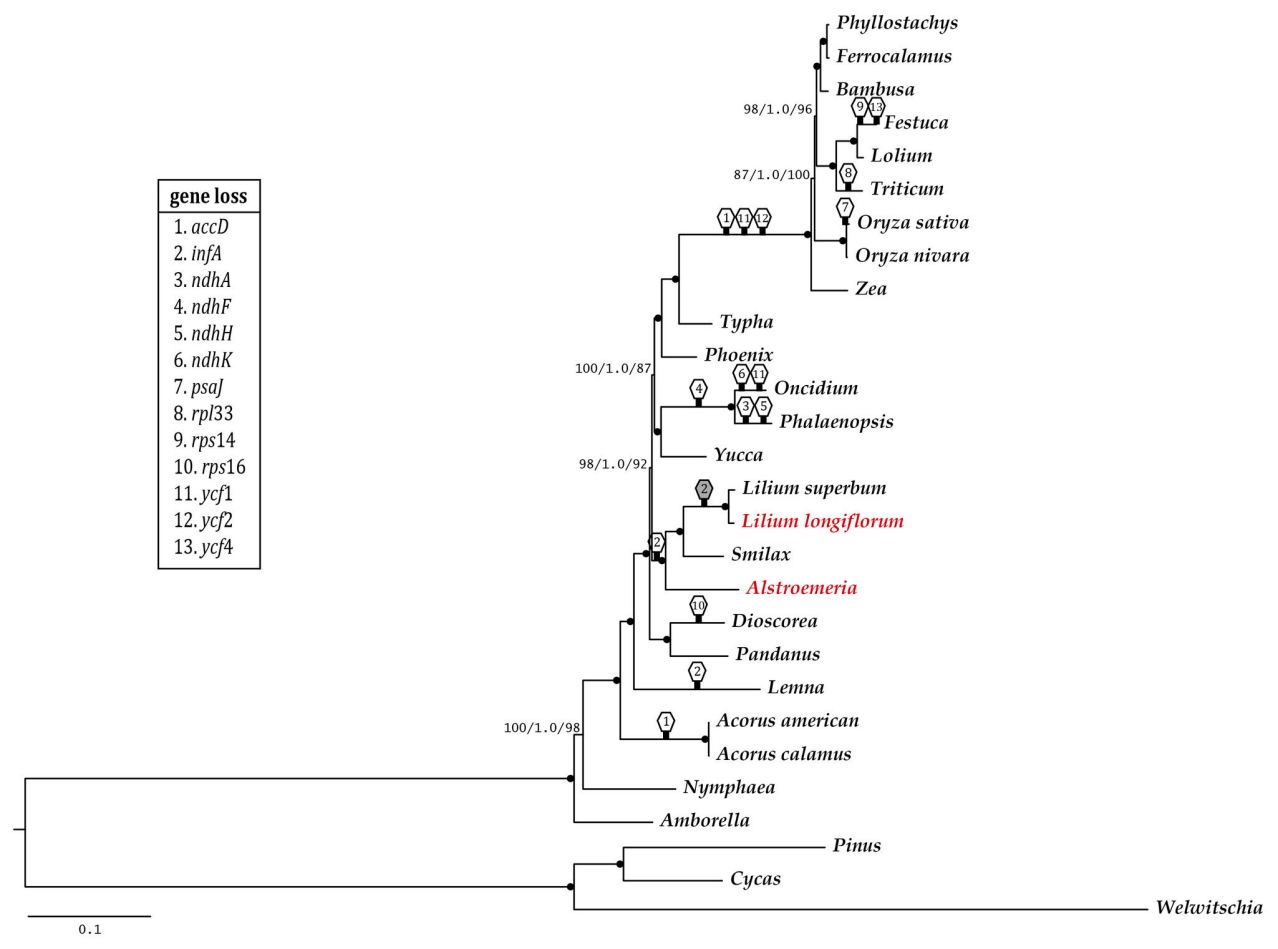
RAxML tree monocot orders using 79 protein-coding genes. Support values for ML, BI and MP are provided at the nodes. A branch with dotted end indicated a high supporting values BP100 in RAxML / PP1.0 in BI, and BP 100 in MP tree and gene loss (sexangle with number) were described on the branch.

Tree topologies for RAxML (-lnL of 564070.1945), Bayesian inference analysis (BI), and the most parsimony analysis (MP) were congruent with each other and all clades were strongly supported in those trees (Figure 2). Monocots were monophyletic with strong support (BP 100 in both the RAxML and MP tree, PP 1.0 in the BI tree) and *Acorus* (order Acorales) was positioned in the basal clade within the monocots. *Lemna* (order Alismatales) was subsequently divergent. *Dioscorea* (order Dioscoreales) and *Pandanus* (order Pandanales) weresister to each other and made a strongly supported clade. A sister relationship between order Liliales and order Asparagales - commelinids clade was also improved (BP 98 in RAxML, PP 100 in BI, and BP 92 in MP). Within Liliales, acloser relationship between Liliaceae and Smilacaceae was formed, rather than Alstroemeriaceae. *Phoenix* (Arecales) was a sister of the Poales clade and Typha (Typhaceae) was located in the basal position in Poales. We constructed a phylogenetic tree according to their substitution model, by a combined data matrix of genes with GTR-model (GTR+G, GTR+I, GTR+I+G, total of them), TVM-model (TVM+G, TVM+I+G, total of them), and K81uf–model (K81uf+G and total of them) generated the RAxML tree, which has the same topology with overall data matrix combined of 79 genes even with the supporting values of branches often decreased (data not shown).

Major gene and intron deletions were found in several orders and are mapped on Figure 2: *accD* in Acorales, *rps16* in Dioscoreales, three genes (*accD*, *ycf1* and *ycf2*) and two introns (*rpoC1* and *clpP*) in Poaceae and *ndhF* and *ndhA* in Orchidaceae of Asparagales ( *Oncidium* has a partial *ndhA* gene). In addition, *ndhK*, *ndhH*, both of *rps14* and *ycf4*, *psaJ* and *rpl33* were lost in *Oncidium* , *Phalaenopsis* , *Festuca* , *O. sativa* and *Triticum* *. infA* was absent in *Lemna* (Alismatales) and Liliales, although *Lilium* has pseudo-*inf*A.

### Comparison of the plastid genome sequences of three families of the order Liliales

**Figure 3:**
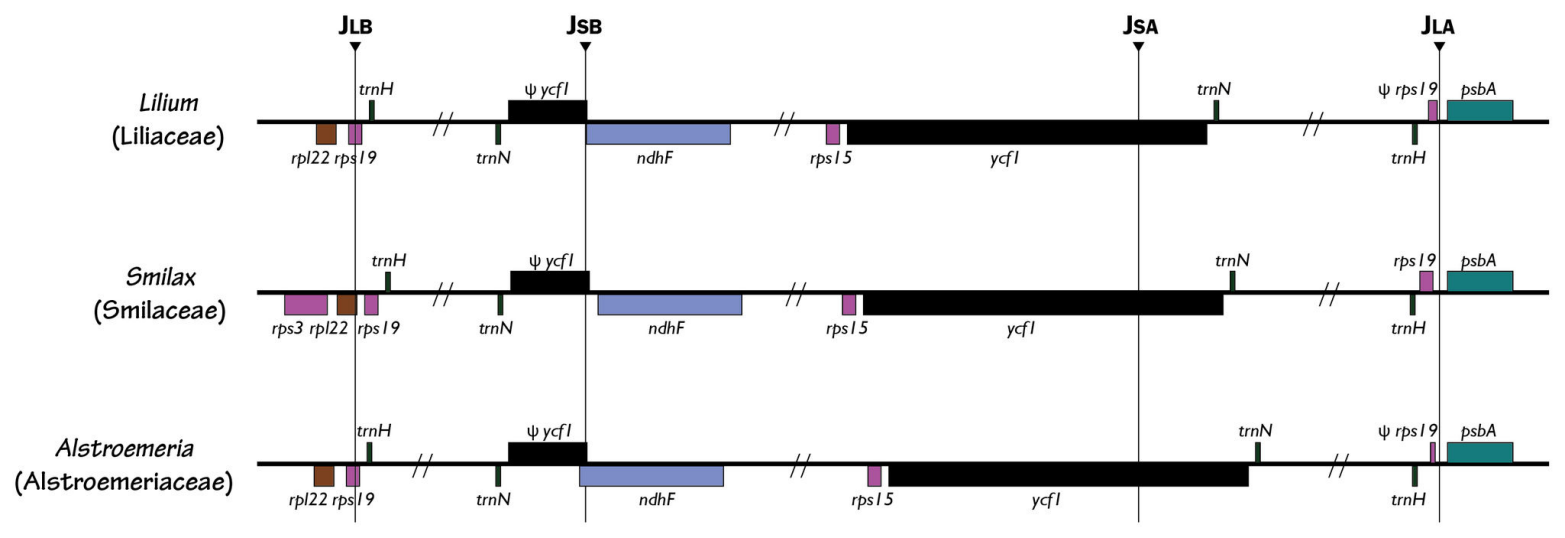
Schemes describing the IR junction in three plastid genomes represent of the family Liliaceae, Smilacaceae, and Alstroemeriaceae of Liliales. The colour of each gene was identical to Figure 1.

Results of whole-genome comparison of three families, Liliaceae, Smilacaceae, and Alstroemeriaceae, revealed similar plastid genome structures and the gene contents were highly conserved within order Liliales (data not shown) except *inf*A, which was deleted in *Smilax* and *Alstroemeria* . They also showed similar identities of 87-88% when compared to the plastid genome sequence of *Phalaenopsis* (Asparagales). Plastid genome of *Smilax* was longer than the other two genomes and it arises from the slightly larger LSC, IRs, and SSC and in their similar G+C contents (Table 3). IR junctions were varied among three families, despite the same expansion pattern in *Lilium* and *Alstroemeria* (Figure 3). Junction of LSC-IRb (JLB in Figure 3) was confirmed at *rps*19 in *Lilium* and *Alstroemeria* , while it was at *rpl*22 in Smilax. IRb–SSC boundary (JSB in Figure 3) was extended to *ndh*F gene only in *Alstroemeria* and 1bp of it was included in *Lilium*. IRa boundaries were positioned near to 3’ end of *ycf*1 (JSA in Figure 3) and with an end point of partial *rps*19 in *Lilium* and *Alstroemeria* , and complete *rps*19 with partial *rpl*22 in *Smilax*, although thses was not annotated in their original submission (JLA in Figure 3).

**Table 3:** Comparison of the plastid genome sequences of 3 families in Liliales.

Species(Family)	*Lilium longiflorum* (Liliaceae)	*Smilax china* (Smilacaceae)	*Alstroemeria aurea (Alstroemeriaceae)*
Total length (bp)	152,793	157,878	155,510
LSC (bp)	82,230	84,608	84,241
SSC (bp)	17,523	18,536	17,867
IRs (bp)	26,520	27,367	26,701
% of G+C	37.02	37.26	38.05
% of A+T	62.98	62.74	61.95
Conserved region compare to *Phalaenopsis* (Asparagales) plastid genome	123,888bp (88.0%)	124,907 (88.4%)	127,738bp (87.5%)

**Figure 4:**
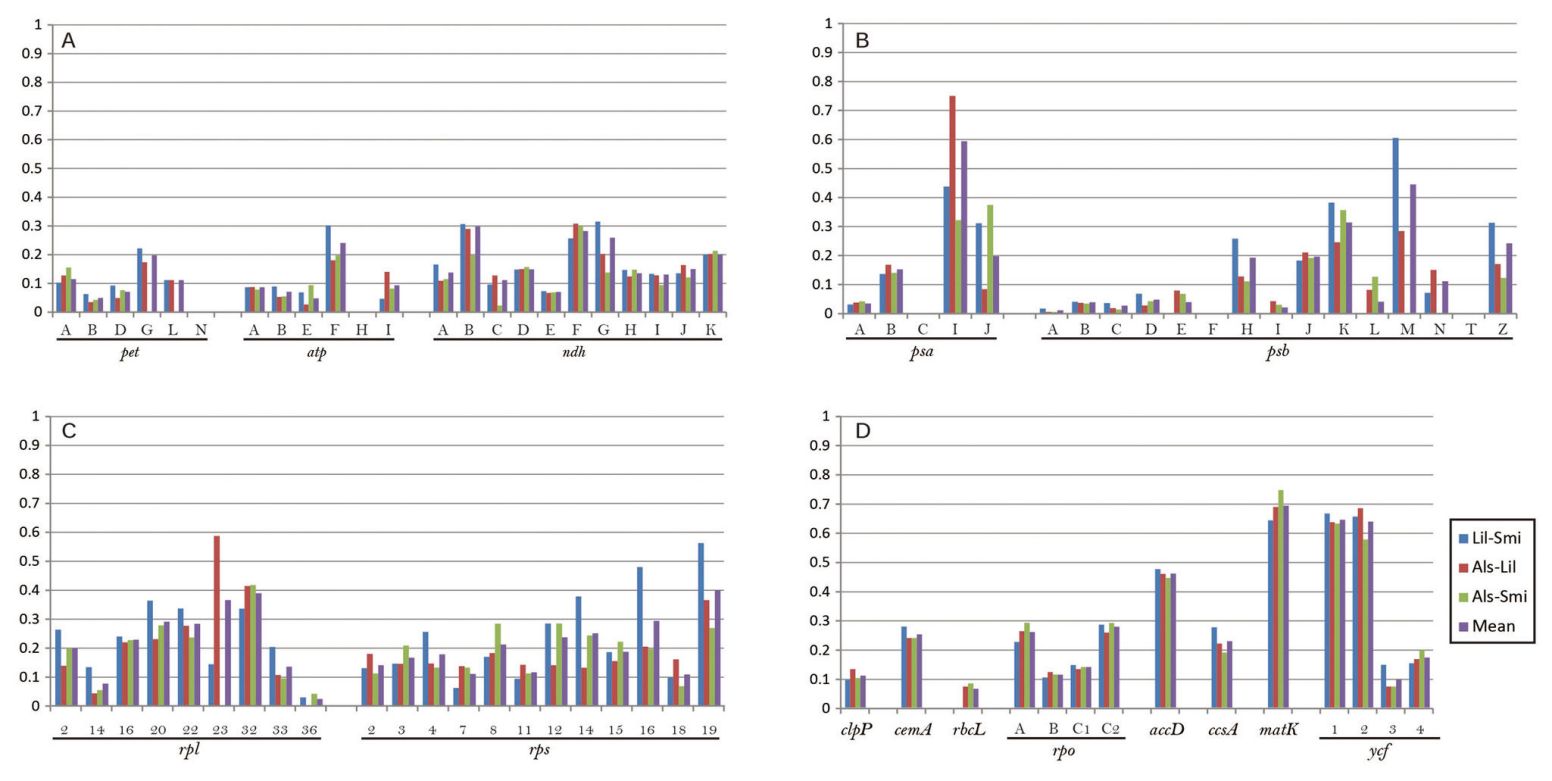
Ka/Ks ratio of pair-wise comparison among the three families of Liliales according to the function of each gene. A) genes encoding cytochrome, ATP synthase, and NADH hydrogenease, B) genes encoding Photosynthesis, C) genes encoding ribosomal protein, D) the other genes.

Both substitution per synonymous sites (Ks), non-synonymous sites (Ka) and their ratio (Ka/Ks) were calculated in 78 genes of three families as well as sequence variations (Table S2). Ks was highest at *psa*J in comparison of *Lilium* vs *Smilax* (0.4751), and at *rpl*36 in *Lilium* vs *Alstroemeria* and *Smilax* vs *Alstroemeria* (0.5319 and 0.5445). Ka was highest at *psa*J in comparison of *Lilium* vs *Smilax* and *Smilax* vs *Alstroemeria* (0.1478 and 0.1751), and at *mat*K in *Lilium* vs *Alstroemeria* (0.1539). However, Ka/Ks ratio was highest at *mat*K in comparison of *Lilium* vs *Smilax* and *Smilax* vs *Alstroemeria* (0.6405 and 0.7439), and at *psa*I in *Lilium* vs *Alstroemeria* (0.7498). Average Ka/Ks ratio was 0.1938, 0.1744, and 0.1611 in comparison of *Lilium* vs *Smilax*, *Lilium* vs *Alstroemeria* and *Smilax* vs *Alstroemeria* , respectively. Each Ka/Ks ratio of 78 genes in three different combinations were compared in Figure 4 based on the group of genes. The most variable genes over 13% of variation were *psa*J (20.74%), *ycf*1 (18.13%), *rpl*32 (17.82%), *rpl*22 (17.42%), *mat*K (16.7%), *ccs*A (15.87%), *psb*K (15.63%), *ndh*F (14.19%), *rps*15 (13.92%), *rpo*C2 (13.36%), and *rpl*36 (13.16%). Out of them, the four genes *mat*K, *ccs*A, *rpo*C2, and *ycf*1 were longer than 1,000bp in total length (Table S2).

21 tandom repeat sequences were found (75bp in maximum within *ndh*E) over 20bp in *Smilax* plastid genome, 11 repeats (48bp in maximum within *pet*B intron) in *Alstroemeria* , and 9 repeats (162bp in maximum between *rps*12-*clp*P IGS) in *Lilium* (data not shown). Most of the repeat sequences were found in the non-coding regions.

### Sequence variations between two *Lilium* species

Inter-specific sequence variation was analyzed in 78 genes, excluding *cem*A which was absent in the database of *L* *. superbum* (Table S3). Our analysis included partial sequences for *L* *. superbum* because of its incomplete sequence information. The variation ratio was calculated among truly aligned sequences between two species. We described sequence information of the ten most variable genes in Table 4 and it was highest in *rps*15 with 3.66% variation. Most of them are comprised of less than 1,000bp except three genes *rpo*A, *mat*K, and *ndh*F. In contrast, 11 genes were completely conserved, *pet*N, *psa*I, *psa*J, *psb*F, *psb*J, *psb*L, *psb*T, *psb*Z, *rpl*23, *rpl* 32, and *rps*7. We found that some of the genes have indel sequences. 6bp insertion (TTGGCG) of repeat sequence was found in *L* *. longiflorum* compared with *L* *. superbum* . This similar pattern was also distributed in *inf*A and another repeat sequence composed 6bp (CTTTTA) was deleted in *L* *. longflorum* . 6bp insertion (CTTTAG) and 13bp deletion (ATATCTATTTTGATGATAGTGACA) were also detected in *rpl*20 and *ycf*2, respectively. In *ndh*G, eight T of poly-T region was deleted (Table S3).

**Table 4:** Most variable 10 genes in comparison of *Lilium* *longiflorum* vs *Lilium* *superbum* .

gene name	total length	no. of variable sites		% of variable site	indel (type)
*rps*15	273	10		3.66	
*pet*G	114	4		3.51	
*rpl*36	114	3		2.63	
*inf*A	231	5		2.16	CTTTTA (del/repeat)
*rps*3	657	12		1.83	
*rps*14	303	5		1.65	
*rpo*A	1008	16		1.59	
*mat*K	1539	24		1.56	
*ndh*F	2223	32		1.44	
*rps*19	279	4		1.43	

## Discussion

Here, we analyzed the complete plastid genome sequences of two important ornamental lily species ( *Lilium* *longflorum* and *Alstroemeria* *aurea* ) of the order Liliales and it allowed us to solve the confused sister relationship of this order. We also achieved a comparative analysis in inter-familial and inter-specific variation of true lilied plastid genomes.

### Sister relationship of the order Liliales in monocots

Phylogenetic analyses of 79 plastid protein-coding genes produced a well-resolved phylogeny of seven monocot orders and it was mostly congruent with the results of Chase and colleagues [26]. The basal position of Acorales (Acorus) and the subsequent divergence of Alismatales (*Lemna*) were reconfirmed. These two basal orders are commonly aquatic or emergent aquatic [23]. Petrosaviales, which was recognised in APG III [25], was regarded as a sister of the lilied/commelinid clade [26,27], but unfortunately, it was not included in this study. In recent phylogenetic studies using plastid IR sequences, these relationships were also weakly supported as the Liliales and Asparagales+commelinids clade with BP 68 and Asparagales and commelinids clade with BP 53 [28]. Givnish and colleagues [27] introduced a well-defined relationship of monocots using a ML tree composed of branches with strong support based on 81 plastid genes. Although they focused on the phylogenetic relationship of Poales and a MP tree using the same data matrix, they showed a different topology with a closer relationship between Dioscoreales and Asparagales. The sister relationships among the Dioscorelaes-Pandanales, Liliales, Asparagales, and commelinids clade, which showed the rapid divergence among the groups, were well supported. However, the contradiction between poor resolution and closely related group around Liliales still remained even though an attempt using various data matrix of chloroplast genes [29]. Our phylogenetic tree revealed a strong sister relationship of Liliales to the Asparagales+commelinids clade with improved resolution compared to the previous phylo-genomic study [29] using *Smilax* and *L* *. superbum* data as a representatives of Liliales. Additionally, in Poales, Typhaceae was located on the root of the order and Poaceae showed a well-resolved phylogenetic relationship as the subfamily Panicoideae [Ehrhartoideae (Bambusoideae+Pooideae)]. No conflict was observed compared to previous studies of Poales [11,13].

Gene loss events in major clade of monocots was congruent with the previous studies [22,29,47], except *inf*A gene loss in Liliales, although it was present in *Lilium* as a pseudo-gene.

### Inter-familial variation of plastid genome structure in Liliales

The gene contents and orders in the plastid genome were congruent among three families, except *inf*A loss in *Smilax* and *Alstroemeria* . If unclear IR boundary information is given, incorrect gene order phylogenies are recovered. Therefore, the maintenance of the IR is necessary in the evolution of chloroplast genomes in most cases. Yue and colleagues [48] proposed that the IR provides an insulation mechanism that stabilizes the genome structure and the genes in single copy regions do not commute across the IR. Generally, IRs of monocots contained a *trnH-rps19* gene cluster near the IRa–LSC junction; moreover, they expand more progressively than non-monocot angiosperms [49]. They explained that a double-strand break (DSB) event first occurred at IRb and led to the expansion of the IR to *trnH*, followed by a successive DSB event within IRa leading to the expansion of the IR to *rps19* or to *rpl22*. Results comparing IR expansion among three families of Liliales showed that they have a typical JLA of monocots with a *trnH-rps19* cluster. However, JLB was extended to rpl22 in *Smilax* plastid genome. Moreover, we found that JSB was moved to *ndh*F gene in *Alstroemeria* plastid genome (Figure 3) which explains the length variation in the total sequences of three different plastid genomes, as well as a length variation in inter-genic spacer region (Table 3). Ka/Ks ratios according to the pair-wise comparison of substitution among three families were different to the gene partition using whole data of major monocots lineages of Liu et al. [29]. From the results, we suggest that *ccs*A, *mat*K, *ndh* gene series (A, B, D, F, and H), *rpoA*, and *rbc*L will be suitable genes for phylogenetic study of monocots concerning available length using PCR amplification reaction and proportion of variable sites, though *ndh* genes were often missing especially in some lineage of Asparagales. Most of repeat sequences were distributed in the non-coding regions of inter-genic spacer or intron. Although, many of the repeat sequences were in *Smilax*, and the largest repeat, over 100bp, was uniquely found in *Lilium*.

### *infA* divergence among three families

Millen and colleagues [50] suggested that *infA*, which codes for translation initiation factor 1, has been entirely lost or has become a pseudogene *ca* 24 separate times in 309 angiosperms. In four species, this gene was regarded to be transferred from chloroplast to nuclear DNA independently during angiosperm evolution. According to their results, this parallel event occurred in *Tricyrtis* and *Smilax*, a member of Liliales. Our data revealed that this event also occurred in *Alstroemeria* , which is closer to the basal Liliales than *Lilium* or *Smilax*. Interestingly, even in the *Lilium* plastid genome, *infA* seems to have lost its function because it has AAT instead of AGT in the start codon position and includes two premature stop codons, although we do not know what kind of mutation has occurred in the start position of *infA*. Further study is needed to improve our understanding of *infA* gene evolution in Liliales.

### Inter-specific variation between two *Lilium* species

Results of the complete plastid genome sequence of two lilies in this study make it possible to analyse the details of sequence variation in species level although it was restricted just in the coding region. We compared the variation of 78 plastid genes between *L* *. longiflorum* and *L* *. superbum* , which has been used as a representative of the order Liliales until this time. 11genes of 78 genes were conserved between two species, 19 genes were variable over 1% in the entire aligned sequence, and 5 indels, including two repeat sequences composed of 6bp, were also found. The result will provide an informative guide line for recognizing the species and genus of the order Liliales in the near future when more sequence data is accumulated.

Although there was a limitation of material amount, we describe here a simple method for extracting plastid DNA with a simple buffer composition, in the hope that it will be applied in monocot plastome research, which has already been applied in some of the Asparagales members, for example, Asparagaceae, Alliaceae, and Orchidaceae.

## Supporting Information

Figure S1 Electrophoretogram of isolated plastid DNA in the present study. M) lambda-HindIII digest, Lil) *Lilium* *longiflorum* (268 ng/µl), Als) *Alstroemeria* *aurea* (314 ng/µl).
(TIF)

Table S1 Substitution models for each gene used in the phylogenetic study. (DOCX)

Table S2 Substitution rates and sequence variations among 3 families of Liliales. (DOCX)

Table S3 Comparison of the sequence variation between two *Lilium* species. (DOCX)

Data File S1 Aligned matrix of combined 79 genes for the present study. (DOCX)
