# Supplementary figures and images for: Comparative Genome Analysis and Phylogenetic Relationship of Order Liliales Insight from the Complete Plastid Genome Sequences of Two Lilies (Lilium longiflorum and Alstroemeria aurea)
Source: PLoS One. 2013 Jun 18;8(6):e68180. doi: 10.1371/journal.pone.0068180 (PMC3688979; doi:10.1371/journal.pone.0068180)

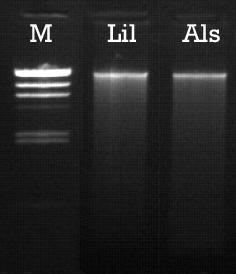

Supplement: Figure S1 — M) lambda-HindIII digest, Lil) Lilium longiflorum (268 ng/µl), Als) Alstroemeria aurea (314 ng/µl). (TIF) [file pone.0068180.s001.tif]
