# Supplementary material for: Comparative Genome Analysis and Phylogenetic Relationship of Order Liliales Insight from the Complete Plastid Genome Sequences of Two Lilies (Lilium longiflorum and Alstroemeria aurea)
Source: PLoS One. 2013 Jun 18;8(6):e68180. doi: 10.1371/journal.pone.0068180 (PMC3688979; doi:10.1371/journal.pone.0068180)
Supplement: Table S1 — (DOCX) [file pone.0068180.s002.docx]

Table S1. Substitution models for each gene used in the phylogenetic study.

| **Model** | **Genes** |
| --- | --- |
| TVM+I+G | accD, atpF, atpI, ccsA, cemA, matK, ndhF, ndhI, petA, psaB, psbK, rpl16, rps3, rps11, rps12, ycf4 |
| TVM+I | petN, rps7 |
| TVM+G | atpE, ndhC, ndhD, ndhE, ndhH, ndhK, psbH, psbI, psbJ, psbZ, rpl20, rpl22, rpl23, rps4, rps8, rps14, rps15, rps18, rps19, ycf2 |
| TIM+I+G | petD |
| TrN+G | psaJ |
| K81uf+I+G | atpH |
| K81uf+I | petG , psbT |
| K81uf+G | clpP, petL, psbE, psbF, psbL, rpl2 |
| HKY+I | psbN |
| HKY+G | psaI |
| GTR+I+G | atpA, atpB, ndhB, petB, psaA, psbA, psbB, psbC, psbD, rbcL, rpl14, rpl32, rpoB, rpoC1, rpoC2, rps16, ycf1, ycf3 |
| GTR+I | psbM |
| GTR+G | infA, ndhA, ndhG, ndhJ, psaC, rpl32, rpl33, rpoA, rps2 |
