# Supplementary material for: Comparative Genome Analysis and Phylogenetic Relationship of Order Liliales Insight from the Complete Plastid Genome Sequences of Two Lilies (Lilium longiflorum and Alstroemeria aurea)
Source: PLoS One. 2013 Jun 18;8(6):e68180. doi: 10.1371/journal.pone.0068180 (PMC3688979; doi:10.1371/journal.pone.0068180)
Supplement: Table S2 — (DOCX) [file pone.0068180.s003.docx]

| Table S2. Substitution rates and sequence variations among 3 families of Liliales. | | | | | | | | | | | | | |
| --- | --- | --- | --- | --- | --- | --- | --- | --- | --- | --- | --- | --- | --- |
| GENE | Ks | | | Ka | | | Ka/Ks | | | Total length | singleton variable sites | | % of variable sites |
|  | Lil. vs Smi. | Lil. vs Als. | Smi. vs Als. | Lil. vs Smi. | Lil. vs Als. | Smi. vs Als. | Lil. vs Smi. | Lil. vs Als. | Smi. vs Als. |  | between 2sp. | among 3sp. |  |
| accD | 0.1395 | 0.1627 | 0.1970 | 0.0660 | 0.0744 | 0.0873 | 0.4731 | 0.4573 | 0.4431 | 1506 | 182 | 6 | **12.48** |
| atpA | 0.1933 | 0.2330 | 0.2763 | 0.0167 | 0.0203 | 0.0216 | 0.0864 | 0.0871 | 0.0782 | 1524 | 140 | 4 | 9.45 |
| atpB | 0.1397 | 0.2745 | 0.2626 | 0.0125 | 0.0144 | 0.0144 | 0.0895 | 0.0525 | 0.0548 | 1503 | 125 | 4 | 8.58 |
| atpE | 0.2436 | 0.3750 | 0.2144 | 0.0168 | 0.0100 | 0.0201 | 0.0690 | 0.0267 | 0.0938 | 408 | 38 | 2 | 9.80 |
| atpF | 0.1464 | 0.2998 | 0.2674 | 0.0442 | 0.0540 | 0.0529 | 0.3019 | 0.1801 | 0.1978 | 555 | 63 | 4 | **12.07** |
| atpH | 0.0954 | 0.2047 | 0.1853 | 0.0000 | 0.0000 | 0.0000 | 0.0000 | 0.0000 | 0.0000 | 246 | 13 | 1 | 5.69 |
| atpI | 0.1525 | 0.1859 | 0.2291 | 0.0071 | 0.0261 | 0.0188 | 0.0466 | 0.1404 | 0.0821 | 744 | 55 | 3 | 7.80 |
| ccsA | 0.2146 | 0.3749 | 0.4206 | 0.0588 | 0.0819 | 0.0791 | 0.2740 | 0.2185 | 0.1881 | 1002 | 146 | 13 | **15.87** |
| cemA | 0.1659 | 0.2948 | 0.2015 | 0.0459 | 0.0699 | 0.0478 | 0.2767 | 0.2371 | 0.2372 | 690 | 80 | 3 | **12.03** |
| clpP | 0.2271 | 0.1809 | 0.2364 | 0.0215 | 0.0237 | 0.0237 | 0.0947 | 0.1310 | 0.1003 | 615 | 56 | 0 | 9.11 |
| matK | 0.1555 | 0.2242 | 0.1976 | 0.0996 | **0.1539** | 0.1470 | **0.6405** | 0.6864 | **0.7439** | 1617 | 258 | 12 | **16.70** |
| ndhA | 0.2076 | 0.3161 | 0.2942 | 0.0344 | 0.0344 | 0.0338 | 0.1657 | 0.1088 | 0.1149 | 1092 | 124 | 7 | **12.00** |
| ndhB | 0.0215 | 0.0549 | 0.0591 | 0.0066 | 0.0159 | 0.0117 | 0.3070 | 0.2896 | 0.1980 | 2358 | 61 | 1 | 2.63 |
| ndhC | 0.1137 | 0.1137 | 0.1551 | 0.0109 | 0.0145 | 0.0036 | 0.0959 | 0.1275 | 0.0232 | 363 | 19 | 0 | 5.23 |
| ndhD | 0.2079 | 0.3328 | 0.2964 | 0.0309 | 0.0498 | 0.0466 | 0.1486 | 0.1496 | 0.1572 | 1512 | 181 | 9 | **12.57** |
| ndhE | 0.2362 | 0.3230 | 0.2838 | 0.0173 | 0.0216 | 0.0196 | 0.0732 | 0.0669 | 0.0691 | 381 | 29 | 2 | 8.14 |
| ndhF | 0.2109 | 0.2482 | 0.2630 | 0.0540 | 0.0765 | 0.0787 | 0.2560 | 0.3082 | 0.2992 | 2283 | 307 | 17 | **14.19** |
| ndhG | 0.1568 | 0.2108 | 0.2727 | 0.0495 | 0.0427 | 0.0374 | 0.3157 | 0.2026 | 0.1371 | 534 | 59 | 2 | **11.42** |
| ndhH | 0.1816 | 0.2426 | 0.2764 | 0.0267 | 0.0300 | 0.0409 | 0.1470 | 0.1237 | 0.1480 | 1182 | 119 | 3 | **10.32** |
| ndhI | 0.2199 | 0.2971 | 0.2976 | 0.0294 | 0.0381 | 0.0281 | 0.1337 | 0.1282 | 0.0944 | 549 | 60 | 1 | **11.11** |
| ndhJ | 0.1510 | 0.2025 | 0.2047 | 0.0205 | 0.0332 | 0.0247 | 0.1358 | 0.1640 | 0.1207 | 477 | 36 | 3 | 8.18 |
| ndhK | 0.1818 | 0.2658 | 0.2245 | 0.0365 | 0.0538 | 0.0479 | 0.2008 | 0.2024 | 0.2134 | 878 | 85 | 2 | 9.91 |
| petA | 0.1873 | 0.2172 | 0.1985 | 0.0193 | 0.0277 | 0.0308 | 0.1030 | 0.1275 | 0.1552 | 963 | 87 | 1 | 9.14 |
| petB | 0.1311 | 0.1767 | 0.1928 | 0.0082 | 0.0062 | 0.0082 | 0.0625 | 0.0351 | 0.0425 | 648 | 41 | 0 | 6.33 |
| petD | 0.1491 | 0.1699 | 0.1641 | 0.0138 | 0.0083 | 0.0125 | 0.0926 | 0.0489 | 0.0762 | 528 | 32 | 0 | 6.06 |
| petG | 0.1606 | 0.2054 | 0.3041 | 0.0356 | 0.0357 | 0.0000 | 0.2217 | 0.1738 | 0.0000 | 114 | 11 | 0 | 9.65 |
| petL | 0.1280 | 0.1280 | 0.0000 | 0.0143 | 0.0143 | 0.0000 | 0.1117 | 0.1117 | N/A | 96 | 4 | 0 | 4.17 |
| petN | 0.1613 | 0.2239 | 0.2921 | 0.0000 | 0.0000 | 0.0000 | 0.0000 | 0.0000 | 0.0000 | 90 | 6 | 0 | 6.67 |
| psaA | 0.1647 | 0.2299 | 0.2189 | 0.0052 | 0.0087 | 0.0093 | 0.0316 | 0.0378 | 0.0425 | 2253 | 155 | 4 | 7.06 |
| psaB | 0.1083 | 0.1489 | 0.1739 | 0.0148 | 0.0250 | 0.0244 | 0.1367 | 0.1679 | 0.1403 | 2206 | 139 | 8 | 6.66 |
| psaC | 0.2274 | 0.4164 | 0.2743 | 0.0000 | 0.0000 | 0.0000 | 0.0000 | 0.0000 | 0.0000 | 246 | 21 | 0 | 8.54 |
| psaI | 0.0897 | 0.1159 | 0.2233 | 0.0393 | 0.0869 | 0.0719 | 0.4381 | **0.7498** | 0.3220 | 111 | 13 | 0 | **11.71** |
| psaJ | **0.4751** | 0.2635 | 0.4678 | **0.1478** | 0.0222 | **0.1751** | 0.3111 | 0.0843 | 0.3743 | 135 | 27 | 1 | **20.74** |
| psbA | 0.1459 | 0.2020 | 0.2020 | 0.0025 | 0.0012 | 0.0012 | 0.0171 | 0.0059 | 0.0059 | 1062 | 61 | 1 | 5.84 |
| psbB | 0.1683 | 0.2585 | 0.2688 | 0.0069 | 0.0095 | 0.0091 | 0.0410 | 0.0368 | 0.0339 | 1527 | 119 | 3 | 7.99 |
| psbC | 0.1820 | 0.3010 | 0.2717 | 0.0066 | 0.0056 | 0.0037 | 0.0363 | 0.0186 | 0.0136 | 1422 | 113 | 4 | 8.23 |
| psbD | 0.1440 | 0.2008 | 0.2299 | 0.0099 | 0.0056 | 0.0099 | 0.0688 | 0.0279 | 0.0431 | 1063 | 62 | 2 | 6.02 |
| psbE | 0.0913 | 0.1314 | 0.1532 | 0.0000 | 0.0104 | 0.0104 | 0.0000 | 0.0791 | 0.0679 | 252 | 12 | 0 | 4.76 |
| psbF | 0.0678 | 0.0678 | 0.0671 | 0.0000 | 0.0000 | 0.0000 | 0.0000 | 0.0000 | 0.0000 | 120 | 3 | 0 | 2.50 |
| psbH | 0.1656 | 0.2882 | 0.2778 | 0.0428 | 0.0367 | 0.0307 | 0.2585 | 0.1273 | 0.1105 | 222 | 26 | 0 | **11.71** |
| psbI | 0.1253 | 0.2758 | 0.3961 | 0.0000 | 0.0119 | 0.0119 | 0.0000 | 0.0431 | 0.0300 | 111 | 8 | 1 | 8.11 |
| psbJ | 0.0619 | 0.1641 | 0.2414 | 0.0113 | 0.0345 | 0.0463 | 0.1826 | 0.2102 | 0.1918 | 123 | 11 | 0 | 8.94 |
| psbK | 0.1709 | 0.3002 | 0.2965 | 0.0654 | 0.0736 | 0.1056 | 0.3827 | 0.2452 | 0.3562 | 192 | 29 | 1 | **15.63** |
| psbL | 0.0417 | 0.1327 | 0.0858 | 0.0000 | 0.0109 | 0.0109 | 0.0000 | 0.0821 | 0.1270 | 117 | 4 | 0 | 3.42 |
| psbM | 0.0852 | 0.1813 | 0.0846 | 0.0516 | 0.0516 | 0.0000 | 0.6056 | 0.2846 | 0.0000 | 105 | 8 | 0 | 7.62 |
| psbN | 0.1391 | 0.0663 | 0.1375 | 0.0100 | 0.0100 | 0.0000 | 0.0719 | 0.1508 | 0.0000 | 132 | 6 | 0 | 4.55 |
| psbT | 0.1900 | 0.0432 | 0.1378 | 0.0000 | 0.0000 | 0.0000 | 0.0000 | 0.0000 | 0.0000 | 108 | 4 | 0 | 3.70 |
| psbZ | 0.0914 | 0.1674 | 0.1152 | 0.0286 | 0.0286 | 0.0142 | 0.3129 | 0.1708 | 0.1233 | 189 | 13 | 0 | 6.88 |
| rbcL | 0.1766 | 0.2800 | 0.2761 | 0.0064 | 0.0200 | 0.0228 | 0.0362 | 0.0714 | 0.0826 | 1464 | 129 | 3 | 9.02 |
| rpl2 | 0.0279 | 0.0280 | 0.0375 | 0.0058 | 0.0102 | 0.0132 | 0.2079 | 0.3643 | 0.3520 | 822 | 33 | 1 | 4.14 |
| rpl14 | 0.1352 | 0.3326 | 0.3658 | 0.0181 | 0.0144 | 0.0198 | 0.1339 | 0.0433 | 0.0541 | 369 | 36 | 1 | **10.03** |
| rpl16 | 0.1268 | 0.2016 | 0.1637 | 0.0304 | 0.0442 | 0.0372 | 0.2397 | 0.2192 | 0.2272 | 408 | 33 | 4 | 9.07 |
| rpl20 | 0.1280 | 0.2617 | 0.2384 | 0.0465 | 0.0604 | 0.0663 | 0.3633 | 0.2308 | 0.2781 | 366 | 46 | 0 | **12.57** |
| rpl22 | 0.2465 | 0.3863 | 0.3404 | 0.0829 | 0.1069 | 0.0805 | 0.3363 | 0.2767 | 0.2365 | 396 | 68 | 1 | **17.42** |
| rpl23 | 0.0319 | 0.0317 | 0.0000 | 0.0046 | 0.0186 | 0.0139 | 0.1442 | 0.5868 | N/A | 282 | 6 | 0 | 2.13 |
| rpl32 | 0.2072 | 0.2455 | 0.2977 | 0.0696 | 0.1016 | 0.1243 | 0.3359 | 0.4138 | 0.4175 | 174 | 31 | 0 | **17.82** |
| rpl33 | 0.0967 | 0.3099 | 0.2764 | 0.0197 | 0.0330 | 0.0263 | 0.2037 | 0.1065 | 0.0952 | 204 | 18 | 1 | 9.31 |
| rpl36 | 0.3944 | **0.5319** | **0.5445** | 0.0115 | 0.0000 | 0.0230 | 0.0292 | 0.0000 | 0.0422 | 114 | 14 | 1 | **13.16** |
| rpoA | 0.1858 | 0.1919 | 0.1914 | 0.0416 | 0.0500 | 0.0554 | 0.2239 | 0.2606 | 0.2894 | 1030 | 106 | 4 | **10.68** |
| rpoB | 0.1579 | 0.2215 | 0.2335 | 0.0162 | 0.0269 | 0.0262 | 0.1026 | 0.1214 | 0.1122 | 3213 | 268 | 10 | 8.65 |
| rpoC1 | 0.1304 | 0.2464 | 0.2636 | 0.0189 | 0.0323 | 0.0366 | 0.1449 | 0.1311 | 0.1388 | 2082 | 189 | 9 | 9.51 |
| rpoC2 | 0.1657 | 0.2800 | 0.2415 | 0.0475 | 0.0717 | 0.0697 | 0.2836 | 0.2561 | 0.2886 | 4198 | 538 | 23 | **13.36** |
| rps2 | 0.1773 | 0.2302 | 0.2822 | 0.0231 | 0.0414 | 0.0316 | 0.1303 | 0.1798 | 0.1120 | 711 | 72 | 1 | **10.27** |
| rps3 | 0.1772 | 0.1932 | 0.2028 | 0.0259 | 0.0280 | 0.0423 | 0.1462 | 0.1449 | 0.2086 | 657 | 58 | 2 | 9.13 |
| rps4 | 0.1036 | 0.2194 | 0.2165 | 0.0265 | 0.0321 | 0.0287 | 0.2558 | 0.1463 | 0.1326 | 606 | 54 | 0 | 8.91 |
| rps7 | 0.0451 | 0.0833 | 0.0642 | 0.0028 | 0.0114 | 0.0085 | 0.0621 | 0.1369 | 0.1324 | 468 | 13 | 1 | 2.99 |
| rps8 | 0.1594 | 0.2153 | 0.1325 | 0.0270 | 0.0393 | 0.0376 | 0.1694 | 0.1825 | 0.2838 | 399 | 33 | 3 | 9.02 |
| rps11 | 0.1744 | 0.2592 | 0.2349 | 0.0164 | 0.0367 | 0.0264 | 0.0940 | 0.1416 | 0.1124 | 417 | 39 | 3 | **10.07** |
| rps12 | 0.0279 | 0.0280 | 0.0375 | 0.0058 | 0.0102 | 0.0132 | 0.2079 | 0.3643 | 0.3520 | 918 | 20 | 0 | 2.18 |
| rps14 | 0.0927 | 0.1984 | 0.1807 | 0.0350 | 0.0261 | 0.0439 | 0.3776 | 0.1316 | 0.2429 | 303 | 25 | 1 | 8.58 |
| rps15 | 0.2338 | 0.3797 | 0.2863 | 0.0434 | 0.0587 | 0.0633 | 0.1856 | 0.1546 | 0.2211 | 273 | 36 | 2 | **13.92** |
| rps16 | 0.1036 | 0.2436 | 0.2239 | 0.0497 | 0.0499 | 0.0440 | 0.4797 | 0.2048 | 0.1965 | 261 | 27 | 1 | **10.73** |
| rps18 | 0.1756 | 0.2155 | 0.2854 | 0.0172 | 0.0346 | 0.0193 | 0.0979 | 0.1606 | 0.0676 | 306 | 27 | 1 | 9.15 |
| rps19 | 0.0672 | 0.1656 | 0.1684 | 0.0378 | 0.0605 | 0.0453 | 0.5625 | 0.3653 | 0.2690 | 279 | 25 | 1 | 9.32 |
| ycf1 | 0.1686 | 0.2293 | 0.2349 | 0.1119 | 0.1453 | 0.1479 | 0.6637 | 0.6337 | 0.6296 | 5863 | 1005 | 58 | **18.13** |
| ycf2 | 0.0271 | 0.0503 | 0.0503 | 0.0177 | 0.0343 | 0.0289 | 0.6531 | 0.6819 | 0.5746 | 6909 | 288 | 5 | 4.24 |
| ycf3 | 0.0699 | 0.1783 | 0.1783 | 0.0102 | 0.0128 | 0.0128 | 0.1459 | 0.0718 | 0.0718 | 513 | 30 | 0 | 5.85 |
| ycf4 | 0.1766 | 0.2518 | 0.1871 | 0.0267 | 0.0416 | 0.0367 | 0.1512 | 0.1652 | 0.1962 | 555 | 51 | 3 | 9.73 |
