# Supplementary material for: Comparative Genome Analysis and Phylogenetic Relationship of Order Liliales Insight from the Complete Plastid Genome Sequences of Two Lilies (Lilium longiflorum and Alstroemeria aurea)
Source: PLoS One. 2013 Jun 18;8(6):e68180. doi: 10.1371/journal.pone.0068180 (PMC3688979; doi:10.1371/journal.pone.0068180)
Supplement: Table S3 — (DOCX) [file pone.0068180.s004.docx]

| Table S3. Comparison of the sequence variation between two *Lilium* species. | | | | | |
| --- | --- | --- | --- | --- | --- |
|  |  |  |  |  |  |
| gene name | total length | true length | no. of variable sites | % of variable site* | indel (type)** |
| atpA | 1524 | 1524 | 6 | 0.39 |  |
| atpB | 1503 | 1503 | 2 | 0.13 | TTGGCG (in/repeat) |
| atpE | 408 | 408 | 3 | 0.74 |  |
| **atpF** | **1340** | **159 (exon1)** | **2** | **1.26** |  |
| atpH | 246 | 246 | 1 | 0.41 |  |
| atpI | 744 | 744 | 3 | 0.40 |  |
| **ccsA** | **687** | **687** | **7** | **1.02** |  |
| clpP | 2004 | 618 (exon) | 4 | 0.65 |  |
| **infA** | **231** | **231** | **5** | **2.16** | **CTTTTA (del/repeat)** |
| **matK** | **1539** | **1539** | **24** | **1.56** |  |
| **ndhA** | **2092** | **1092 (exon)** | **11** | **1.01** |  |
| ndhB | 2215 | 1566 (exon) | 3 | 0.19 |  |
| ndhC | 363 | 363 | 2 | 0.55 |  |
| ndhD | 1506 | 1506 | 12 | 0.80 |  |
| ndhE | 306 | 306 | 1 | 0.33 |  |
| **ndhF** | **2223** | **2223** | **32** | **1.44** |  |
| ndhG | 543 | 543 | 3 | 0.55 | 8T (del/poly-T) |
| ndhH | 1182 | 1182 | 9 | 0.76 |  |
| ndhI | 540 | 540 | 5 | 0.93 |  |
| ndhJ | 477 | 477 | 3 | 0.63 |  |
| ndhK | 762 | 762 | 6 | 0.79 |  |
| petA | 963 | 963 | 5 | 0.52 |  |
| petB | 1461 | 645 (exon2) | 3 | 0.47 |  |
| petD | 1233 | 477 (exon2) | 2 | 0.42 |  |
| **petG** | **114** | **114** | **4** | **3.51** |  |
| **petL** | **96** | **96** | **1** | **1.04** |  |
| petN | 90 | 90 | 0 | 0.00 |  |
| psaA | 2253 | 2253 | 11 | 0.49 |  |
| psaB | 2205 | 2205 | 8 | 0.36 |  |
| psaC | 246 | 246 | 1 | 0.41 |  |
| psaI | 105 | 105 | 0 | 0.00 |  |
| psaJ | 135 | 135 | 0 | 0.00 |  |
| psbA | 1062 | 1062 | 4 | 0.38 |  |
| psbB | 1527 | 1527 | 6 | 0.39 |  |
| psbC | 1416 | 1416 | 7 | 0.49 |  |
| psbD | 1062 | 1062 | 4 | 0.38 |  |
| psbE | 252 | 252 | 1 | 0.40 |  |
| psbF | 120 | 120 | 0 | 0.00 |  |
| psbH | 222 | 222 | 2 | 0.90 |  |
| psbI | 111 | 111 | 1 | 0.90 |  |
| psbJ | 123 | 123 | 0 | 0.00 |  |
| psbK | 192 | 192 | 1 | 0.52 |  |
| psbL | 117 | 117 | 0 | 0.00 |  |
| psbM | 105 | 105 | 1 | 0.95 |  |
| psbN | 132 | 132 | 1 | 0.76 |  |
| psbT | 102 | 102 | 0 | 0.00 |  |
| psbZ | 189 | 189 | 0 | 0.00 |  |
| rbcL | 1464 | 1464 | 9 | 0.61 |  |
| rpl2 | 1503 | 825 | 1 | 0.12 |  |
| **rpl14** | **369** | **369** | **5** | **1.36** |  |
| rpl16 | 1416 | 402 (exon) | 3 | 0.75 |  |
| **rpl20** | **354** | **354** | **4** | **1.13** | **CTTTAG (in)** |
| rpl22 | 393 | 393 | 3 | 0.76 |  |
| rpl23 | 282 | 282 | 0 | 0.00 |  |
| rpl32 | 174 | 174 | 0 | 0.00 |  |
| rpl33 | 204 | 204 | 1 | 0.49 |  |
| **rpl36** | **114** | **114** | **3** | **2.63** |  |
| **rpoA** | **1008** | **1008** | **16** | **1.59** |  |
| rpoB | 3207 | 3207 | 10 | 0.31 |  |
| **rpoC1** | **2820** | **2079 (exon)** | **21** | **1.01** |  |
| **rpoC2** | **4125** | **4125** | **46** | **1.12** |  |
| rps11 | 417 | 417 | 2 | 0.48 |  |
| rps12 | 915 | 366 (exon 1) | 1 | 0.27 |  |
| rps12 | 1497 | 825 (exon) | 2 | 0.24 |  |
| **rps14** | **303** | **303** | **5** | **1.65** |  |
| **rps15** | **273** | **273** | **10** | **3.66** |  |
| rps16 | 1138 | 210 | 2 | 0.95 |  |
| rps18 | 306 | 306 | 2 | 0.65 |  |
| **rps19** | **279** | **279** | **4** | **1.43** |  |
| rps2 | 711 | 711 | 6 | 0.84 |  |
| **rps3** | **657** | **657** | **12** | **1.83** |  |
| rps4 | 606 | 606 | 1 | 0.17 |  |
| rps7 | 468 | 468 | 0 | 0.00 |  |
| **rps8** | **399** | **399** | **4** | **1.00** |  |
| ycf1 | 5577 | 1208 | 1 | 0.08 |  |
| ycf2 | 6654 | 6654 | 13 | 0.20 | ATATCTATTTTGATGATAGTGACA (del) |
| ycf3 | 1950 | 513 (exon) | 3 | 0.58 |  |
| ycf4 | 555 | 555 | 4 | 0.72 |  |

* % of variable sites in a truly aligned sequence

** in: insertion compare to *Lilium superbum* / del: deletion compare to *Lilium superbum*
