# Supplementary material for: Comparative Genome Analysis and Phylogenetic Relationship of Order Liliales Insight from the Complete Plastid Genome Sequences of Two Lilies (Lilium longiflorum and Alstroemeria aurea)
Source: PLoS One. 2013 Jun 18;8(6):e68180. doi: 10.1371/journal.pone.0068180 (PMC3688979; doi:10.1371/journal.pone.0068180)
Supplement: Data File S1 — (DOCX) [file pone.0068180.s005.docx]

**Supplimentary data. Aligned matrix of combined 79 genes for the present study**

>Acorus_americanus

------------------------------------------------------------

------------------------------------------------------------

------------------------------------------------------------

------------------------------------------------------------

------------------------------------------------------------

------------------------------------------------------------

------------------------------------------------------------

------------------------------------------------------------

------------------------------------------------------------

------------------------------------------------------------

------------------------------------------------------------

------------------------------------------------------------

------------------------------------------------------------

------------------------------------------------------------

------------------------------------------------------------

------------------------------------------------------------

------------------------------------------------------------

------------------------------------------------------------

------------------------------------------------------------

------------------------------------------------------------

------------------------------------------------------------

------------------------------------------------------------

------------------------------------------------------------

------------------------------------------------------------

------------------------------------------------------------

------------------------------------------------------------

------------------------------------------------------------

------------------------------------------------------------

------------------------------------------------------------

------------------------------------------------------------

------------------------------------------------------------

------------------------------------------------------------

------------------------------------------------------------

-ATGGCAACCCTTCGAGCCGATGAAATTAGTAATATTATCCGTGAACGTATTGAACAATA

TACTAGAGAAGTGAAGGTTGTGAATACCGGTACAGTCCTTCAAGTAGGCGACGGCATTGC

TCGTATTCATGGTCTTGATGAAGTAATGGCAGGTGAATTAGTCGAATTTGAAGAGGGTAC

GATAGGTATTGCTCTTAATTTGGAATCCAATAATGTTGGCGTTGTACTAATGGGTGACGG

TTTGACAATACAAGAGGGAAGTTCTGTAAAAGCAACAGGAAGAATTGCTCAGATACCGGT

GAGTGAGGCTTATTTGGGTCGTGTTGTAAATGCTCTGGCTAAACCTATTGATGGGAGAGG

TGAAATTGCAGCTTCTGAATTCCGGTTAATCGAATCTCCTGCCCCAGGTATTATTTCGAG

ACGTTCCGTATATGAGCCCCTTCAAACAGGTCTTATCGCTATTGATTCGATGATCCCTGT

AGGGCGCGGTCAGCGAGAATTAATTATTGGGGACAGACAGACCGGTAAAACAGCGGTAGC

CACAGATACTATTCTCAACCAAAAAGGACAAAATGTAATATGCGTTTATGTAGCTATTGG

CCAAAAAGCATCTTCCGTCGCGCAGGTAGTGACTAATTTCCAGGAGCGAGGGGCGATGGA

ATACACTATTGTGGTAGCCGAAACGGCGGATTCCCCCGCTACATTACAATACCTGGCTCC

TTATACAGGAGCGGCTTTGGCTGAATATTTTATGTACCGTGAACGACATACTTCAATAAT

TTATGATGATCTCTCCAAACAAGCGCAAGCTTATCGCCAAATGTCTCTTCTATTAAGAAG

ACCGCCCGGACGTGAAGCTTACCCAGGAGATGTTTTTTATTTGCATTCCCGCCTTTTGGA

AAGAGCCGCTAAATCAAGTTCTCGTTTAGGTGAAGGAAGTATGACTGCTTTACCAATAGT

TGAAACTCAATCTGGGGATGTTTCGGCTTATATTCCTACTAATGTAATTTCCATTACAGA

TGGGCAAATATTCTTATCCGCCGATTTATTCAATGCCGGAATACGACCTGCTATTAATGT

GGGTATTTCTGTCTCCAGAGTAGGATCCGCAGCTCAAATTAAAGCCATGAAACAAGTAGC

TGGCAAATCAAAATTGGAACTAGCTCAATTCGCGGAGTTAGAAGCTTTTGCACAATTCGC

TTCTGATCTCGATAAAGCTACTCAGAATCAATTGGCAAGAGGTAAACGATTGCGCGAGTT

ACTTAAACAATCCCAATCAGAACCTCTCGCGGTGGACGAACAAGTGGTTACTATTTATAC

CGGAACGAATGGATATCTTGATACGTTAGAAATTGGGCAGGTAAAGGAATTTCTCGTTAA

GTTACGTACCTACTTAAAAAAGAATAAACCTCAGTTTCAAGAAATCATATCTTCTACCAA

GACCTTTACCGAGGAAGCAGAAGCTCTTTTGAAGGAAGCTATTCAGGAGCAGCTCGAACT

CTTTCTACTTCAGGAACAAACA---------------------TAAATGAGAATAACTCC

TACTAC------TTCTGGTT------CTGCGGTTCCCTCACTTGACGAGAAAAACTTAGG

GCGTATCGTCCAAATCATTGGCCCAGTACTGGATGTCGCCTTTCCCCCGGGAAAGATGCC

TAATATTTACAACGCTTTGGTGGTTAAGGGTCGAGATACTGCCGGTCAGCCAATTAATGT

GACTTGTGAGGTACAGCAATTATTAGGAAATAATCGGGTTCGAGCTGTGGCTATGAGTGC

TACAGATGGTTTGACGAGAGGAATGGAAGTGATTGACACGGGAGCTGCTCTAAGTGTTCC

AGTCGGTGGAGCTACTCTCGGAAGAATTTTCAACGTTCTTGGGGAGCCCGTTGATAATTT

AGGCCCTGTAGATACTCGCACAACATCTCCTATTCATAGATCTGCGCCTGCATTTATACA

GTTAGATACGAAATTATCAATCTTTGAAACAGGCATTAAAGTGGTAGATCTTTTAGCTCC

TTATCGCCGGGGAGGAAAAATCGGACTATTTGGGGGAGCTGGAGTGGGTAAAACAGTACT

CATCATGGAATTGATCAACAACATTGCCAAAGCGCATGGAGGTGTATCCGTATTTGGCGG

AGTAGGCGAGCGTACTCGTGAAGGAAATGATCTTTACATGGAAATGAAAGAATCCGGAGT

AATTAATGAAGAAAAGATTGAAGAATCTAAAGTAGCTCTAGTGTATGGTCAGATGAATGA

ACCGCCGGGCGCTCGTATGAGAGTTGGTTTGACTGCCCTAACCATGGCGGAATATTTCCG

GGATGTTAATGAACAGGATGTACTTCTATTTATCGACAATATCTTCCGGTTCGTACAAGC

GGGATCAGAAGTATCTGCCTTATTAGGGCGGATGCCTTCCGCCGTGGGTTATCAACCTAC

CCTTAGTACAGAAATGGGTTCT---TTGCAAGAAAGAATTACTTCTACAAAAGAGGGATC

TATAACGTCTATTCAAGCGGTTTATGTACCTGCGGATGATTTGACTGACCCTGCTCCTGC

CACCACATTTGCACATTTGGATGCTACTACTGTACTATCAAGAGGATTAGCTGCCAAAGG

TATTTATCCGGCAGTAGATCCTTTAGATTCAACGTCAACTATGCTCCAACCTAGGATCGT

TGGCGAGGAACATTATGAAACTGCGCAAAGAGTTAAGCAAACTTTACAACGTTACAAAGA

ACTTCAGGACATTATAGCTATCCTTGGGTTGGACGAATTATCCGAAGAGGATCGTTTAAC

CGTAGCAAGAGCACGAAAAATTGAGCGTTTCTTATCACAACCCTTCTTCGTAGCAGAAGT

ATTTACTGGTTCTCCAGGTAAATATGTTGGTCTCGCGGAAACGATTAGGGGGTTTCAACT

GATCCTTTCAGGAGAATTAGATGGTCTTCCCGAGCAGGCCTTTTATTTGGTGGGTAACAT

CGATGAAGCTACCGCGAAGGCTATGAATTTAGAAGTGGAGAGCAAATTGAAGAAATGAAT

GAACTTAAAGCTTTGTTTACTGACTCCTAATCGAATTATTTGGGATTCAGAAGTGCAAGA

AATCATTCTATCTACTAATAGTGGACAAATTGGCATATTACCAAATCACGCCTCTATTGC

CACAGCTGTAGATATCGGTCTTTTGAGAATACGCCGC---GACGACCGATGGTTAGCGGT

GGCTCTGATGGGGGGGTTTGCTAGAATAGGAAATAATGAGATCACCATTTTAGGAAATGA

TGCCGAGATGGGTACTGACATTGATCCGCAAGAAGCTCAACGAGCTCTTGAAATAGCTGA

AGCTAACTTGAGTAAAGCTGAAGGTAAGAGACAAGCAATTGAGGCAAATTTAGCGCTAAG

ACGAGCTAGGACACGGGCGGAGGCTATTAATG---------TTATT------------TC

CCAATAGATGAAAAATATAACCGATTCTTTCGTTTCCTTG---GGCCACTGGCCATTCGC

CGGGAGTTTCGGGTTTAATACCGATATTTTAGCAACAAATCTAATAAATCTAAGTGTAGT

GCTTGGTGTATTGATCTTTTTTGGAAAGGGAGTGTGTGCGAGTTGTGTATTTCAAGAATA

-GGCCGGATTCGGCCGGCTGTACTTTA---------------------------------

------------------------------------------------------------

----------------------------TTTCTTA-------------------------

------------------------------------------------------------

------------------------------------------------------------

------------------------------------------------------------

------------------------------------------------------------

------------------------------------------------------------

------------------------------------------------------------

------------------------------------------------------------

-----------AAAAAAAAAAAAAATTCGGAAAGA-------AAGGTGCACCATCTCG-A

CGAATTACTTCTGAATA----------AATTCATAAATCAATC-----ATATGTAAGAAC

CATAGCATTTCGTGATTCATTGGT-----AAATTAACTTTGA-----TTCTCTATTAA--

---CCAAGAATA-TGGGACCA-----TTAACATGGTTAAAGCT-AAACCGTTTGAAGTCT

AGGC--ATAACAC-----GGTACTCTTTCTACCGCTACGTTAGTA------CCAAGATGG

TT-------TCAAAATG----------------AATAGTGGAAAATTTATCCAATATAGA

ACACTCATATTGATAAAATCATT-TGAACTGGACCATTTACTAA----------------

--AA--AAGGGGGAGCGCCTCACCCTT------TTATCCAATGCTGAATCGACGACCTAT

GTAT---------AAAAT--------AAGAATAA--TTTTTTGGATT--------TGAAG

------------------------------------------------------------

------------------------------------------------------------

------------------------------------------------------------

----------------------------------------------------------AA

AAAAAAAACACACACAACTTTGCTGATAATTACAGATTTATTT-GTCTGGTCGGAAGAGC

TC-----------TCC--GAATAT-----TCTGGTCTTGTAT-----CAATCAGTTT---

-----------------------------------------TGATATTTTGGAATATGAG

TAGAAAAG-------AGAGGGTAGGCTCATTACAT-----TAACAGATATGGGAAAA-CC

TATA---------------------A--GTAATTGAGCGTGAGAGCCAAATGAATCGAAA

GATTCGTGTTTGGTTCGGGAAGAGATCA-TGGGATTCGTGAAAT-----AAGTAGTCAGA

TAATCTACTTTCATTAAGTGATTTATTAGATAATCGAAGACAGAGGATCTTGAGTACTAT

TCGAAATTCAGAAGAACTGCGTAGAGGAGCCCTTGAGCAGCTCGAAAAAGCCCGGGCTCG

CTTACGGAAAGTAGAAATGGAAGCAGATGAGTATCGAGTGAACGGATACTCCGAGATAGA

ACGAGAAAAGATGAATTTGATTAATGCCACTTATGAAAATTTGGAACGATTAGAAAATTA

CAAAAATGAAACCCTTCATTTTGAACAACAAAGAGCAATTAATCAGGTCCGGCAACGAGT

TTTCCAACAAGCGTTACAAGGAGCTCTAGGAACTCTGAATAATTGTT------TGAATAG

TGAATTACATTTCCGTACCATCAGTGCTAATATTGGCATGCTTGGGGCCATGAAA-----

GAAATAACTGATTAGATGAATCCACTGATTTCTGCCGCTTCCGTTATTGCTGCTGGATTA

GCCGTAGGACTTGCTTCTATTGGACCTGGGGTTGGTCAAGGTACTGCTGCAGGCCAAGCC

GTAGAAGGTATTGCGAGACAACCAGAAGCAGAAGGTAAAATACGAGGTACTTTATTGCTT

AGTCTGGCTTTTATGGAAGCTTTAACAATTTACGGACTGGTCGTAGCATTAGCACTTTTA

TTTGCGAATCCTTTTGTCTAAATGAATGTTATACTATGTTCCAGCAACATGCTCAAAGGG

TTATATGATATATCTGGTGTAGAAGTGGGCCAACATTTATATTGGCAAATAGGAGGGTTC

CAAGTCCATGCCCAAGTACTAATCACTTCTTGGGTCGTAATTGCTATCTTATTAGGTTCA

GTCACTGTCGCTGTTCGGAATCCACAAACCATTCCGACCAACGGTCAGAATTTCTTTGAG

TATGTTCTTGAATTCATTCGAGACTTGAGCAAAACACAGATTGG---AGAAGAATATGGT

CCTTGGGTTCCCTTTATTGGAACAATGTTCCTATTTATTTTTGTTTCGAATTGGTCAGGC

GCTCTTTTACCTTGGAAACTCATAGAGTTACCTCACGGGGAGTTAGCCGCACCCACGAAT

GATATAAATACTACTGTTGCTTTAGCTTTACCCACATCTGTAGCATATTTCTATGCGGGT

CTTACCAAGAAAGGATTGGGTTATTTCGGGAAATACATTCAACCAACTCCGATCCTTTTA

CCAATTAATATCCTAGAAGATTTCACAAAACCTTTATCACTGAGTTTTCGACTTTTCGGA

AATATCTTGGCTGATGAATTAGTAGTTGTTGTTCTTGTTTCTTTAGTACCTTTGGTAGTT

CCTATACCTGTCATGTTTCTTGGATTATTCACAAGCGGCATTCAAGCTCTTATTTTTGCA

ACTTTAGCCGCGGCCTATATAGGCGAATCCATGGAAGGTCATCATTGAATGATATTTTCA

ACTTTAGAACATATATTAACTCACATATCTTTTTCGATTATTTCAGTTGTGATTACAATT

CAGTTGATGAACTTATTAGTCCACGAACTGGTACAACTAGGTGATGCGTCAGAAAAAGGC

ATGATAGCTACCTTTTTCTCTATCACGGGATTATTGGTTACTCGTTGGATCTATTCGGGT

CATTTCCCACTAAGTGATTTATATGAATCATTAATCTTCCTTTCATGGAGTTTCTCCATT

ATTCATATGGTTCCATATTTTAGGAACCACAGAAATC------ATT---TCAGTGCAATA

ACTGCGCCAAGTGCCATTTTTACCCAAGGCTTTGCTACTTCGGGTCTTTTAACGGAAATG

CATCAATCCATAATATTAGTACCCGCTCTACAATCCCAGTGGTTAATGATGCATGTAAGT

ATGATGTTATTGAGCTATGCAGCTCTTTTATGCGGGTCATTATTATCAGTTGCTCTTCTA

GTTATTACGTTTCGAAAAAACAGCGATATTTTTGA------TAAAAGA---AATAATTTC

TTAATT------AGGTCATTTTTC---------TTTGGTGAGATTGAA------TACTTG

AATGAAAA---ACGC---------AGTGTTTT---ACAAAACACTTCGTTT---GATTCA

TTTACAAATTATCACAAATATCAATTGACTCAACGATTAGATTTTTGGAGTTATCGGGTC

ATTAGTCTAGGGTTTATCTTTTTAACCATAGGTATTCTATCGGGGGCAGTGTGGGCTAAT

GAGGCATGGGGATCTTATTGGAATTGGGACCCAAAGGAAATTTGGGCATTTATTACCTGG

GCTATATTCGCAATTTATTTACATACTAGAACGAATCATAGTTTGCAAGGT------GCG

AATTCGGCAATTGTGGCTTCTATCGGATTTCTTATAATTTGGATATGTTATTTTGGAGTC

AATCTATTAGGAATAGGTTTACATAGTTATGGTTCATTC------ACATTA-ATATCTAA

T------------------------TGA--------------------------------

------------------------------------------------------------

------------------------------------------------------------

------------------------------------------------------------

----------GTGCCAAAAAAGAAAGGATTTACTCCCCTCCCATATCTTGCATCTATAGT

ATTTTTGCCCTGGTGGGTCTCTCTCTCATTTAATAAGAGTCTGGAACCTTGGGTTACCAA

TTGGTGGAATACCAGACAATCTGAAACCTTTTTGAATGATATTCAAGAGAGGAACGTTCT

AGAAAGATTCATCGAATTAGAAGAACTATTCCTGTTGGACGAAATGCTAAAGGAGAACCC

GGAGACACGTATGAAAAATCTTCGTATAGGAATCCACAACGAAACGATCCAAT-TGGTCA

AAACAGACAATGAGTATCATCTCCATACCATTTTGCATTTCTCGACAAATATAATCTGTT

TCGCTATTCTAAGTGTTTATTCTATTCTGGGTAATGAAGAACTTGTCATTCTTAATTCTT

GGGTTCAGGAATTTCTTTATAACTTAAGCGACACAATAAAAGCTTTTTCGATTCTTTTAG

TTACTGATTTATGGATTGGATTCCATTCACCCCACGGTTGGGAACTAATGATTGGTTCGG

TCTACAATGATTTTGGATTAGCTCATAACGAACAAATTATATCTGGGCTTGTTTCCACTT

TTCCAGTTATTCTAGATACGATTGTGAAATATTGGATCTTCCATTATTTAAATCGTGTCT

CTCCTTCACTTGTAGTGATTTATCATTCAATGAATGA---CTGAATGCCCATTGGTGTTC

CAAAAGTACCTTTTCGGAGTCCTGGAGAG---GAAGATGCAGCTTGGGTTGACATATAGT

GCGACTTGTCAGACATATTTGGTCCTATGGAATTTC-CCCGTTCTCTCCTCTGCTCTGAT

CGAGATATCCTCTGTTTCGCCCAA----------------GAAAG-----------ATTA

ATTGAACTATCAAT----------AACTCGGAGCGTGAA--ATGTAATTAGATCGACTTT

TTTGGAGGACCAATATT-----CTCAATTATAATAATTTATGGTTT-CTCAGGCCAAT--

-----AAAATAAAG-----TATTCAGGCTTCGTTCATAAAATACCAAATTTGTAATAA--

------------------------------------------------------------

------------------------------------------------------------

------------------------------------------------------------

-----------------------------------------------------ATATTTA

TATGATCCTAT-----------TTATAAATATCA--------------------------

--------------TAGTAGTGATCTC-----------AAACTGCC-----AACCAATGA

AGTAATATGAT--GA---ATACATCGAATATT---TTGTCGGAAGGTATGAAATGAATTG

----------------AAAAAAAAAAGCAATA----------------------------

-ACAAAAGGAAGTGGTACTGGTGGGGTTATTTGTCCTATATGTGCAAATCAAATTCGAAC

AGATCTTTTCCCGGAGTAG----AACATGAACCTAAAAGAAAAGAATTGAAAGAAAAGAA

TTGAAG-AGGCCTTTTCGGGAACAAGAAAATTACATCGTGATTTGAATCTCTATGAAACA

ATACCATCAACGAGAG----------GTTAATTCAAAAAGTTCAATGACTTTTTATATAT

TTT----------------------------TATAGAAAAGGGAGCCAAAGCTC------

---GGGTTTCATATTAAAAAAAAAAAAAAACAA---------------------------

--------TAAAAAAAAATTAGGGCTAGAATCGATACATTGATCC----TTTTTCTC---

---AATTTTT----TGAACCGTATGCATACAAAGGTGCATGTACGGTTCTTAGGGAATAC

AATTTTGTCTTAATCAACCGACTTCATCGAGAAAGATTACTTTTTTTAGGCCAAGAGCTT

GATAGCGAAATCTCGAATCAACTTGTTGGTCTCATGGTATATCTCAGTATAGAGGATAAG

ACCAGGGATTTTTTTTTGTTTATA--AATTCTCCCGGCGGATGGGTAATTCCGGGAATAG

GTCTTTATGACACTATGCAATTTGTGCCACCCGATGTACATACAATATGCATGGGATTAG

CTGCTTCAATGGGATCTTTCATTCTTGTCGGAGG-------AGAAATTACCA---AACGT

CTAGCATTCC-CTCACGCTTGGCGCCAATGAGTTTT---TTTATTT------------GA

GAAAAAGAAGACTATGCC-TTCGCCATATCCAATAGGAA---------------------

------------------------------------------------------------

-------------------------TTATAAGTAATAATAGCATGGCACGTCGAGTTCGA

TATGAACAAACCATTATTGTT---------TTTTCAAAACATGAAATTCTGTATCGAGAT

AGTAGTATGAAAC-AAAGGTTATTTCCGAC----TTTATCTTAT-------GTAT----G

AGAGATCCATT--CTAGCGTCACAAACCTTTTTGCTTCCACACCAGA----AGTCTCTTT

CCGATATTTCTGTTATGAAAATGAAAGAAAGAGTACGAAAAAAAAAAAAAAAAAAAGAGC

ATTTTTGACTTATTTTTGTTTGA---TCAGATCAGAAAGAAACTTTGGGATTGCTGAATC

ACAGATGAGATAAAT-------------ATATAAAGCAACAGAACCATCATAGTAT----

-------------------TTTTGGAGCCTACGAAACAAAAGGAAGGTTGGTAAATGGAT

CATTCAACGAT----CCGGGTCAGGTGGATTCGATTTATCTC-----------TTTTTCG

A-----TGGAAGGAAGGGAAAAGTAAATTCCATTGCAGAGCCGTATGC-----ACAAAAG

GTGCCTGTACGGTTGTTCACTTCTATCTTTTTCTTCTTTATCCCTTCT------------

-------------TTTAGCCCGATCATGTG------AGATAGAAAAACCATTCAT----G

ATGTTATATG-----------ATCAGGGTTATGATCCACCAACCTGCCAGTTCTTTTTAT

GAGGCACAAGCAGGGGAATTTGTCCTGGAAGCGGAAGAACTATTGAAACTTCGC----GA

AACCCTCACAAGAGTTTACGTA--CAAAGAACTGGTAAACCATTATGGGTTGTATCCGAA

GATATGGAAAGGGATGTTTTTCTGTCAGCAACAGAAGCCCAAGCTCATGGTATTGTTGAT

CTTGTAGGGGACGAAA------------AT------ATGG-------GAGATTTAGTGTA

A-----------------ATGAAAGAACA-------------------------------

-----AAAATTGATTCAT---GAAGGTTTAATTACTGAATCACTTCCTAATGGTATGTTT

TGGGTG------CGTTTAGATAATGAAGATCTGGTTCTAGGTTATATTTCTGGAAGGATC

CGACGCAGTTCTATACGGATACTACCGGGAGATAGAGTAAAAATCGAAGTAAGTCGTTAT

GATTCAACCAGAGGACGCATAA--TTTATAGACTTC------------------------

------------------------------------------------------------

------GCAATAAAGATTCGAACGAG------------TAAATGGAAGAAT---------

---TCAAAGTCTATTTCG-----AAAAAGATGG----GTCTCGTCAACAATAC------T

TCCTATATCCACTTCTCTTTCAGGAGTACATCTACGCACTTGCTCATAATCATGTTTTAA

ATGG------ATTGATTTTTTACGAATCCTCGGAAAATTTCGTTT------ATGATAATA

AATTTAGTTTAATAATTGTAAAACGTTTAATTACTCAAATGTATCAACAGAATTCTTTGA

GTAATTTGGTTAATGATTCTAACC------GAAATCGATTAGTTCGGCAAAACAAGAA--

-------------TTTTTATTATCAAACGATATTAGAGGGTTTTTCAGTCATTATGGAAA

TTCCATTCTCCATAATATTTTTATCTTCCGTTGAAGAAAAAAAAGCAAAAATACCAAAAA

TTCA------GAATTTACGATCTATTCATGCAACATTTCCCTTTTTAGAAGACAAATTAT

CACATTTAAATCATGTATCAGATATATTAATACCCTATCCCATCCATCTCGAAATCCTGG

TTCAAGTTCT------GCAAGGCTGGATACAAGATGTTCCGTCTTTACATTTATTGCGGT

TCTTTCTCCATGAGTTTCATAATTGGAATAGTCTC---ATTAC------TCCAAAGA---

------AATCCATTTCTTTTCTT---TTTTCAAAGGGGAATCAAAGACTCTTCTTGT---

TCTTATATAATTCTTATGTATTTGAGTGTGAATCCGCATTAGTCTTTCTCCGTAAAC---

AATCCTTTTATTTACGATCAACATCTTTTGGAACCTTTGTTGAGCGAACACA-TTTCTAT

GGAAAAATAGAGC---------------------ATATTG---------TAGTAGTGCGT

AGG---AAGAATTT------TCA--AAAGGCCTTA------TCTTTGTTCAAAGATCCTT

TCATCCATTATATCCGATATAAAGGAAAATCAATTCTGGCTTCAAAGGGGACTCATTTTC

TGATGAAGAAATGGAAATATCACCTTCTAAATTTCTGGCAATGTCATTTTGACTTTTGGT

CTCAACCGCATAGGATTCATATAAACCAATTATCAAATCATTCTTTCTATTTTATGGGCT

ATCTGTCAAGTGTACGAA-----TCAATTTTTCGACGGTAAGGAGTCAAATGCTAGAGAG

TTCATTTCTAATGGATACTCCTACTAAA------AAATTCGATACTATAGTCCCAATTAT

TCCTCTAATCGGATCATTGGCTAAAGCTAAGTTTTGTAACGTATCGGGGCATCCTGTTAG

TAAGCCGGTCTGGGCTGATTTGTCAGATGCTGATATTATTGATCGATTTGGACGAATATG

TAGAAATCTTTCTCATTATCACAGTGGATCCTCAAAAAAACAGAGTTTGTATCGAGTAAA

GTATATACTTCGCCTTTCGTGTGCGAGAACTTTGGCCCGTAAACATAAAAGTACAGTGCG

CGCTTTTTTCAAACGATTAGGTTCAGAATTCTTAGAAGAATTCTTTACGGATAAAGAACA

AGTT--------CTTTCTTTGATC-----TTCCC---------TTCCCTTCGTTCACCTT

CACACAGGGTCCATAAAGGAGAACGTATTTGGTATTTGGATATTATCCGTATCAATGACC

TGGTGAATA------------------------------------------ATTCATGA-

--------------------------------------------------ATGATAATTG

CTACGAC------AGAAATACAAGCTATCAATTCTTTTTCTAGATCGGAATCCTTATCCT

TAAAAGAGGTCTACGGACTCATATGGCTGCTTGTACCTATTTTTACTCTTATATTGGTAA

TTATAATAGGGGTACTAGTGATTGTTTGGTTGGAAAGAGAAATATCTGCGGGGATACAAC

AACGTATTGGGCCTGAATATGCCGGCCCATTGGGAATTCTTCAAGCTCTAGCGGATGGAA

CCAAACTGCTTTTCAAAGAAGATCTTCTCCCATCTAGAGGAGATATTAGTTTATTCAGCC

TCGGGCCGTCTATAGCGGTCATATCAACTCTACTAAGTTATTTAGTAATTCCTTTTGGCT

ATCACCTTGTTTTAGCTGATCTCAGTATCGGCGTTTTTTTATGGATTGCCATTTCAAGTA

TTGCTCCTATTGGACTTCTTATGTCAGGATATGGCTCGAATAATAAATATTCCTTTTCAG

GTGGTCTACGAGCTGCCGCTCAATCTATTAGTTATGAAATACCATTAACTCTATGTGTAT

TATCAATATCTCTACGTGTGATTCGTTGAAACATGAACTTTTATCCCCCCTTTTATCCCC

TTTCCTTTTTAAGGGGG-------------T-----------------------------

---------TGAATGTATTGAATACATCTCTTCAGCTTTTATTCATCATTCG--------

--------------------ATTCGGGTTGATAAACTGAACTAG------ATAGTTATAT

GAGTGAAATAAAAC-----AGCTTAGAAATTCGCAGTAAGAAGATTGAATCTCATTCCCT

AT-GTACGAGAGTAAAGTCGAAGTAAACATAAG-----CAGTGCAAACGATTTACCCCAA

GATTTAGATT---------GGCATCATAT-----CTTGAAGCGGGT----GCAAAAGATC

CACTATGTATGGACGTTCTACTATTGTCTTGT----------------------ATGTAT

TACCATACCGGGGATCAATCAAAAATGAGTGGACATTTAGGAACA-CCAAGGTACA----

-------CAAAGGATTAGTAATGGAGATAATATAACGTGTCAAAAGAGGTATT-TCCTCA

TTCA----------TAAAACGAATCAAAAT-------GGGCTTTAAGTTCGTAGAAATGA

TCGAGCAGTACTTCCCTATGATTCCGATCTAGAGTAT-ACTCCTATTCACTCATTAAAGA

AA--TAACTATCAGG-AACGAATTAATCCCTTATTTTCTTTT----------CGAGTACC

TTCCTTTGAGAAAGAAGAACTGGAATAAAAGAAATAGAATACTATTCTAGG---------

--------AAAAATGAATAATAAAGGATCTTTCTTT------------------------

-----ATTCTTTCTTTCCTC------T-ACCCATA----TTCAGACATACGGAATTCTTA

T---C----------ATTATTCATGAACTAG----------TATAGTATACC-TAATTCT

TTTCATAATAGAGGGGTATGGG-------TCGAAATATCTATTAATACAGCGAGTATTTT

AT------TGAAGGATTAAGTTATTACTGA-ACAAAGAG--AAATCAAAATCATAA----

---AGGATAAGATCAATT-CAGAAGCG----CTTTTATT-----TATTATT-TTCTAGCA

GACAGAATTCGATTGGTCTAATTCA-----GGACTCCCCGCTGCATCTTTATTTATTCTA

TATAACCTAG--------AATGAGATGCTGAGTGAATTAATTTAATTTAATACCAAACAA

ACCAG--------TCCTTAGATTTATTTGT----GACCATAAAGGAGCCGTATGAGGTGA

AAATCTCATGTACGGTTCTGGAATAGCAATGAGAACAGTGATGTTTTCATCGACTATGAT

TATCTAATAGTTCAAGTACAGTTGATATAGTTGAGGCACAGTCAAAATATGGTTTTTGGG

GATGGAATCTGTGGCGTCAACCTATAGG-ATTTTTAGTTTTCCTAGTTT--CTTCTCTAG

CCGAATGTGAAAGATTACCCTTTGATTTACCAGAAGCAGAGGAAGAATTAGTAGCAGGTT

ATCAAACTGAATATTCAGGCATCAAATTTGGTTTATTTTACGTTGCTTCTTACCTAAATC

TACTAGTTTCTTCATTATTCGTAACAGTTCTTTACTTAGGCGGGTGGAATCTCTCTATTC

CATACATATTTATTCCTGAACTTTTCGGA---------AAAAACAAAACAGGTGGAATCT

TTGGAATGACAATTGGTATCCTAATTACATTAGCTAAAGCTTATTTGTTCCTGTTCATTT

CTATCGCAACAAGATGGACTTTACCTAGGTTAAGAATAGACCAACTATTAAATCTTGGAT

GGAAATTTCTCTTACCTATTTCCCTCGGTAATCTATTATTGACAACCTCTTCCCAACTTG

TTTCACTATAA-------ATGATCTGGCATGTACAGAATGAAAACTTCATTCTCGATTCT

ACGAGAATTTTTATGAAAGCGTTTCATTTGCTTCTCTTCGATGGAAGTTTCATTTTCCCA

GAATGTATCCTAATTTTTGGCCTAATTCTTCTTCTGATGATCGATTCAACCTCTGATCAA

AAAGATAGACCTTGGTTCTATTTCATATCTTCAACAAGTTTAGTAATGAGCATAGCGGCC

CTATTGTTCCGATGGAGAGAAGAACCTATGATTAGCTTTTCGGGAAATTTCCAAACGAAC

AATTTCAACGAAATCTTTCAATTTCTTATTTTACTATGTTCAACTCTATGTATTCCTCTA

TCCGTAGAGTACATTGAATGTACAGAAATGGCTATAACAGAATTTCTGTTATTCGTATTA

ACAGCTACTCTAGGAGGAATGTTTTTATGTGGTGCTAACGATTCAATAACTATCTTTGTA

GCTCCAGAATGTTTTAGTTTATGCTCCTA-----CCTCTTATCTG-------G-ATATAC

CAAGAG---AGATGTACGG--TCTAATGA-GGCTACTATGAAATATTTACTCATGGGTGG

GGCAAGCTCTT-CTATTCTGGTTCATGGTTTCTCTTGGCTATATGGTTTATCCGGGGGGG

AGAT-CGAG-CTTCAAGAAATAGTGAATGGTCTTATCAATACACAAATGTATAACTCCCC

GGGAATTTCAATTGCGCTTATATTCATCACTGTAGGAATTGGGTTCAAGCTTTCCCTAGC

CCCTTTTCATCAATGGACTCCTGACGTATACGAAGGAGTGCGGTTCGTTCGACAAATTCC

TACCTCTATATCTATCTCT------GAGATGTTTGGATTTTTCAAAACTCCGTGGACATG

CAGAAGAGAAATGCTATCCCCACTCGGACCAAGACATAACTTTTACC-----AAAAGTTT

ATTGTGATCTTTTTGT----------TCAAATAACAATTAAGG----TGAAGCAGGGTCA

GGAACAACGAATC-TCTTTAT-GATAAACAGATCCATTTTGCAAGTTCGTTATTA--CGG

GTAGTTCCCACAAAAGATCGGACTAATG-ACGTATACAATACTTGAATTATCG-ATGTAG

ATGCTACATAGTTGGTTCTCAT-CCTTCAGAGACTACGAGTGTAATAGGAG---CATCCG

TCGACAAAAGGATCACC------------CTAAGATGAT---------------------

-CATCT--------CATGGCTATTGAGAACGAATCAAATCAGACGGTTCTATTTCTCAAT

C-TTTCTGACTTGCTCCTACGG--AACCAAAGTCGAAAAGATTG--AAAAAGTCAGTCAT

TCACAACCACTGATGAAGGATTCCTCGAAAAGTTAAGGAT-----TAGTAATCCTTTTTA

GAAATCG------------AATGGATTCAGTCTTATACATACGCGAGGAAGGTAATCAAA

AAAGAAAGAAGAAC----------------------------------------------

------------------------------------------------------------

-------------------TC-ATCTTCTTTCTTTTATCACTTAGGAGCCGTGCGAGATG

AAAGTCTCATGCACGGTTTTGAATGAGAGAAAGAAGTGAGG-AATCCTC-TTTTCGACTC

TGACTCTCCCACTCCA-GTCGTTGCTTTTCTTTCTGTTACTTCGAAAGTAGCTGCTTCAG

CTTCAGCCACGCGAATTTTCGATATTCCTTTTTATTTCTCATCAAACGAATGGCATCTTC

TTCTGGAAATCCTAGCTATTCTTAGCATGATATTGGGGAATCTCATTGCTATTACTCAAA

CAAGCATGAAACGTATGCTTGCATATTCGTCTATCGGTCAAATCGGATATGTAATTATTG

GAATAATTGTTGGAGACTCAAATGATGGATATGCGAGCATGATAACTTATATGCTGTTCT

ATATCTCCATGAATCTAGGAACTTTTGCTCGCATTGTATTATTTGGTCTACGTACCGGAA

CTGATAACATTCGAGATTATGCAGGATTATACACGAAAGATCC-TTTTTTGGCTCTCTCT

TTAGCCCTATGTCTCTTATCCCTAGGAGGTCTTCCTCCACTAGCAGGTTTTTTCGGAAAA

CTCCAT---------CTCTTCTGGTGTGGATGGCAGGCAGGCCTATATTTCTTGGTTTCA

ATAGGACTCCTTACGAGCGTTGTTTCTATCTACTATTATCTAAAAAT-------AATCAA

GTTATTAATGA--CTGGACGAAACCAAGAAATAACCCCTCACGTGCGAAATTATAGAAGA

TCTC------CTTTAAGATCAAACAATTCCATCGAATTGAGTATGATTGTATGTGTGATA

GCATCTACTATACCAGGAATATCAATGAACCCCATTCTTGCAATTGCTCAGGATACCCTC

TTTTAGATGTTTCTGCTTCACGAATAT--GATATTTTCTGGGCATTTCTACTA-----AT

ATCAAGTGTTATTCCTATTTTAGCATTTTTAATTTCCGGAGTTTTAGCCCCAACGAGGGA

AGGACCCGAGAAGCTTTCTAGTTATGAA-TCGGGTATAGAA-CCAATAGGGGATGCTTGG

G-TACAATTCCGAATCCGATATTATATGTTTGCTTTAGTTTTTGTTGTTTT--TGATGTT

GAAACGGTCTTTCTTTATCCATGGGCAATGAGTTTCGATGTATTGGGTGTATCCGTATTT

CTAGAAGCTTTGA--TTTTCGTGCTTATCCTAATTGTTGGTTCAGTTTATGCATGGCGAA

AGGGGGCATTGGAATGGTCTTAG---ACGAGTTATTTTCC------TTGGTTAACCATAA

TTGTTGTTTTTCCGATATCCGCAGGTCTTTCAATTTTCTTTCTCCCGCATAGAGGGAATA

AGGTGGTTCGGTGGTATACTATATGTATTTGTTTGC------TAGAACTCCTTTTAATGA

CCTA-----------TGTGTTCTGTTATCACTT-TCAATTGAACGATCCATTAATCCAAT

TGGATGAAGATTATGAATGGATAAATATTTTTGATTTTCACTGGAGACCAGGAATCGATG

GACTTTCCATAGGACCCATTCTACTGACGGGATTTATCACTACTTTAGCTACTTTAGCGG

CTTGGCCGGTTACTCGAGATTCGCGATTGTTCCATTTCCTGATGTTAGCAATGTACAGTG

GTCAAATAGGATTATTTTCTTCTCGAGACCTTTTACTTTTTTTTCTCATGTGGGAGTTAG

AATTAATTCC-CGTTTACCTACTTTTATCTATGTGGGGTGGGAAGAAACGGCTGTACTCG

GCTACCAAGTTTATTTTGTACACAGCGGGGGGTTCTGTTTTTCTTTTAATGGGAGTTCTG

GGTATGGGTTTA------TATGGTTCCAATGAACCAACATTAAATTTTGAAACATCGGCT

AATCAATCGTATCCTGTGTCATTGGAAATACTATTTTATTTTGGATTCCTTATTGCTTAT

GCTGTCAAATTACCGATTATACCCCTCCATACATGGTTACCAGATACCCATGGAGAAGCC

CATTACAGTACATGTATGCTTCTAGCTGGAATCTTATTAAAAATGGGAGCATATGGATTA

GTTCGGATCAATATGGAATTATTGCCCCACGCTCATTCTATATTTTCCCCCTGGTTGGTA

CTAGCGGGAACGTTGCAAATAATCTATGCAGCTTCAACTTCTCTTGGTCAAGTTAATTTA

AAAAAGAGAATCGCCTATTCCTCCGTATCTCATATGGGTTTCACAATTATAGGAATTGGT

TCTATAACCGATACAGGACTCAATGGAGCCATTTTACAGTTACTCTCTCATGGATTTCTT

GGTGCTGCACTTTTTTTCTTAGCGGGAACAAGTTGTGATAGAATACGTCTTATTTATCTC

GACGAAATGGGGGGAATATCC-------------ATCCCGATGCCCAAAATATTTACCAT

GTTCAGTAGCTTCTCAATGGCTTCTCTTGCATTGCCAGGAATGAGTGGTTTTGTTGCGGA

AGCAGTAGTATTTTTTGGAATAATTACTAGTCAAAAATTTCTTTTTTTGCCAAAAATACT

AATTACTTTTGTAATGGCAATTGGAATGATATTAACTCCTATTTATTTATTATCCATG--

------TTACGCCAGATCTTCTATGGATACAAGCTATTTAATATGACAAACTCTTATTTT

ATGGATTCTGGACCACGAGAACTATTTGTTTCGATCTGTATC-TTTTTGCCCGTAATA-G

GTATTGGTATTTAT---CCCGATT-TCGTTCTATCGTTATCAGTTGACAAGGTGGAAGCT

ATTTTATCTAATTACTTTTATAGATAGATGATGCTGGAGTATGTACTTTTTTTGAGTGCT

TATTTATTTTCCATCGGTATCTATGGATTGATTACAAGTCGAAACATGGTTAGAGCACTT

ATGTGTCTTGAACTTATACTGAATGCGGTTAATATCAATCTCGTAACATTTTCTGATTTA

TTTGATAGTCGCCAATTAAA----------------------------------------

-----------------------------------AGGAGACATTTTTTCGATCTTTGTT

ATAGCTATTGCAGCCGCTGAAGCAGCTATTGGACCAGCTATTGTTTCATCGATCTATCGT

AACAGAAAATCCATTCGTATTAATCAATCAAATTTGTTGAATAAATAGATGGAACATACA

TATCAATATGCATGGATAATACCTTTCCTTCCATTTCCAGTTACTATATCAATAGGGTTG

GGACTTCTGCTCGTTCCAACGGCAACGAAAAATCTTCGCCGTATTTGGGCTTTTTTTAGT

GTTTTATTGCTAAGTATAGCTATGGTGTTTTCGGCCGATCTGGCTATTCAGCAAATAAAC

GGGAGTTTTATCTATCAATATTCATGGTCTTGGACGATTAATAACACTTTTTCATTAGAG

TTTGGATACTTAATTGATCCACTTACCTCTATTATGTTAATACTAATCACTACTGTTGGA

ATCATGGTTCTTATTTATAGTGATAATTATATGTCTCATGATCAAGGATATTTAAGATTT

TTTGCTTATATGAGTTTTTTCAATACTTCTATGTTAGGATTAGTTACTAGTTCTAATTTG

ATACAAATTTATATTTTTTGGGAACTCGTAGGAATGTGCTCCTATTTATTAATAGGGTTT

TGGTTCACACGACCAACTGCAGCAAATGCTTGTCAAAAAGCGTTTGTAACTAATCGTGTA

GGGGATTTTGGTTTGTTATTAGGAATCTTAGGTCTTTATTGGATAACAGGTAGTTTCGAA

TTTCGGGATTTGTTCGAAATTTTAAAAAACTTGATC---C------ATAATAATGAAGTC

AATTCTTTATTTGCCGCCCTGTGCGCCTCACTATTATTTGTTGGTGCGGTTGCGAAATCC

GCACAATTTCCACTTCATGTATGGTTACCTGATGCTATGGAGGGCCCTACCCCCATTTCG

GCTCTTATACACGCTGCTACTATGGTAGCGGCGGGCATTTTTCTTGTAGCTAGGCTTCTT

CCTCTTTTCACAGTCATACCTTACATAATGAATTTCATTTCTTTGATAGGTATAATAACA

GTCCTTTTAGGGGCTACTTTAGCTCTTGCTCAAAGAGACATTAAAAGAAGTTTAGCCTAC

TCTACAATGTCTCAGTTAGGTTATATTATGTTAGCTCCAGGTATAGGCTCTTATCGAGCT

GCTTTATTCCATTTGATCACTCACGCCTATTCTAAAGCATTATTGTTTTTAGGATCTGGA

TCAATTATTCATTCAATGGAACCTATTGTTGGATATTCACCAGAGAAAAGTCAGAACATG

ATTCTTATGGGTGGTTTAAGAAGATATGTTCCAATTACAAAAACCACTTTTTTCTTAGGT

ACACTTTCTCTTTGTGGTATGCCCCCTCTTGCTTGTTTTTGGTCCAAAGATGAAATTCTT

AATGATACTTGGTTATATTCACCAATTTTCGCAATAATAGCTTGGTCCACAGCAGGATTA

ACTGCATTTTATATGTTTCGGGTATATCTCCTTACTTTTGATGGTCATTTACAAGTTCAT

TTTCAAAATTTCAGTAGTACTAAAAATAGCTCCTTCTATTCAA------TATCTATATGG

GGAAAAGAAGTACCGAAACCACTTAACGTAAAT----TTGTTTTTAT----CAAC-----

--------AAT--GAACACTAACGAAAAAA---TGTCTTTT------------TTTTCG-

-----AAAAATACATATCA----A--ATTGATAGAAATGGAAAAAACCGA----------

ATTCGTTACT---TTAGTACTCAGTTTGGAAATAAGTAC------ACTTCCATGTATCCC

CATGAATCGGACAATACTATGCTTTTTCCGATGCTTGTATTGGTCTTATTTACTTTGTTC

ATTGGATTTATAGGAATTCCTTTCGATCAA---------------GGAGTAATTGATTTG

GATATATTATCAAAATGGTTAACTCCATCGATA------AACCTTTTACATTCCAATTCC

GGCGATTCCTTTGATTGGTATGAATTTGTGACAAATGCAATTTATTCAGTCACTATATCC

TTTTTGGGGATATTTCTA-GCATACATATTTTATGGGTCTGTTTATTCATCTTTTCAAAA

TTTGGACTTAATCAATTCGTTTGTTCGAATAGATTCTAA---GAGAAT------TTTATC

GGATCGAATAATAAATGGGATATACAATTGGTCATATAATCGTGGTTACATCGACGTTTT

TTATGGAAAAGTTTTAAGTAATACTATAAGAGGACTTGCAGAATTAATTCATTTTTTTGA

TAGACGAGTTATTGATGGGATTACAAATGGGGTTGGTGTTGTAAGTTTCTTTGTAGGAGA

AGGGATAAAATCT---GTAGGGGGTGGACGAATCTCATCTTATATCTTCTTGTATGCATT

TTCTGTATCAATTTGT----------TTAATAA---------TTTATTATT---TTTTCA

GTTTCTAA--------------------------------------ATGGATTTACCTGG

ACCAATACATGATGTTCTTTTAGTATTTCTGGGATCAGGTCTTATATTAGGAGGTTTAGG

AGTGGTATTACTTACCAATCCAATTTATTCTGCCTTTTCATTGGGATTGG---TTCTTGT

TTGTATATCCTT-ATTCTATATTCTATCGAACTCCTATTTTGTAGCTGCTGCACAGCTCC

TTATTTACGTGGGAGCCGTAAATGTCTTAATCATATTTGCTGTGATGTTCATGAATGGTT

CAGACTATTCCAACGATTTCTATCTTTGGACCGTTGGGGATGGAGTCACTTCACTGGTTT

GTACAAG---------TATTCTTTTTTCACTAATTACT-ACTATCCTAGATACGTCATGG

TACGGAATTATTTGGAATACAGGATCGAACCAGATTGTAGAGCAGGACTTAACAAGTAAC

GTTCAACAAATTGGAATTCATTTATCAACAGATTTTTATCTTCCATTTGAACTCGTTTCA

ATAATTCTTTTAGTTGCCTTGATAGGTGCAATTACTATGGCGCGTCAGTAC---------

TAA---------------------ATGACTGTACCAGCTACAAG------ACAAGACCTT

ATGATAGTTAATATGGGTCCTCACCACCCATCAATGCACGGTGTTCTTCGCCTGATCGTT

ACTCTCGACGGTGAAGATGTTATTGACTGTGAACCCATATTGGGTTATTTACATAGAGGG

ATGGAAAAAATTGCAGAAAACCGAACAATTATACAATATCTGCCTTATGTAACACGTTGG

GATTATTTAGCTACTATGTTCACAGAAGCAATAACAGTAAATGGGCCCGAACAATTGGGA

AATATTCAAATACCTAAAAGAGCCAGCTACATCAGAGTAATTATGTTAGAGCTGAGTCGG

ATAGCTTCTCATTTGTTATGGCTTGGTCCATTTATGGCGGATATCGGTGCACAAACACCC

TTTTTTTATATTTTCAGAGAGAGGGAATTGATATATGATCTATTCGAAGCTGCTACAGGT

ATGCGAATGATGCATAATTATTTCCGTATAGGAGGAGTTGCTGCTGATCTACCTTATGGC

TGGATAGATAAATGTTTAGATTTCTGCGATTATTTTTTAACAGGAATTGTTGAATATGAA

AAGCTTATTACACAGAATCCTATTTTTTTGGAACGAGTTGAAAGAGTGGGCATTATTAGT

GGGGAGGAAGCCATAAATTGGGGTTTATCAGGACCCATGTTACGAGCTTCCGGAATAGAA

TGGGATCTTCGTAAAGTTGATAATTA--TGAGTGTTATAATGAATTTGATTGGGAAGTCC

AATGGCAAAAAGAAGGAGATTCATTAGCTCGTTATTTAGTCCGAATTAGTGAAATGAAAG

AATCCATAAAAATTATTCAACAGGCTCTAGAAGGAATCCCTGGGGGGCCCTATGAAAATT

TAGAGGTTCGGCGC------TTTGATAAAGTCAAGGATTCGGAAT----GGAATGATTTT

GAATATAGGTTTATTAGTAA--AAAGC-------CTTCGCCTACTTTTGAATTGGCGAAA

CAAGA---ACTTTATGTGAGAGTAGAAGCACCAAAGGGAGAATTAGGAATTTTTCTGATA

GGAGATAATAGTGTTTTCCCCTGGAGATGGAAAATTCGTCCACCCGGTTTCAT-------

---CAATTTGCAAATTCTTCCTCAACTAGTTAAAAGAATGAAATTGGCCGATATCATGAC

GATACTAGGTAGTATAGATATCATTATGGGAGAAGTTGATCGTTGAATGTTCCCTATGGT

GACTGGGTTCATGAATTATGGTCAACAAACAGTACGAGCTGCAAGGTACATTGGTCAAAG

TTTCATGATCACCTTATCGCACGCGAATCGTTTACCTGTAACTATTCAATATCCTTATGA

AAAATTGATCACATCAGAGCGTTTTCGTGGTCGAATCCACTTTGAATTTGATAAATGTAT

CGCTTGTGAAGTATGTGTTCGTGTATGCCCTATAGATCTACCCGTTGTTGATTGGAGATT

GGACACAGATGTTAGAAAA---AAACAATTGCTTAATTATAGTATTGATTTTGGAGTCTG

TATATTTTGTGGCAACTGCGTCGAGTATTGTCCAACAAACTGTTTATCCATGACTGAAGA

ATATGAACTTTCTACTTATGATCGTCACGAATTGAATTATAATCAAATTGCTTTGGGGCG

GTTACCGATGTCAGTAATTGAGGATTACACAATTCGAACAACTACAAATT------TGAC

TCCAATT---AAAATAGCTAAAAATAAACCCCTG---GGTTCACGAACGATTACTAATTA

A---------------AT------------------------------------------

---GCAGGGTCGTTTATCTGCTTGGCTAGTCAAGCATGAGCTAGTTCATAGATCTTTGGG

CTTCGATTACCAAGGAATAGAGATTTTACAAATAAAACCCGAGGATTGGGATTCCATTGC

TGTCATTTCATACGTATATGGTTACAATTATTTACGTTCCCAGTGTGCCTATGATGTAGC

ACCCGGCGGATTTTTAGCCAGTGTCTATCATCTTACGAGAATACAGTATGGTGTGGATCA

ACCGGAAGAGGTATGCATAAAAGTATTTGCTCCAAGGAGGAATCCTAAAATCCCGTCTGT

TTTCTGGATTTGGAGAAGTGCTGATTTTCAAGAACGGGAATCTTATGATATGTTGGGAAT

TTCTTATGAAAATCATCCGCGCCTTAAACGTATTTTGATGCCTGAAAGTTGGATAGGCTG

GCCCTTACGTAAGGATTATATTGCCCCCAATTTCTATGAAATTCAAGATGCTCATTGA--

------------------------------------------------------------

------------------------------------------------------------

----------------------------------------------------------AT

GAATTCTATTGAGTTTCCGTTACTTGACCGAACAACCCCAAATTCAGTTATTTCAACTAC

ATTGAATGATCTTTCGAACTGGTCAAGACTATCCAGTTTATGGCCGCTTCTCTACGGTAC

CAGTTGTTGCTTCATCGAATTTGCTTCATTAATAGGCTCGCGATTCGACTTTGATCGTTA

TGGATTGGTACCAAGATCGAGTCCTA-GGCAAGCGGACCTAATTTTAACAGCTGGCACAG

TAACAATGAAAATGGCTCCCTCTTTAGTGAGATTGTATGAGCAAATGCCTGAACCAAAAT

ATGTCATTGCTATGGGAGCTTGCACTATTACCGGAGGGATGTTCAGTACCGATTCTTATA

GTACCGTTCGGGGAGTCGATAAGCTAATTCCTGTCGATGTCTATTTGCCGGGCTGCCCCC

CTAAGCCAGAGGCAGTTATAGATGCTATAACAAAACTTCGTAAGAAGTTATCTCGAGAAA

TCTCTGAAGATAGAATGGGGT------CTCAACGTGAAAATCGATGTT----------TT

ACGACCAATCACAAATTTTATGTTCGACGCAGTACTCATACTGGAAATTACGATCAAGGA

TTACTCTATCAATCGCC---------------ACCTACTGCAGAAATACCTTCGGAAA--

----------------------CAGAACCCTTTTTCAAATACAAAAGTTCAGTATCTTCC

CGCGAATTAGGGAATGAATCAGGCAAGGAAGATGTTTCTATACAGAATAAATAA------

---------------------------------ATGC---AAAATCGAAATACCTTTTCT

TGGGTAAAGGAACAGATGACTCGATTCATTTCAGTATCGATCATGATATATGTAATAACT

CGGACATCTATTGCAAATGCATATCCCATTTTTGCACAACAGGGTTATGAAAATCCACGA

GAAGCAACTGGACGCATTGTATGTGCTAATTGCCATTTGGCGAATAAGCCCGTGGATATT

GAAGTTCCACAAGCCGTGCTTCCTGATACTGTATTTGAAGCAGTTGTTAGAATCCCTTAT

GATAAGCAACTGAAACAAGTTCTTGCTAATGGTAAAAAGGGGTCTTTGAATGTAGGGGCT

GTTCTTATTTTACCCGAGGGATTTGAATTAGCCCCCCCCGATCGTATTTCTCCAGAAATG

AAAGAAAAGATG---GGGAATCTGGCTTTTCAGAGTTATCGCCCTACTAAAAAAAATATT

ATTGTGATAGGTCCTGTTCCGGGTCAGAAATATAGTGAAATCGTCTTTCCTATTCTTTCC

CCCGACCCTGCTACAAAGAAAGATGTTCACTTCTTAAAATATCCCATATATGTGGGCGGG

AACAGGGGAAGGGGTCAGATTTATCCCGATGGGAGCAAGAGTAACAATACAGTCTATAAT

GCTACAGCCGCTGGTGTGGTAAGCAGAATCCTACGTAAGGAAAAG---GGGGGTTATGAA

ATAACCATAGCTGATGCATCGGATGGACATCAAGTGGTTGATATTATACCTCCAGGACCA

GAACTTCTTGTTTCGGAGGGTGAATCCATTAAGCTTGATCAACCATTAACGAGTAATCCC

AATGTGGGCGGATTCGGGCAGGGCG-ATGCA--GAAATAGTACTTCAAG-ATCCATTACG

CG-TCCAAGGTCTTTTGTTCTTCTTGGCATCTGTTATTTTGGCACAAATCTTTTTGGTTC

TTAAAAA-GAAACAGTTTGAAAAGGTTCAATTGTACG-AAATGAATTTTTAGATGAGTGT

GTGACTTGTTATAA-TTGATCTTATTG-ATAGTACAGAGAATGGATCTGTCATC---TCG

ATAGAGATGATTCT-ATCTCGTCGGATA-------TTCAT----TCTAGTATCTGGAGCA

CGG-----AATATATAGA---------ATAGATC-----AAGAAATATTTGAACTATGAT

TCATACCTACTGT-------TCAAACCTCGCGACCGGACTCAAAAAAAAATTATCCAAGG

GA-------TA-----------TTTTGAAAATCAAACGAT---TTTTCTTCCTTCAGAAT

TGTGCTTA-TTTATTTTGGCTGAAGGACAGT--AGTTTTTTATTTGGATTT---TGGGTC

ATT----ACA-TCCATTCATCA-AAT-----AGGTGATAATCAAAG-----GGTTCTT-A

CTCAGAGAACCTTTGGGCTTAGCTTGGAATTT------ATCGAATCATCGTGGTTATAGT

ATGAATCTGAGGTTACAAT-----CGATTCGTGGGGTCTGAACAAGAGAATTCCTATTAA

TCAAT--------------------------------------------ATGTAAATCTG

CATTACGCACAAACAAAAAAAAAATCCAAT----------AAATAGGAAAATAGAAGATT

CAAGAGGCC----T-GTAACGCG--------ATCAACAT---------AAAGAAAGGCGG

ATGAGCTAACTTGA------------------TATTTTGGCATTAT--------CATCTC

AAAGAAGAGATTCCGGATATTGGTTA--CTTGTTCGGATCTTCACAGGGAAGGGAAGATT

GAATCAA-----GTGGCTAA-------GAGGTTTCAAACTTTCTATTCTAT---------

ATCCGTTG-CAACCACTATTTG------GTTGGTTCCGCTTGAGCCGTACGAGATGAAAT

TCTCATATCCGGTTCTTAGAGGGGGAAGTCTTCTCGGTTTACCTATCTCAATAAAGTATA

TGATTGGTTCGAGGAACGTCTCGAGATTCAGGCGATTGCAGATGATATAACTAGTAAATA

TGTTCCTCCTCATGTCAACATATTTTATTGTCTAGGAGGGATCACACTTACTTGTTTTTT

AGTACAAGTAGCTACGGGCTTTGCTATGACTTTTTACTATCGTCCAACTGTTACAGATGC

TTTTGCATCTGTTCAATATATAATGACTGAGGTCAACTTTGGTTGGTTAATACGATCAGT

TCATCGATGGTCAGCAAGTATGATGGTACTAATGATGATCCTGCACGTATTTCGTGTGTA

TCTTACAGGTGGATTTAAAAAACCCCGTGAATTGACTTGGGTTACAGGTGTGGTTCTGGG

TGTATTGACTGCATCTTTTGGTGTAACTGGTTATTCTTTACCTTGGGACCAAATCGGTTA

TTGGGCAGTAAAAATTGTTACAGGCGTACCTGACGCTATTCCTGTAATAGGATCTCCTTT

GGTAGAGTTATTACGTGGAAGTGCTAGTGTGGGTCAATCCACTTTGACTCGTTTTTATAG

TTTACACACTTTTGTCTTACCTCTTCTTACTGCCGTATTTATGTTAATGCACTTCCCAAT

GATACGTAAGCAAGGGATTTCAGGTCCTTTATAGATGGGAGTGTGTGACTTGAACTA-TT

GATTGGGCCGTGCAGATATA-GAATTTTATCCGCCACATTGGAATTCCGAACC-----CA

ATGTGTCTCCATATCCAAC-----CACCG-----CATAATTCCCCC--------CGCGGA

GCATAGCATAGGCCGGTTCGCTTGAGGAGAATCTTTTCTATGATCATACCTGA--AT-CA

TGTGATGCATGAACAGGCTCCGTAAGATCCCGTA-----------GAATAAGTGATATGG

AATGATCCAGATTATGTTCTATCTATTCCACATAC-------------------------

--------------------------------------------------TTAATAGTAG

GGAAATGCATTCATTTCTTCTGCATTAATCCCGCCGATCTATGATACTATCGGAGTGAAA

CAAGGGATCTAAGGAAGAGCAGAGGCTAGACTTT----------GTTAGTACCAAGTAAA

TCC--------TTTGTATGTAATAAGA--TTCGAA---ATAGTGCGGGGATAAGCACA--

-----AATTACAAGACATGAGACGATCCAAAAAGCA-CTTGATCAC-------GATCAAG

TTTGTAAGCCTACTTGGGTATTGAGCATTTACCTGTAA-----GAACGGAATGCCTTTCA

ATGCA-----TG-------GTTCCAACTTTGGAAAACAGAATCCGGTAAATCTTTT----

-------CTTACATAGAATCATTGTATAT--------------GTGTAGATA-GAGCCGA

AATATATATTTTC---------------------------TAGGGATCCATTTCTTGCAT

TCCTTTGATTC-----TTGCTCGAGCCGGATGATGAAAAATTATCATGTCCGGTTCCGTA

GGGGGATGGATTTA-------TAAGAATTCACCTATCCCAATAACAAAGAAACCTGACTT

GAATGATCCTGTATTAAGAGCTAAGTTGGCTAAAGGGATGGGACATAATTATTATGGAGA

ACCCGCGTGGCCCAACGACCTTTTATATATCTTTCCAGTAGTAATTTTAGGTACTATTGC

GTGTAATGTGGGCCTGGCGATTCTAGAACCATCAATGATTGGTGAACCCGCGGATCCATT

TGCAACTCCTTTGGAAATATTACCCGAATGGTACTTTTTTCCCGTATT------------

------------TCAAATACTCCGTACAGTTCCCAATAAGTTACTGGGTGTTCTTTTAAT

GGCTTCAGTACCGCTGGGATTATTTACAGTACCCTTTTTGGAGAATGTTAATAAATTCCA

AAATCCCTTTCGTCGTCCAGTAGCTACAACAGTCTTTTTGATCGGTACTGCAGTAGCCCT

TTGGTTAGGTATTGGAGCCACATTACCTATTGATAAATCCCTAACTTTA-GGTCTTTTTT

AA----------------------------------------------------------

-----ATGATTGAAGTTTTTCTATTTGGAATCGTATTAGGTCTAATTCCTATTACTTTGG

CAGGATTGTTCGTAACTGCATATTTACAATACAGACGCGGTGATCAGTTGGACCTTTGA-

--ATGCCTACTATAACTAGTTATTTCGGTTTTCTACTGGCTGCTTCAACTATAACCACAG

CTCTATTCATTGGTCTGAGCAAGATACGGCTAATTTGAATGGATATTGTAAGTCTTGCTT

GGGCTGCTTTAATGGTAGTCTTTACATTTTCCCTTTCACTCGTAGTATGGGGAAGAAGTG

GACTCTAGATGATTATTCGTTCGCCGGAACCAGAAGTGAAAATTGTGGTAGATAGGGATC

CCATAAAAACTTCTTTCGAGGCATGGGCCAGACCCGGCCATTTTTCAAGAACAATAGCTA

A---AGGCCCTGATACTACCACTTGGATCTGGAACCTACATGCTGATGCTCACGATTTCG

ATAGCCATACCAGTGATTTGGAGGAGATTTCTCGAAAAGTATTTAGTGCTCATTTCGGTC

AACTCTCCATCATCTTTCTTTGGCTGAGTGGCATGTACTTCCATGGCGCCCGTTTTTCTA

ATTATGAAGCATGGCTGAGTGATCCTACTCACATTAGACCCAGTGCCCAGGTAGTTTGGC

CAATAGTGGGTCAAGAAATTTTGAATGGTGATGTGGGCGGGGGTTTCCGCGGAATACAAA

TAACCTCTGGTTTTTTTCAAATTTGGCGAGCATCTGGAATAACTAGTGAATTACAACTTT

ATTGTACCGCAATTGGCGCGTTGGTCTTTGCAGCGTTAATGCTTTTTGCAGGTTGGTTCC

ATTATCACAAAGCTGCTCCAAAATTGGCTTGGTTCCAAGATGTCGAATCTATGTTGAATC

ACCACTTAGCGGGGTTACTAGGACTGGGGTCTCTTTCTTGGGCGGGACACCAAATACATG

TATCTTTGCCGATTAACCAATTTCTCGACGCGGGAGTGGATCCTAAAGAGATACCACTTC

CTCATGAATTTATATTGAATCGGGATCTTTTGGCTCAACTTTATCCAAGTTTTGCCGAAG

GAGCAACTCCATTTTTCACCTTGAATTGGTCAAAATATGCAGAATTTCTTACTTTTCGGG

GAGGATTAGATCCTATAACAGGCGGCCTATGGTTGAGCGATATTGCGCACCATCATTTAG

CTATTGCAATTCTTTTCCTGATAGCTGGTCATATGTATAGAACCAACTGGGGCATTGGTC

ATGGACTAAAAGATATTTTGGAGGCTCATAAGGGTCCATTTACGGGCCAAGGTCATAAAG

GCCTTTATGAAATCC-TAACCACG--TCATGGCATGCTCAATTAGCTCTTAACCTGGCTA

TGTTAGGCTCTTTAACCATTGTTGTAGCTCACCATATGTATTCCATGCCCCCCTATCCAT

ACCTAGCTACTGACTATGGTACACAACTTTCGTTGTTCACACATCACATGTGGATCGGTG

GATTTCTCATAGTTGGTGCTGCTGCACATGCAGCCATTTTTATGGTAAGAGACTACGATC

CAACTACTCGATACAACGATCTATTAGATCGTGTCCTTAGGCACCGTGATGCAATCATAT

CACATCTAAACTGGGCATGTATATTTCTAGGCTTTCACAGTTTTGGTTTGTATATTCATA

ATGATACCATGAGTGCCTTAGGGCGCCCCCAGGATATGTTTTCAGATACCGCTATACAAT

TACAACCCATCTTTGCTCAATGGGTACAAAACACCCATGCTTTAGCACCTGTTGCGACAG

CTCCTGGTGCAACAACAGGCACCAGCTTAGCTTGGGGGGGTGGTGAGTTAGTAGCAGTAG

GCGGCAAAGTAGCTTTGTTACCTATTCCATTAGGAACTGCGGATTTCTTGGTACATCACA

TTCATGCATTTACGATCCATGTGACTGTATTGATACTACTGAAAGGTGTTCTGTTTGCTC

GGAGCTCCCGTTTAATACCTGATAAAGCGAATCTTGGCTTTCGTTTCCCTTGTGATGGTC

CTGGAAGAGGAGGAACATGTCAAGTATCCGCCTGGGATCATGTCTTCTTAGGTCTATTCT

GGATGTACAATTCAATTTCGGTAGTCATATTCCATTTCAGTTGGAAAATGCAATCGGATG

TTTGGGGTAGTATAAGCGATCAAGGGGTGGTAACTCATATCACAGGAGGAAACTTTGCAC

AGAGTTCCATTACTATTAATGGGTGGCTTCGGGATTTCTTATGGGCACAGGCATCTCAGG

TAATTCAGTCTTATGGTTCTTCATTATCTGCATATGGTCTTTTTTTCCTAGGTGCTCATT

TTGTATGGGCTTTCAGTTTAATGTTTCTATTCAGCGGCCGTGGTTATTGGCAAGAACTTA

TTGAATCCATCGTTTGGGCTCATAACAAATTAAAAGTTGCTCCTGCTACTCAGCCTAGAG

CCTTGAGCATTGTACAAGGACGTGCTGTAGGAGTAACTCATTACCTTCTGGGTGGAATTG

CCACAACATGGGCATTCTTCTTAGCAAGAATTATTGCAGTAGGATAAATGGCATTAAGAT

TTCCAAGGTTTAGCCAAGGCTTAGCTCAGGACCCCACTACTCGTCGTATTTGGTTTGGTA

TTGCTACCGCACATGACTTCGAGAGTCATGATGATATTACTGAGGAACGTCTTTATCAGA

ACATTTTTGCTTCTCACTTTGGTCAGTTAGCAATAATCTTTCTGTGGACTTCCGGAAATC

TGTTTCATGTAGCTTGGCAAGGAAATTTTGAGTCATGGGTACAGGACCCTTTACACGTAA

GACCTATTGCTCACGCAATTTGGGATCCTCATTTTGGTCAACCGGCTGTCGAAGCCTTTA

CTCGAGGAGGTGCTCCTGGACCAGTGAATATCGCTTATTCCGGCGTTTATCAGTGGTGGT

ATACAATCGGATTACGCACCAATGAAGATCTTTATACTGGAGCTCTTTTTCTATTATT--

----TCTTTCTGCTATATCCTTAATAGGGGGTTGGTTACACCTACAACCCAAATGGAAAC

CAAGCGTTTCTTGGTTCAAAAATGCGGAATCCCGTCTGAATCATCATTTGTCAGGACTTT

TCGGAGTAAGTTCCTTGGCTTGGACAGGGCATTTAGTTCATGTCGCTATTCCCGCATCA-

--AGGGGCGAGTATGTCAGATGGAATAATTTCTTAGATGTATTACCGCACCCCCAAGGTT

TGGGACCACTTTTTAGCGGTCAGTGGAATCTTTATGCCCAAAACCCCGATTCCAGTAGTC

ATTTATTCGGTACCTCCCAAGGAGCGGGAACTGCCATTCTAACTCTTCTCGGGGGATTCC

ATCCGCAAACGCAAAGTTTATGGCTGACCGATATGGCTCATCATCATTTAGCTATTGCAT

TTATTTTCCTGATAGCTGGT-CATATGTATAGAACCAACTTCGGGATTGGGCACAGTATA

AAAGATCTTTTAGAAGCACATACTCCTCCGGGGGGTCGATTGGGGCGTGGGCATAAGGGT

CTTTATGATACAATCAATAATTCGCTTCATTTTCAATTAGGTCTTGCTCTGGCCTGTTTA

GGAGTTATTACTTCCTTGGTAGCTCAACACATGTACTCTTTACCTGCTTACGCATTCATA

GCGCAAGACTTTACTACTCAAGCTGCGTTGTATACTCATCACCAATACATCGCAGGTTTC

ATCATGACGGGAGCCTTTGCTCACGGAGCTATATTCTTCATTAGAGATTACAATCCGGAA

CAGAATGAAGATAATGTATTGGCAAGAATGTTAGACCATAAAGAAGCTATCATATCTCAT

TTAAGTTGGGCCAGTCTGTTTTTGGGGTTCCATACCTTGGGACTTTATGTTCATAACGAC

GTCATGCTCGCTTTTGGGACTCCGGAAAAACAAATCTTGATTGAACCTATATTTGCCCAG

TGGATACAATCCGCTCATGGTAAGACTTTATATGGGTTTGATGTACTCTTATCTTCAACG

AGTGGCCCAGCATTCAATGCTGGTCGAAGCATTTGGTTACCCGGTTGGTTGAGTGCTGTT

AATGAGAATAGTAATTCACTATTCTTAACAATAGGTCCTGGAGACTTCTTGGTTCA-TCA

TGCTATTGCTCTAGGTTTGCATACAACTACATTGATCTTAGTAAAGGGTGCTTTAGATGC

GCGTGGTTCGAAGTTAATGCCAGATAAAAAAGATTTCGGTTATAGTTTTCCTTGCGACGG

CCCGGGACGGGGGGGTACTTGTGATATTTC-TGCTT--GGGATGCATTTTATTTGGCGGT

TTT-CTGGATGTTAAATACCATTGGATG-GGTTACTTTTT-ATTGGCATTGGAAGCACAT

CACATTATGGCAAGGTAATGTTTCACAATTTAACGAATCCTCTACTTATTTGATGGGATG

GTTAAGAGATTATCTATGGTTAAACTCTTCCCAACTTATCAACGGATACAATCCGTTTGG

TATGAATAGTTTATCGGTCTGGGCATGGATGTTCTTATTTGGACATCTTGTTTGGGCTAC

TGGATTTATGTTCTTAATTTCCTGGCGTGGATATTGGCAGGAATTAATTGAAACTTTAGC

ATGGGCTCATGAACGCACACCTTTGGCTAATTTGATTCGATGGAGAGATAAGCCAGTGGC

TCTTTCCATCGTGCAAGCAAGATTGGTTGGATTAGCCCACTTTTCCGTAGGTTATATATT

CACTTATGCAGCTTTCTTGATTGCCTCTACATCAGGTAAATTTGGTTAAATGTCACATTC

AGTAAAGATTTATGATACATGTATAGGGTGTACTCAATGTGTACGAGCCTGTCCTACAGA

TGTATTGGAAATGATACCCTGGGACGGATGTAAAGCTAAGCAAATTGCTTCTGCGCCAAG

AACAGAGGACTGTGTAGGTTGTAAGAGATGCGAATCCGCCTGTCCAACAGACTTCTTGAG

TGTCCGGGTTTATTTAGGGAATGAGACAACTCGCAGCATGGGTCTACCTTATTGAATGAC

AGATCTCAA---------------------------------------------------

---------CTTACCCTCTATTTTTGTGCCTTTAGTAGGCCTAGTCTTTCCGGCAATTGC

AATGGCTTCTTTATTTCTTCATGTTCAAAAAAACAAGA----------------------

-------------------------------------------TTGTCTAAATGCAAGAT

ATAAAAACATATCTCTCTGTGGCACCTGTGCTAACTACTCTATGGTTTGGGTCTTTAGCA

GGTTTATTGATAGAGATCAATCGTTTATTCCCGGATG------CCTTGTCATTCCCATTT

TTT------------------TAAATGACTGCAATTTTAGAGAGACGCGAAAGTACAAGC

CTATGGGGTCGCTTCTGTAACTGGATAACCAGCACCGAAAACCGCCTTTACATTGGATGG

TTCGGTGTTTTAATGATCCCTACCTTATTGACCGCAACTTCTGTATTTATTATCGCCTTC

ATCGCTGCTCCTCCAGTAGATATTGATGGTATCCGTGAACCTGTTTCCGGGTCTTTACTT

TATGGAAACAACATTATTTCTGGTGCCATTATTCCTACATCTGCAGCTATAGGTTTGCAC

TTTTACCCAATTTGGGAAGCAGCATCCGTTGATGAGTGGTTATACAATGGTGGTCCTTAT

GAACTAATTGTTCTACACTTCTTACTTGGTGTAGCTTGTTATATGGGTCGTGAGTGGGAA

CTTAGTTTCCGTCTGGGTATGCGTCCTTGGATTGCTGTCGCATATTCAGCTCCTGTTGCA

GCTGCTACTGCTGTTTTCTTGATCTACCCTATTGGTCAAGGAAGCTTCTCTGATGGTATG

CCTCTAGGAATATCTGGTACTTTCAACTTTATGATTGTATTCCAGGCTGAACACAACATC

CTTATGCATCCATTCCACATGTTAGGCGTAGCTGGTGTATTCGGCGGCTCCCTATTTAGT

GCTATGCATGGTTCCTTGGTAACCTCTAGTTTAATCAGGGAAACTACTGAAAATGAATCT

GCTAATGAAGGTTACAGATTCGGTCAAGAGGAAGAAACTTATAATATCGTCGCTGCTCAC

GGTTATTTTGGCCGATTAATCTTCCAATATGCTAGTTTCAACAATTCCCGTTCCCTACAT

TTCTTCTTGGCTGCTTGGCCTGTAGTAGGTATCTGGTTCACTGCTTTAGGTATTAGTACT

ATGGCTTTCAACCTAAACGGTTTCAATTTCAACCAATCCGTAGTTGACAGTCAAGGTCGT

GTTATTAACACTTGGGCTGATATCATCAACCGTGCTAACCTTGGTATGGAAGTAATGCAT

GAACGTAATGCTCACAACTTCCCTCTAGACCTAGCTTCTGTTGAAGCACCTTCTACAAAT

GGCTAAATGGGTTTGCCTTGGTATCGTGTTCATACCGTCGTATTGAATGATCCCGGTCGG

TTGCTTTCTGTCCACATAATGCATACAGCTCTAGTTGCTGGTTGGGCCGGGTCAATGGCT

CTATACGAATTAGCGGTTTTTGATCCCTCTGACCCCGTTCTTGATCCAATGTGGAGACAA

GGTATGTTCGTTATACCCTTCATGACTCGTTTAGGAATAACCAATTCGTGGGGTGGTTGG

AGTATCACAGGAGGAACTATAACGAATCCGGGTATTTGGAGTTACGAAGGTGTGGCAGGG

GCACATATTGTGTTTTCTGGCTTGTGCTTTTTAGCAGCTATTTGGCATTGGGTATATTGG

GACTTAGAAATATTCTGTGATGAACGTACGGGAAAACCCTCTTTGGATTTGCCCAAGATC

TTTGGAATTCATTTATTTCTCTCAGGAGTGGCTTGCTTTGGCTTTGGCGCATTTCATGTA

ACAGGCTTGTATGGTCCTGGAATATGGGTGTCTGATCCTTATGGACTAACCGGAAAAGTA

CAACCCGTAAATCCAGCTTGGGGCGCGGAAGGTTTTGATCCTTTT-GTTCCGGGC--GGA

ATAGCCTCTCATCATATTGCAGCGGGGACTTTGGGCATACTGGCAGGCCTATTCCACCTT

AGTGTCCGTCCACCCCAGCGTCTATACAAAGGATTACGTATGGGCAATATTGAAACTGTA

CTTTCGAGTAGTATCGCTGCTGTGTTTTTTGCAGCTTTCGTAGTTGCGGGAACTATGTGG

TATGGTTCAGCAACTACCCCCATCGAATTATTTGGTCCGACGCGTTACCAGTGGGATCAA

GGATACTTCCAGCAAGAAATATATCGAAGAGTTGGTGCTGGACTAGCCGAAAATCTGAGT

TTATCGGAAGCTTGGTCTAAAATTCCTGAAAAATTAGCTTTTTATGATTACATCGGTAAT

AATCCGGCAAAAGGTGGATTATTTAGAGCAGGCTCAATGGACAACGGAGATGGAATAGCC

GTTGGATGGTTAGGACACCCCGTCTTTCGAGATAAGGAAGGGCGTGAACTTTTTGTACGC

CGTATGCCTACTTTTTTTGAAACATTTCCGGTAGTTTTGGTAGATGGAGACGGAATTGTG

CGAGCCGATGTTCCTTTTCGAAGGGCAGAATCCAAATATAGTGTCGAACAAGTAGGCGTA

ACTGTTGAGTTCTATGGTGGCGAACTCAATGGAGTCAGTTATAGCGATCCTGCTACTGTT

AAAAAATATGCTAGACGTGCCCAATTAGGTGAAATTTTTGAATTAGATCGCGCTACTTTA

AAATCCGATGGTGTTTTTCGTAGCAGTCCGAGGGGTTGGTTCACTTTTGGACATGCTTCA

TTTG-CTTTACTCTTC--TTTTTCGGGCACATTTGGCATGGAGCTAGAACCTTGTTCAGA

GATGTTTTTGCTGGTATTGATCCAGATTTGGATGCTCAAGTGGAATTTGGGGCATTCCAA

AAAATTGGAGATCCAACTACAAGGCGACAAGCAGTTTGAATGAAAACCTTATATTCCCTG

AGGAGGTTCTACCCCGTGGAAACGCTCTTTAATGGAACTTTAGCTTTAGCTGGTCGTGAC

CAAGAAACCACTGGTTTCGCTTGGTGGGCCGGGAATGCCAGACTTATCAATTTGTCCGGT

AAACTACTCGGGGCTCACGTAGCCCATGCCGGATTAATCGTATTCTGGGCAGGAGCAATG

AACCTCTTTGAAGTGGCTCATTTCGTACCAGAGAAGCCCATGTATGAACAAGGATTAATT

TTACTTCCCCATCTAGCTACTCTAGGTTGGGGGGTAGGTCCGGGTGGGGA-AGTTATAGA

CACCTTTCCATACTTTGTATCTGGAGTACTTCATCTAATTTCCTCTGCAGTCTTAGGCTT

TGGCGGTATTTATCATGCGCTTCTTGGACCTGAGACTCTAGAAGAATCCTTTCCGTTCTT

CGGTTATGTATGGAAAGATAGAAATAAAATGACCACAATTTTAGGTATTCATCTAATTTT

GTTAGGGATAGGTGCTTTTCTTCTAG---TATTCAAGGCTCTTTATTTTGGGGGTGTATA

TGACACCTGGGCTCCTGGGGGGG---GAGATGTAAGAAAAATAACCAACTTAACACTTAG

CCCAAGTGTTATATTTGGTTATTTACTAAAATCTCCCTTTGGAGGAGAAGGATGGATTGT

TAGTGTGGACGATTTAGAAGATATAATTGGAGGACATGTATGGTTAGGTTCCATTTGTAT

ACTTGGTGGAATCTGGCATATCTTGACCAAACCCTTTGCATGGGCTCGCCGCGCATTTGT

ATGGTCTGGAGAGGCTTACTTGTCTTATAGTTTAGGTGCTTTAGCTGTCTTTGGGTTCAT

CGCTTGTTGTTTCGTCTGGTTCAATAATACCGCCTATCCTAGTGAGTTTTATGGACCTAC

TGGGCCGGAGGCTTCTCAAGCTCAAGCATTTACTTTTCTAGTTAGAGACCAACGTCTTGG

GGCTAACGTGGGATCCGCTCAGGGACCTACTGGTTTAGGTAAATATCTAATGCGGTCCCC

AACGGGAGAGGTCATTTTTGGAGGAGAAACCATGCGCTTTTGGGATCTCCGTGCTCCTTG

GTTGGAACCTCTAAGGGGTCCCAATGGTTTGGACTTGAGTAGGCTGAAAAAAGACATACA

ACCTTGGCAAGAACGACGTTCGGCGGAATATATGACTCATGCTCCTTTAGGGTCTTTAAA

TTCCGTGGGTGGTGTAGCTACCGAGATCAATGCAGTCAATTATGTCTCTCCTAGAAGTTG

GTTAGCTACTTCTCATTTTGTTCTAGGATTCTTCTTTTTTGTGGGTCATTTGTGGCACGC

AGGAAGAGCCCGCGCAGCGGCAGCAGGATTTGAAAAAGGAATCGATCGTGATTTAGAACC

TGTTCTTTCCATGACTCCTCTTAATTGAATGACTATAGCCCTTGGTAGATTTACCAAAGA

GGAAAATGATTTATTCGATATTATGGACGACTGGTTACGGAGAGACCGTTTCGTTTTTGT

AGGTTGGTCCGGTCTATTGCTCTTCCCTTGTGCTTATTTCGCTTTAGGAGGTTGGTTCAC

AGGTACAACTTTTGTAACTTCGTGGTATACCCACGGATTGGCTAGTTCATATTTAGAAGG

CTGCAATTTCTTAACCGCTGCAGTTTCTACTCCTGCTAATAGTTTAGCACATTCTTTGTT

GCTACTATGGGGTCCTGAAGCACAAGGCGATTTTACTCGTTGGTGTCAATTAGGCGGTCT

GTGGACTTTTGTTGCTCTACACGGTGCTTTCGGACTAATCGGTTTCATGTTACGTCAATT

CGAACTTGCTCGATCTGTTCAATTGCGACCTTATAATGCAATCGCATTTTCTGGTCCAAT

TGCTGTTTTCGTTTCTGTATTCCTAATTTATCCACTGGGTCAGTCTGGTTGGTTCTTTGC

ACCCAGTTTTGGTGTGGCAGCTATATTTCGATTCATCCTCTTCTTCCAGGGGTTTCATAA

TTGGACCCTAAACCCATTTCATATGATGGGAGTTGCCGGAGTATTGGGTGCTGCTCTGCT

ATGCGCTATTCATGGTGCTACCGTAGAAAATACTTTATTTGAGGACGGTGACGGCGCAAA

TACATTCCGTGCTTTTAACCCAACGCAAGCCGAAGAGACTTATTCGATGGTCACTGCTAA

CCGATTTTGGTCCCAAATCTTTGGGGTTGCTTTTTCTAATAAACGTTGGTTACATTTCTT

TATGTTATTTGTACCAGTAACCGGTTTATGGATGAGTGCCCTTGGAGTAGTTGGTCTGGC

TCTGAACCTACGTGCCTATGACTTC-GTTTCCCAGGAAATCCGTGCCGCGGAAGATCCTG

AATTTGAGACTTTCTACACCAAAAATATTCTCTTAAATGAGGGTATTCGTGCTTGGATGG

CAGCTCAGGATCAGCCTCATGAAAACCTTATATTCCCTGAGGAGGTTCTACCCCGTGGAA

ACGCTCTTTAAATGTCTGGAAGCACGGGAGAACGCTCTTTTGCTGATATTATTACCAGTA

TTCGATACTGGGTTATTCATAGCATTACTATACCTTCCCTATTCATTGCGGGTTGGTTAT

TCGTCAGTACAGGTTTAGCTTACGATGTGTTTGGAAGTCCTCGTCCAAACGAGTATTTTA

CAGAAAGCCGGCAAGGGATTCCATTAATAACTGGCCGTTTTGATCCTTTGGACCAACTTG

ATGAATTTAGTAGATCCTTTTAGATGACCATAGATCGAACCTATCCAATTTTTACAGTAA

GATGGTTGGCTGTTCATGGACTAGCTGTACCTACCGTTTCTTTTTTGGGGTCAATATCAG

CAATGCAGTTCATCCAACGATAA---------ATGGCTACACAAACCGTTGAGGGTAGTT

CTAGATCTG------GGCCAAGACGAACAATTGTAGGGGATTTATTGAAACCATTGAATT

CAGAATATGGTAAAGTAGCCCCGGGATGGGGAACTACTCCTTTTATGGGAGTCGCAATGG

CCCTATTTGCGATATTCCTATGTATTATTTTGGAGATTTATAATTCTTCCGTTTTACTGG

ATGGAGTTTCACCGAAT------TAG------------------ATG-----------CT

TACTCTCAAACTCTTCGTTTACACAGTAGTGATATTTTTT---------------GTTTC

TCTATTTATCTTCGGATTCCTATCTAATGATCCAGGACGTAATCCTGGACGTGAGGAA--

----TAAATGGCTGATACTA---------------------CTGGA---AGGATTCCTCT

TTGGCT---GATAGGTACTGT-AGCTGGTAT-TCCTGT-GATCG-GTTCAGTGGGTGTTT

T-------------CTTTTACGGTTCA-TATTCCGGATTGGGCTCATCTCTGTAGATGCT

TAATA---TACTTAATTTGATCTGTATCTGTCTTAATTCTGCCC---CTTATTCGAGTA-

-----GTTTTTTCTGCGCTAAATTGCCCGAGGCTTATGCTGTTTTCAATCCAATTGTAGA

TTTTATGCCAGTCATACCTGTACTCTTTTTTCTCTTAGCCTTTGTTTGGCAAGCCGCTGT

AAGTTTTCGCTGAATGACACAAT---------CAAACCCAAACGAACAAAATGTTGAATT

GAATCGTACCAGTCTATACTGGGGGTTATTACTCA-TTTTTGTACTTGCTGTTTTATTTT

CCAATTATTTCTTCAATTGAATGGAAGTAAATATTCTCGCATTTATTGCTACTGCACTGT

TCATTCTAGTTCCGACTGCCTTTTTACTTATCATCTACGTAAAAACAGTCAGTCAAAATG

AT---------TAAATGGAAACAGCAACCTTAGTCGCCATCTTCATATCTGGTTTACTTG

TAAGCTTTACTGGGTATGCCTTATATACCGCTTTTGGGCAACCCTCTCAACAACTAAGAG

ATCCATTCGAGGAACACGGAGACTAG---------ATGGAAGCATTGGTTTATACATTCC

TGTTAGTCTCGACTCTAGGGATAATTTTTTTCGCTATCTTCTTTCGAGAACCGCCAAAGG

T---------TCCTACTAAAAAGATGAAATGAATGACTATTGCTTTTCAATTGGCTATTT

TTGCATTAATTGCGACTTCATCGATCTTATTGATTAGTGTACCCGTTGTATTTGCTTCTC

CTGATGGTTGGTCAAGTAACAAAAATGTTGTATTTTCCGGTACCTCATTATGGATTGGGT

TAGTCTTTCTGGTAGGTATCCTTAATTCGCTCATCTCTTGA-------------------

--ATGTCACCACAAACAGAGACTAAAGCAGGTGTTGGATTCAAAGCTGGTGTTAAAGATT

ACAAATTGACTTATTATACTCCTGAATATGAAACCAAAGATACTGATATCTTGGCAGCAT

TTCGAGTAACTCCTCAACCCGGAGTTCCACCCGAGGAAGCAGGGGCTGCGGTAGCTGCCG

AATCCTCTACTGGTACATGGACAACTGTGTGGACCGATGGACTTACCAGCCTTGATCGTT

ACAAAGGCCGATGCTACCACATCGAGCCCGTTGTTGGGGAGCAAAATCAATATATTGCTT

ATGTAGCTTATCCTTTAGACCTTTTTGAAGAAGGTTCTGTTACTAACATGTTTACTTCCA

TTGTGGGTAATGTATTTGGGTTCAAAGCCCTACGAGCTCTACGTTTGGAGGATCTTCGAA

TTCCCCCTGCTTATTCCAAAACTTTCCAAGGCCCGCCCCATGGAATCCAGGTTGAGAGAG

ATAAATTGAACAAGTATGGTCGTCCCCTATTGGGATGTACTATTAAACCAAAATTGGGGT

TATCCGCGAAGAACTACGGTAGAGCAGTTTATGAATGTCTCCGTGGTGGACTTGATTTTA

CCAAGGATGATGAGAACGTGAACTCACAGCCATTTATGCGTTGGAGAGACCGTTTCTTAT

TTTGTGCTGAAGCAATTTATAAAGCGCAAGCGGAAACAGGTGAAATCAAAGGACATTATT

TGAATGCTACTGCGGGTACATGTGAAGAAATGATGAGAAGGGCCCAATGTGCCAGAGAAT

TGGGAGTTCCTATCGTAATGCATGACTACTTAACAGGTGGATTCACTGCAAATACTAGCT

TGGCTATTTATTGCCGAAACAACGGCCTACTTCTTCACATCCATCGTGCAATGCATGCAG

TTATTGATAGACAGAAGAATCATGGTATGCACTTTCGTGTACTAGCTAAAGCGCTACGTA

TGTCTGGTGGAGATCACATTCACGCCGGTACAGTAGTAGGTAAACTGGAAGGGGAACGTG

AAATGACTTTAGGTTTTGTTGATTTACTACGTGATGATTATATTGAAAAAGACCGAAGTC

GTGGTATTTTTTTCACTCAAGATTGGGTCTCTATGCCAGGTGTTTTGCCCGTAGCTTCCG

GTGGTATTCACGTTTGGCATATGCCTGCCTTGACCGAGATCTTTGGGGATGATTCTGTAC

TACAGTTCGGTGGAGGAACTTTAGGACACCCTTGGGGAAATGCACCTGGTGCAGTAGCCA

ATCGGGTAGCTTTAGAAGCGTGTGTACAAGCTCGTAATGAGGGACGTGATCTTGCTCGCG

AAAGTACTCAAATTATCCGT-----------------------GAAGCTTGTA-------

-------AATGGAGCCCTGAGCTAGCCGCTGCTTGTGAAGTATGGAAGGAAATCAAATTT

GA---GTTCGAACCAGTAGATAAGCTAGATGTTAAAAAAAACTAAACGGCGATACATTTA

TACAAAACTTCTACCTCGAGCACACGCAATGGAGCCGTA---------GACAGTCAAGTG

AAATCCAATCCACGAAATAATTTGATCTATGGACAGCATCATTGTGGTAAAGGTCGTAAT

GCCAGAGGAATCATTACCGCAGGGCATAGAGGGGGAGGTCAT-AAGCGTCTATACCGTAA

AATCGATTTTCGACGGAATAAAAAAGACATATCTGGTAGAATCGTAACCATAGAATACGA

CCCTAATCGAAATGCATACATTTGTCTCATACACTATGGGGATGGTGAGAAGAGATATAT

TTTACATCCCAGAGGGGCTATAATTGGAGATACCATTGTTTCTGGTACAGAAGTTCCTAT

ATCAATGGGAAATGCCCTACCTTTGAGTGCGGTTTGAACTATTGATTTACGTAATTGGAA

GTAACCAATTAGGTTTACGACGAAACCTAGAAATCGATCACTGATCCAATTGGAGTACCT

CTACAGGATAGACCTCAACAGAAAACTGAAGAGGAACGGCAGCAAGTGATTGAGTTCAGT

AGTTCCTCATATAAAATTATTGACTCTAGAGATATGGTAATATGGAGAAGAC-----AAA

ATTGATTGTTTGAAGCACGGACAGAACCGGA-AGCGCCCCTTGTTTCAAAGAGAGGAGGA

CGGGTTATTCACATTTAATTTGATGGTCAGAGGCGAATTGAAAGCTAAGCAGTGGTAATT

AT-------------AAGGATCCCCCAGGGGAAAAATAG---------------------

----------AGATGTCTCCTACGTTACCCGTAATATG-----TGGAAGTATCGACGTAA

TTTCATAGAGTCATTCGGTC--TGAATGCTACATGAAGAACATAAGCCAGATGATGGAAC

GAGGAGACCTAGGATGTAGAAGATCATACCATGAGTGATTCGGCAGATTTGGATTC----

-CTATATATCCACTCCTGTGGTACTTCATC---ATATGATTCATATAAGATCCATCTGTC

TAGATATCATCATATACATCTAGAAAGCCGTATGCTTTGGAAGAAGCTTGTACAGTTTGG

GAAGGGGTTTTTATTGATCAAAAAGAAGAATCTACTTCAACCGATATGCCCTTAGGCACG

GCCATACATAACATAGAAATCACACTTGGAAAGGGTGGACAATTAGCTAGAGCAGCGGGT

GCTGTAGCGAAACTGATTGCAAAAGAGGGTAAATCGGCCACATTAAGATTACCATCCGGG

GAGGTCCGTTTGATATCCAAAAACTGCTCAGCAACAGTCGGACAAGTAGGTAATGTTGGG

GCGAACCAACAAAGTTTGGGTAGAGCCGGATCTAAGTGTTGGCTAGGTAAGCGTCCTGTA

GTAAGAGGAGTAGTTATGAACCCTGTGGACCATCCCCACGGGGGTGGTGAAGGGAGAGCC

CCAATTGGTAGAAAAAAACCCACAACCCCTTGGGGTTACCCTGCGCTTGGAAGAAGAAGT

AGGAAAAGGAATAAATATAGTGATCGTTTTATTCTTCGTCGCCG--TAAA----------

------TAGATGATTCAACCTCAGACCCTTTTAAATGTAGCGGATAACAGCGGGGCTCGA

GAATTGATGTGTATTCGAATCATAGGA---GCCAGTAATCGCCGATATGCTCATATTGGT

GACGTTATTGTTGCTGTAATCAAAGAAGCAGTACCCAATATGTCTCTAGAAAGATCAGAA

GTGGTCAGAGCTGTAATTGTACGTACATGTAAAGAACTCAAACGTGACAACGGTATGATA

ATACGATATGATGACAATGCAGCAGTTGTCATTGATCAAGAAGGGAATCCAAAAGGAACT

CGAGTTTTTGGTGCGATCGCTCGGGAATTGAGACATTTGAATTTCACTAAAATAGTTTCC

CTAGCTCCTGAGGTATTATAAATGCTTAGTGTGTGACTCGTT-A-----ATTTTTCTTTA

GATT---------TATTAGGATTCCAAAAATG--------------------GACCGGAC

CGTTATG-----------------------------------------------------

-AACTAATAACTATAGAA-----------------CTAATAACCAACTTATTGCTTCG--

---TGTTATCGAGATA----CAAAAGAAGAATTGCTATATGA------------------

----------------------------------TGTAT-CAATCATATAGTTATAGCAA

CTGAAATACTATTTATTTCCCTAAAAGAAAAATCAATCTTA-----TTATG--GGTTATG

AAAAAATA------GGTATGTG--GATA-AATGAAAGGGTGAGAG-AAAGAGAGAAGGAG

AATCT---CA-------ATGATATATGATTCTAA--TA--TGTATGGTCTATGAATCACC

TCCTA-----AAAGGCAATGTAATAACGCATCA-AT------------------------

------------------------------------------------------------

-------------------------------------------T----------------

------------------CAATACCCATAGGAAAAAT----------------ACAAATA

A---TAAAGAGCCCCAAAGTCAATAGAGACTGAGAAGATTGACTCAGGA-----------

ACTTAGTTGGGAGCTCCATTGCAGAGTTCGGGCCTAGCCA-TGAAT---AGAGAAGCTAT

AGGAACGATGGAACCTGTGACTGCATAAGATTCTATTGAATGAAAACGAATTCTAATGAT

TCATTAGGTGGGATGGCGGAACGAACCAGG-AACCAATTGATTTATTCGTAGAGGT----

----CATACATACGCTACGAG-TGAAGCAG--------GAAAGAGTCAATATTCGCCCGC

GAAAGCCTTATTGA----------------------------------------------

-ATTCAATATTTTGGGCAT--------TAAAGTA-----------------AAAAAAAAA

CATT---------------------------------------------ATCCATAATAA

ATTAA-ATAGAACGAAGTT-----------------------AAATA-------------

------------ATGAGAAAGAAAAATCTATATCTAG-----------------------

ATCTTGTATATACAGCT---------------------------------TCCTACATAA

C------AGATTCAATA--------TCAAC------------------------------

------------------------------------------------------------

-------------------AA-------ATACCA--CGATTTTTTGTATTATTCAT----

TAAATACACCTGTAT-----CTGTAATATTTTTGAAT-----------------------

------------------------------------------------------------

-----------TCATATTCATTCGCGAGGAGCTGGATGAGAAGAAACTCTCATGTCCGGT

TCTGCAGTAGAGATGG-----AATTGAGAA--ATAACCATCAACTATAACCCCAAAAGAA

CCAGATTCCGTAAACAACATAGAGGAAGAATGAAGGGAATGTCTTAT---CGAGGCAGTC

ATATTTGTTTCGGAAGATACGCTCTTCAGGCACTTGAACCATCTTGGATCACATCTAGAC

AAATAGAAGCGGGGCGAAGGGCAATGACACGATATGCACGTCGTGGTGGAAAAATCTGGG

TACGTATATTTCCGGACAAACCCGTTACAATAAGACCCGCGGAAACACGTATGGGTTCGG

GGAAGGGATCCCCCGAATACTGGGTATCCGTTGTTAAACCGGGTCGAATACTTTATGAGA

TGGGTGGAGTATCAGAAACTGTAGCCAGAGCCGCTATTGAAATAGCCGCGTCCAAAATGC

CTATAAGAACGCAATTTATTA---TTGCGGGA------TAGATGACCAGAGTTAGGCGAG

GATATATAGCTCGGAGACGTCGAACAAAAATTCGTTTATTTGCAGCAACCTTTCGAGGGG

CTCATTCAAGACTTACTCGAGCTGCCACTCAACAGAAAATGAGAGCTTTGGTTTCTGCTC

ATCGAGATAGAGGCAAGCAAAAGAGAGATTTTCGTCGTTTGTGGATCACTCGGATAAATG

CGGTAACTCGTGAGAATGG---GGTATGCTATAGTTATAGTCGATTAATGCACAATCTGT

ATAAGAGGCAGTTGCTTCTTAATCGTAAAATACTTGCACAAATAGCTATATTAAATAAGA

ATTGTCTTCACATAATTTCCAATGA------G-ATCATC-AAATAA--------------

---------ATGATAAAGAAAAATTGGATTA--------CTACGGGTACGGAAG------

---------CACGGG---------------------------------CTTTAGCTC---

-------------------AAAATATATCTATGTCTGCTTACAAAGCGAGAAGAGTAATT

GATCAGATTCGTGGGCGTTCCTATGAGGAAACAATTATGATACTAGAACTGATGCCTTAT

CGAGCATCTTTTCCTATTTTAAAATTGGTTTACTCTGCAGCAGCAAATGCTATTAACAAT

ATGGGTTTGAGCGAAGCTGATTTATTCATTAGTAAAGCAGAAGTCAATGGAGGTACTTTC

GTGAAAAAGTTACGACCCCGAGCTCGGGGACGTAGTTATGCGATAAAAAGGCCCACTTGT

CATATAACAATTGTATTGAAGG-------ATAAAG--------ATAAATCGCTT------

---------------------TTAAATGAATAA------------------ATGGATGGA

ATCAAATATGCAGTATTTACCGAAAAAAGTATTCGGTTATTGGGGAACAATCAATATACT

TCTAATGTCGAATCAGGATCAACTAGGACAGAAATAAAGCATTGGGTCGAACTCTTCTTT

GGCGTCAAGGTAATAGCTATGAATAGTCATCGAC------TCCCGGGAAAGGGTAGAAGA

GTGGGACCTATTAAGGGACATACAATGCATTACAGACGTATGATCATTACGCTTCAACCG

GGTTATTCTATTCCACCTCT-TATAGAGAAAAGAACTTAAATGGCGGTTCCAAAGAAACG

TACTTCTATATCAAAAAAACGTATTCGTAAAAATTTTTGGAA--GAAAAAGGGATATTTT

G-CCGCGGTAAAAGCTTTTTCT-------TTAGCTAAATCAG------TTTCCACCGG--

----------ACAGTCAAAAAGTTTTT-TTGTGCGACAAACAA---GTAATAAATCCTTG

GAA------------------------------------------TAAATGGCCAAGGGT

AAAGATGCCAGATTAACAGTTATTTTGGAATGTACCA---GTTGTGTCCG------AAAT

GGTGTCAAT------AAGGAATCGCCGGGTATTTCCAGATATATTACTCAAAAAAATCGA

CACAATACATCGAGTCGATTAGAATTGAGAAAATATTGTCGCTATTGTTATAAGCATACG

ATTCACGGGGAAATAAAGAAA------TAGATGAAAATAAGAGCCTCTGTTCGTAAAATT

TGCGAAAAATGTCGACTAATCCGTAGGCGGGGACGAATTATAGTTATTTGTTATAATCCG

AAACATAAACAAAGACAGGGGTAAATGGTTCGAGAGGAAGTAGTAGGATCCACTCGGGCA

CTACAGTGGAAGTGTGTTGAATCAAGAGCAGACAGTAAGCGCCTTTATTATGGTCGTTTC

A-TTCTGTCC--CCACTTCTGAAAGGACAAGCCG-ATACTATA-GGTATC-GCCATGCGA

AGGG--CTTTACTTGGAG-AAATAGAAGGAA--CATGTATC----ACACGTGCAAAATCT

----GAGAAGGTACCACATGAATATTC--------TACGATAGCGGGCATTGAAGAATCA

GTACATGAAATTTTAATGAATTTGAAAGAAATTGTATTGAGAAGTAATCTTTATGGAACT

CGAGACGCATCTATTTGCGTCAGAGGTCCTAGATGCGTAACTGCTCAAGACATCATCTCA

CCACCTTCCGTAGAAGTAGTTGATACTACACAACATATAGCTAGCCTGACGGAACCGATT

GATTTGTGTATTGGATTACAAATCCAGAGGGATCGCGGATATCGTATGAAAACCACAAAA

AACTCTCAAGATGGAAGTTA----------TCCGATAGATGCTGTATCCATGCCTGTTCG

AAATGCGAATCATAGTATTCATTCTTATGGAAATGGTAATC------AGAAACAAGAGAT

ACTTTTCCTCGAAATATGGACAAATGGAAGTTTAACTCCTAAAGAAGCACTTCACGAAGC

TTCCCGTAATTTGATTGATTTATTTATTCCTTTTCTACATGCAGAGGAAGAGGA---CAT

------TAATTTCGAGGAAAATCAAAACAGGTT---TACTGTACCCCCTTTTACCTTTCC

TGATAGATTGGCT------AATCTAAAGAAAAACAAAAAAGAAATTGCATTGAAATGTAT

TTTTATTGACCAATCAGAATTGCCTCCCA-GGACCTATAATTGTCTCAAAAGGTCCAATA

TACATACATTATTGGACCTTTTGAGTAACAG------TCAAGAAGATCTTATGAAAATTG

AATATTTTCGAATAGAAGATGTAAAACAGATATTGGACACTCTA-CAGAAGCATTTCGC-

---AATTGATTTA--CCTAAGAATA------AGTTT---------TAA------------

---------------ATGGGATTTCTTTTTTCGATAAACAGGAAACTTAAGATGCCCCGG

GA---TGGAAATGAGGGAATGTTCACAATACCTGGATTTAGTCAGATCCAATTTGAGGGA

TTTTGTAGGTTCATTGATCAGGGCTTGATGGAAGAATTTCATAAGTTTCCAAAAATTGAA

GATACAGACCAAGAAATTGAATTTCAATTATTTGTGGAAAGATATCAATTGGTAGAACCC

TTGATAAAAGAAAGAGATGCTGTATATGAATCACTCACATATTCCTCGGAATTATATGTA

CCCGCGGGATTAATTTGGAAAACCGGTAGAGATATGCAAG--AACAAACCATATTTATTG

GAAACATTCCTCTAATGAATTCCCTGGGAACCTTTATAGTAAATGGAATATACAGAATTG

TAATCAATCAAATATTGCAAAGCCCCGGTATTTACTACCGTTCAGAATTGGACCATAACG

GAAT---TTCTGTTTATACCAGTACCATAATATCAGATTGGGGAGGAAGATCAGAATTAG

AAATCGATAGAAAATCAAGGATATGGGCCCGTGTGAGTAGGAAACAAAAAATATCTATTC

TAGTTTTATCATCAGCTATGGGTTCGAATCTAAGAGAAATTCTAGATAATGTTTGCTACC

CCGAAATTTTC------------TTGTCTTTTCTGAATGATAGGGAGAAA-------AAA

AAGA-----TTGGGTCAA-AAGAAAATGCCATTTTGGAATTTTATCAACAATTTGCTT--

-----GTGTA--GGTGGAGATCCGGTATTTTCTGAATCCTTATGTAAAGAATTACAAAAG

AAATTTTTTCAACAAAGATGTGAATTAGGAAGGATTGGACGACGAAATATGAACCGGAGA

TTAAATCTTGATATACCTCAGAGCAATACATTTTTGTTACCACGAGATGTATTGGCTGCC

GCGGATCATTTGATCGGAATGAAATTTGGAATGGGTACACT------TGACGATATGAAT

CACTTGAAAAATAAACGTATTCGTTCTGTAGCAGATCTGTTACAGGATCAATTCGGCTTG

GCTCTTGTTCGTTTAGAAAATGCAGTTCGAGGAACTATATGTGGAGCA------------

---ATCAGGCATAAATTGATACTGACTCCTCAAAATTTGGTCAGTTCAACTTC-------

-----ATTAACAACCACTTATGAATCGTTTTTCGGCCTACACCCTTTATCTCAAGTTTTG

GATCGAACTAATCCATTGACGCAAATCGTTCATGGGCGAAAATTGAGTTATTTGGGTCCT

GGAGGATTGACAGGGCGAACTGCTAGTTTTCGAATACGAGATATCCATCCTAGTCACTAT

GGACGGATTTGCCCAATTGACACGTCCGAAGGAATCAATGTTGGACTTATTGGATCCTTA

GCTATTCATGCCAGGATTGGTCAT---TGGGGGTCTATAGAGAGTCCGTTTTATGAAGTA

T---ATCAGAGATC------AAAAGAGAC---AAAGATGGTTTTTTTATCACC------A

AGTAGAGATGAATATTAT------ACGGTAGCGACAGGAAATTCTTTGGCTTTGAATCGA

GGTGGTATTCAGGAAGAACAGATTGTTCCCGCCCGATACCGTCAAGAATTCCTGACTATT

GCATGGGAACAGATTCATCTTCGAAGTATTTTTCCCTTCCAATATTTTTCTATTGGAGCT

TCCCTGATTCCGTTTATCGAGCATAATGATGCGAATCGGGCTTTAATGAGTTCGAATATG

CAGCGTCAAGCAGTTCCGCTTTCCCGGTCCGAGAAGTGCATTGTTGGGACTGGATTGGAA

CGACAAGCGGCTCTGGATTCGGGGGTTTCAGCTATAGCCGAATGCGAGGGAAAGATTATT

CATACCGATACTCATAAGATCGTTTTATCAGGTCATGGAGACACTATAAGCATTCCATTG

GTTATGTATCAACGTTCGAACAAAAATACTTGTATGCATCAAAATCCTCAGGTTCGGCGG

GGGAAATGCATTAAAAAAGGACAAATTTTAGCGGATGGTGCGGCTACAGTTGGTGGCGAA

CTCGCTTTAGGAAAAAACGTATTAGTAGCTTATATGCCATGGGAAGGTTACAATTTTGAG

GACGCGGTACTCATTAGCGAACGTCTGGTATATGAAGATATTTATACTTCTTTTCACATC

CGTAAATATGAAATTCAGACTCATGTGACAAGCCAGGGTC---CTGAAAGAATTACTCAT

GAAATACCGCATTTGGAAGCTCATTTACTCCGCAATTTAGACAGAAATGGAATTGTGGCG

CTGGGATCTTGGGTAGAAACAGGCGATATTTTAGTAGGTAAATTAACCCCTCAGACAGCT

AACGAATCATCGTATGCCCCGGAAGATAGATTATTACGAGCCATACTTGGGATTCAGGTA

TCCACTGCAAAAGAAACTTGTCTAAAACTACCTATAGGCGGAAGGGGTCGAGTTATTGAT

GTGAGATGGATCCA----------------------------GAAAAAG-----------

---------------------GGAGGTTCCAGCTA-------------------------

--------------------TAATCCAGAAACGATTCGTGTATATATTTCACAGAAACGT

GAAATCAAAGTGGGTGATAAAGTAGCTGGAAGACACGGGAATAAAGGTATCATTTCCAAA

ATTTTATCTAGACAAGATATGCCTTATTTGCAAGATGGAACGCCGGTTGATATGGTTTTC

AACCCATTAGGAGTACCTTCACGAATGAATGTGGGACAAATATTTGAATGCTCGCTCGGG

TTAGCGGGGGACCTGCTAGACAGACATTATAGAATAGCACCTTTTGATGAGAGATATGAG

CAAGAGGCTTCGAGAAAACTAGTGTTTTCTGAATTATATGAAGCCAGTAAGCAAACAGCA

AATCCATGGGTATTTGAACCCGAATATCCCGGAAAAAGCAGAATATTTGATGGAAGAACG

GGAGATCCTTTTGAACAGCCTGTTCTAATAGGAAAGTCCTATATCCTGAAATTAATTCAT

CAAGTTGACGATAAAATCCACGGTCGTTCCAGTGGACATTACGCACTTGTTACACAACAA

CCTCTTAGAGGAAGGGCCAAACAGGGCGGACAGCGCGTAGGAGAAATGGAAGTTTGGGCT

CTCGAAGGGTTTGGTGTTGCTCATATTTTACAAGAGATGCTTACTTATAAATCTGATCAT

ATTAGAGCTCGTCAGGAAGTACTTGGTACTACAATCATTGGAGGAACAATACCTACCCCC

GAGGATGCTCCAGAATCTTTTCGATTGCTCGTTCGCGAATTACGATCTTTGGCTTTGGAA

CTGAATCATTTCCTTGTATCTGAGAAGAACTTCCAGATTAATAGGAAGGAAGCT------

---TGA---------------------ATGATCGACC---GGTATAAACATCAACAACTT

CAAATTGGATCAGTTTCTCCTCAACAAATAAGTGCTTGGGCCAACAAAATCCTACCTAAT

GGAGAGATAGTTGGAGAGGTCACAAAACCCTATACTTTTCATTACAAAACCAATAAACCG

GAAAAAGATGGATTATTTTGTGAAAGAATCTTTGGACCTATAAAAAGTGGAATTTGCGCT

TGTGGAAATTA-----------------------TCGAGTAATTGGCGCTGAAAAAG---

-AAGACCCCAAATTTTGTGAACAATGTGGAGTCGAATTTATTGATTCTCGGATACGAAGA

TATCAAATGGGATACATCAAGCTCGCATGTCCAGTGACTCATGTGTGGTATTTGAAACGT

CTTCCTAGTTATATCGCGAATCTTTTAGATAAACCCCTTAAGGAATTAGA------AGGC

CTAGTATACTGCGATGTGTGATTTGATCG-AAATTATCATTTTACAGATTCGGACTGAGA

AACTGTCATCCCATTCAATCCGACG---GGGGTGCCCTTGGCTCTGACATGTATCTTGGG

AGGAGTAACATGAAGCTTAGAATTTTGGATGCATTCAATACTTC----C-AAATAAAAGG

GGAATTGATCCATGGTCGAT---T-CTCTAACAG----ATAA-ACGGGAATTGCTAGTTG

TACCTCGTGAAAAAAA--------AAAAAGACTTTTTTCGTCAAATT------TGCCATT

TCATTTCTTTT--------------AGAGAG--ATTTTGTTCAAGTAAGC--AAACA---

----GCAGCATGGTTACAGGAGTCTATCTATCGCATATATG-----CTTCAAAGGACATC

GTG-GCATA-------ACCATCGAGGTGAAGTAGAG---------ACCT--AAAAGATCG

AATGGAACGATAGATAGTATCGACAAGTAAATTCCTTATGAGTTTCAAGGTATTCCTCG-

----AGAGGAATTCATTATTCGAAGGGAAGTAGACTACTCAATA-----ATTTCACATTC

CACTTATCTT-ATGTC------GTAGTCGTAATTGGATAAGTAATTCAGAAAGTAAAAG-

-TCAAAGGACAGATG-AATCA-ATGAAATATTGGTTAGTC----CTAACCGGG--AACTT

GAGTAAGGAGTAG-------CTGTTTGTTTTGTATTGTAGGGTTTTTTCTCGAACAAAGT

TTGAAAAC----GCGAACCCTCACTTTTTATTTAGTGTAACTACTTTAGCCGGATGAGAG

GAAACCTTCACGTCCGGTTTTTA-AGGGGGGAATCC-----TCTAGGAACCTATCCCTAT

TTTTCTTTTGCTAGGCCCATAGCTAAAAAACCGACTTTCTTACGATTACGAGGTTTATTC

GAATATGAAA---TCCAATCCTGGAAATACAGCATCCCCCTTTTTTTTAC----------

-----------TACCCAAGGCTTCGATACATTTCGAAATAGAGAAATCTCTACCGGAGCG

GGTGCTATCAGGGAACAATTAGCCGATTTGGATTTGCGAATTGTTATCGATAATTCATCG

GTAGAATGGAAGGACTT---------------------------AGGAGACGAAGGGTCC

------ACTGGGAATGAATGGGAAGATAGAAAAATTGGACGAAGAAAGGATTTTTTAGTT

AGACGCATGGAATTAGCTAAACATTTTATTCGAACAAATGTAGAACCAGAGCGGATGGTT

TTGTGCCTATTACCAGTTCTTCCTCCCGAGTTGAGACCAATTATTCAGATAGATGGGGGT

AAACTAATGAGT---TCGGATATTAATGAACTCTATAGAAGAGTTATCTATCGGAACAAT

ACTCTTACCGATCTATTAAAAA--CAAGTAGATCTACGCC-------------AGGGGAA

TTAGTAATGTGTCAGGAGAAATTGGTGCAAGAAGCCGTAGATACACTTCTTGATAATGGA

ATCCGCGGACAACCAATGAGGGATGGCCATAATAAAGTGTACAAGTCATTTTCAGATGTA

ATTGAAGGCAAGGAGGGGAGATTTCGTGAGACTTTACTTGGTAAACGGGTCGATTATTCG

GGTCGTTCCGTCATTGTCGTAGGCCCTTCACTTTCATTACATCGATGCGGATTACCTCGA

GAAATAGCAATAGAGCTTTTCCAGACATTTGTAATTCGCGGTCTAATCAGACAACATCTT

GCTTCTAACATAGGAATTGCTAAAAGCAAAATTCGGGAAAAAGAACCGATCGTATGGGAA

ATACTTCAAGAAGTTATGCAGGGACATCCTGTATTGCTAAATAGAGCGCCCACCCTGCAT

AGATTAGGCATACAGGCGTTCCAACCCGTTTTAGTGGAGGGACGTGCTATTTGTTTACAT

CCATTAGTTTGTAAGGGATTCAATGCAGATTTTGATGGAGATCAAATGGCTGTTCATGTA

CCTTTATCTTTGGAAGCTCAAGCAGAGGCTCGTTTACTTATGTTTTCTCATATGAATCTC

TTGTCACCAGCTATTGGAGATCCCATTTCTGTACCAACTCAAGATATGCTTATTGGCCTC

TATGTATTAACGATGGGGAATCGTCGAGGTATTTTTGTAAATAGGTATAATCCA------

TGTAATCGCAGAAATTATCAAAATAAAACAGTTGACAATAATA---------ACTATAAA

------CATACGAAAGAGAAAAAACCCTATTTTTTGAGTTCCTATGATGCACTTGGAGCT

TATCAGCAGAAACGAATCAATTTACATAGTCCTTTGTGGCTCCGGTGGCGAC---TAGAT

CAACGTGTCATTG------GTTCA------AGAGAAGTTCCTATCGAAGTTCAATATGAA

TCTTTGGGTACCTATCAGGAGATTTATGGGCACTATCTAATAGTAAGAAGTGTAAAAAAA

GAAATACTTTGTATATACATTCGAACCACTGTTGGTCATATTTCTTTTTATCGAGAAATA

GAAGAATC----------CGTACAAGGGTTTTGTCGAG--CCTACTCATATG--------

--------------GTACCTAA------------ATGGAGGTACTTATG--GCAGAACGG

GC---------------CGATCTGGTCTATCACAATAAAGCGATAGATGGAACTGCCATG

AAACGACTTATTAGCAGATTAATAGATCACTTCGGAATGGCATATACATCACACATCCTG

GATCAAGTAAAGACTCTGGGTTTTCGGCAAGCCACTGCTACATCTATTTCATTAGGAATT

GATGATCTTTTAACAATACCTTCTAAAGGATGGCTAGTCCAAGATGCTGAACAACAAAGT

TTTATTTTGGAGAAACACCATTCTTATGGAAATGTACACGCGGTAGAAAAATTACGTCAA

TCCATTGAGATATGGTATGCTACAAGTGAATATTTGAGACAAGAAATGAATCCTAATTTT

CGGATGACTGATCCTTCTAATCCAGTCCATATAATGTCCTTTTCGGGAGCTAGAGGAAAT

GCATCTCAGGTGCACCAATTAGTAGGTATGAGAGGACTAATGTCGGATCCCCAGGGACAA

ATGATTGATTTACCCATCCAAAGCAATTTACGTGAAGGACTTTCTTTAACCGAATATATA

ATTTCCTGCTACGGAGCCCGCAAAGGAGTTGTAGATACTGCTGTACGAACATCAGATGCC

GGATACCTCACTCGTAGACTTGTTGAAGTAGTTCAACACATTGTTGTACGCAGAACAGAT

TGTGGCACTATCCGAGGTATTTCTGTGCAACC---------TCGAAA------CGGGATG

ACGGAAAGGATGTTTTTTCAAAC------ACTAATCGGTCGTGTATTAGCAGACGATGTA

TATATAGGTCTACGATGCATTGCCGCTCGAAATCAAGATATTGGGATTGGACTTGTCAAT

CGATTCATAACC------------------TTTCGAGCACAACCAGTATATATTCGAACT

CCCTTTACTTGTAGGAGTACCTCTTGGATCTGTCAATTATGTTATGGCCGGAGTCCTACT

CACGGCGACCTGGTCGAATTGGGGGAAGCGGTAGGTATTATTGCGGGACAATCAATTGGG

GAACCGGGGACTCAACTAACATTAAGAACCTTTCATACTGGTGGAGTATTCACAGGGGGT

ACTGCAGAACATGTACGAGCTCCTTCTAATGGAAAAATAAAATTCAATGAGGAGTTGGTT

CATCCCACACGTACACGTCACGGACATCCTGCTTTTCTATGTTCTATAG---ACCTGTAT

GTAACTGT---TGAGGGTCGGGATATTATACATAATGTCAATATTCCACCAAAAAGTTTG

ATTTTAGTTCAAAATGATCAATATGTAGAATCAGAACAAGTGATTGCCGAGATTCGCGCC

GGAACATCCACTTTTAATTTTAAAGAGAGGGTCCAGAAACATATTTATTCCGAATCAGCG

GGAGAAATGCACTGGAGTACCGATGTCTATCATGCACCTGAATATACATATGGTAATGTT

CATCTATTACCAAAAACAAGTCATTTATGGATATTATCCGGAGGGCCATACAGA------

TCCAGTATAG---TGTCTTCGTCGCTCCACAAGGATCAAGATCAAACGAATGCTCATTCT

TTTTCTGTTGAACGGCGA------TATATTTCTGACCTCTCAATGACTA---ATGGTCGA

GTGAGACACAAATTATTTAGTTCGGATCTTTCTGGTAAAAG---------GGGGGATAGG

ATTCTTGATTATTCA---AG---ACCCGA--TCAAATCATATCC------AAGGGTCATT

GGAATTTTATATACCCTTC------TATTCTCCACGAGAATTCAGATTTCTTAGCGAAGA

GGCGAAAAAATAGATTCATCATCCCGTTCCAATATGATC----AAGAAGGGGAGAAAGAA

CTAA----------TACCGCATT------CTGGTATTTCGATCGAAATACCCATAAACGG

TATGTTGCGTAGAAATAGTATTCTTGCTTATTTTGACGATACACGATACAGAAGAAGCA-

-----GTTCCGGAATTACTAAAT-------------------------------------

------------------------------------------------------------

------------------------------------------------------------

------------------------------------------------------------

------------------------------------------------------------

------------------------------------------------------------

------------------------------------------------------------

--------------------------------------------------ACGGGACCGT

------------------------------------------------------------

------------------------------AGAGATAGATTCAATCGTTAAAAAAGAGGA

TTTCATTGAGTATCGAGGAACAAAAGAATTTAGTTCGAAATAC------CAAATGAAAGT

AGATCGATTTTTTTTCATTCCCGAAGAAGTGCATATCTTACCTGGATCTTCGTCCATAAT

GGTCCGGAACAACAGTCTCATCGGAGTAGACACACGAATCACTTT------AAATATAAG

AAGCCGAGTGGGTGGATTGGTTCGAGTGGAGAGAAAAAAAAAAAATATTGAACTCAAAAT

TGTTTCTGGAGATATCCATTTTCCTGGAGAGACAGATAAGATATCCAGGCACAGCGGTAT

CTTGATACCGCCAGGAACGG---AAAAAAAAAACTCTAAGGAATCAAAAACAAAAT----

-----TGAAAAATTGGATCTATGTCCA---GCGGATCACGCCTACTAAGA------AAAA

GTATTTTGTTTTGGTCCGACCCGTAGCCACATATGAAATAGCAGATGGGATCAATTTAGC

AACGCTTTTCCCTCAGGATCTATTGCAGGAAAGGGATAATGTTCAACTTCGAGTTGTTAA

TTATATCCTTTATGGAAATGGCAAACCAATTCGAGGAATTTCTCACACAAGTATTCAATT

AGTTCGTACTTGCTTAGTATTGAATTGGGACCAAGAACAAAATGGTTCTAT---AGAAG-

--GTGTTCGTGCTTCCTTTGTTGAAGTAAGGGCAAACGATCTTATTAGAGATTTCATAAG

AATTGAGTT------ATGCAAGTCCGCTATTTTGTATACCGGAAAGAGGAAGGATATCGC

AGGTTCAGGATTGATCCACGATAATGTATCGGATCGCACAAATAT---CAATCCTGTTTA

TTTGAAGGATAAGATTCCATCATTTATTCAACATCAAGGGAC------TGTTGGTACGTT

GTTGAATCGAAATAAGGAATGCC------AATCTTTGATTCTTTTGTCATCATCTAATTG

TTTTCGAATTGGTCCATTTAATGGTTCAAAATATCACAATGTGCCAAAAGAATCAATT--

----AAAGAGGATCCTATAATTCCAATTA---------------------GAGATTCGCT

GGGTCTTTTAGGTACTACTGTACCAAAAATT------GCGAATTTTTTTTATTTATCTTA

CCATGTAATAACTCATAATCAT---------ATCTTGTTAACTAAATATTTGCTACG---

---TGACCATTTAAAACAGGCTT---------TCCAAGTAC------TTAGATATTGTTT

AATGGATGAAAATAGGAGAAT------TTATAATCCCGATCCGTGCAGTAACATCATTTT

TAATGCATTCGATTTGAATTGGCGCTTTCTCCATCACGA------TTATTCTGAAGAGAC

ATCCAC------AATACTTAGCCTTGGACAATTTGTTTGTGAAAATGTATGTCTATTTAA

ACACGGACC------ACAGAT---AAAATCGGGTCAAGTTATAATTGTTCATGTTGACTC

CTTTGTAATAAGAGCAGCCAAGCCGTATTTGGCCACTCCAGGAGCAACTGTTCATGGCCA

TTATGGAGAAATACTTTACGGAGGAGATACATTAATTACATTTATATATGAAAAATCAAG

ATCTGGTGATATAACGCAGGGTCTTCCAAAAGTAGAACAAGTGTTAGAAGTACGTTCAAT

TGATTCCATATCGACGAACCTCGAAAAAAGGGTTGAAGGTTGGAACGAACATATAACAAA

AATTCTTGGAATTCCCTGGGGATTCTTGATCGGCGCCGAGTTAACCATAGCGCAAAGTCG

TATCTCTTTGGTTAATAAGATCCAAAAAGTTTATCGATCTCAGGGGGTGCAGATTCATAA

TAAGCATATAGAGATTATTGTACGTCAAATAACATCAAAAGTGTTGGTTTCAGAAGATGG

AATGTCTAATGTTTTTTCACCGGGAGAACTTATCGGATTGTTGCGGGCGGAACGAACAGG

TCGCGCTTTGGAAGAAGCGATCTGTTATCGAGCAATCTTATTGGGAATAACAAGAGCGTC

TTTGAATACTCAAAGTTTCATATCCGAAGCGAGTTTTCAAGAAACTGCTCGAGTTTTGGC

AAAAGCGGCTCTACGGGGTCGTGTTGATTGGTTGAAAGGCCTGAAAGAGAACGTTGTTCT

AGGGGCGATGATACCCGTCGGTACGGGATTCAAAGCATTAG-TACACCGG---T-CAAG-

------AAAACCCAACAACATTCATTTGGAAATCAAAAAGAATAATCTATTTGA-GGGGC

AAGTGGGAGATGATATTTTGTTCTACCACAGAGAATTATTT-------G------GTTCT

TGCG-GC-----CCAAATAATTTCCATGAT---------------ACATCAGAACAGTCA

TTT---------------------------------ATGAAATTTCATGATTCCTAGATG

ACA---------------------------------------------AAAAGATATTGG

AACATCAATTTGGAAGAGATGATGGAA-GCAGG--------AGTGCATTTCGGTCACGGT

ACTAGGAAATGGAATCCTAGAATGGCACCTTACATTTCTGCAAAGCGTAAAGGTATTCAT

ATTACAAATCTTACTAGAACTGCTCGTTTTTTATCAGAAGCCTGTGATTTACTTTTTGAT

GCAGCAAGTGGGGGAAAACACTTCTTAATCGTTGGTACCAAAAAGAAAGCAGCTGATTCA

GTAGCATCGGCTGCAATAAGGGCTCGGTGTCATTATGTTAATAAAAAATGGCTTGGTGGT

ATGTCAACGAATTGGTCCACTACAGAAACGAGACTTCAAAATTTTAGGGACTTAAGAGCA

GAACAAAAGGCTGGGAAAATCGACC---GTCTCCCGAAAAGGGATGCAGCAATGTTGAAG

AGACAATTATCTACCTTGCAAACATATCTGGGTGGGATCAAATATATGACGGGTTTACCT

GATATTGTAATCATCGTTGATCAGCAAGAAGACTATACGGCTCTTCGAGAATGTGTCATT

TTAGGAATTCCGACTATTTGTTTAATCGATACAAATTCTGACCCGGATCTCGCAGACATT

TCGATTCCCGCCAACGATGACGCTATAGCTTCAATCCGATTGATTCTTAACAAATTAGTA

TTCGCAATTTGC----GAGGGTCGTTCTAGCTATATAAG------------AAATCGTTA

AATGGGACAAAAAATAAATCCACTTGGTTTCAGACTTGGTGCA--ACCCAAAGTCATCTT

TCCCTTTGGTTCGCACAACCAAAAAGTTATTCTATGGGTTTACAGGAAGATGAAAAAATA

CGGGAGTGTATCAAGAATTATGTACAAAAAAACCCGAGAC--------------------

-------------------------------------------------TGTCTTCAGGA

TTCGAA------GGAATAGCGCGTATAGAAATTAAAAAAAGAATCGATCTGATCCAGGTC

ATAATCTATATCGGATTCCCAAAT---TTATTCATAGAGGGTCGAACGCAAGGAATCAAA

GAATTACAGATGAGTGTACAAAAAAGATTAAATTCTGTGAATCAGAGACTCAACATTGCT

ATCGCAAGAATTTCAAAACCTTATGGGCAACCCAATATTCTTGCTGAATATATAGCTTTA

CAACTAAAAAATAGAGTTTCGTTTCGAAAAGCAATGAAAAAAGCTATTGAATTGACTGAA

CAAGCAGATACAAAAGGAATTCAAATTCAAATCGCAGGACGTATCGATGGAAAAGAAATT

GCACGTGTCGAATGGATAAGAGAAGGTAGGGTTCCCCTACAAACCATTCGAGCTAAAATT

GATCATTGTTTCCATAAAGTTCAAACTATCTATGGGGTATTAGGCATAAAAATTTGGATA

TTTGTAGACGAG---GAATAA------ATGTCTCGCTACCGAGGGCCTCGTTTAAAAAAA

ATACGCCGTCTGGGGGCTTTACCCGGACTAACTAGTAAAGGAACTAGACCCGGAAGTGAT

CTTAGAAATCAATTTC----GTTCTG--GTAAAAGATCCCAATATCGTATTCGTCTTGAA

GAAAAACAGAAATTGCGTTTTCATTATGGTCTGACAGAGCGACAATTACTTAGATATGTT

CATATCGCCGGAAAAGCCAAAGGATCAACAGGTCAGGTTTTACTACAACTACTTGAAATG

CGTTTGGATAACATCCTTTTTCGATTGGGTATGGCTCCTACGATTCCTGGAGCCCGGCAA

TTAGTTAA--CCAT-AGACATATTTTAG--TTAATGGTC--GTATAGT-AGATATACCAA

GTTATCGTTGCAAACCCA--GAGACATTA-TTACTAC--GAAGGATAAGC----AAAGAT

CGAAAGTTCTGATTCAAAATAATATGGATTCATCGACCCGCGAGGAATTGCCAAAACATT

TGACTCT---TGACTCATTCCAACATAAAGGATTAGTAAATCAAATCATAGATAG-TAAA

TGGGTCGGTTTGAAAATTAATGAGTTGTTAGTTGTGGAATATTATTCCCGCCAGACTTAA

ATGTCACGTCGAGGTACTGCAGA------AGAAAAAACTGCAAAATCCGATCCAATTTAT

CGTAATCGATTAGTTAACATGTTGGTTAACCGTATTCTGAAACACGGAAAAAAATCATTG

GCTTATCAAATTATCTATCGAGCCTTGAAAAAGATTCAACAAAAGACAGAAACAAATCCA

CTATCTGTTTTACGTCAAGCAATACGTGGAGTAACTCCCGATATAGCAGTAAAATCAAGA

CG---TGTAGGCGGATCGACTCATCAAGTTCCTATTGAAATAGGATCTACACAAGGAAAA

GCACTTGCCATTCGTTGGTTATTAGCGGCATCCCGAAAACGT---CCGGGTCGAAATATG

GCTTTCAAATTAAGTTCCGAATTAGTAGATGCTGCCAAAGGGAGTGGCGATGCCATACGC

AAAAAGGAAGAGACTCATAAAATGGCAGAGGCAAATAGAGCTTTTGCACATTTTCGTTAA

ATGGGTAAGGACACTATTGCCGATATAATAACTTCTATAAGAAATGCTCAAATGGCTAAA

AAAGGGACAGTTCGAATAGCATCTACGAATCTTACCGAAAACGTTGTTAAAATACTTCTG

CGAGAAGGTTTTATTGAAAACGTTAGAAAACATCGGGAAAGTAATAAAGATTTCTTGGTT

TTAACCCTGCGACAT------AGAAGGACTAGGAAAGG----------------------

------------------------------------------------------------

--------------------------------------GATATAT---------------

------------------------------------------------------------

------------------------------------------------------------

------------------------------------------------------------

------------AGAACTA------TTTTAAAGCGTATCAGCCGACCTGGTTTACGAATC

TATTCCAACTATCAAGGAATTCCCAAGATTTTAGGCGGAATAGGGATTGTAATTGTTTCT

ACTTCTAGAGGTATAATGACAGATCGGGAGGCTCGACTACAGGGAATTGGAGGAGAAATG

TTATGTTATATATGTTGAATGACAAAAACTATACCAAGAATTGGTTCACGTAGGAATGGA

CGTATTGGTTTACGT------------------------AAGACTGGACGTAGA------

------------------ATACCAAAAGGAATTATTCATGTTCAGGCAAGTTTCAACAAT

ACCATTGTGACTGTTACAGATGTTCGGGGTCGAGTAGTTTCTTGGTCCTCCGCCGGTACT

TGTGGATTCAAAGGCACAAGAAGAGGGACGCCGTTTGCGGCTCAAACCGCAGCGGGAAAT

GCTATTCGTACAGTAGTAGATCAGGGTATGCAACGAGCAGAAGTCATGATAAAAGGTCCT

GGGCTCGGAAGAGACGCAGCATTACGAGCCATTCGTAGAAGCGGCATATTATTAAATTTC

GTACGTGACGTGACCCCTATGCCACATAATGGATGTAGACCTCCTAAAAAAAGACGTGTG

TAGATGCCAACTATTAAACAACTTATTAGAAACACAAGACAGCCAATCCGAAATGTCACA

AAATCCCCCGCTCTTCGAGGATGTCCTCAGCGTCGAGGAACATGTACTAGGGTGTAT-AC

TATCACCCCCAAAAAACCAAACTCTGCCTTACGTAAAGTTGCCAGAGTACGATTAACCTC

TGGATTTGAAATCACTGCTTATATACCTGGTATTGGCCATAATTTACAAGAACATTCTGT

AGTCTTAGTAAGAGGTGGGAGGGTTAAGGATTTACCCGGTGTGAGATATCACATTGTTCG

AGGAACCCTAGATGCTGTCGGAGTAAAGGATCGTCAACAAGGGCGTTCTAGTGCGTTGTA

GATT----CTTATCCAAGACTTGTATCATT---TGATGATGCCATGTGAATCGCTAGAAA

CATGTGAAGTGTATGGCTAACCCAATAACGAAAGTTTCGTAAGGGGACTGGAGCAGGCTA

CCATGAGACAAAA-GATCTTCTTT-CTAAAGAGATTCGATTCGGAACTATT------ATA

TGTCCAAGGTCCAATATTGAAATCATTTCAGAGGTTTTCCCTTACTTTGTCCGTGTCAAC

AAACAATTCGAAATACCTCGACTTTTTTAGAACAGGTCCGAGTCAAATAGCAATGATTCG

AAGCACTTCTTT-----TTACACTCTTTCGGAAACCCAAGGACTCGATCGTATGGATATG

TAAAATACAGGATTTCCAATCCTAGCAGGA-AAAGGAGGGAAACGGATACTCAATTT---

---AAAGTGAGTAAACAGAATTCCATAC--------TCGATCTCAT-----AGATACATA

TAGAATTCTGCGGAAAGCCGTATTCGATGAAAGTCGTATGTACGGCTTGGAGGGAGATCT

TTCATATCTTTCGAGATCCACCCTACAATATGGGGTCAAAAAGCCAAAATAA--------

---------ATGGCAAGGAAAGGTTTGATTGAGAGGGAGAAGAAGAGGAAGAAATTGGAA

CAGAAATATCATTCGATTCGTGGATCCTCAAAAAAAGAAAT---AAGAAAAGTTCCATCG

TTGAGT------GACAAATGGGAAATTCATGGAAAATTGCAATCCCCACCACGTAATAGT

GCACCTGTACGTCTTCATCGACGTTGTTTTTTGACCGGAAGACCCAGAGCTAACTATAGG

GATTTTGGGTTATCCGGACACGTACTTCGAGAAATGGTTCAGGCATGTTTGTTGCCGGGC

GCAACAAGATCAAGTTGGTAA------------------------------------ATG

GT---CAAAAATTCATTTATCT-CGCTTATTCCG------CAAGAAGAAAAAGAAGAA--

----AACCGGGGATCTGTTGAATTTCAAGTATTCAGTTTCACCAATAAGATACGGAGACT

TACTTCACACTTGGAATTACACAGAAAAGATTATTTATCCCAGAGAGGTTTACGAAAAAT

TCTAGGAAAACGTCAACGGCTGCTGGCTTATTTGTCAAAGAAAAATAGAGTACGTTATAA

TAAATTAATTGGTCAGTTGGATATTCGGGAGCCAAAAACTCGTTAAATGGTAAAACTTCG

TTTAAAACGATGCGGTAGAAAGCAACGTGCGACTTGAAGGACATGATCGGC--TGTGGAA

TATTACATCCACCATTTTCTATAGGAATGAAGATGCTCTTGGCTCGACATAGTTTGTTCT

GTTCCATTGGGA----CCCAAATTTTGTTGGGTTG-----------------TAA--AT-

----------------------------------------AGTACATGATGGAGCTCGAG

-CAGAA-AG-----GATTGA----TTTATT-TTTC-AGGGGGAAGAATCTAGGGTTAGCA

CC------AATCAATAAGTTGGAACAGCTTTGTAA---GTATATCTATACTA--------

-------------------AATAGAGAAATCGAAGGGTT----------CAAATTCGAAC

CCAACAAGTTTGAAATAAAAAAA---------AAAAATTGTCGGAAT-TGGTAAAACTAT

TTCGATCAA-----AAGTGTATCAAAGTGGAATT----------TATTGTTCGTATG---

----ATTTTTCCATA-----------------------------TAAAGAAATAAAAAGG

GTATGTTGCTGCCATTTTGA------AACAATTAAGGATCACCGAAGTAATGTCTAAACC

CAATGATTC----------AAAGCAAAGATAAAGGATCCCGGAACAAGAAAACACAATTT

TCAA---TTGT------CTTAA----------------------CAATTGGATCCGAATG

AGAAAT--------------CAAA----ATGGATTCGAGACGAGACAAAG-AAAAGAGGT

TAGAGACGGCTCAAGAAA-------TG--------TCTAAAGAGTTCTTT----------

---TCAG----AATTACCCAACTTG-AGTTATGAGTACGAATGATATTTT-------TTT

TTTTGAAGGAAGAAGGTAAAAACGACTCAAATTTCAAATTAGAGTCTAATTACCGAT---

-------------TTTATGAATCCATTTGACA-----CTATATACAG-------------

------------------------------------------------------------

------------------AAGAGAAATTAGAA----------------------------

-------------------TCATTCTTTCTCGAGCCGTATGAGGAGAAAACCTCATATAC

GTTTCTA-----GGGGGGGTATTGTTTATATATATCTATCCCAATGAGCCATTTATCGAA

---TCGTTGCAATTGATGTTCGATCCCGAAGAGAGGGAAGAGATCTTTGTAAAGTGGGTT

TTTATGATCCGATAAAAAATCAAACTTATTTAAATGTTCCTGTTATTCTAAATTTTCTGG

AAAAGGGGGCTCAACCTACAGAAACTGTTCATGATATTTTAAAGAAGGCAGAGGTATTT-

---------AAGGAACTTCAAGG------------------------AAATCAAACGAAA

TGAATGGATAAA------------------------------------------------

---------TCCAAGCAAC-----------------------------------------

------------------------------------------------------------

-------------------TTTTTCGTAAATCCAAGCGATC-------------------

--TTTTCGTAGGCGTTTGCC------------------------CCCAATTGGATCGGGG

GATCGAATTGATTATAGAAACATGAGTTTAATTAGTCGATTTATTAGTGAACAGGGAAAA

ATATTATCTAGACGAGTGAATAGATTGACCTTGAAACAACAACGATTAATTACTATTGCT

ATAAAACAAGCCCGTATTTTATCTTCGTTACCTTTT-----------CTTAAT------A

ACGAGAAACAATTTGAAAGAACCGAGTCAATACCTAGA-CCTACCGGTCCTAGAAGCAGA

AATAAATAG---------------------------------------------------

----------------------------------------------------GTGACACG

TTC------------------------------------------------------ATT

AAAAAAAAATCCTTTTGTGGCCAATCATTTATTGGAAAAAATTGAGAAGCTCAACATG--

----------------------AAAGAGGAGAAAGAAATAATAGTAACTTGGTCCCGGGC

ATCTACCATTATACCCACAATGATCGGTCATACAATTGCTATTCATAATGGAAAGGAACA

CTTACCCATTTATATAACAGATCGTATGGTCGGTCACAAATTGGGAGAATTCGCACCTAC

TCTTACTTTCCGGGG------ACACGCGAGAAA---------------------------

------------------------------------CGATAATAAATCTCGTCGTTAAAT

GATT-------------------------------TTCAAATCATT---TCTACTAGGTA

A---------TCTTGGATCCTTATACA--------------TAAAGATAATAAATTCAGT

CGTTGTGGTCGGACTATATTATGGATTTTTGACA------ACATTCTCCATAGGGCCC--

--TCTTATT--------TCTTCCTTCTCCGAGCTCGGATTATGGAAGAAGGAACCGAGAG

GGAGGTATCAGCAACAACTGGTTTTATTACGGGACAGCTCATGATGTTCATATCGATCTA

TTATGCGCCTCTGCATCTAGCATTGGGTAGACCTCATACAATAACTGTCCTAGTTATACC

GTATCTTTT-------GTTTCAT------TTCTTCTCGAACAATCAAAAACAATTTTTTG

--ATTATGGAT---------CTAC---TACCAGAAATTCAATG---------CGTAATCT

CAGCA--------------------TTCAATGTGTA------------------------

------TTCCTAACTAATCTAATTTTTCAACTCTTCAACCATTTAATGTTACCAAGTTCA

ACGTTAGCGAGATTAGTCAACATTTATATGTTTCGATGCAACAACAAGATGTTATTTGTA

ACAAGTAGTTTTGTTGGTTGGTTAATTGGTCACATTTTATTCATGAAATGGGTTGGA---

------------TTGGTAGTATCCTGGATACGGCAAAATCATTCTATTCGATCTAATGTA

CTTATTCGATCTAATAAGTACCTTGGGTTAAAATTGAAAAGTGCTATAGCTCA-------

--AATCTTGAGTATT-ATCTTCTTTATCGCCTGTGTCAA--CTATTTAGCCAGACTACCG

GATCCTAGTGTCACT---------------------------------------------

------------------------AAGAAACTG------AACGAAACCTCAAAAACGGAA

ACGGAAGAAGAAGAAAGTAAAGAAAGTCAAAAAAG-------------------------

---------------------------------------TAAAGAAAGTGAGGA------

---------------------------------AGAAAGAGACG------TAGAAAAAGA

AACAA------------------------------CTTCCGAAACG------AAGGAGAC

T--------------AAACAG---GAACAAGAGGGATC--CACCG---------------

-AGCAAGACCCTTCCC---CTTATT------GGGAAGAAAA------------GG-----

--AGGATCCGGAC---AAA---------------ATAGATGAAACGGAAGAGATCCGAGT

GAATGGAAAGGAAAAAAAAAAGGATGAAACGGAAG--AGATCCG---------AGTGAAT

---------GGAAA----------GGAAAAAAAAAA------------------------

-----------------------------------------GGATGAATTCCGCTTT---

---------------CACTTTAAAGAGAC--------------AGACTAT-----AAACG

TAGCCCAGG------TTAC---------GAAAACTCT------TATCTTGATGGGTATCA

AGATAA------TT--------GGGACTTGCAA------AG-------------------

TGAA--------GAAGAAGAAGAAGAAGAAA------GTACTTTAGGGT-----------

TTGAAAAACCTCTTGT---------------------AACTTGTCTTTTCGA--------

-----------------CTATAAACGAT--GGAATCGACCTTTTAGATATATAAAAAA--

--------------------------CAAAACA----------------------TTCGA

------GAA---TGCTATAAGAGACGAAATGTCACAA-----------------------

---------------------------------TATTTTTTTTATACATGTCTGAA----

---TAATGGGAAAAAAA------------------AAATATCTTTTAC------------

--------------ATATCCACCTAGTTTAGCGACTTTTTCG---------GAAATAAT-

-AGAAAGA----------------------------AAGATGTCTTTG------------

TACACAACAAAAAAACTAACTG-----ATGACG----------------AC------TTG

TATAG------TCATTGGG----------------TTTCCAAC--------AATGAAGAG

AAAAAGAACAACTTA-AGTAATGAGTTGATAAACCGAA------------TAAAAGTTTT

AG---------------ACAAGGGATCCCTTGCTCCGGATGTGCTCGAAAAAAGG-----

---------ACGAGATTGTGT----------------GATGATGAGAATGAACAA--GAA

TGCTTACCTAAAAAGTATGATCC--TTTGTTGAATGGATCATATCGCGGAAGAATAAAGA

AATTAGAATC------------------------------AGAATCAACTAG-------G

AACGA---------TTCAATCATTTC---------------C------------GCAAA-

--------------------------AGGTTCACTAGAAAAAATTTGGAAAAATAAGATA

CACAGTCTTAT--------------------TACTAATGATTCTCG------------AG

AATTTGA-------------------------------ACATCAAATGGATCCGTT----

--TGACGGAGAATCTTTAT------------------------------CGGCATATATG

ACTCATTCCTTAACC------TCAATCAGTAAATTGTCTTTGGAATTAGTATCTATTTTC

CATTTTGAGGATCTT---------GCTTTATTGACAGAACAAAAAAGAA-----------

----TTGATTTTGAAA-------ATCAAACGAAACGGTTG----------------AAAT

TTCTATTCGA--------TGTAATTAC-----------AGCTGACGCAAATAATCAAA--

C---CATTGAAAAT-----------AAATTTAT------TGAAA-------T------A-

-----GAAAAGATCGACAAAAA-------GATTCCTAGA---------TGGGTATA----

-----------------CAAATTGATATCCGAG---GA--GGATTATTTGGCACAA---C

AAGA--------------------------------ACAACAGGA--AGAAGAAAA----

------------------------------------------------------------

------------TG---AGGA-------AGAAGCACCACTGG---ATTTTGGAATTC---

-------------------------GTTCGAGAA------------------AAGGCAGA

CG------------------TGTAGTAATTTAT-------------------ACTGATAA

AAATCAGAATCGGGATCAGGATCC-------------------CAATCC------TAAT-

------------------------------------------------------------

------------------------------------------------------------

-----------------------------------ACCGATAATACTACTACCGAGAACG

ATAAT-------------------------------------------------------

-----AGTGATACG---------------GGGGAA------GAAATAGCTTTGATACGTT

ACGCGCAACAATCAGATTTTAGTCGGGATCTGATCAATGGATCCATACGCGCTAAAAGAC

GTAAAATAGTTC---TTTGGGAAA-----------TGTTAC---AAGCAAATGCGCATTC

CCC------ACTTTTTTTAGAT----------CAAGTAGCAAAAATGGTTTCTTTT---G

ATCT---------CTTTGATGATCT---------TCGTGAAACAATGAATCT------TA

TTTTTAGGAATTGGATAACAAAGGAGC------CAGAATT---------ACAAATTTTG-

-----------GATTCTGAAGAGAAAGA-------------------GGACAGAGAAAAA

CTGA---------TAAA-------------GATAAGGGAGGAAG----------------

-----------------ATGA------ACGG---ATAATAATATCCGAAACTTGGGATAA

CGTTATGTGTGCCCAAGCAA-------------TAAGAGGCTGTATGCTAGTAACCCACT

CATTTATTAGAAAATACATTATATTGCCTTTATTAATAATAGCTAAAAACATTGGGCGTA

TATTATTATTTCAGCTCTCTGAGTGGGATGAGGATTTTAAGGACTGGAATAGAGAAATGC

ATGTTAAATGCACCTATAATGGTGTTCAATTATCAGAAACAGAATTTCCTCAAAACTGGT

TAAAGGATGGTATTCAGATAAAGATCCTATTTCCTTTTTCTCTAAAACCTTGGCGCGAAT

------CTA--AGGCAACACCC------TCTACTGGAGGTCTAATGAAAGAGAAAAAAAG

AAAAAATGACGATTTTT--------GTTTTTTAACAGTTTGGGGAATGGAAGCGGAACTC

CCTTTTGGT---CCCCCTCGAAACC----GACCTTCT-----TTTTTTAAACCCATTTTT

GAAGAACTTGA------------CACAAACA------------TTCGAAAGGTGGAAAAC

CAAA------GTTTT---GTTTTCAAAG---------AAAA---------------AACA

AAAGACT---------TTTTAAAGAAAAAAAC---------G---GGATGGGTTA-----

--------------------------------------CAAAAATCGTTTTAT-----TG

AAAAATA------AAATAAGGAACTTTTTT------------------------------

---------------------ACAAAAGTAAACCCCAATTTATTATTCGGATTGAA----

-----GAAAGTATAT---------GAACCAAGTGAGAATCAA-----AAAGATTCCATAA

T------------TAGTAATAAGA---------------TTACCC------AC-----GA

ATCGACTGTTCAAATTCCATCCAGCAATTGGACAAATTATTCACCGATAG----------

------------------AAAAAAAAA--------TGAAGGATCTGTCTGATAGGACAAT

CACAACCAGGAGT---------------------CAAA------TAGAA------CGCAT

CAC----AAA--------------AGACAAGCAAA------AAGGATTTCTAACTTCA--

-------GATATAAATATTCGTTCTAATGAGACAA---------GTTGTAATGCTAAAA-

-----GAACGGAATT-------GCAGAAA---------GATATTTTACGGAT--------

-AGCA---AAAA------------------------------------------------

----------------------------AAAGAAG---------------TATCCGATTC

ATCCGTAAA---TTACACTC-----------------TTTTGTGAAA-------------

---------TCTTTCATTGAAAGAATATACCTAGATA-----------------------

-------------------------------------TCTTTCTATGTACCATTAACA--

TTCCCCGAATTAATCTA------CAACT---------------TTTCTTTGAATCAATAA

AAAAG-------------------ATTCTCAATAAAT---CCATTTCT-----------A

ATGATGAAAGAAAT---------------AAAGA--------------------------

-------------------------AAAAATTGATGAAACAAATCAAAATAC--------

------AA------TTCACTTTATTTCTACT--------ATAAGAAATTCG---------

------------TTTTCTAATA---GTAATCTTAA------------------------T

AATAAATCAAAGATTT---------------------------ATTGGGATTTCTCTTTA

TTGTCCCAAGCATATGTATTATATAAATTATCACAAACCCAAGTGATTAAAAGGTATCAA

TTGAAATCTGTACTTCAATAT------CACAGAGCATATCCTTTTCTGAAGGATAGAATC

AAG------------GACCTTTTTGGGACACCAAGAATAGTTCATGCCAAATCAAGGCCT

AAGAAACTTCCTATTTCGAGAATA-AATGCATGGAAAAACTGGTTAAGGGGTCATTATCA

ATACAATTTATC-TCAGA--CTAGATGGTCTGAATTAGTACCGAAAAAATGGCGAGAGAA

ATTCAATCAAC---GTACGA------TTAAAA------ATAAATACTCAAGAAAATTGAA

TTCATCTGAAAAAGAAAAAAACCAACAAGCTCATTACGCG----AAAGAAAT------TT

CTTATGT------AGTGGATTCATTGT---------------------------CGAGTC

AAAAAGGAAAATT----GAAAAAAGACTACAGATATGATCTTCTATCACATAAATATATT

AATT---------CCAATTATGAGGATAG---------------------GGAGTACTCA

GATATTTCGAGATTAT----------------------CATTACAAATAAATGGG-----

------------------------------------------------------------

------GATCGAGAAATTCCATA------TGATTAC-AAT---------AGACAAGAATC

CGA------TT---ATGTACTAGTTGGTTTACCTATTAGTGATTATCTAGGAGAAGAATA

TTTTATTGGCGTAGATAAAA------ATTCGGA-----TAGAAA---ATATTT-TGATC-

GGAGGATTCTCCGTTTTG-A-----------CCTTAGAAAGGATCTTA------ATATTA

AGACCCAGATCAATAGGGATACAGAGACC---------------AACATGAATAAAAA--

-------CGGAACTAATAA--TTATCCAGCAATTG-GTAAGAAA-----AATCCTTTTTC

TC------TCCCGATTCATCAAGAAATCAATT------CATCCAAGAAAAAAAAACAAAA

ATT------TTTTGATTGGATG---GGAATGCGCGAAGAAATTTT---------ATATCG

TCCGATAT-----------------CGAATCTGGAGCCTTGGTTT-------------TT

CCCAGAATTTGTGCTACG------------------TTACGATGCATATAAGAGTAAACC

ACGGATCATATCAATCAAATCTCTTCTTTTAGATTCTCAGAAAGACGAGCGAAATGAAA-

-----------------------------TAATTAGTAAAACAAAAAACA----------

------TCAACAAAAAAAA-------------------TCAAAAAAAAGAT---------

---CTATCTAATCAA------AA--AAAACATCTTGAAGTAGAGAA---------TCAAG

AAAAAGAGGATTTTCGACAAGTAGATCTTAGACCCAATCCAACAA-------ATCAAA--

--GGGATCCTAT--------------------TGTATCAGAT------------ACACGA

AACC-------------------AACAAAAAGATATTG----------------------

-----------------AAGAGGA---------TTCCGTGGGATCAGGCGTTAAAAAACG

TAGAAAAAAAAAGAAATT---------------CAAGAG-TAAGAAGGAAGCAGAATTAG

ACTTTTTTCTAAAA-----AAATAT----------CTCCT--------TTTTC-------

---AATTGAGATG-------------GG---------ATGAGCTTTTGAAT---------

-AAAAAAA----------TGATGAC-----------------------------------

-TAATATTAAGGTATATTGTCTCCTGCTTAG-------ACTGAAA--AATCT-------A

AAGGAAATCGCAA-------------------------------TATC----CTCCATTG

AAAGA-----------------GGAGAGATGTGCCTGGATTTAATGCT------AAT---

---GCAAAAAGATCTATCTCTTAGGG----------------------------------

AATTGATA--------AAAAAAGGAAT------ATTC-----------------------

------ATTAT------CGAACCGATTCGTTTATCTAGAAAATGGGA-------------

-------------------------------------------------CGGAAAATTGA

TTATGTATCAAACCATAGGTATTTCATTG-------GTCGCTGAGA--------------

-ATAAGGATC------CAATTAATATAAGATGTCGAAAAAAAGGAT--------------

-----ATGCTGAT---GAGAATTCTTTCAATAAATCCGTTAGACAACAAGAAAAAATGCT

TGT---AGAT-AGAGACGAA---------------------------------AATGATT

ATGATTTGC------------TTGTTCCTGAAAATATTCTATCTCCTAGACGTCGTAGAG

AATTGAGAATCCTAATTTGTTTTAATTCCGGGAATG---AGAGTGCT------GTGGATG

GAAATTCAGTATTTTACAATAACAAGAATGTGG------AGAGCTGTAGAC-----AATT

TTTGGATGA-----------GGACAAGCATCT------TGATACAGATGC------AAAG

------AAATTTATGAAATTG------------------AAATTTTTTCTTTGGCCCAAT

TATCGATTAGAAGATTTAGCTTGTATGAATCGCTATTGGTTTGATACCACTAATGGCAGT

CGTTTCAGTATGTTAAGGATTCATATGTATCC-ACGATTCCTAATCAGTTGGTGGTAG--

-------------------------------------------------ATGAAACGACA

TCAAT------------------------TCAG---------AT----------------

------------------------------------------------------------

-TCTGGATTTTCGAATTGAGAGAGATATTGAGAGAGATCAAGAATTCGCCCCATTTCTTA

GATTCATGGACCAAATTCGATTCAGTGAGATCTTTGATTCACATTTTTTTCCACCAAGAA

CGTTTGATGAAACTCTTTGACCCCCGAATTTGGAGTATCCTACTGTCACGCGATTCAC--

----AGGGTTCAACAAGCAATCGATATTTCATTATCAAAGGTTTAGTACTTCTTGTAGTA

GCGGTCCTTATATCTCGTATTAACAATCGA---AATATGGTCGAAAGAAAAAATCTCTAT

TTGAGGGGGC---------------TTCTTCC---TATACCTATGAATTCCATTGGACCC

GGAAAT------------GAGACATTGGAAGAATCCTTTTGGTCTTCCGATATCAATAGG

TTGATTGTTTTGC------TCCTGTATCTTCCAAAAGGGAAAAAGACCTCTGAGAGTTGT

TTCATGGATCCGAAAGAGAGTCTTTGGCTTTTCCCAATAAATAAAATAAATCAAAAGTGT

ATCATGCCCGAATCTAACC------------GGGGTTCGCGGTGGTGGAGGAACCGGATC

GGAAAAAAGAGGGATTCTAGTTGTAAGATATCTACTAAAACCGTAGCTGGAATTGAGATC

TCATCCAAAGAGAAAGATCTAAAATATCTGGAGTTTCTTTTTTTATCCT------ATACG

GATGATCCGATCCGCAAG------------------------------------------

------------------------------------------------------------

------------------------------------------------------------

------------------------------------------------------------

------------------------------------------------------------

------------------------------------------------------------

------------------------------------------------------------

------------------------------------------------------------

------------------------------------------------------------

------GATCGGTTTTTTAGCAAGGTACAGAATGTATCGTCAAATATTCAATATGATTCC

ACAAGATCTATTTTCGTTCAAGTAACGGATTTTAGCCAATCGAAAGGA---TCTTCTGAT

CAATCCAGAGATCACTTCGATTCCATTAGTAAGGAGGATTCAGAATATCACACATTGATC

GATCAAACAG--------------------------------------------------

------------------------------------------------------------

------------------------------------------------------------

-------AGATTCCGCAAC---------TAAAAGGAAGATCGATTCCTGGGGATC---CT

TCCTTTCTTCAAACGGAACGAAC------AGAGATAGAATCAGATCGATTCCCGAAATGC

CTTTCTGGATCTTCCCCAATGTCCCCG---------CTATTCCCCGAACATGAGAAGCAG

ATGATTATTCATCGGCTTCCG------GAAGAAATCGACGAATTTCTTGGGAATCCTACA

AGATCAATTCGTTCTTTTTTCTCTGACAGATGGTCA------GAACTTCATATG---GGT

TCGAATCCTACTGACAGGTCCACTAGAGATCAGAA------------ATGGTTGAAGAAA

CAACAAGATG---TTTCTTTTGTCTCTTCCCGGCGATCAGA------------AAATAAA

GAAATGGTTGATATATTCAAGATAATTACTTATT------TACAAAATACCGTCTCAATT

CATCCT-----ATTTCATCAGATCCGGGATGTGATATG---GTTCCGAAGGATGAACCAG

AT---ATGGACAGTTCCAATAAGATTTCATTCTTGAACAAAAATCCATTTTTTGATTTAT

TTCATCTATTCCACGATCGGAACAAGGGGGGGTACACATTACACCACGA---TTTTGAAT

CAGAAGAGAGATTTCAAGAAATGGCAGATCTATTCACTCTATCAATAACCGAGCCGGATC

TGGTGTATCATAGGGGATTTGCCTTTTCGATTGAT---------------TCCTGCGGAT

TAGATCAAAAAAAATTAGTGA------ATGGAAAGGAAAAAACAAAGGATGAATCGAAAA

AGAAATCTTTATTGGTTCTACCTCCTCTTTTTTATGAAGAGAATGCCTTTTTTTATCGAA

GCATCAG-AAAAAAACCGGTCTGGATCCACTGCGGGAATGATTTG---------------

------------TTTGCTAGCAACAACATAATGGAGGCAGTCAATCAATATAGATTGATC

CGAAATCTGATTCAAATCCAATATAGCGCCTATGGGTACATAAGAAATGTATTGAATCGA

TTCTTTTTAATGAATAGATCCGATCGCAACTTCGAATATGGAATTCAAAGGGAT---CAA

ATAGGAAATGAT----ACTCTGAATCATATAACTATAATGAAATATACGATCAACCAACA

TTTATCGAATTTGAAAAAGAGTCAGAAGAAATGGTTTGATCCTCTT----ATTTCTCGAA

CCGAGAGATCCATGAATCGGGATCCTAATGCATATAGATACAAATGGTCCAACGGGAGTA

AGAATTTCCAGGAACATTTGGAACATTTCGTTTCTGAACAGAAGAACCGTTTT-------

--CAAGTAGTGTTTGATC------AATTACG-----------------TATTAATCAATA

TTCGATTAATT-GGTCCGAGGCTATCGACAAACAAGATTTGTCTAAGTCACTTCGTTTCT

TT--TTGTCCAAGTCA--CTT------------------------CTCTTTTTGTC----

---------------CAAGCCACTTCGTTTCTTTTTGTCCAAGTCACTTCCTTTGTTCTT

TGTGAGTATCGGGAATAGCCCCATTCATAGGTCCGAGATCCACATCTATGAATTGAAAGG

TCCGAATGAGCCG---CTCT---------------------GCAATCAGTTGTTAAAATC

AATAGGTGTTCAAATCATTCATTTGCATAAATTGAAACC------------------TGA

TCATGATA--CTTCCCAAAGATCGAAATTCTTGATCAATGGAGGAACAATATCACCATTT

TTGTTCAAT-AAGATACCAA------AGTG-GATGATTGACTCATTCCATACTAGAAAGA

ATCGCAGCAAATCCTTTGCTAACACGGATTCCTATTTCTCAATGATATCCCACGATCGAG

ACAATTGGCTGAATCCC-GTGAAACCATTTCATAGAAGTTCATTGATATCGTCTTTTTAT

AAAGCAAATCAACTTCGATTCTTGAATAATCCACATCACTTCTGGTTCTATTGTAACAAA

AGATTCCCCTTTTATGT---GGAAAAGACCCGTATCAATAATTATGATCGTACATATGGA

CAATTCCTCAATATCTTGTTCATTCGCA-ACAAAATATTTTCTTTGTGCGTC------GG

TAAAAAAAAACA---------------------TCTTTTA------CCGATTTATTCACA

GGTATCTGACATATTCATACCTAACGATTTTC------CACAAAGTGGTGACGAAACG--

----------TATAACTT---------GTACAAATCTTTCCGTTTTCCAATTCGACCCGA

TCCATTCGTTCGT------AGAGCTCTTTACTCGATCGCAGACATTTCTGGAACACCTCT

AACAGAGGAACAAATAGTCAATTTTGAAAGAACTTATTGTCAGCCTCTTTCAGATATGAA

TCTATCCGATTCAGAAGGGAAGAACTTGCATCAGTATCTCA------GTTTCAATTCAAA

CATTGGTTTGATTCACACTCCATGTTCTGAGAAAAATTTACCATACGTAA---------A

----------------GAGGAAA-------------------------------------

--AAACAGAGTCTTT----ATCTGAGGAAAAAACGGAGTC--------------------

------TTTATCTAAAGA------------------------------------------

--------AATGCGTTGAGAAAGGGCAGATGTATAGAACCTCTCAACAACGGGATAGTGC

TTTT---------TCAAAATGGAATCTGTTCCAAACATATATGCCATGGTTCCTTACTTC

GACAGGGTGCAAATATCTAACTTCCGTCCTTTTAGATATTTTTTCAGACCGATTGTCGAT

ACTAAGTAGTAGTCCAAAATTGGTATCCATTTTGAATTCTATTATGCA------------

TAGATCAGATATATCACGGCTAATT------AAAAAATGGTGGACGATTCTTCCACAAT-

----------GGAATCTGATAAGTGA----------------------------GATTTC

GAGTAAGTG------TTTACAGAA---TCTT--------CTTCTGTCCG-AAGAAATGAT

TCATCGAAATAATGAGTCACCCGTTTCAATGATATTGACACATCTGAGATCAACAA--AT

GCTCGGGAGTTCCTCTATTCAATCCTTTTCCTTCTTCTTGTTGCTGGATATCTCGTTCGT

ACGCATCTTCTCTTTGTTTCCCGAGTCTCTAATGAGTTACAGACAGAGTTAGAAAAGATC

AA----ATCTTTGAT---------GATTCCATCATAC-ATGATTG---------------

---------------------------AGTTG----------------------------

-------------------CAAAAACTTCTGTATAGGTATCCTACATCTACATCTAAATC

TGAATCTGAATCTGAATCTGAATATGAATATGAACTGAAT--------------------

----TCTTT--CTTGTTAAATAATCTCTTT------GCTCTGG-----------------

--------AACAATTAG-------------------------------------------

------------------------------------------------------------

--------------------------------------------ATTGGAAT--------

----------ATCGATCTCATAAGTATCATATCAAATACCA---------TCACTTTTT-

-------------CGAGAAATACGAGACATCTAAGTCGTA--------------------

------------------------------------------------------------

CAAG------------------------------TAAAGAGATCTA------TTCATTGA

TAAGAGAAAGAAAAAA--CGTGAACAGTGATTGGATTGAAGACAGAGT------------

-------------------------AGAATCCTGGGTTACGATCAATGATTTGA------

---------------TTGAT----------------------------------------

-----GAGGACGAAAGAGACTTGGCGGTTCAGTTCTCCACCTTAACGACCGAAAAAAGGA

TTGATCAAATTCTATGGAGTCTGACTCATAGTCATCCTGTATCAAAGAATGATCTCGATT

ATCAAATAATTGAACAACCGGGATTAATTTCCTTACGATACTTAGTTGACATTCATAAAA

AGGATCTAATGAATTATGAGTTCAATGGATCCTGTTTAGCAGAAAAACGGATATTCCTTG

CTAATTATCGGACAATCACTTATTC------ACAAACCCCAT------------------

------------CTCGTGGAAAACCCTTTTCACTCCGCTTAGACCTATCCCCCTCAAGGG

GTATTTTATTGATAGGTTCTATAGGAACTGGACGATCACATTTGGTCAAATACCTAGCGA

CAAACTCCTATGTTCCTTTCATTACGGTATTTCCGGACAGGGGCCTGTCTGACAGGCCTA

TATATTGTTTTATGGATGAG---------------GATACCGATCCTGATGAGGATTATG

------------------------ACTATATTGATGATGATATCGATATCGATAGTGATA

ACTTTGATAC------GGAGCTGGAAACTATCGCGGATGTGGTAACTATGGATAATATGC

TGCTGAA---AGTAATCC------GATTTGATATCCTCGGTCAATTCGAATTAGCAAAAA

CAATGTCTCCTTGCATAATCTGGATTCCAAACATT----------CATGATCTGT-----

------------------------------------------------------------

---------------ATTTGAATGA---------------------------GTCGAATT

ACTTATCCC-----------TCGGTCTATTAACGAACTTTCTCTCCAGTCTCTACGGGG-

------ATTGTGAAA------CCACT------AGAAATATTCTTGTTTTTGCTTCGACTC

ATCTTCCCCAATTAGTGGATCCCGTTCTAATAGATCCGAAGAAATTCAATACATGCATTA

AGATACGAAGGCTTCTTAGTCCACAACAACGGAAGCACTTTTTCAATCTTTTATATACTA

GGGGATTTCACTCGGAAAATAAAATGTTCCATGCTAACGGATTCGGGTCCATAACCATGG

GTTCCAATGCACGAGATCTTGTAGCACTTACCAACGAGGTCCTATCAATTAGTATTACAC

AGAAGAAATCAATTCTAGAAACTAATACAATTAGATTAGCTCTTCATAGACAAACTTGGG

ATTTGCGATCC---------CATG---TAAGATCGGTTC---CGGATCATGGGATCCTTT

TCTATCAGATAGGAAGGGCTGTTGCACAAAATGTACTTCTAAGTAATTGCTGCACAGATC

CTATATCTATCTATATGAAGAAGAAATCATGTAATGAAGGGTTTTCTTATTTGTACAAAT

GGTACTACGAACTT---------GGAACGAGCATGAAGAAATTAACGATACTTCTTTATC

TTTTGAGTTGTTCTGCCGGATCGGTCGCTCAAGATCTTTGGT------CTCCACCCGGAC

CCGATGAAAACAGTTGGATCACTTCTGATAGATTCGTTGAGAATGATTCTGATCTAGTTG

ATAGCCTA---------------TTAGAAATAGAAGGC------GCTCTGGTAGGATCCT

GGC---------------------------------------------------------

---------------------TTCTTCGGTCCGAACCAAGGAATCCGTTAGATATGATGC

AAAAAAGATCTTGTTCTATCTTT------------------AAAAAAGACGAATCGGAGT

TTGCAGAAGGG------GAAGGAGCCCTCGCCCTCGACCCG------------------C

AAGAGATGCTTTTCTACAATCAAATAGTTTGGGCTCCTAGACTATGGCGCCCT------C

CTTGTGGCAAACTATTTGATTGT-------------------------------------

-----ATCGAATATTCTCAAG---------------------------------------

------------------------------------------------------------

------------AGGATGATG-------------------------------ATGATTGG

GAGTTCTGGCAGAGT----------------GGAA----CCAAGTATTACTGGATGCGAG

ATAG------------------CGAGGGAGGACAGGGCTTTTGGGTTTCTCAACTAAAAA

AATTCCTTTGGAACCCTGAGGCTGCGGATCCATTCCTTTTCCTATTCAAAGATCAGCCCT

TTGTCTCTGATTCTGATTTTTCATCATATCATCAACCAT------TCTCCAAT------T

TTTTGGTAGAGCTGATGCCTCC--------------------------------------

----------TTTC----------------------------------------------

------------------------------------------------------------

------------------------------------------------------------

------------------------------------------------------------

--------------AAAGCTTCTTCTGTATCTCTATATAAAGGCTGGGTCAACAAGAAGT

TGCAAGAAATG------TGCCTCGAATACTTGAGTGATCGCG---AGAGATGGCTTAGA-

-----ACCAATAGTTCATTATCTCATGGATCTTTCCGTTCTAATACTCTATCCGAGAGTT

ATCAGTATTTAGCAAATCTGTTCCTATC---TAACGCAACGCTATTGGATCAAATGACAA

AGACATTGTTGAGAAAGAGATGGCTTTTCCCGGATGAAATGAAAGATTTGATTCATGGAA

CAGGAGGTGGA-------------------ACAGAATTGACCGGGCGGTAG---------

--------ATGCCTAGATCTCGAATAAATGCAAATTTTATTGACAAGACCTCTACAATTG

TAGCCAATATCTTATTACGAATAATTCCGACCACTTCAGGAGAAAAAGAGGCATTTACTT

ATTACAGAGATGGTGCGATTTGATTC--TTTTTTTTTTTTTTTTTTTTTTTTAGCCCTCA

CC--------------------------CCAGTCTTAC--TAAAAAGAG-ACAGGATTCG

G------GATAT-----A-AAAAAAAGATGATGAAAAA----TAAACCTGCGCTTGGTGA

AGGTGAAGT-----TTGAAGATCGAACAATTCCTTCTATCGTGTATCCTCGATTGATGCA

GCCTCAGATACTTCAAT----TGTCGATTCTAGTATTGAGCAAAAGGTTACGCCTATA--

----GGT----TCTGT-ATTG-----CGGGTCAATCCTACTCCC-C----GAATACAAAT

AGGC-GGCATAG----GGGA-ATAAGCACTACACC------T-----AGGAATCAACAAC

ACGAAAACTTTGTTATA---------AATTACCTTTT---CCTTATCGGGATTGGAACTA

ACAA-------------GAATGGTTGGGACAACAAGCATCCATCTCGTTCGTACTTTGGA

TACCCGTAT-----AACCATCGAAGATCGTTGAAGTGACTAATTC--CT-GTAAATAGGG

GGCGTTGAGGACAAAGAAATTGTTGG----AGTTATCATTTCTATCT----AGCACCAAG

ACGGTTGGT--GT------------------TAAGAAAGGACCTCTTGCAGGAAGGATGG

CTAGAGATTTCGTGTAAAAA-TACCAGCCCCTGTATGTTCATAAAA--TAATGAGAATAT

TTCATTCTTT----TTCGATTCCATG-------------------------GATTATTCT

CCTCATTAC---TATGCACAGAA-GGGAGGAGCCATATGAGATGAAAATCTCACGTACGG

TTCTGGAACGGAGATTCTTTGA-ATAAAATAAACGAAAACGACCGTAACGGATGTCAGCC

CAATCCGAAGGAAATTATGCGGAAGCTTTACAGAATTATTATGAAGCTACGCGACCAGAA

ATTGATCCCTACGATCGAAGTTATATACTTTATAACATAGGCCTTATACACACAAGTAAC

GGAGAACATACGAAGGCTTTGGAATATTATTTCCGAGCACTAGAACGAAATCCATTCTTA

CCGCAAGCTTTTAATAATATGGCCGTGATCTGTCATTACGTGCGACTATCTCCACT----

--ATAG------------------------------------------------------

-------------------AAAG-------AAGGAAATAA-AGATCAAATCCGCTAGTA-

-----------------ACT-ACTTACTAGAAAC----------------AAAATAGGCG

TTCTA-CATATGCATTGTCCGAA--GCAACGATTTT----TATCAGCTGTAGCAAAGAAA

GATA------CTTCACGGTAGCC-AAAATAGGA--AGAAATAAGTAG-GCCTGTATACTA

TATTCTATGGAT-----AAAG--GATCGAATTGATAGAGGAAGCGCCGTAAAGATCAATT

AGCGAGGTT---TT-GGGCCGATACAA-T-----AAAAACTGCTTACT----TATCTCAT

GA-TACGAGATA---AAAGTTAGG----AATCAACTTATGTAATAGAGTTGATCCAC---

----TAAAGTACTGAGCAGCGGCGTAGCATCAGATCCCAAAGAT-AGTAAGTCCTTTCTT

TCTTAT--------GAAG---------------GAAAGTCTTTTTC-AAAGATTCTAT-A

TTAAT-----------------CAATTTCTATAT---------GAAACCGAGATAGTTAC

CT-TTCAGAAA-------ATTATAACGATAGAG------GTAGATACTTAT------GCT

TCATTTTTCTGAAGGTGGGAGAAAAGATAAAACTGA-TTATTCATC-----AAAA-TTAG

AGTTTCAAACTT-ATGTAATTAAC-----TTCTTCTGGTTA-AC--------CTAAG---

AACTGGGATAGAT------TTCTTAGAAATCTCAC------------AAATAGGGTAGAT

T-----AGGATGGGAC----GCCAAAAGAATTGATGGCGGAGCCGTATGAGATAGGAAAC

TCTCAAGTACGGTTCTAAGGGAAGGAATTGACCC-------ACCTATTCCGACCGGGGAG

AACAGGCCATTCGACAGGGCGATTCTGAAATTGCGGAGGCTTGGTCCGATCAAGCTGCTG

AGTATTGGAAACAAGCTATAGCTCTTACTCCAGGTAATTATATTGAAGCGCAGAATTGGT

TGAAGATCACAAGGCGGTTCGAA------TAAATGAACTGGCGATCAGAACATATATGGA

TAGAATTTATAACGGGGTCTCGAAAAACAAGTAATTTTGGCTGGGCCTGTATCCTTTTTT

TAGGTTCACTAGGATTCTTAGTGGTTGGAGCTTCCAGTTATCTTGGTAGGAATCTGATAT

CTGTATTCCCATCTCAGCAAATAGTTTTTTTTCCACAAGGGATCGTGATGTCTTTCTATG

GGATTGCAGGACTGTTCATTAGCTCCTATTTGTGGTGCACTATTTCGTGGAATGTAGGTA

GTGGTTATGATCGATTCGATAGAAAAGAAGGAATAGTGTGTATTTTTCGTTGGGGATTTC

CTGGAATAAATCGTCGCATTTTCCTTCGATTCTTTATAAGAGATATCCAGTCTATTAGAA

TAGAAGTTAAAGAGGGTCTTTATTCT---CGTCGTGTCCTTTATATGGAAATCAGAGGTC

AGGGAGCGATTCCCTTGACTCGTACTGATGAGAA---TTTGACTCCACGAGAAATTGAAC

AAAAAGCAGCTGATTCAGCCTATTTCTTGCGTGTCCCAATTGAGG------TATTTTGA-

------------------------------------------------------------

------------------------------------------------------------

--------------------------------

>Acorus_calamus

------------------------------------------------------------

------------------------------------------------------------

------------------------------------------------------------

------------------------------------------------------------

------------------------------------------------------------

------------------------------------------------------------

------------------------------------------------------------

------------------------------------------------------------

------------------------------------------------------------

------------------------------------------------------------

------------------------------------------------------------

------------------------------------------------------------

------------------------------------------------------------

------------------------------------------------------------

------------------------------------------------------------

------------------------------------------------------------

------------------------------------------------------------

------------------------------------------------------------

------------------------------------------------------------

------------------------------------------------------------

------------------------------------------------------------

------------------------------------------------------------

------------------------------------------------------------

------------------------------------------------------------

------------------------------------------------------------

------------------------------------------------------------

------------------------------------------------------------

------------------------------------------------------------

------------------------------------------------------------

------------------------------------------------------------

------------------------------------------------------------

------------------------------------------------------------

------------------------------------------------------------

-ATGGCAACCCTTCGAGCCGATGAAATTAGTAATATTATCCGTGAACGTATTGAACAATA

TACTAGAGAAGTGAAGGTTGTGAATACCGGTACAGTCCTTCAAGTAGGCGACGGCATTGC

TCGTATTCATGGTCTTGATGAAGTAATGGCAGGTGAATTAGTCGAATTTGAAGAGGGTAC

GATAGGTATTGCTCTTAATTTGGAATCCAATAATGTTGGCGTTGTACTAATGGGTGACGG

TTTGACAATACAAGAGGGAAGTTCTGTAAAAGCAACAGGAAGAATTGCTCAGATACCGGT

GAGTGAGGCTTATTTGGGTCGTGTTGTAAATGCTCTGGCTAAACCTATTGATGGGAGAGG

TGAAATTGCAGCTTCTGAATTCCGGTTAATCGAATCTCCTGCCCCAGGTATTATTTCGAG

ACGTTCCGTATATGAGCCCCTTCAAACAGGTCTTATCGCTATTGATTCGATGATCCCTGT

AGGGCGCGGTCAGCGAGAATTAATTATTGGGGACAGACAGACCGGTAAAACAGCGGTAGC

CACAGATACTATTCTCAACCAAAAAGGACAAAATGTAATATGCGTTTATGTAGCTATTGG

CCAAAAAGCATCTTCCGTCGCGCAGGTAGTGACTAATTTCCAGGAGCGAGGGGCGATGGA

ATACACTATTGTGGTAGCCGAAACGGCGGATTCCCCCGCTACATTACAATACCTGGCTCC

TTATACAGGAGCGGCTTTGGCTGAATATTTTATGTACCGTGAACGACATACTTCAATAAT

TTATGATGATCTCTCCAAACAAGCGCAAGCTTATCGCCAAATGTCTCTTCTATTAAGAAG

ACCGCCCGGACGTGAAGCTTACCCAGGAGATGTTTTTTATTTGCATTCCCGCCTTTTGGA

AAGAGCCGCTAAATCAAGTTCTCGTTTAGGTGAAGGAAGTATGACTGCTTTACCAATAGT

TGAAACTCAATCTGGGGATGTTTCGGCTTATATTCCTACTAATGTAATTTCCATTACAGA

TGGGCAAATATTCTTATCCGCCGATTTATTCAATGCCGGAATACGACCTGCTATTAATGT

GGGTATTTCTGTCTCCAGAGTAGGATCCGCAGCTCAAATTAAAGCCATGAAACAAGTAGC

TGGCAAATCAAAATTGGAACTAGCTCAATTCGCGGAGTTAGAAGCTTTTGCACAATTCGC

TTCTGATCTCGATAAAGCTACTCAGAATCAATTGGCAAGAGGTAAACGATTGCGCGAGTT

ACTTAAACAATCCCAATCAGAACCTCTCGCGGTGGACGAACAAGTGGTTACTATTTATAC

CGGAACGAATGGATATCTTGATACGTTAGAAATTGGGCAGGTAAAGGAATTTCTCGTTAA

GTTACGTACCTACTTAAAAAAGAATAAACCTCAGTTTCAAGAAATCATATCTTCTACCAA

GACCTTTACCGAGGAAGCAGAAGCTCTTTTGAAGGAAGCTATTCAGGAGCAGCTCGAACT

CTTTCTACTTCAGGAACAAACA---------------------TAAATGAGAATAACTCC

TACTAC------TTCTGGTT------CTGCGGTTCCCTCACTTGACGAGAAAAACTTAGG

GCGTATCGTCCAAATCATTGGCCCAGTACTGGATGTCGCCTTTCCCCCGGGAAAGATGCC

TAATATTTACAACGCTTTGGTGGTTAAGGGTCGAGATACTGCCGGTCAGCCAATTAATGT

GACTTGTGAGGTACAGCAATTATTAGGAAATAATCGGGTTCGAGCTGTGGCTATGAGTGC

TACAGATGGTTTGACGAGAGGAATGGAAGTGATTGACACGGGAGCTGCTCTAAGTGTTCC

AGTCGGTGGAGCTACTCTCGGAAGAATTTTCAACGTTCTTGGGGAGCCCGTTGATAATTT

AGGCCCTGTAGATACTCGCACAACATCTCCTATTCATAGATCTGCGCCTGCATTTATACA

GTTAGATACGAAATTATCAATCTTTGAAACAGGCATTAAAGTGGTAGATCTTTTAGCTCC

TTATCGCCGGGGAGGAAAAATCGGACTATTTGGGGGAGCTGGAGTGGGTAAAACAGTACT

CATCATGGAATTGATCAACAACATTGCCAAAGCGCATGGAGGTGTATCCGTATTTGGCGG

AGTAGGCGAGCGTACTCGTGAAGGAAATGATCTTTACATGGAAATGAAAGAATCCGGAGT

AATTAATGAAGAAAAGATTGAAGAATCTAAAGTAGCTCTAGTGTATGGTCAGATGAATGA

ACCGCCGGGCGCTCGTATGAGAGTTGGTTTGACTGCCCTAACCATGGCGGAATATTTCCG

GGATGTTAATGAACAGGATGTACTTCTATTTATCGACAATATCTTCCGGTTCGTACAAGC

GGGATCAGAAGTATCTGCCTTATTAGGGCGGATGCCTTCCGCCGTGGGTTATCAACCTAC

CCTTAGTACAGAAATGGGTTCT---TTGCAAGAAAGAATTACTTCTACAAAAGAGGGATC

TATAACGTCTATTCAAGCGGTTTATGTACCTGCGGATGATTTGACTGACCCTGCTCCTGC

CACCACATTTGCACATTTGGATGCTACTACTGTACTATCAAGAGGATTAGCTGCCAAAGG

TATTTATCCGGCAGTAGATCCTTTAGATTCAACGTCAACTATGCTCCAACCTAGGATCGT

TGGCGAGGAACATTATGAAACTGCGCAAAGAGTTAAGCAAACTTTACAACGTTACAAAGA

ACTTCAGGACATTATAGCTATCCTTGGGTTGGACGAATTATCCGAAGAGGATCGTTTAAC

CGTAGCAAGAGCACGAAAAATTGAGCGTTTCTTATCACAACCCTTCTTCGTAGCAGAAGT

ATTTACTGGTTCTCCAGGTAAATATGTTGGTCTCGCGGAAACGATTAGGGGGTTTCAACT

GATCCTTTCAGGAGAATTAGATGGTCTTCCCGAGCAGGCCTTTTATTTGGTGGGTAACAT

CGATGAAGCTACCGCGAAGGCTATGAATTTAGAAGTGGAGAGCAAATTGAAGAAATGAAT

GAACTTAAAGCTTTGTTTACTGACTCCTAATCGAATTATTTGGGATTCAGAAGTGCAAGA

AATCATTCTATCTACTAATAGTGGACAAATTGGCATATTACCAAATCACGCCTCTATTGC

CACAGCTGTAGATATCGGTCTTTTGAGAATACGCCGC---GACGACCGATGGTTAGCGGT

GGCTCTGATGGGGGGGTTTGCTAGAATAGGAAATAATGAGATCACCATTTTAGGAAATGA

TGCCGAGATGGGTACTGACATTGATCCGCAAGAAGCTCAACGAGCTCTTGAAATAGCTGA

AGCTAACTTGAGTAAAGCTGAAGGTAAGAGACAAGCAATTGAGGCAAATTTAGCGCTAAG

ACGAGCTAGGACACGGGCGGAGGCTATTAATG---------TTATT------------TC

CCAATAGATGAAAAATATAACCGATTCTTTCGTTTCCTTG---GGCCACTGGCCATTCGC

CGGGAGTTTCGGGTTTAATACCGATATTTTAGCAACAAATCTAATAAATCTAAGTGTAGT

GCTTGGTGTATTGATCTTTTTTGGAAAGGGAGTGTGTGCGAGTTGTGTATTTCAAGAATA

-GGCCGGATTCGGCCGGCTGTACTTTA---------------------------------

------------------------------------------------------------

----------------------------TTTCTTA-------------------------

------------------------------------------------------------

------------------------------------------------------------

------------------------------------------------------------

------------------------------------------------------------

------------------------------------------------------------

------------------------------------------------------------

------------------------------------------------------------

-----------AAAAAAAAAAAAAATTCGGAAAGA-------AAGGTGCACCATCTCG-A

CGAATTACTTCTGAATA----------AATTCATAAATCAATC-----ATATGTAAGAAC

CATAGCATTTCGTGATTCATTGGT-----AAATTAACTTTGA-----TTCTCTATTAA--

---CCAAGAATA-TGGGACCA-----TTAACATGGTTAAAGCT-AAACCGTTTGAAGTCT

AGGC--ATAACAC-----GGTACTCTTTCTACCGCTACGTTAGTA------CCAAGATGG

TT-------TCAAAATG----------------AATAGTGGAAAATTTATCCAATATAGA

ACACTCATATTGATAAAATCATT-TGAACTGGACCATTTACTAA----------------

--AA--AAGGGGGAGCGCCTCACCCTT------TTATCCAATGCTGAATCGACGACCTAT

GTAT---------AAAAT--------AAGAATAA--TTTTTTGGATT--------TGAAG

------------------------------------------------------------

------------------------------------------------------------

------------------------------------------------------------

----------------------------------------------------------AA

AAAAAAAACACACACAACTTTGCTGATAATTACAGATTTATTT-GTCTGGTCGGAAGAGC

TC-----------TCC--GAATAT-----TCTGGTCTTGTAT-----CAATCAGTTT---

-----------------------------------------TGATATTTTGGAATATGAG

TAGAAAAG-------AGAGGGTAGGCTCATTACAT-----TAACAGATATGGGAAAA-CC

TATA---------------------A--GTAATTGAGCGTGAGAGCCAAATGAATCGAAA

GATTCGTGTTTGGTTCGGGAAGAGATCA-TGGGATTCGTGAAAT-----AAGTAGTCAGA

TAATCTACTTTCATTAAGTGATTTATTAGATAATCGAAGACAGAGGATCTTGAGTACTAT

TCGAAATTCAGAAGAACTGCGTAGAGGAGCCCTTGAGCAGCTCGAAAAAGCCCGGGCTCG

CTTACGGAAAGTAGAAATGGAAGCAGATGAGTATCGAGTGAACGGATACTCCGAGATAGA

ACGAGAAAAGATGAATTTGATTAATGCCACTTATGAAAATTTGGAACGATTAGAAAATTA

CAAAAATGAAACCCTTCATTTTGAACAACAAAGAGCAATTAATCAGGTCCGGCAACGAGT

TTTCCAACAAGCGTTACAAGGAGCTCTAGGAACTCTGAATAATTGTT------TGAATAG

TGAATTACATTTCCGTACCATCAGTGCTAATATTGGCATGCTTGGGGCCATGAAA-----

GAAATAACTGATTAGATGAATCCACTGATTTCTGCCGCTTCCGTTATTGCTGCTGGATTA

GCCGTAGGACTTGCTTCTATTGGACCTGGGGTTGGTCAAGGTACTGCTGCAGGCCAAGCC

GTAGAAGGTATTGCGAGACAACCAGAAGCAGAAGGTAAAATACGAGGTACTTTATTGCTT

AGTCTGGCTTTTATGGAAGCTTTAACAATTTACGGACTGGTCGTAGCATTAGCACTTTTA

TTTGCGAATCCTTTTGTCTAAATGAATGTTATACTATGTTCCAGCAACATGCTCAAAGGG

TTATATGATATATCTGGTGTAGAAGTGGGCCAACATTTATATTGGCAAATAGGAGGGTTC

CAAGTCCATGCCCAAGTACTAATCACTTCTTGGGTCGTAATTGCTATCTTATTAGGTTCA

GTCACTGTCGCTGTTCGGAATCCACAAACCATTCCGACCAACGGTCAGAATTTCTTTGAG

TATGTTCTTGAATTCATTCGAGACTTGAGCAAAACACAGATTGG---AGAAGAATATGGT

CCTTGGGTTCCCTTTATTGGAACAATGTTCCTATTTATTTTTGTTTCGAATTGGTCAGGC

GCTCTTTTACCTTGGAAACTCATAGAGTTACCTCACGGGGAGTTAGCCGCACCCACGAAT

GATATAAATACTACTGTTGCTTTAGCTTTACCCACATCTGTAGCATATTTCTATGCGGGT

CTTACCAAGAAAGGATTGGGTTATTTCGGGAAATACATTCAACCAACTCCGATCCTTTTA

CCAATTAATATCCTAGAAGATTTCACAAAACCTTTATCACTGAGTTTTCGACTTTTCGGA

AATATCTTGGCTGATGAATTAGTAGTTGTTGTTCTTGTTTCTTTAGTACCTTTGGTAGTT

CCTATACCTGTCATGTTTCTTGGATTATTCACAAGCGGCATTCAAGCTCTTATTTTTGCA

ACTTTAGCCGCGGCCTATATAGGCGAATCCATGGAAGGTCATCATTGAATGATATTTTCA

ACTTTAGAACATATATTAACTCACATATCTTTTTCGATTATTTCAGTTGTGATTACAATT

CAGTTGATGAACTTATTAGTCCACGAACTGGTACAACTAGGTGATGCGTCAGAAAAAGGC

ATGATAGCTACCTTTTTCTCTATCACGGGATTATTGGTTACTCGTTGGATCTATTCGGGT

CATTTCCCACTAAGTGATTTATATGAATCATTAATCTTCCTTTCATGGAGTTTCTCCATT

ATTCATATGGTTCCATATTTTAGGAACCACAGAAATC------ATT---TCAGTGCAATA

ACTGCGCCAAGTGCCATTTTTACCCAAGGCTTTGCTACTTCGGGTCTTTTAACGGAAATG

CATCAATCCATAATATTAGTACCCGCTCTACAATCCCAGTGGTTAATGATGCATGTAAGT

ATGATGTTATTGAGCTATGCAGCTCTTTTATGCGGGTCATTATTATCAGTTGCTCTTCTA

GTTATTACGTTTCGAAAAAACAGCGATATTTTTGA------TAAAAGA---AATAATTTC

TTAATT------AGGTCATTTTTC---------TTTGGTGAGATTGAA------TACTTG

AATGAAAA---ACGC---------AGTGTTTT---ACAAAACACTTCGTTT---GATTCA

TTTACAAATTATCACAAATATCAATTGACTCAACGATTAGATTTTTGGAGTTATCGGGTC

ATTAGTCTAGGGTTTATCTTTTTAACCATAGGTATTCTATCGGGGGCAGTGTGGGCTAAT

GAGGCATGGGGATCTTATTGGAATTGGGACCCAAAGGAAATTTGGGCATTTATTACCTGG

GCTATATTCGCAATTTATTTACATACTAGAACGAATCATAGTTTGCAAGGT------GCG

AATTCGGCAATTGTGGCTTCTATCGGATTTCTTATAATTTGGATATGTTATTTTGGAGTC

AATCTATTAGGAATAGGTTTACATAGTTATGGTTCATTC------ACATTA-ATATCTAA

T------------------------TGA--------------------------------

------------------------------------------------------------

------------------------------------------------------------

------------------------------------------------------------

----------GTGCCAAAAAAGAAAGGATTTACTCCCCTCCCATATCTTGCATCTATAGT

ATTTTTGCCCTGGTGGGTCTCTCTCTCATTTAATAAGAGTCTGGAACCTTGGGTTACCAA

TTGGTGGAATACCAGACAATCTGAAACCTTTTTGAATGATATTCAAGAGAGGAACGTTCT

AGAAAGATTCATCGAATTAGAAGAACTATTCCTGTTGGACGAAATGCTAAAGGAGAACCC

GGAGACACGTATGAAAAATCTTCGTATAGGAATCCACAACGAAACGATCCAAT-TGGTCA

AAACAGACAATGAGTATCATCTCCATACCATTTTGCATTTCTCGACAAATATAATCTGTT

TCGCTATTCTAAGTGTTTATTCTATTCTGGGTAATGAAGAACTTGTCATTCTTAATTCTT

GGGTTCAGGAATTTCTTTATAACTTAAGCGACACAATAAAAGCTTTTTCGATTCTTTTAG

TTACTGATTTATGGATTGGATTCCATTCACCCCACGGTTGGGAACTAATGATTGGTTCGG

TCTACAATGATTTTGGATTAGCTCATAACGAACAAATTATATCTGGGCTTGTTTCCACTT

TTCCAGTTATTCTAGATACGATTGTGAAATATTGGATCTTCCATTATTTAAATCGTGTCT

CTCCTTCACTTGTAGTGATTTATCATTCAATGAATGA---CTGAATGCCCATTGGTGTTC

CAAAAGTACCTTTTCGGAGTCCTGGAGAG---GAAGATGCAGCTTGGGTTGACATATAGT

GCGACTTGTCAGACATATTTGGTCCTATGGAATTTC-CCCGTTCTCTCCTCTGCTCTGAT

CGAGATATCCTCTGTTTCGCCCAA----------------GAAAG-----------ATTA

ATTGAACTATCAAT----------AACTCGGAGCGTGAA--ATGTAATTAGATCGACTTT

TTTGGAGGACCAATATT-----CTCAATTATAATAATTTATGGTTT-CTCAGGCCAAT--

-----AAAATAAAG-----TATTCAGGCTTCGTTCATAAAATACCAAATTTGTAATAA--

------------------------------------------------------------

------------------------------------------------------------

------------------------------------------------------------

-----------------------------------------------------ATATTTA

TATGATCCTAT-----------TTATAAATATCA--------------------------

--------------TAGTAGTGATCTC-----------AAACTGCC-----AACCAATGA

AGTAATATGAT--GA---ATACATCGAATATT---TTGTCGGAAGGTATGAAATGAATTG

----------------AAAAAAAAAAGCAATA----------------------------

-ACAAAAGGAAGTGGTACTGGTGGGGTTATTTGTCCTATATGTGCAAATCAAATTCGAAC

AGATCTTTTCCCGGAGTAG----AACATGAACCTAAAAGAAAAGAATTGAAAGAAAAGAA

TTGAAG-AGGCCTTTTCGGGAACAAGAAAATTACATCGTGATTTGAATCTCTATGAAACA

ATACCATCAACGAGAG----------GTTAATTCAAAAAGTTCAATGACTTTTTATATAT

TTT----------------------------TATAGAAAAGGGAGCCAAAGCTC------

---GGGTTTCATATTAAAAAAAAAAAAAAACAA---------------------------

--------TAAAAAAAAATTAGGGCTAGAATCGATACATTGATCC----TTTTTCTC---

---AATTTTT----TGAACCGTATGCATACAAAGGTGCATGTACGGTTCTTAGGGAATAC

AATTTTGTCTTAATCAACCGACTTCATCGAGAAAGATTACTTTTTTTAGGCCAAGAGCTT

GATAGCGAAATCTCGAATCAACTTGTTGGTCTCATGGTATATCTCAGTATAGAGGATAAG

ACCAGGGATTTTTTTTTGTTTATA--AATTCTCCCGGCGGATGGGTAATTCCGGGAATAG

GTCTTTATGACACTATGCAATTTGTGCCACCCGATGTACATACAATATGCATGGGATTAG

CTGCTTCAATGGGATCTTTCATTCTTGTCGGAGG-------AGAAATTACCA---AACGT

CTAGCATTCC-CTCACGCTTGGCGCCAATGAGTTTT---TTTATTT------------GA

GAAAAAGAAGACTATGCC-TTCGCCATATCCAATAGGAA---------------------

------------------------------------------------------------

-------------------------TTATAAGTAATAATAGCATGGCACGTCGAGTTCGA

TATGAACAAACCATTATTGTT---------TTTTCAAAACATGAAATTCTGTATCGAGAT

AGTAGTATGAAAC-AAAGGTTATTTCCGAC----TTTATCTTAT-------GTAT----G

AGAGATCCATT--CTAGCGTCACAAACCTTTTTGCTTCCACACCAGA----AGTCTCTTT

CCGATATTTCTGTTATGAAAATGAAAGAAAGAGTACGAAAAAAAAAAAAAAAAAAAGAGC

ATTTTTGACTTATTTTTGTTTGA---TCAGATCAGAAAGAAACTTTGGGATTGCTGAATC

ACAGATGAGATAAAT-------------ATATAAAGCAACAGAACCATCATAGTAT----

-------------------TTTTGGAGCCTACGAAACAAAAGGAAGGTTGGTAAATGGAT

CATTCAACGAT----CCGGGTCAGGTGGATTCGATTTATCTC-----------TTTTTCG

A-----TGGAAGGAAGGGAAAAGTAAATTCCATTGCAGAGCCGTATGC-----ACAAAAG

GTGCCTGTACGGTTGTTCACTTCTATCTTTTTCTTCTTTATCCCTTCT------------

-------------TTTAGCCCGATCATGTG------AGATAGAAAAACCATTCAT----G

ATGTTATATG-----------ATCAGGGTTATGATCCACCAACCTGCCAGTTCTTTTTAT

GAGGCACAAGCAGGGGAATTTGTCCTGGAAGCGGAAGAACTATTGAAACTTCGC----GA

AACCCTCACAAGAGTTTACGTA--CAAAGAACTGGTAAACCATTATGGGTTGTATCCGAA

GATATGGAAAGGGATGTTTTTCTGTCAGCAACAGAAGCCCAAGCTCATGGTATTGTTGAT

CTTGTAGGGGACGAAA------------AT------ATGG-------GAGATTTAGTGTA

A-----------------ATGAAAGAACA-------------------------------

-----AAAATTGATTCAT---GAAGGTTTAATTACTGAATCACTTCCTAATGGTATGTTT

TGGGTG------CGTTTAGATAATGAAGATCTGGTTCTAGGTTATATTTCTGGAAGGATC

CGACGCAGTTCTATACGGATACTACCGGGAGATAGAGTAAAAATCGAAGTAAGTCGTTAT

GATTCAACCAGAGGACGCATAA--TTTATAGACTTC------------------------

------------------------------------------------------------

------GCAATAAAGATTCGAACGAG------------TAAATGGAAGAAT---------

---TCAAAGTCTATTTCG-----AAAAAGATGG----GTCTCGTCAACAATAC------T

TCCTATATCCACTTCTCTTTCAGGAGTACATCTACGCACTTGCTCATAATCATGTTTTAA

ATGG------ATTGATTTTTTACGAATCCTCGGAAAATTTCGTTT------ATGATAATA

AATTTAGTTTAATAATTGTAAAACGTTTAATTACTCAAATGTATCAACAGAATTCTTTGA

GTAATTTGGTTAATGATTCTAACC------GAAATCGATTAGTTCGGCAAAACAAGAA--

-------------TTTTTATTATCAAACGATATTAGAGGGTTTTTCAGTCATTATGGAAA

TTCCATTCTCCATAATATTTTTATCTTCCGTTGAAGAAAAAAAAGCAAAAATACCAAAAA

TTCA------GAATTTACGATCTATTCATGCAACATTTCCCTTTTTAGAAGACAAATTAT

CACATTTAAATCATGTATCAGATATATTAATACCCTATCCCATCCATCTCGAAATCCTGG

TTCAAGTTCT------GCAAGGCTGGATACAAGATGTTCCGTCTTTACATTTATTGCGGT

TCTTTCTCCATGAGTTTCATAATTGGAATAGTCTC---ATTAC------TCCAAAGA---

------AATCCATTTCTTTTCTT---TTTTCAAAGGGGAATCAAAGACTCTTCTTGT---

TCTTATATAATTCTTATGTATTTGAGTGTGAATCCGCATTAGTCTTTCTCCGTAAAC---

AATCCTTTTATTTACGATCAACATCTTTTGGAACCTTTGTTGAGCGAACACA-TTTCTAT

GGAAAAATAGAGC---------------------ATATTG---------TAGTAGTGCGT

AGG---AAGAATTT------TCA--AAAGGCCTTA------TCTTTGTTCAAAGATCCTT

TCATCCATTATATCCGATATAAAGGAAAATCAATTCTGGCTTCAAAGGGGACTCATTTTC

TGATGAAGAAATGGAAATATCACCTTCTAAATTTCTGGCAATGTCATTTTGACTTTTGGT

CTCAACCGCATAGGATTCATATAAACCAATTATCAAATCATTCTTTCTATTTTATGGGCT

ATCTGTCAAGTGTACGAA-----TCAATTTTTCGACGGTAAGGAGTCAAATGCTAGAGAG

TTCATTTCTAATGGATACTCCTACTAAA------AAATTCGATACTATAGTCCCAATTAT

TCCTCTAATCGGATCATTGGCTAAAGCTAAGTTTTGTAACGTATCGGGGCATCCTGTTAG

TAAGCCGGTCTGGGCTGATTTGTCAGATGCTGATATTATTGATCGATTTGGACGAATATG

TAGAAATCTTTCTCATTATCACAGTGGATCCTCAAAAAAACAGAGTTTGTATCGAGTAAA

GTATATACTTCGCCTTTCGTGTGCGAGAACTTTGGCCCGTAAACATAAAAGTACAGTGCG

CGCTTTTTTCAAACGATTAGGTTCAGAATTCTTAGAAGAATTCTTTACGGATAAAGAACA

AGTT--------CTTTCTTTGATC-----TTCCC---------TTCCCTTCGTTCACCTT

CACACAGGGTCCATAAAGGAGAACGTATTTGGTATTTGGATATTATCCGTATCAATGACC

TGGTGAATA------------------------------------------ATTCATGA-

--------------------------------------------------ATGATAATTG

CTACGAC------AGAAATACAAGCTATCAATTCTTTTTCTAGATCGGAATCCTTATCCT

TAAAAGAGGTCTACGGACTCATATGGCTGCTTGTACCTATTTTTACTCTTATATTGGTAA

TTATAATAGGGGTACTAGTGATTGTTTGGTTGGAAAGAGAAATATCTGCGGGGATACAAC

AACGTATTGGGCCTGAATATGCCGGCCCATTGGGAATTCTTCAAGCTCTAGCGGATGGAA

CCAAACTGCTTTTCAAAGAAGATCTTCTCCCATCTAGAGGAGATATTAGTTTATTCAGCC

TCGGGCCGTCTATAGCGGTCATATCAACTCTACTAAGTTATTTAGTAATTCCTTTTGGCT

ATCACCTTGTTTTAGCTGATCTCAGTATCGGCGTTTTTTTATGGATTGCCATTTCAAGTA

TTGCTCCTATTGGACTTCTTATGTCAGGATATGGCTCGAATAATAAATATTCCTTTTCAG

GTGGTCTACGAGCTGCCGCTCAATCTATTAGTTATGAAATACCATTAACTCTATGTGTAT

TATCAATATCTCTACGTGTGATTCGTTGAAACATGAACTTTTATCCCCCCTTTTATCCCC

TTTCCTTTTTAAGGGGG-------------T-----------------------------

---------TGAATGTATTGAATACATCTCTTCAGCTTTTATTCATCATTCG--------

--------------------ATTCGGGTTGATAAACTGAACTAG------ATAGTTATAT

GAGTGAAATAAAAC-----AGCTTAGAAATTCGCAGTAAGAAGATTGAATCTCATTCCCT

AT-GTACGAGAGTAAAGTCGAAGTAAACATAAG-----CAGTGCAAACGATTTACCCCAA

GATTTAGATT---------GGCATCATAT-----CTTGAAGCGGGT----GCAAAAGATC

CACTATGTATGGACGTTCTACTATTGTCTTGT----------------------ATGTAT

TACCATACCGGGGATCAATCAAAAATGAGTGGACATTTAGGAACA-CCAAGGTACA----

-------CAAAGGATTAGTAATGGAGATAATATAACGTGTCAAAAGAGGTATT-TCCTCA

TTCA----------TAAAACGAATCAAAAT-------GGGCTTTAAGTTCGTAGAAATGA

TCGAGCAGTACTTCCCTATGATTCCGATCTAGAGTAT-ACTCCTATTCACTCATTAAAGA

AA--TAACTATCAGG-AACGAATTAATCCCTTATTTTCTTTT----------CGAGTACC

TTCCTTTGAGAAAGAAGAACTGGAATAAAAGAAATAGAATACTATTCTAGG---------

--------AAAAATGAATAATAAAGGATCTTTCTTT------------------------

-----ATTCTTTCTTTCCTC------T-ACCCATA----TTCAGACATACGGAATTCTTA

T---C----------ATTATTCATGAACTAG----------TATAGTATACC-TAATTCT

TTTCATAATAGAGGGGTATGGG-------TCGAAATATCTATTAATACAGCGAGTATTTT

AT------TGAAGGATTAAGTTATTACTGA-ACAAAGAG--AAATCAAAATCATAA----

---AGGATAAGATCAATT-CAGAAGCG----CTTTTATT-----TATTATT-TTCTAGCA

GACAGAATTCGATTGGTCTAATTCA-----GGACTCCCCGCTGCATCTTTATTTATTCTA

TATAACCTAG--------AATGAGATGCTGAGTGAATTAATTTAATTTAATACCAAACAA

ACCAG--------TCCTTAGATTTATTTGT----GACCATAAAGGAGCCGTATGAGGTGA

AAATCTCATGTACGGTTCTGGAATAGCAATGAGAACAGTGATGTTTTCATCGACTATGAT

TATCTAATAGTTCAAGTACAGTTGATATAGTTGAGGCACAGTCAAAATATGGTTTTTGGG

GATGGAATCTGTGGCGTCAACCTATAGG-ATTTTTAGTTTTCCTAGTTT--CTTCTCTAG

CCGAATGTGAAAGATTACCCTTTGATTTACCAGAAGCAGAGGAAGAATTAGTAGCAGGTT

ATCAAACTGAATATTCAGGCATCAAATTTGGTTTATTTTACGTTGCTTCTTACCTAAATC

TACTAGTTTCTTCATTATTCGTAACAGTTCTTTACTTAGGCGGGTGGAATCTCTCTATTC

CATACATATTTATTCCTGAACTTTTCGGA---------AAAAACAAAACAGGTGGAATCT

TTGGAATGACAATTGGTATCCTAATTACATTAGCTAAAGCTTATTTGTTCCTGTTCATTT

CTATCGCAACAAGATGGACTTTACCTAGGTTAAGAATAGACCAACTATTAAATCTTGGAT

GGAAATTTCTCTTACCTATTTCCCTCGGTAATCTATTATTGACAACCTCTTCCCAACTTG

TTTCACTATAA-------ATGATCTGGCATGTACAGAATGAAAACTTCATTCTCGATTCT

ACGAGAATTTTTATGAAAGCGTTTCATTTGCTTCTCTTCGATGGAAGTTTCATTTTCCCA

GAATGTATCCTAATTTTTGGCCTAATTCTTCTTCTGATGATCGATTCAACCTCTGATCAA

AAAGATAGACCTTGGTTCTATTTCATATCTTCAACAAGTTTAGTAATGAGCATAGCGGCC

CTATTGTTCCGATGGAGAGAAGAACCTATGATTAGCTTTTCGGGAAATTTCCAAACGAAC

AATTTCAACGAAATCTTTCAATTTCTTATTTTACTATGTTCAACTCTATGTATTCCTCTA

TCCGTAGAGTACATTGAATGTACAGAAATGGCTATAACAGAATTTCTGTTATTCGTATTA

ACAGCTACTCTAGGAGGAATGTTTTTATGTGGTGCTAACGATTCAATAACTATCTTTGTA

GCTCCAGAATGTTTTAGTTTATGCTCCTA-----CCTCTTATCTG-------G-ATATAC

CAAGAG---AGATGTACGG--TCTAATGA-GGCTACTATGAAATATTTACTCATGGGTGG

GGCAAGCTCTT-CTATTCTGGTTCATGGTTTCTCTTGGCTATATGGTTTATCCGGGGGGG

AGAT-CGAG-CTTCAAGAAATAGTGAATGGTCTTATCAATACACAAATGTATAACTCCCC

GGGAATTTCAATTGCGCTTATATTCATCACTGTAGGAATTGGGTTCAAGCTTTCCCTAGC

CCCTTTTCATCAATGGACTCCTGACGTATACGAAGGAGTGCGGTTCGTTCGACAAATTCC

TACCTCTATATCTATCTCT------GAGATGTTTGGATTTTTCAAAACTCCGTGGACATG

CAGAAGAGAAATGCTATCCCCACTCGGACCAAGACATAACTTTTACC-----AAAAGTTT

ATTGTGATCTTTTTGT----------TCAAATAACAATTAAGG----TGAAGCAGGGTCA

GGAACAACGAATC-TCTTTAT-GATAAACAGATCCATTTTGCAAGTTCGTTATTA--CGG

GTAGTTCCCACAAAAGATCGGACTAATG-ACGTATACAATACTTGAATTATCG-ATGTAG

ATGCTACATAGTTGGTTCTCAT-CCTTCAGAGACTACGAGTGTAATAGGAG---CATCCG

TCGACAAAAGGATCACC------------CTAAGATGAT---------------------

-CATCT--------CATGGCTATTGAGAACGAATCAAATCAGACGGTTCTATTTCTCAAT

C-TTTCTGACTTGCTCCTACGG--AACCAAAGTCGAAAAGATTG--AAAAAGTCAGTCAT

TCACAACCACTGATGAAGGATTCCTCGAAAAGTTAAGGAT-----TAGTAATCCTTTTTA

GAAATCG------------AATGGATTCAGTCTTATACATACGCGAGGAAGGTAATCAAA

AAAGAAAGAAGAAC----------------------------------------------

------------------------------------------------------------

-------------------TC-ATCTTCTTTCTTTTATCACTTAGGAGCCGTGCGAGATG

AAAGTCTCATGCACGGTTTTGAATGAGAGAAAGAAGTGAGG-AATCCTC-TTTTCGACTC

TGACTCTCCCACTCCA-GTCGTTGCTTTTCTTTCTGTTACTTCGAAAGTAGCTGCTTCAG

CTTCAGCCACGCGAATTTTCGATATTCCTTTTTATTTCTCATCAAACGAATGGCATCTTC

TTCTGGAAATCCTAGCTATTCTTAGCATGATATTGGGGAATCTCATTGCTATTACTCAAA

CAAGCATGAAACGTATGCTTGCATATTCGTCTATCGGTCAAATCGGATATGTAATTATTG

GAATAATTGTTGGAGACTCAAATGATGGATATGCGAGCATGATAACTTATATGCTGTTCT

ATATCTCCATGAATCTAGGAACTTTTGCTCGCATTGTATTATTTGGTCTACGTACCGGAA

CTGATAACATTCGAGATTATGCAGGATTATACACGAAAGATCC-TTTTTTGGCTCTCTCT

TTAGCCCTATGTCTCTTATCCCTAGGAGGTCTTCCTCCACTAGCAGGTTTTTTCGGAAAA

CTCCAT---------CTCTTCTGGTGTGGATGGCAGGCAGGCCTATATTTCTTGGTTTCA

ATAGGACTCCTTACGAGCGTTGTTTCTATCTACTATTATCTAAAAAT-------AATCAA

GTTATTAATGA--CTGGACGAAACCAAGAAATAACCCCTCACGTGCGAAATTATAGAAGA

TCTC------CTTTAAGATCAAACAATTCCATCGAATTGAGTATGATTGTATGTGTGATA

GCATCTACTATACCAGGAATATCAATGAACCCCATTCTTGCAATTGCTCAGGATACCCTC

TTTTAGATGTTTCTGCTTCACGAATAT--GATATTTTCTGGGCATTTCTACTA-----AT

ATCAAGTGTTATTCCTATTTTAGCATTTTTAATTTCCGGAGTTTTAGCCCCAACGAGGGA

AGGACCCGAGAAGCTTTCTAGTTATGAA-TCGGGTATAGAA-CCAATAGGGGATGCTTGG

G-TACAATTCCGAATCCGATATTATATGTTTGCTTTAGTTTTTGTTGTTTT--TGATGTT

GAAACGGTCTTTCTTTATCCATGGGCAATGAGTTTCGATGTATTGGGTGTATCCGTATTT

CTAGAAGCTTTGA--TTTTCGTGCTTATCCTAATTGTTGGTTCAGTTTATGCATGGCGAA

AGGGGGCATTGGAATGGTCTTAG---ACGAGTTATTTTCC------TTGGTTAACCATAA

TTGTTGTTTTTCCGATATCCGCAGGTCTTTCAATTTTCTTTCTCCCGCATAGAGGGAATA

AGGTGGTTCGGTGGTATACTATATGTATTTGTTTGC------TAGAACTCCTTTTAATGA

CCTA-----------TGTGTTCTGTTATCACTT-TCAATTGAACGATCCATTAATCCAAT

TGGATGAAGATTATGAATGGATAAATATTTTTGATTTTCACTGGAGACCAGGAATCGATG

GACTTTCCATAGGACCCATTCTACTGACGGGATTTATCACTACTTTAGCTACTTTAGCGG

CTTGGCCGGTTACTCGAGATTCGCGATTGTTCCATTTCCTGATGTTAGCAATGTACAGTG

GTCAAATAGGATTATTTTCTTCTCGAGACCTTTTACTTTTTTTTCTCATGTGGGAGTTAG

AATTAATTCC-CGTTTACCTACTTTTATCTATGTGGGGTGGGAAGAAACGGCTGTACTCG

GCTACCAAGTTTATTTTGTACACAGCGGGGGGTTCTGTTTTTCTTTTAATGGGAGTTCCG

GGTATGGGTTTA------TATGGTTCCAATGAACCAACATTAAATTTTGAAACATCGGCT

AATCAATCGTATCCTGTGTCATTGGAAATACTATTTTATTTTGGATTCCTTATTGCTTAT

GCTGTCAAATTACCGATTATACCCCTCCATACATGGTTACCAGATACCCATGGAGAAGCC

CATTACAGTACATGTATGCTTCTAGCTGGAATCTTATTAAAAATGGGAGCATATGGATTA

GTTCGGATCAATATGGAATTATTGCCCCACGCTCATTCTATATTTTCCCCCTGGTTGGTA

CTAGCGGGAACGTTGCAAATAATCTATGCAGCTTCAACTTCTCTTGGTCAAGTTAATTTA

AAAAAGAGAATCGCCTATTCCTCCGTATCTCATATGGGTTTCACAATTATAGGAATTGGT

TCTATAACCGATACAGGACTCAATGGAGCCATTTTACAGTTACTCTCTCATGGATTTCTT

GGTGCTGCACTTTTTTTCTTAGCGGGAACAAGTTGTGATAGAATACGTCTTATTTATCTC

GACGAAATGGGGGGAATATCC-------------ATCCCGATGCCCAAAATATTTACCAT

GTTCAGTAGCTTCTCAATGGCTTCTCTTGCATTGCCAGGAATGAGTGGTTTTGTTGCGGA

AGCAGTAGTATTTTTTGGAATAATTACTAGTCAAAAATTTCTTTTTTTGCCAAAAATACT

AATTACTTTTGTAATGGCAATTGGAATGATATTAACTCCTATTTATTTATTATCCATG--

------TTACGCCAGATCTTCTATGGATACAAGCTATTTAATATGACAAACTCTTATTTT

ATGGATTCTGGACCACGAGAACTATTTGTTTCGATCTGTATC-TTTTTGCCCGTAATA-G

GTATTGGTATTTAT---CCCGATT-TCGTTCTATCGTTATCAGTTGACAAGGTGGAAGCT

ATTTTATCTAATTACTTTTATAGATAGATGATGCTGGAGTATGTACTTTTTTTGAGTGCT

TATTTATTTTCCATCGGTATCTATGGATTGATTACAAGTCGAAACATGGTTAGAGCACTT

ATGTGTCTTGAACTTATACTGAATGCGGTTAATATCAATCTCGTAACATTTTCTGATTTA

TTTGATAGTCGCCAATTAAA----------------------------------------

-----------------------------------AGGAGACATTTTTTCGATCTTTGTT

ATAGCTATTGCAGCCGCTGAAGCAGCTATTGGACCAGCTATTGTTTCATCGATCTATCGT

AACAGAAAATCCATTCGTATTAATCAATCAAATTTGTTGAATAAATAGATGGAACATACA

TATCAATATGCATGGATAATACCTTTCCTTCCATTTCCAGTTACTATATCAATAGGGTTG

GGACTTCTGCTCGTTCCAACGGCAACGAAAAATCTTCGCCGTATTTGGGCTTTTTTTAGT

GTTTTATTGCTAAGTATAGCTATGGTGTTTTCGGCCGATCTGGCTATTCAGCAAATAAAC

GGGAGTTTTATCTATCAATATTCATGGTCTTGGACGATTAATAACACTTTTTCATTAGAG

TTTGGATACTTAATTGATCCACTTACCTCTATTATGTTAATACTAATCACTACTGTTGGA

ATCATGGTTCTTATTTATAGTGATAATTATATGTCTCATGATCAAGGATATTTAAGATTT

TTTGCTTATATGAGTTTTTTCAATACTTCTATGTTAGGATTAGTTACTAGTTCTAATTTG

ATACAAATTTATATTTTTTGGGAACTCGTAGGAATGTGCTCCTATTTATTAATAGGGTTT

TGGTTCACACGACCAACTGCAGCAAATGCTTGTCAAAAAGCGTTTGTAACTAATCGTGTA

GGGGATTTTGGTTTGTTATTAGGAATCTTAGGTCTTTATTGGATAACAGGTAGTTTCGAA

TTTCGGGATTTGTTCGAAATTTTAAAAAACTTGATC---C------ATAATAATGAAGTC

AATTCTTTATTTGCCGCCCTGTGCGCCTCACTATTATTTGTTGGTGCGGTTGCGAAATCC

GCACAATTTCCACTTCATGTATGGTTACCTGATGCTATGGAGGGCCCTACCCCCATTTCG

GCTCTTATACACGCTGCTACTATGGTAGCGGCGGGCATTTTTCTTGTAGCTAGGCTTCTT

CCTCTTTTCACAGTCATACCTTACATAATGAATTTCATTTCTTTGATAGGTATAATAACA

GTCCTTTTAGGGGCTACTTTAGCTCTTGCTCAAAGAGACATTAAAAGAAGTTTAGCCTAC

TCTACAATGTCTCAGTTAGGTTATATTATGTTAGCTCCAGGTATAGGCTCTTATCGAGCT

GCTTTATTCCATTTGATCACTCACGCCTATTCTAAAGCATTATTGTTTTTAGGATCTGGA

TCAATTATTCATTCAATGGAACCTATTGTTGGATATTCACCAGAGAAAAGTCAGAACATG

ATTCTTATGGGTGGTTTAAGAAGATATGTTCCAATTACAAAAACCACTTTTTTCTTAGGT

ACACTTTCTCTTTGTGGTATGCCCCCTCTTGCTTGTTTTTGGTCCAAAGATGAAATTCTT

AATGATACTTGGTTATATTCACCAATTTTCGCAATAATAGCTTGGTCCACAGCAGGATTA

ACTGCATTTTATATGTTTCGGGTATATCTCCTTACTTTTGATGGTCATTTACAAGTTCAT

TTTCAAAATTTCAGTAGTACTAAAAATAGCTCCTTCTATTCAA------TATCTATATGG

GGAAAAGAAGTACCGAAACCACTTAACGTAAAT----TTGTTTTTAT----CAAC-----

--------AAT--GAACACTAACGAAAAAA---TGTCTTTT------------TTTTCG-

-----AAAAATACATATCA----A--ATTGATAGAAATGGAAAAAACCGA----------

ATTCGTTACT---TTAGTACTCAGTTTGGAAATAAGTAC------ACTTCCATGTATCCC

CATGAATCGGACAATACTATGCTTTTTCCGATGCTTGTATTGGTCTTATTTACTTTGTTC

ATTGGATTTATAGGAATTCCTTTCGATCAA---------------GGAGTAATTGATTTG

GATATATTATCAAAATGGTTAACTCCATCGATA------AACCTTTTACATTCCAATTCC

GGCGATTCCTTTGATTGGTATGAATTTGTGACAAATGCAATTTATTCAGTCACTATATCC

TTTTTGGGGATATTTCTA-GCATACATATTTTATGGGTCTGTTTATTCATCTTTTCAAAA

TTTGGACTTAATCAATTCGTTTGTTCGAATAGATTCTAA---GAGAAT------TTTATC

GGATCGAATAATAAATGGGATATACAATTGGTCATATAATCGTGGTTACATCGACGTTTT

TTATGGAAAAGTTTTAAGTAATACTATAAGAGGACTTGCAGAATTAATTCATTTTTTTGA

TAGACGAGTTATTGATGGGATTACAAATGGGGTTGGTGTTGTAAGTTTCTTTGTAGGAGA

AGGGATAAAATCT---GTAGGGGGTGGACGAATCTCATCTTATATCTTCTTGTATGCATT

TTCTGTATCAATTTGT----------TTAATAA---------TTTATTATT---TTTTCA

GGTTCTAA--------------------------------------ATGGATTTACCTGG

ACCAATACATGATGTTCTTTTAGTATTTCTGGGATCAGGTCTTATATTAGGAGGTTTAGG

AGTGGTATTACTTACCAATCCAATTTATTCTGCCTTTTCATTGGGATTGG---TTCTTGT

TTGTATATCCTT-ATTCTATATTCTATCGAACTCCTATTTTGTAGCTGCTGCACAGCTCC

TTATTTACGTGGGAGCCGTAAATGTCTTAATCATATTTGCTGTGATGTTCATGAATGGTT

CAGACTATTCCAACGATTTCTATCTTTGGACCGTTGGGGATGGAGTCACTTCACTGGTTT

GTACAAG---------TATTCTTTTTTCACTAATTACT-ACTATCCTAGATACGTCATGG

TACGGAATTATTTGGAATACAGGATCGAACCAGATTGTAGAGCAGGACTTAACAAGTAAC

GTTCAACAAATTGGAATTCATTTATCAACAGATTTTTATCTTCCATTTGAACTCGTTTCA

ATAATTCTTTTAGTTGCCTTGATAGGTGCAATTACTATGGCGCGTCAGTAC---------

TAA---------------------ATGACTGTACCAGCTACAAG------ACAAGACCTT

ATGATAGTTAATATGGGTCCTCACCACCCATCAATGCACGGTGTTCTTCGCCTGATCGTT

ACTCTCGACGGTGAAGATGTTATTGACTGTGAACCCATATTGGGTTATTTACATAGAGGG

ATGGAAAAAATTGCAGAAAACCGAACAATTATACAATATCTGCCTTATGTAACACGTTGG

GATTATTTAGCTACTATGTTCACAGAAGCAATAACAGTAAATGGGCCCGAACAATTGGGA

AATATTCAAATACCTAAAAGAGCCAGCTACATCAGAGTAATTATGTTAGAGCTGAGTCGG

ATAGCTTCTCATTTGTTATGGCTTGGTCCATTTATGGCGGATATCGGTGCACAAACACCC

TTTTTTTATATTTTCAGAGAGAGGGAATTGATATATGATCTATTCGAAGCTGCTACAGGT

ATGCGAATGATGCATAATTATTTCCGTATAGGAGGAGTTGCTGCTGATCTACCTTATGGC

TGGATAGATAAATGTTTAGATTTCTGCGATTATTTTTTAACAGGAATTGTTGAATATGAA

AAGCTTATTACACAGAATCCTATTTTTTTGGAACGAGTTGAAAGAGTGGGCATTATTAGT

GGGGAGGAAGCCATAAATTGGGGTTTATCAGGACCCATGTTACGAGCTTCCGGAATAGAA

TGGGATCTTCGTAAAGTTGATAATTA--TGAGTGTTATAATGAATTTGATTGGGAAGTCC

AATGGCAAAAAGAAGGAGATTCATTAGCTCGTTATTTAGTCCGAATTAGTGAAATGAAAG

AATCCATAAAAATTATTCAACAGGCTCTAGAAGGAATCCCTGGGGGGCCCTATGAAAATT

TAGAGGTTCGGCGC------TTTGATAAAGTCAAGGATTCGGAAT----GGAATGATTTT

GAATATAGGTTTATTAGTAA--AAAGC-------CTTCGCCTACTTTTGAATTGGCGAAA

CAAGA---ACTTTATGTGAGAGTAGAAGCACCAAAGGGAGAATTAGGAATTTTTCTGATA

GGAGATAATAGTGTTTTCCCCTGGAGATGGAAAATTCGTCCACCCGGTTTCAT-------

---CAATTTGCAAATTCTTCCTCAACTAGTTAAAAGAATGAAATTGGCCGATATCATGAC

GATACTAGGTAGTATAGATATCATTATGGGAGAAGTTGATCGTTGAATGTTCCCTATGGT

GACTGGGTTCATGAATTATGGTCAACAAACAGTACGAGCTGCAAGGTACATTGGTCAAAG

TTTCATGATCACCTTATCGCACGCGAATCGTTTACCTGTAACTATTCAATATCCTTATGA

AAAATTGATCACATCAGAGCGTTTTCGTGGTCGAATCCACTTTGAATTTGATAAATGTAT

CGCTTGTGAAGTATGTGTTCGTGTATGCCCTATAGATCTACCCGTTGTTGATTGGAGATT

GGACACAGATGTTAGAAAA---AAACAATTGCTTAATTATAGTATTGATTTTGGAGTCTG

TATATTTTGTGGCAACTGCGTCGAGTATTGTCCAACAAACTGTTTATCCATGACTGAAGA

ATATGAACTTTCTACTTATGATCGTCACGAATTGAATTATAATCAAATTGCTTTGGGGCG

GTTACCGATGTCAGTAATTGAGGATTACACAATTCGAACAACTACAAATT------TGAC

TCCAATT---AAAATAGCTAAAAATAAACCCCTG---GGTTCACGAACGATTACTAATTA

A---------------AT------------------------------------------

---GCAGGGTCGTTTATCTGCTTGGCTAGTCAAGCATGAGCTAGTTCATAGATCTTTGGG

CTTCGATTACCAAGGAATAGAGATTTTACAAATAAAACCCGAGGATTGGGATTCCATTGC

TGTCATTTCATACGTATATGGTTACAATTATTTACGTTCCCAGTGTGCCTATGATGTAGC

ACCCGGCGGATTTTTAGCCAGTGTCTATCATCTTACGAGAATACAGTATGGTGTGGATCA

ACCGGAAGAGGTATGCATAAAAGTATTTGCTCCAAGGAGGAATCCTAAAATCCCGTCTGT

TTTCTGGATTTGGAGAAGTGCTGATTTTCAAGAACGGGAATCTTATGATATGTTGGGAAT

TTCTTATGAAAATCATCCGCGCCTTAAACGTATTTTGATGCCTGAAAGTTGGATAGGCTG

GCCCTTACGTAAGGATTATATTGCCCCCAATTTCTATGAAATTCAAGATGCTCATTGA--

------------------------------------------------------------

-------------------------------------------------ATGGTCTTAGC

TCCTGAATATTCGGACAATC------AAAAAGAAGGAAAAGATTACATCGAGACAGTTAT

GAATTCTATTGAGTTTCCGTTACTTGACCGAACAACCCCAAATTCAGTTATTTCAACTAC

ATTGAATGATCTTTCGAACTGGTCAAGACTATCCAGTTTATGGCCGCTTCTCTACGGTAC

CAGTTGTTGCTTCATCGAATTTGCTTCATTAATAGGCTCGCGATTCGACTTTGATCGTTA

TGGATTGGTACCAAGATCGAGTCCTA-GGCAAGCGGACCTAATTTTAACAGCTGGCACAG

TAACAATGAAAATGGCTCCCTCTTTAGTGAGATTGTATGAGCAAATGCCTGAACCAAAAT

ATGTCATTGCTATGGGAGCTTGCACTATTACCGGAGGGATGTTCAGTACCGATTCTTATA

GTACCGTTCGGGGAGTCGATAAGCTAATTCCTGTCGATGTCTATTTGCCGGGCTGCCCCC

CTAAGCCAGAGGCAGTTATAGATGCTATAACAAAACTTCGTAAGAAGTTATCTCGAGAAA

TCTCTGAAGATAGAATGGGGT------CTCAACGTGAAAATCGATGTT----------TT

ACGACCAATCACAAATTTTATGTTCGACGCAGTACTCATACTGGAAATTACGATCAAGGA

TTACTCTATCAATCGCC---------------ACCTACTGCAGAAATACCTTCGGAAA--

----------------------CAGAACCCTTTTTCAAATACAAAAGTTCAGTATCTTCC

CGCGAATTAGGGAATGAATCAGGCAAGGAAGATGTTTCTATACAGAATAAATAA------

---------------------------------ATGC---AAAATCGAAATACCTTTTCT

TGGGTAAAGGAACAGATGACTCGATTCATTTCAGTATCGATCATGATATATGTAATAACT

CGGACATCTATTGCAAATGCATATCCCATTTTTGCACAACAGGGTTATGAAAATCCACGA

GAAGCAACTGGACGCATTGTATGTGCTAATTGCCATTTGGCGAATAAGCCCGTGGATATT

GAAGTTCCACAAGCCGTGCTTCCTGATACTGTATTTGAAGCAGTTGTTAGAATCCCTTAT

GATAAGCAACTGAAACAAGTTCTTGCTAATGGTAAAAAGGGGTCTTTGAATGTAGGGGCT

GTTCTTATTTTACCCGAGGGATTTGAATTAGCCCCCCCCGATCGTATTTCTCCAGAAATG

AAAGAAAAGATG---GGGAATCTGGCTTTTCAGAGTTATCGCCCTACTAAAAAAAATATT

ATTGTGATAGGTCCTGTTCCGGGTCAGAAATATAGTGAAATCGTCTTTCCTATTCTTTCC

CCCGACCCTGCTACAAAGAAAGATGTTCACTTCTTAAAATATCCCATATATGTGGGCGGG

AACAGGGGAAGGGGTCAGATTTATCCCGATGGGAGCAAGAGTAACAATACAGTCTATAAT

GCTACAGCCGCTGGTGTGGTAAGCAGAATCCTACGTAAGGAAAAG---GGGGGTTATGAA

ATAACCATAGCTGATGCATCGGATGGACATCAAGTGGTTGATATTATACCTCCAGGACCA

GAACTTCTTGTTTCGGAGGGTGAATCCATTAAGCTTGATCAACCATTAACGAGTAATCCC

AATGTGGGCGGATTCGGGCAGGGCG-ATGCA--GAAATAGTACTTCAAG-ATCCATTACG

CG-TCCAAGGTCTTTTGTTCTTCTTGGCATCTGTTATTTTGGCACAAATCTTTTTGGTTC

TTAAAAA-GAAACAGTTTGAAAAGGTTCAATTGTACG-AAATGAATTTTTAGATGAGTGT

GTGACTTGTTATAA-TTGATCTTATTG-ATAGTACAGAGAATGGATCTGTCATC---TCG

ATAGAGATGATTCT-ATCTCGTCGGATA-------TTCAT----TCTAGTATCTGGAGCA

CGG-----AATATATAGA---------ATAGATC-----AAGAAATATTTGAACTATGAT

TCATACCTACTGT-------TCAAACCTCGCGACCGGACTCAAAAAAAAATTATCCAAGG

GA-------TA-----------TTTTGAAAATCAAACGAT---TTTTCTTCCTTCAGAAT

TGTGCTTA-TTTATTTTGGCTGAAGGACAGT--AGTTTTTTATTTGGATTT---TGGGTC

ATT----ACA-TCCATTCATCA-AAT-----AGGTGATAATCAAAG-----GGTTCTT-A

CTCAGAGAACCTTTGGGCTTAGCTTGGAATTT------ATCGAATCATCGTGGTTATAGT

ATGAATCTGAGGTTACAAT-----CGATTCGTGGGGTCTGAACAAGAGAATTCCTATTAA

TCAAT--------------------------------------------ATGTAAATCTG

CATTACGCACAAACAAAAAAAAAATCCAAT----------AAATAGGAAAATAGAAGATT

CAAGAGGCC----T-GTAACGCG--------ATCAACAT---------AAAGAAAGGCGG

ATGAGCTAACTTGA------------------TATTTTGGCATTAT--------CATCTC

AAAGAAGAGATTCCGGATATTGGTTA--CTTGTTCGGATCTTCACAGGGAAGGGAAGATT

GAATCAA-----GTGGCTAA-------GAGGTTTCAAACTTTCTATTCTAT---------

ATCCGTTG-CAACCACTATTTG------GTTGGTTCCGCTTGAGCCGTACGAGATGAAAT

TCTCATATCCGGTTCTTAGAGGGGGAAGTCTTCTCGGTTTACCTATCTCAATAAAGTATA

TGATTGGTTCGAGGAACGTCTCGAGATTCAGGCGATTGCAGATGATATAACTAGTAAATA

TGTTCCTCCTCATGTCAACATATTTTATTGTCTAGGAGGGATCACACTTACTTGTTTTTT

AGTACAAGTAGCTACGGGCTTTGCTATGACTTTTTACTATCGTCCAACTGTTACAGATGC

TTTTGCATCTGTTCAATATATAATGACTGAGGTCAACTTTGGTTGGTTAATACGATCAGT

TCATCGATGGTCAGCAAGTATGATGGTACTAATGATGATCCTGCACGTATTTCGTGTGTA

TCTTACAGGTGGATTTAAAAAACCCCGTGAATTGACTTGGGTTACAGGTGTGGTTCTGGG

TGTATTGACTGCATCTTTTGGTGTAACTGGTTATTCTTTACCTTGGGACCAAATCGGTTA

TTGGGCAGTAAAAATTGTTACAGGCGTACCTGACGCTATTCCTGTAATAGGATCTCCTTT

GGTAGAGTTATTACGTGGAAGTGCTAGTGTGGGTCAATCCACTTTGACTCGTTTTTATAG

TTTACACACTTTTGTCTTACCTCTTCTTACTGCCGTATTTATGTTAATGCACTTCCCAAT

GATACGTAAGCAAGGGATTTCAGGTCCTTTATAGATGGGAG-------------------

------------------------------------------------------------

------------------------------------------------------------

------------------------------------------------------------

------------------------------------------------------------

------------------------------------------------------------

------------------------------------------------------------

------------------------------------------------------------

------------------------------------------------------------

------------------------------------------------------------

------------------------------------------------------------

------------------------------------------------------------

------------------------------------------------------------

------------------------------------------------------------

------------------------------------------------------------

------------------------------------------------------------

-----------------------------------------TAACAAAGAAACCTGACTT

GAATGATCCTGTATTAAGAGCTAAGTTGGCTAAAGGGATGGGACATAATTATTATGGAGA

ACCCGCGTGGCCCAACGACCTTTTATATATCTTTCCAGTAGTAATTTTAGGTACTATTGC

GTGTAATGTGGGCCTGGCGATTCTAGAACCATCAATGATTGGTGAACCCGCGGATCCATT

TGCAACTCCTTTGGAAATATTACCCGAATGGTACTTTTTTCCCGTATT------------

------------TCAAATACTCCGTACAGTTCCCAATAAGTTACTGGGTGTTCTTTTAAT

GGCTTCAGTACCGCTGGGATTATTTACAGTACCCTTTTTGGAGAATGTTAATAAATTCCA

AAATCCCTTTCGTCGTCCAGTAGCTACAACAGTCTTTTTGATCGGTACTGCAGTAGCCCT

TTGGTTAGGTATTGGAGCCACATTACCTATTGATAAATCCCTAACTTTA-GGTCTTTTTT

AA----------------------------------------------------------

-----ATGATTGAAGTTTTTCTATTTGGAATCGTATTAGGTCTAATTCCTATTACTTTGG

CAGGATTGTTCGTAACTGCATATTTACAATACAGACGCGGTGATCAGTTGGACCTTTGA-

--ATGCCTACTATAACTAGTTATTTCGGTTTTCTACTGGCTGCTTCAACTATAACCACAG

CTCTATTCATTGGTCTGAGCAAGATACGGCTAATTTGAATGGATATTGTAAGTCTTGCTT

GGGCTGCTTTAATGGTAGTCTTTACATTTTCCCTTTCACTCGTAGTATGGGGAAGAAGTG

GACTCTAGATGATTATTCGTTCGCCGGAACCAGAAGTGAAAATTGTGGTAGATAGGGATC

CCATAAAAACTTCTTTCGAGGCATGGGCCAGACCCGGCCATTTTTCAAGAACAATAGCTA

A---AGGCCCTGATACTACCACTTGGATCTGGAACCTACATGCTGATGCTCACGATTTCG

ATAGCCATACCAGTGATTTGGAGGAGATTTCTCGAAAAGTATTTAGTGCTCATTTCGGTC

AACTCTCCATCATCTTTCTTTGGCTGAGTGGCATGTACTTCCATGGCGCCCGTTTTTCTA

ATTATGAAGCATGGCTGAGTGATCCTACTCACATTAGACCCAGTGCCCAGGTAGTTTGGC

CAATAGTGGGTCAAGAAATTTTGAATGGTGATGTGGGCGGGGGTTTCCGCGGAATACAAA

TAACCTCTGGTTTTTTTCAAATTTGGCGAGCATCTGGAATAACTAGTGAATTACAACTTT

ATTGTACCGCAATTGGCGCGTTGGTCTTTGCAGCGTTAATGCTTTTTGCAGGTTGGTTCC

ATTATCACAAAGCTGCTCCAAAATTGGCTTGGTTCCAAGATGTCGAATCTATGTTGAATC

ACCACTTAGCGGGGTTACTAGGACTGGGGTCTCTTTCTTGGGCGGGACACCAAATACATG

TATCTTTGCCGATTAACCAATTTCTCGACGCGGGAGTGGATCCTAAAGAGATACCACTTC

CTCATGAATTTATATTGAATCGGGATCTTTTGGCTCAACTTTATCCAAGTTTTGCCGAAG

GAGCAACTCCATTTTTCACCTTGAATTGGTCAAAATATGCAGAATTTCTTACTTTTCGGG

GAGGATTAGATCCTATAACAGGCGGCCTATGGTTGAGCGATATTGCGCACCATCATTTAG

CTATTGCAATTCTTTTCCTGATAGCTGGTCATATGTATAGAACCAACTGGGGCATTGGTC

ATGGACTAAAAGATATTTTGGAGGCTCATAAGGGTCCATTTACGGGCCAAGGTCATAAAG

GCCTTTATGAAATCC-TAACCACG--TCATGGCATGCTCAATTAGCTCTTAACCTGGCTA

TGTTAGGCTCTTTAACCATTGTTGTAGCTCACCATATGTATTCCATGCCCCCCTATCCAT

ACCTAGCTACTGACTATGGTACACAACTTTCGTTGTTCACACATCACATGTGGATCGGTG

GATTTCTCATAGTTGGTGCTGCTGCACATGCAGCCATTTTTATGGTAAGAGACTACGATC

CAACTACTCGATACAACGATCTATTAGATCGTGTCCTTAGGCACCGTGATGCAATCATAT

CACATCTAAACTGGGCATGTATATTTCTAGGCTTTCACAGTTTTGGTTTGTATATTCATA

ATGATACCATGAGTGCCTTAGGGCGCCCCCAGGATATGTTTTCAGATACCGCTATACAAT

TACAACCCATCTTTGCTCAATGGGTACAAAACACCCATGCTTTAGCACCTGTTGCGACAG

CTCCTGGTGCAACAACAGGCACCAGCTTAGCTTGGGGGGGTGGTGAGTTAGTAGCAGTAG

GCGGCAAAGTAGCTTTGTTACCTATTCCATTAGGAACTGCGGATTTCTTGGTACATCACA

TTCATGCATTTACGATCCATGTGACTGTATTGATACTACTGAAAGGTGTTCTGTTTGCTC

GGAGCTCCCGTTTAATACCTGATAAAGCGAATCTTGGCTTTCGTTTCCCTTGTGATGGTC

CTGGAAGAGGAGGAACATGTCAAGTATCCGCCTGGGATCATGTCTTCTTAGGTCTATTCT

GGATGTACAATTCAATTTCGGTAGTCATATTCCATTTCAGTTGGAAAATGCAATCGGATG

TTTGGGGTAGTATAAGCGATCAAGGGGTGGTAACTCATATCACAGGAGGAAACTTTGCAC

AGAGTTCCATTACTATTAATGGGTGGCTTCGGGATTTCTTATGGGCACAGGCATCTCAGG

TAATTCAGTCTTATGGTTCTTCATTATCTGCATATGGTCTTTTTTTCCTAGGTGCTCATT

TTGTATGGGCTTTCAGTTTAATGTTTCTATTCAGCGGCCGTGGTTATTGGCAAGAACTTA

TTGAATCCATCGTTTGGGCTCATAACAAATTAAAAGTTGCTCCTGCTACTCAGCCTAGAG

CCTTGAGCATTGTACAAGGACGTGCTGTAGGAGTAACTCATTACCTTCTGGGTGGAATTG

CCACAACATGGGCATTCTTCTTAGCAAGAATTATTGCAGTAGGATAAATGGCATTAAGAT

TTCCAAGGTTTAGCCAAGGCTTAGCTCAGGACCCCACTACTCGTCGTATTTGGTTTGGTA

TTGCTACCGCACATGACTTCGAGAGTCATGATGATATTACTGAGGAACGTCTTTATCAGA

ACATTTTTGCTTCTCACTTTGGTCAGTTAGCAATAATCTTTCTGTGGACTTCCGGAAATC

TGTTTCATGTAGCTTGGCAAGGAAATTTTGAGTCATGGGTACAGGACCCTTTACACGTAA

GACCTATTGCTCACGCAATTTGGGATCCTCATTTTGGTCAACCGGCTGTCGAAGCCTTTA

CTCGAGGAGGTGCTCCTGGACCAGTGAATATCGCTTATTCCGGCGTTTATCAGTGGTGGT

ATACAATCGGATTACGCACCAATGAAGATCTTTATACTGGAGCTCTTTTTCTATTATT--

----TCTTTCTGCTATATCCTTAATAGGGGGTTGGTTACACCTACAACCCAAATGGAAAC

CAAGCGTTTCTTGGTTCAAAAATGCGGAATCCCGTCTGAATCATCATTTGTCAGGACTTT

TCGGAGTAAGTTCCTTGGCTTGGACAGGGCATTTAGTTCATGTCGCTATTCCCGCATCA-

--AGGGGCGAGTATGTCAGATGGAATAATTTCTTAGATGTATTACCGCACCCCCAAGGTT

TGGGACCACTTTTTAGCGGTCAGTGGAATCTTTATGCCCAAAACCCCGATTCCAGTAGTC

ATTTATTCGGTACCTCCCAAGGAGCGGGAACTGCCATTCTAACTCTTCTCGGGGGATTCC

ATCCGCAAACGCAAAGTTTATGGCTGACCGATATGGCTCATCATCATTTAGCTATTGCAT

TTATTTTCCTGATAGCTGGT-CATATGTATAGAACCAACTTCGGGATTGGGCACAGTATA

AAAGATCTTTTAGAAGCACATACTCCTCCGGGGGGTCGATTGGGGCGTGGGCATAAGGGT

CTTTATGATACAATCAATAATTCGCTTCATTTTCAATTAGGTCTTGCTCTGGCCTGTTTA

GGAGTTATTACTTCCTTGGTAGCTCAACACATGTACTCTTTACCTGCTTACGCATTCATA

GCGCAAGACTTTACTACTCAAGCTGCGTCGTATACTCATCACCAATACATCGCAGGTTTC

ATCATGACGGGAGCCTTTGCTCACGGAGCTATATTCTTCATTAGAGATTACAACCCGGAA

CAGAATGAAGATAATGTATTGGCGAGAATGTTAGACCATAAAGAAGCTATCATATCTCAT

TTAAGTTGGGCCAGTCTGTTTTTGGGGTTCCATACCTTGGGACTTTATGTTCATAACGAC

GTCATGCTCGCTTTTGGGACTCCGGAAAAACAAATCTTGATTGAACCTATATTTGCCCAG

TGGATACAATCCGCTCATGGTAAGACTTTATATGGGTTTGATGTACTCTTATCTTCAACG

AGTGGCCCAGCATTCAATGCTGGTCGAAGCATTTGGTTACCCGGTTGGTTGAGTGCTGTT

AATGAGAATAGTAATTCACTATTCTTAACAATAGGTCCTGGAGACTTCTTGGTTCA-TCA

TGCTATTGCTCTAGGTTTGCATACAACTACATTGATCTTAGTAAAGGGTGCTTTAGATGC

GCGTGGTTCGAAGTTAATGCCAGATAAAAAAGATTTCGGTTATAGTTTTCCTTGCGACGG

CCCGGGACGGGGGGGTACTTGTGATATTTC-TGCTT--GGGATGCATTTTATTTGGCGGT

TTT-CTGGATGTTAAATACCATTGGATG-GGTTACTTTTT-ATTGGCATTGGAAGCACAT

CACATTATGGCAAGGTAATGTTTCACAATTTAACGAATCCTCTACTTATTTGATGGGATG

GTTAAGAGATTATCTATGGTTAAACTCTTCCCAACTTATCAACGGATACAATCCGTTTGG

TATGAATAGTTTATCGGTCTGGGCATGGATGTTCTTATTTGGACATCTTGTTTGGGCTAC

TGGATTTATGTTCTTAATTTCCTGGCGTGGATATTGGCAGGGATTAATTGAAACTTTAGC

ATGGGCTCATGAACGCACACCTTTGGCTAATTTGATTCGATGGAGAGATAAGCCAGTGGC

TCTTTCCATCGTGCAAGCAAGATTGGTTGGATTAGCCCACTTTTCCGTAGGTTATATATT

CACTTATGCAGCTTTCTTGATTGCCTCTACATCAGGTAAATTTGGTTAAATGTCACATTC

AGTAAAGATTTATGATACATGTATAGGGTGTACTCAATGTGTACGAGCCTGTCCTACAGA

TGTATTGGAAATGATACCCTGGGACGGATGTAAAGCTAAGCAAATTGCTTCTGCGCCAAG

AACAGAGGACTGTGTAGGTTGTAAGAGATGCGAATCCGCCTGTCCAACAGACTTCTTGAG

TGTCCGGGTTTATTTAGGGAATGAGACAACTCGCAGCATGGGTCTACCTTATTGAATGAC

AGATCTCAA---------------------------------------------------

---------CTTACCCTCTATTTTTGTGCCTTTAGTAGGCCTAGTCTTTCCGGCAATTGC

AATGGCTTCTTTATTTCTTCATGTTCAAAAAAACAAGA----------------------

-------------------------------------------TTGTCTAAATGCAAGAT

ATAAAAACATATCTCTCTGTGGCACCTGTGCTAACTACTCTATGGTTTGGGTCTTTAGCA

GGTTTATTGATAGAGATCAATCGTTTATTCCCGGATG------CCTTGTCATTCCCATTT

TTT------------------TAAATGACTGCAATTTTAGAGAGACGCGAAAGTACAAGC

CTATGGGGTCGCTTCTGTAACTGGATAACCAGCACCGAAAACCGCCTTTACATTGGATGG

TTCGGTGTTTTAATGATCCCTACCTTATTGACCGCAACTTCTGTATTTATTATCGCCTTC

ATCGCTGCTCCTCCAGTAGATATTGATGGTATCCGTGAACCTGTTTCCGGGTCTTTACTT

TATGGAAACAACATTATTTCTGGTGCCATTATTCCTACATCTGCAGCTATAGGTTTGCAC

TTTTACCCAATTTGGGAAGCAGCATCCGTTGATGAGTGGTTATACAATGGTGGTCCTTAT

GAACTAATTGTTCTACACTTCTTACTTGGTGTAGCTTGTTATATGGGTCGTGAGTGGGAA

CTTAGTTTCCGTCTGGGTATGCGTCCTTGGATTGCTGTCGCATATTCAGCTCCTGTTGCA

GCTGCTACTGCTGTTTTCTTGATCTACCCTATTGGTCAAGGAAGCTTCTCTGATGGTATG

CCTCTAGGAATATCTGGTACTTTCAACTTTATGATTGTATTCCAGGCTGAACACAACATC

CTTATGCATCCATTCCACATGTTAGGCGTAGCTGGTGTATTCGGCGGCTCCCTATTTAGT

GCTATGCATGGTTCCTTGGTAACCTCTAGTTTAATCAGGGAAACTACTGAAAATGAATCT

GCTAATGAAGGTTACAGATTCGGTCAAGAGGAAGAAACTTATAATATCGTCGCTGCTCAC

GGTTATTTTGGCCGATTAATCTTCCAATATGCTAGTTTCAACAATTCCCGTTCCCTACAT

TTCTTCTTGGCTGCTTGGCCTGTAGTAGGTATCTGGTTCACTGCTTTAGGTATTAGTACT

ATGGCTTTCAACCTAAACGGTTTCAATTTCAACCAATCCGTAGTTGACAGTCAAGGTCGT

GTTATTAACACTTGGGCTGATATCATCAACCGTGCTAACCTTGGTATGGAAGTAATGCAT

GAACGTAATGCTCACAACTTCCCTCTAGACCTAGCTTCTGTTGAAGCACCTTCTACAAAT

GGCTAAATGGGTTTGCCTTGGTATCGTGTTCATACCGTCGTATTGAATGATCCCGGTCGG

TTGCTTTCTGTCCACATAATGCATACAGCTCTAGTTGCTGGTTGGGCCGGGTCAATGGCT

CTATACGAATTAGCGGTTTTTGATCCCTCTGACCCCGTTCTTGATCCAATGTGGAGACAA

GGTATGTTCGTTATACCCTTCATGACTCGTTTAGGAATAACCAATTCGTGGGGTGGTTGG

AGTATCACAGGAGGAACTATAACGAATCCGGGTATTTGGAGTTACGAAGGTGTGGCAGGG

GCACATATTGTGTTTTCTGGCTTGTGCTTTTTAGCAGCTATTTGGCATTGGGTATATTGG

GACTTAGAAATATTCTGTGATGAACGTACGGGAAAACCCTCTTTGGATTTGCCCAAGATC

TTTGGAATTCATTTATTTCTCTCAGGAGTGGCTTGCTTTGGCTTTGGCGCATTTCATGTA

ACAGGCTTGTATGGTCCTGGAATATGGGTGTCTGATCCTTATGGACTAACCGGAAAAGTA

CAACCCGTAAATCCAGCTTGGGGCGCGGAAGGTTTTGATCCTTTT-GTTCCGGGC--GGA

ATAGCCTCTCATCATATTGCAGCGGGGACTTTGGGCATACTGGCAGGCCTATTCCACCTT

AGTGTCCGTCCACCCCAGCGTCTATACAAAGGATTACGTATGGGCAATATTGAAACTGTA

CTTTCGAGTAGTATCGCTGCTGTGTTTTTTGCAGCTTTCGTAGTTGCGGGAACTATGTGG

TATGGTTCAGCAACTACCCCCATCGAATTATTTGGTCCGACGCGTTACCAGTGGGATCAA

GGATACTTCCAGCAAGAAATATATCGAAGAGTTGGTGCTGGACTAGCCGAAAATCTGAGT

TTATCGGAAGCTTGGTCTAAAATTCCTGAAAAATTAGCTTTTTATGATTACATCGGTAAT

AATCCGGCAAAAGGTGGATTATTTAGAGCAGGCTCAATGGACAACGGAGATGGAATAGCC

GTTGGATGGTTAGGACACCCCGTCTTTCGAGATAAGGAAGGGCGTGAACTTTTTGTACGC

CGTATGCCTACTTTTTTTGAAACATTTCCGGTAGTTTTGGTAGATGGAGACGGAATTGTG

CGAGCCGATGTTCCTTTTCGAAGGGCAGAATCCAAATATAGTGTCGAACAAGTAGGCGTA

ACTGTTGAGTTCTATGGTGGCGAACTCAATGGAGTCAGTTATAGCGATCCTGCTACTGTT

AAAAAATATGCTAGACGTGCCCAATTAGGTGAAATTTTTGAATTAGATCGCGCTACTTTA

AAATCCGATGGTGTTTTTCGTAGCAGTCCGAGGGGTTGGTTCACTTTTGGACATGCTTCA

TTTG-CTTTACTCTTC--TTTTTCGGGCACATTTGGCATGGAGCTAGAACCTTGTTCAGA

GATGTTTTTGCTGGTATTGATCCAGATTTGGATGCTCAAGTGGAATTTGGGGCATTCCAA

AAAATTGGAGATCCAACTACAAGGCGACAAGCAGTTTGAATGAAAACCTTATATTCCCTG

AGGAGGTTCTACCCCGTGGAAACGCTCTTTAATGGAACTTTAGCTTTAGCTGGTCGTGAC

CAAGAAACCACTGGTTTCGCTTGGTGGGCCGGGAATGCCAGACTTATCAATTTGTCCGGT

AAACTACTCGGGGCTCACGTAGCCCATGCCGGATTAATCGTATTCTGGGCAGGAGCAATG

AACCTCTTTGAAGTGGCTCATTTCGTACCAGAGAAGCCCATGTATGAACAAGGATTAATT

TTACTTCCCCATCTAGCTACTCTAGGTTGGGGGGTAGGTCCGGGTGGGGA-AGTTATAGA

CACCTTTCCATACTTTGTATCTGGAGTACTTCATCTAATTTCCTCTGCAGTCTTAGGCTT

TGGCGGTATTTATCATGCGCTTCTTGGACCTGAGACTCTAGAAGAATCCTTTCCGTTCTT

CGGTTATGTATGGAAAGATAGAAATAAAATGACCACAATTTTAGGTATTCATCTAATTTT

GTTAGGGATAGGTGCTTTTCTTCTAG---TATTCAAGGCTCTTTATTTTGGGGGTGTATA

TGACACCTGGGCTCCTGGGGGGG---GAGATGTAAGAAAAATAACCAACTTAACACTTAG

CCCAAGTGTTATATTTGGTTATTTACTAAAATCTCCCTTTGGAGGAGAAGGATGGATTGT

TAGTGTGGACGATTTAGAAGATATAATTGGAGGACATGTATGGTTAGGTTCCATTTGTAT

ACTTGGTGGAATCTGGCATATCTTGACCAAACCCTTTGCATGGGCTCGCCGCGCATTTGT

ATGGTCTGGAGAGGCTTACTTGTCTTATAGTTTAGGTGCTTTAGCTGTCTTTGGGTTCAT

CGCTTGTTGTTTCGTCTGGTTCAATAATACCGCCTATCCTAGTGAGTTTTATGGACCTAC

TGGGCCGGAGGCTTCTCAAGCTCAAGCATTTACTTTTCTAGTTAGAGACCAACGTCTTGG

GGCTAACGTGGGATCCGCTCAGGGACCTACTGGTTTAGGTAAATATCTAATGCGGTCCCC

AACGGGAGAGGTCATTTTTGGAGGAGAAACCATGCGCTTTTGGGATCTCCGTGCTCCTTG

GTTGGAACCTCTAAGGGGTCCCAATGGTTTGGACTTGAGTAGGCTGAAAAAAGACATACA

ACCTTGGCAAGAACGACGTTCGGCGGAATATATGACTCATGCTCCTTTAGGGTCTTTAAA

TTCCGTGGGTGGTGTAGCTACCGAGATCAATGCAGTCAATTATGTCTCTCCTAGAAGTTG

GTTAGCTACTTCTCATTTTGTTCTAGGATTCTTCTTTTTTGTGGGTCATTTGTGGCACGC

AGGAAGAGCCCGCGCAGCGGCAGCAGGATTTGAAAAAGGAATCGATCGTGATTTAGAACC

TGTTCTTTCCATGACTCCTCTTAATTGAATGACTATAGCCCTTGGTAGATTTACCAAAGA

GGAAAATGATTTATTCGATATTATGGACGACTGGTTACGGAGAGACCGTTTCGTTTTTGT

AGGTTGGTCCGGTCTATTGCTCTTCCCTTGTGCTTATTTCGCTTTAGGAGGTTGGTTCAC

AGGTACAACTTTTGTAACTTCGTGGTATACCCACGGATTGGCTAGTTCATATTTAGAAGG

CTGCAATTTCTTAACCGCTGCAGTTTCTACTCCTGCTAATAGTTTAGCACATTCTTTGTT

GCTACTATGGGGTCCTGAAGCACAAGGCGATTTTACTCGTTGGTGTCAATTAGGCGGTCT

GTGGACTTTTGTTGCTCTACACGGTGCTTTCGGACTAATCGGTTTCATGTTACGTCAATT

CGAACTTGCTCGATCTGTTCAATTGCGACCTTATAATGCAATCGCATTTTCTGGTCCAAT

TGCTGTTTTCGTTTCTGTATTCCTAATTTATCCACTGGGTCAGTCTGGTTGGTTCTTTGC

ACCCAGTTTTGGTGTGGCAGCTATATTTCGATTCATCCTCTTCTTCCAGGGGTTTCATAA

TTGGACCCTAAACCCATTTCATATGATGGGAGTTGCCGGAGTATTGGGTGCTGCTCTGCT

ATGCGCTATTCATGGTGCTACCGTAGAAAATACTTTATTTGAGGACGGTGACGGCGCAAA

TACATTCCGTGCTTTTAACCCAACGCAAGCCGAAGAGACTTATTCGATGGTCACTGCTAA

CCGATTTTGGTCCCAAATCTTTGGGGTTGCTTTTTCTAATAAACGTTGGTTACATTTCTT

TATGTTATTTGTACCAGTAACCGGTTTATGGATGAGTGCCCTTGGAGTAGTTGGTCTGGC

TCTGAACCTACGTGCCTATGACTTC-GTTTCCCAGGAAATCCGTGCCGCGGAAGATCCTG

AATTTGAGACTTTCTACACCAAAAATATTCTCTTAAATGAGGGTATTCGTGCTTGGATGG

CAGCTCAGGATCAGCCTCATGAAAACCTTATATTCCCTGAGGAGGTTCTACCCCGTGGAA

ACGCTCTTTAAATGTCTGGAAGCACGGGAGAACGCTCTTTTGCTGATATTATTACCAGTA

TTCGATACTGGGTTATTCATAGCATTACTATACCTTCCCTATTCATTGCGGGTTGGTTAT

TCGTCAGTACAGGTTTAGCTTACGATGTGTTTGGAAGTCCTCGTCCAAACGAGTATTTTA

CAGAAAGCCGGCAAGGGATTCCATTAATAACTGGCCGTTTTGATCCTTTGGACCAACTTG

ATGAATTTAGTAGATCCTTTTAGATGACCATAGATCGAACCTATCCAATTTTTACAGTAA

GATGGTTGGCTGTTCATGGACTAGCTGTACCTACCGTTTCTTTTTTGGGGTCAATATCAG

CAATGCAGTTCATCCAACGATAA---------ATGGCTACACAAACCGTTGAGGGTAGTT

CTAGATCTG------GGCCAAGACGAACAATTGTAGGGGATTTATTGAAACCATTGAATT

CAGAATATGGTAAAGTAGCCCCGGGATGGGGAACTACTCCTTTTATGGGAGTCGCAATGG

CCCTATTTGCGATATTCCTATGTATTATTTTGGAGATTTATAATTCTTCCGTTTTACTGG

ATGGAGTTTCACCGAAT------TAG------------------ATG-----------CT

TACTCTCAAACTCTTCGTTTACACAGTAGTGATATTTTTT---------------GTTTC

TCTATTTATCTTCGGATTCCTATCTAATGATCCAGGACGTAATCCTGGACGTGAGGAA--

----TAAATGGCTGATACTA---------------------CTGGA---AGGATTCCTCT

TTGGCT---GATAGGTACTGT-AGCTGGTAT-TCCTGT-GATCG-GTTCAGTGGGTGTTT

T-------------CTTTTACGGTTCA-TATTCCGGATTGGGCTCATCTCTGTAGATGCT

TAATA---TACTTAATTTGATCTGTATCTGTCTTAATTCTGCCC---CTTATTCGAGTA-

-----GTTTTTTCTGCGCTAAATTGCCCGAGGCTTATGCTGTTTTCAATCCAATTGTAGA

TTTTATGCCAGTCATACCTGTACTCTTTTTTCTCTTAGCCTTTGTTTGGCAAGCCGCTGT

AAGTTTTCGCTGAATGACACAAT---------CAAACCCAAACGAACAAAATGTTGAATT

GAATCGTACCAGTCTATACTGGGGGTTATTACTCA-TTTTTGTACTTGCTGTTTTATTTT

CCAATTATTTCTTCAATTGAATGGAAGTAAATATTCTCGCATTTATTGCTACTGCACTGT

TCATTCTAGTTCCGACTGCCTTTTTACTTATCATCTACGTAAAAACAGTCAGTCAAAATG

AT---------TAAATGGAAACAGCAACCTTAGTCGCCATCTTCATATCTGGTTTACTTG

TAAGCTTTACTGGGTATGCCTTATATACCGCTTTTGGGCAACCCTCTCAACAACTAAGAG

ATCCATTCGAGGAACACGGAGACTAG---------ATGGAAGCATTGGTTTATACATTCC

TGTTAGTCTCGACTCTAGGGATAATTTTTTTCGCTATCTTCTTTCGAGAACCGCCAAAGG

T---------TCCTACTAAAAAGATGAAATGAATGACTATTGCTTTTCAATTGGCTATTT

TTGCATTAATTGCGACTTCATCGATCTTATTGATTAGTGTACCCGTTGTATTTGCTTCTC

CTGATGGTTGGTCAAGTAACAAAAATGTTGTATTTTCCGGTACCTCATTATGGATTGGGT

TAGTCTTTCTGGTAGGTATCCTTAATTCGCTCATCTCTTGAATGAGTTGTAGGGAGGGAC

TTATGTCACCACAAACAGAGACTAAAGCAGGTGTTGGATTCAAAGCTGGTGTTAAAGATT

ACAAATTGACTTATTATACTCCTGAATATGAAACCAAAGATACTGATATCTTGGCAGCAT

TTCGAGTAACTCCTCAACCCGGAGTTCCACCCGAGGAAGCAGGGGCTGCGGTAGCTGCCG

AATCCTCTACTGGTACATGGACAACTGTGTGGACCGATGGACTTACCAGCCTTGATCGTT

ACAAAGGCCGATGCTACCACATCGAGCCCGTTGTTGGGGAGCAAAATCAATATATTGCTT

ATGTAGCTTATCCTTTAGACCTTTTTGAAGAAGGTTCTGTTACTAACATGTTTACTTCCA

TTGTGGGTAATGTATTTGGGTTCAAAGCCCTACGAGCTCTACGTTTGGAGGATCTTCGAA

TTCCCCCTGCTTATTCCAAAACTTTCCAAGGCCCGCCCCATGGAATCCAGGTTGAGAGAG

ATAAATTGAACAAGTATGGTCGTCCCCTATTGGGATGTACTATTAAACCAAAATTGGGGT

TATCCGCGAAGAACTACGGTAGAGCAGTTTATGAATGTCTCCGTGGTGGACTTGATTTTA

CCAAGGATGATGAGAACGTGAACTCACAGCCATTTATGCGTTGGAGAGACCGTTTCTTAT

TTTGTGCTGAAGCAATTTATAAAGCGCAAGCGGAAACAGGTGAAATCAAAGGACATTATT

TGAATGCTACTGCGGGTACATGTGAAGAAATGATGAGAAGGGCCCAATGTGCCAGAGAAT

TGGGAGTTCCTATCGTAATGCATGACTACTTAACAGGTGGATTCACTGCAAATACTAGCT

TGGCTATTTATTGCCGAAACAACGGCCTACTTCTTCACATCCATCGTGCAATGCATGCAG

TTATTGATAGACAGAAGAATCATGGTATGCACTTTCGTGTACTAGCTAAAGCGCTACGTA

TGTCTGGTGGAGATCACATTCACGCCGGTACAGTAGTAGGTAAACTGGAAGGGGAACGTG

AAATGACTTTAGGTTTTGTTGATTTACTACGTGATGATTATATTGAAAAAGACCGAAGTC

GTGGTATTTTTTTCACTCAAGATTGGGTCTCTATGCCAGGTGTTTTGCCCGTAGCTTCCG

GTGGTATTCACGTTTGGCATATGCCTGCCTTGACCGAGATCTTTGGGGATGATTCTGTAC

TACAGTTCGGTGGAGGAACTTTAGGACACCCTTGGGGAAATGCACCTGGTGCAGTAGCCA

ATCGGGTAGCTTTAGAAGCGTGTGTACAAGCTCGTAATGAGGGACGTGATCTTGCTCGCG

AAAGTACTCAAATTATCCGT-----------------------GAAGCTTGTA-------

-------AATGGAGCCCTGAGCTAGCCGCTGCTTGTGAAGTATGGAAGGAAATCAAATTT

GA---GTTCGAACCAGTAGATAAGCTAGATGTTAAAAAAAACTAAACGGCGATACATTTA

TACAAAACTTCTACCTCGAGCACACGCAATGGAGCCGTA---------GACAGTCAAGTG

AAATCCAATCCACGAAATAATTTGATCTATGGACAGCATCATTGTGGTAAAGGTCGTAAT

GCCAGAGGAATCATTACCGCAGGGCATAGAGGGGGAGGTCAT-AAGCGTCTATACCGTAA

AATCGATTTTCGACGGAATAAAAAAGACATATCTGGTAGAATCGTAACCATAGAATACGA

CCCTAATCGAAATGCATACATTTGTCTCATACACTATGGGGATGGTGAGAAGAGATATAT

TTTACATCCCAGAGGGGCTATAATTGGAGATACCATTGTTTCTGGTACAGAAGTTCCTAT

ATCAATGGGAAATGCCCTACCTTTGAGTGCGGTTTGAACTATTGATTTACGTAATTGGAA

GTAACCAATTAGGTTTACGACGAAACCTAGAAATCGATCACTGATCCAATTGGAGTACCT

CTACAGGATAGACCTCAACAGAAAACTGAAGAGGAACGGCAGCAAGTGATTGAGTTCAGT

AGTTCCTCATATAAAATTATTGACTCTAGAGATATGGTAATATGGAGAAGAC-----AAA

ATTGATTGTTTGAAGCACGGACAGAACCGGA-AGCGCCCCTTGTTTCAAAGAGAGGAGGA

CGGGTTATTCACATTTAATTTGATGGTCAGAGGCGAATTGAAAGCTAAGCAGTGGTAATT

AT-------------AAGGATCCCCCAGGGGAAAAATAG---------------------

----------AGATGTCTCCTACGTTACCCGTAATATG-----TGGAAGTATCGACGTAA

TTTCATAGAGTCATTCGGTC--TGAATGCTACATGAAGAACATAAGCCAGATGATGGAAC

GAGGAGACCTAGGATGTAGAAGATCATACCATGAGTGATTCGGCAGATTTGGATTC----

-CTATATATCCACTCCTGTGGTACTTCATC---ATATGATTCATATAAGATCCATCTGTC

TAGATATCATCATATACATCTAGAAAGCCGTATGCTTTGGAAGAAGCTTGTACAGTTTGG

GAAGGGGTTTTTATTGATCAAAAAGAAGAATCTACTTCAACCGATATGCCCTTAGGCACG

GCCATACATAACATAGAAATCACACTTGGAAAGGGTGGACAATTAGCTAGAGCAGCGGGT

GCTGTAGCGAAACTGATTGCAAAAGAGGGTAAATCGGCCACATTAAGATTACCATCCGGG

GAGGTCCGTTTGATATCCAAAAACTGCTCAGCAACAGTCGGACAAGTAGGTAATGTTGGG

GCGAACCAACAAAGTTTGGGTAGAGCCGGATCTAAGTGTTGGCTAGGTAAGCGTCCTGTA

GTAAGAGGAGTAGTTATGAACCCTGTGGACCATCCCCACGGGGGTGGTGAAGGGAGAGCC

CCAATTGGTAGAAAAAAACCCACAACCCCTTGGGGTTACCCTGCGCTTGGAAGAAGAAGT

AGGAAAAGGAATAAATATAGTGATCGTTTTATTCTTCGTCGCCG--TAAA----------

------TAGATGATTCAACCTCAGACCCTTTTAAATGTAGCGGATAACAGCGGGGCTCGA

GAATTGATGTGTATTCGAATCATAGGA---GCCAGTAATCGCCGATATGCTCATATTGGT

GACGTTATTGTTGCTGTAATCAAAGAAGCAGTACCCAATATGTCTCTAGAAAGATCAGAA

GTGGTCAGAGCTGTAATTGTACGTACATGTAAAGAACTCAAACGTGACAACGGTATGATA

ATACGATATGATGACAATGCAGCAGTTGTCATTGATCAAGAAGGGAATCCAAAAGGAACT

CGAGTTTTTGGTGCGATCGCTCGGGAATTGAGACATTTGAATTTCACTAAAATAGTTTCC

CTAGCTCCTGAGGTATTATAAATGCTTAGTGTGTGACTCGTT------AATTTTTCTTTA

GATT---------TATTAGGATTCCAAAAATG--------------------GACCGGAC

CGTTATG-----------------------------------------------------

-AACTAATAACTATAGAA-----------------CTAATAACCAACTTATTGCTTCG--

---TGTTATCGAGATA----CAAAAGAAGAATTGCTATATGA------------------

----------------------------------TGTAT-CAATCATATAGTTATAGCAA

CTGAAATACTATTTATTTCCCTAAAAGAAAAATCAATCTTA-----TTATG--GGTTATG

AAAAAATA------GGTATGTG--GATA-AATGAAAGGGTGAGAG-AAAGAGAGAAGGAG

AATCT---CA-------ATGATATATGATTCTAA--TA--TGTATGGTCTATGAATCACC

TCCTA-----AAAGGCAATGTAATAACGCATCA-ATTC----------------------

------------------------------------------------------------

------------------------------------------------------------

-------------------AATACCCATAGGAAAAATACAAATAA---------------

----TAAAGAGCCCCAAAGTCAATAGAGACTGAGAAGATTGACTCAGGA-----------

ACTTAGTTGGGAGCTCCATTGCAGAGTTCGGGCCTAGCCA-TGAAT---AGAGAAGCTAT

AGGAACGATGGAACCTGTGACTGCATAAGATTCTATTGAATGAAAACGAATTCTAATGAT

TCATTAGGTGGGATGGCGGAACGAACCAGG-AACCAATTGATTTATTCGTAGAGGT----

----CATACATACGCTACGAG-TGAAGCAG--------GAAAGAGTCAATATTCGCCCGC

GAAAGCCTTATTGA----------------------------------------------

-ATTCAATATTTTGGGCATT-------AAAGTAA-----------------AAAAAAAAA

CATT---------------------------------------------ATCCATAATAA

ATTAA-ATAGAACGAAGTT-----------------------AAATA-------------

------------ATGAGAAAGAAAAATCTATATCTAG-----------------------

ATCTTGTATATACAGCT---------------------------------TCCTACATAA

C------AGATTCAATA--------TCAAC------------------------------

------------------------------------------------------------

----------------------------AAATACCACGATTTTTTGTATTATTCAT----

TAAATACACCTGTAT-----CTGTAATATTTTTGAAT-----------------------

------------------------------------------------------------

-----------TCATATTCATTCGCGAGGAGCTGGATGAGAAGAAACTCTCATGTCCGGT

TCTGCAGTAGAGATGG-----AATTGAGAA--ATAACCATCAACTATAACCCCAAAAGAA

CCAGATTCCGTAAACAACATAGAGGAAGAATGAAGGGAATGTCTTAT---CGAGGCAGTC

ATATTTGTTTCGGAAGATACGCTCTTCAGGCACTTGAACCATCTTGGATCACATCTAGAC

AAATAGAAGCGGGGCGAAGGGCAATGACACGATATGCACGTCGTGGTGGAAAAATCTGGG

TACGTATATTTCCGGACAAACCCGTTACAATAAGACCCGCGGAAACACGTATGGGTTCGG

GGAAGGGATCCCCCGAATACTGGGTATCCGTTGTTAAACCGGGTCGAATACTTTATGAGA

TGGGTGGAGTATCAGAAACTGTAGCCAGAGCCGCTATTGAAATAGCCGCGTCCAAAATGC

CTATAAGAACGCAATTTATTA---TTGCGGGA------TAGATGACCAGAGTTAGGCGAG

GATATATAGCTCGGAGACGTCGAACAAAAATTCGTTTATTTGCAGCAACCTTTCGAGGGG

CTCATTCAAGACTTACTCGAGCTGCCACTCAACAGAAAATGAGAGCTTTGGTTTCTGCTC

ATCGAGATAGAGGCAAGCAAAAGAGAGATTTTCGTCGTTTGTGGATCACTCGGATAAATG

CGGTAACTCGTGAGAATGG---GGTATGCTATAGTTATAGTCGATTAATGCACAATCTGT

ATAAGAGGCAGTTGCTTCTTAATCGTAAAATACTTGCACAAATAGCTATATTAAATAAGA

ATTGTCTTCACATAATTTCCAATGA------G-ATCATC-AAATAA--------------

---------ATGATAAAGAAAAATTGGATTA--------CTACGGGTACGGAAG------

---------CACGGG---------------------------------CTTTAGCTC---

-------------------AAAATATATCTATGTCTGCTTACAAAGCGAGAAGAGTAATT

GATCAGATTCGTGGGCGTTCCTATGAGGAAACAATTATGATACTAGAACTGATGCCTTAT

CGAGCATCTTTTCCTATTTTAAAATTGGTTTACTCTGCAGCAGCAAATGCTATTAACAAT

ATGGGTTTGAGCGAAGCTGATTTATTCATTAGTAAAGCAGAAGTCAATGGAGGTACTTTC

GTGAAAAAGTTACGACCCCGAGCTCGGGGACGTAGTTATGCGATAAAAAGGCCCACTTGT

CATATAACAATTGTATTGAAGG-------ATAAAG--------ATAAATCGCTT------

---------------------TTAAATGAATAA------------------ATGGATGGA

ATCAAATATGCAGTATTTACCGAAAAAAGTATTCGGTTATTGGGGAACAATCAATATACT

TCTAATGTCGAATCAGGATCAACTAGGACAGAAATAAAGCATTGGGTCGAACTCTTCTTT

GGCGTCAAGGTAATAGCTATGAATAGTCATCGAC------TCCCGGGAAAGGGTAGAAGA

GTGGGACCTATTAAGGGACATACAATGCATTACAGACGTATGATCATTACGCTTCAACCG

GGTTATTCTATTCCACCTCT-TATAGAGAAAAGAACTTAAATGGCGGTTCCAAAGAAACG

TACTTCTATATCAAAAAAACGTATTCGTAAAAATTTTTGGAA--GAAAAAGGGATATTTT

G-CCGCGGTAAAAGCTTTTTCT-------TTAGCTAAATCAG------TTTCCACCGG--

----------ACAGTCAAAAAGTTTTT-TTGTGCGACAAACAA---GTAATAAATCCTTG

GAA------------------------------------------TAAATGGCCAAGGGT

AAAGATGCCAGATTAACAGTTATTTTGGAATGTACCA---GTTGTGTCCG------AAAT

GGTGTCAAT------AAGGAATCGCCGGGTATTTCCAGATATATTACTCAAAAAAATCGA

CACAATACATCGAGTCGATTAGAATTGAGAAAATATTGTCGCTATTGTTATAAGCATACG

ATTCACGGGGAAATAAAGAAA------TAGATGAAAATAAGAGCCTCTGTTCGTAAAATT

TGCGAAAAATGTCGACTAATCCGTAGGCGGGGACGAATTATAGTTATTTGTTATAATCCG

AAACATAAACAAAGACAGGGGTAAATGGTTCGAGAGGAAGTAGTAGGATCCACTCGGGCA

CTACAGTGGAAGTGTGTTGAATCAAGAGCAGACAGTAAGCGCCTTTATTATGGTCGTTTC

A-TTCTGTCC--CCACTTCTGAAAGGACAAGCCG-ATACTATA-GGTATC-GCCATGCGA

AGGG--CTTTACTTGGAG-AAATAGAAGGAA--CATGTATC----ACACGTGCAAAATCT

----GAGAAGGTACCACATGAATATTC--------TACGATAGCGGGCATTGAAGAATCA

GTACATGAAATTTTAATGAATTTGAAAGAAATTGTATTGAGAAGTAATCTTTATGGAACT

CGAGACGCATCTATTTGCGTCAGAGGTCCTAGATGCGTAACTGCTCAAGACATCATCTCA

CCACCTTCCGTAGAAGTAGTTGATACTACACAACATATAGCTAGCCTGACGGAACCGATT

GATTTGTGTATTGGATTACAAATCCAGAGGGATCGCGGATATCGTATGAAAACCACAAAA

AACTCTCAAGATGGAAGTTA----------TCCGATAGATGCTGTATCCATGCCTGTTCG

AAATGCGAATCATAGTATTCATTCTTATGGAAATGGTAATC------AGAAACAAGAGAT

ACTTTTCCTCGAAATATGGACAAATGGAAGTTTAACTCCTAAAGAAGCACTTCACGAAGC

TTCCCGTAATTTGATTGATTTATTTATTCCTTTTCTACATGCAGAGGAAGAGGA---CAT

------TAATTTCGAGGAAAATCAAAACAGGTT---TACTGTACCCCCTTTTACCTTTCC

TGATAGATTGGCT------AATCTAAAGAAAAACAAAAAAGAAATTGCATTGAAATGTAT

TTTTATTGACCAATCAGAATTGCCTCCCA-GGACCTATAATTGTCTCAAAAGGTCCAATA

TACATACATTATTGGACCTTTTGAGTAACAG------TCAAGAAGATCTTATGAAAATTG

AATATTTTCGAATAGAAGATGTAAAACAGATATTGGACACTCTA-CAGAAGCATTTCGC-

---AATTGATTTA--CCTAAGAATA------AGTTT---------TAA------------

---------------ATGGGATTTCTTTTTTCGATAAACAGGAAACTTAAGATGCCCCGG

GA---TGGAAATGAGGGAATGTTCACAATACCTGGATTTAGTCAGATCCAATTTGAGGGA

TTTTGTAGGTTCATTGATCAGGGCTTGATGGAAGAATTTCATAAGTTTCCAAAAATTGAA

GATACAGACCAAGAAATTGAATTTCAATTATTTGTGGAAAGATATCAATTGGTAGAACCC

TTGATAAAAGAAAGAGATGCTGTATATGAATCACTCACATATTCCTCGGAATTATATGTA

CCCGCGGGATTAATTTGGAAAACCGGTAGAGATATGCAAG--AACAAACCATATTTATTG

GAAACATTCCTCTAATGAATTCCCTGGGAACCTTTATAGTAAATGGAATATACAGAATTG

TAATCAATCAAATATTGCAAAGCCCCGGTATTTACTACCGTTCAGAATTGGACCATAACG

GAAT---TTCTGTTTATACCAGTACCATAATATCAGATTGGGGAGGAAGATCAGAATTAG

AAATCGATAGAAAATCAAGGATATGGGCCCGTGTGAGTAGGAAACAAAAAATATCTATTC

TAGTTTTATCATCAGCTATGGGTTCGAATCTAAGAGAAATTCTAGATAATGTTTGCTACC

CCGAAATTTTC------------TTGTCTTTTCTGAATGATAGGGAGAAA-------AAA

AAGA-----TTGGGTCAA-AAGAAAATGCCATTTTGGAATTTTATCAACAATTTGCTT--

-----GTGTA--GGTGGAGATCCGGTATTTTCTGAATCCTTATGTAAAGAATTACAAAAG

AAATTTTTTCAACAAAGATGTGAATTAGGAAGGATTGGACGACGAAATATGAACCGGAGA

TTAAATCTTGATATACCTCAGAGCAATACATTTTTGTTACCACGAGATGTATTGGCTGCC

GCGGATCATTTGATCGGAATGAAATTTGGAATGGGTACACT------TGACGATATGAAT

CACTTGAAAAATAAACGTATTCGTTCTGTAGCAGATCTGTTACAGGATCAATTCGGCTTG

GCTCTTGTTCGTTTAGAAAATGCAGTTCGAGGAACTATATGTGGAGCA------------

---ATCAGGCATAAATTGATACTGACTCCTCAAAATTTGGTCAGTTCAACTTC-------

-----ATTAACAACCACTTATGAATCGTTTTTCGGCCTACACCCTTTATCTCAAGTTTTG

GATCGAACTAATCCATTGACGCAAATCGTTCATGGGCGAAAATTGAGTTATTTGGGTCCT

GGAGGATTGACAGGGCGAACTGCTAGTTTTCGAATACGAGATATCCATCCTAGTCACTAT

GGACGGATTTGCCCAATTGACACGTCCGAAGGAATCAATGTTGGACTTATTGGATCCTTA

GCTATTCATGCCAGGATTGGTCAT---TGGGGGTCTATAGAGAGTCCGTTTTATGAAGTA

T---ATCAGAGATC------AAAAGAGAC---AAAGATGGTTTTTTTATCACC------A

AGTAGAGATGAATATTAT------ACGGTAGCGACAGGAAATTCTTTGGCTTTGAATCGA

GGTGGTATTCAGGAAGAACAGATTGTTCCCGCCCGATACCGTCAAGAATTCCTGACTATT

GCATGGGAACAGATTCATCTTCGAAGTATTTTTCCCTTCCAATATTTTTCTATTGGAGCT

TCCCTGATTCCGTTTATCGAGCATAATGATGCGAATCGGGCTTTAATGAGTTCGAATATG

CAGCGTCAAGCAGTTCCGCTTTCCCGGTCCGAGAAGTGCATTGTTGGGACTGGATTGGAA

CGACAAGCGGCTCTGGATTCGGGGGTTTCAGCTATAGCCGAATGCGAGGGAAAGATTATT

TATACCGATACTCATAAGATCGTTTTATCAGGTCATGGAGACACTATAAGCATTCCATTG

GTTATGTATCAACGTTCGAACAAAAATACTTGTATGCATCAAAATCCTCAGGTTCGGCGG

GGGAAATGCATTAAAAAAGGACAAATTTTAGCGGATGGTGCGGCTACAGTTGGTGGCGAA

CTCGCTTTAGGAAAAAACGTATTAGTAGCTTATATGCCATGGGAAGGTTACAATTTTGAG

GACGCGGTACTCATTAGCGAACGTCTGGTATATGAAGATATTTATACTTCTTTTCACATC

CGTAAATATGAAATTCAGACTCATGTGACAAGCCAGGGTC---CTGAAAGAATTACTCAT

GAAATACCGCATTTGGAAGCTCATTTACTCCGCAATTTAGACAGAAATGGAATTGTGGCG

CTGGGATCTTGGGTAGAAACAGGCGATATTTTAGTAGGTAAATTAACCCCTCAGACAGCC

AACGAATCATCGTATGCCCCGGAAGATAGATTATTACGAGCCATACTTGGGATTCAGGTA

TCCACTGCAAAAGAAACTTGTCTAAAACTACCTATAGGCGGAAGGGGTCGAGTTATTGAT

GTGAGATGGATCCA----------------------------GAAAAAG-----------

---------------------GGAGGTTCCAGCTA-------------------------

--------------------TAATCCAGAAACGATTCGTGTATATATTTCACAGAAACGT

GAAATCAAAGTGGGTGATAAAGTAGCTGGAAGACACGGGAATAAAGGTATCATTTCCAAA

ATTTTATCTAGACAAGATATGCCTTATTTGCAAGATGGAACGCCGGTTGATATGGTTTTC

AACCCATTAGGAGTACCTTCACGAATGAATGTGGGACAAATATTTGAATGCTCGCTCGGG

TTAGCGGGGGACCTGCTAGACAGACATTATAGAATAGCACCTTTTGATGAGAGATATGAG

CAAGAGGCTTCGAGAAAACTAGTGTTTTCTGAATTATATGAAGCCAGTAAGCAAACAGCA

AATCCATGGGTATTTGAACCCGAATATCCCGGAAAAAGCAGAATATTTGATGGAAGAACG

GGAGATCCTTTTGAACAGCCTGTTCTAATAGGAAAGTCCTATATCCTGAAATTAATTCAT

CAAGTTGACGATAAAATCCACGGTCGTTCCAGTGGACATTACGCACTTGTTACACAACAA

CCTCTTAGAGGAAGGGCCAAACAGGGCGGACAGCGCGTAGGAGAAATGGAAGTTTGGGCT

CTCGAAGGGTTTGGTGTTGCTCATATTTTACAAGAGATGCTTACTTATAAATCTGATCAT

ATTAGAGCTCGTCAGGAAGTACTTGGTACTACAATCATTGGAGGAACAATACCTACCCCC

GAGGATGCTCCAGAATCTTTTCGATTGCTCGTTCGCGAATTACGATCTTTGGCTTTGGAA

CTGAATCATTTCCTTGTATCTGAGAAGAACTTCCAGATTAATAGGAAGGAAGCT------

---TGA---------------------ATGATCGACC---GGTATAAACATCAACAACTT

CAAATTGGATCAGTTTCTCCTCAACAAATAAGTGCTTGGGCCAACAAAATCCTACCTAAT

GGAGAGATAGTTGGAGAGGTCACAAAACCCTATACTTTTCATTACAAAACCAATAAACCG

GAAAAAGATGGATTATTTTGTGAAAGAATCTTTGGACCTATAAAAAGTGGAATTTGCGCT

TGTGGAAATTA-----------------------TCGAGTAATTGGCGCTGAAAAAG---

-AAGACCCCAAATTTTGTGAACAATGTGGAGTCGAATTTATTGATTCTCGGATACGAAGA

TATCAAATGGGATACATCAAGCTCGCATGTCCAGTGACTCATGTGTGGTATTTGAAACGT

CTTCCTAGTTATATCGCGAATCTTTTAGATAAACCCCTTAAGGAATTAGA------AGGC

CTAGTATACTGCGATGTGTGATTTGATCG-AAATTATCATTTTACAGATTCGGACTGAGA

AACTGTCATCCCATTCAATCCGACG---GGGGTGCCCTTGGCTCTGACATGTATCTTGGG

AGGAGTAACATGAAGCTTAGAATTTTGGATGCATTCAATACTTC----C-AAATAAAAGG

GGAATTGATCCATGGTCGAT---T-CTCTAACAG----ATAA-ACGGGAATTGCTAGTTG

TACCTCGTG-AAAAAA--------AAAAAGACTTTTTTCGTCAAATT------TGCCATT

TCATTTCTTTT--------------AGAGAG--ATTTTGTTCAAGTAAGC--AAACA---

----GCAGCATGGTTACAGGAGTCTATCTATCGCATATATG-----CTTCAAAGGACATC

GTG-GCATA-------ACCATCGAGGTGAAGTAGAG---------ACCT--AAAAGATCG

AATGGAACGATAGATAGTATCGACAAGTAAATTCCTTATGAGTTTCAAGGTATTCCTCG-

----AGAGGAATTCATTATTCGAAGGGAAGTAGACTACTCAATA-----ATTTCACATTC

CACTTATCTT-ATGTC------GTAGTCGTAATTGGATAAGTAATTCAGAAAGTAAAAG-

-TCAAAGGACAGATG-AATCA-ATGAAATATTGGTTAGTC----CTAACCGGG--AACTT

GAGTAAGGAGTAG-------CTGTTTGTTTTGTATTGTAGGGTTTTTTCTCGAACAAAGT

TTGAAAAC----GCGAACCCTCACTTTTTATTTAGTGTAACTACTTTAGCCGGATGAGAG

GAAACCTTCACGTCCGGTTTTTA-AGGGGGGAATCC-----TCTAGGAACCTATCCCTAT

TTTTCTTTTGCTAGGCCCATAGCTAAAAAACCGACTTTCTTACGATTACGAGGTTTATTC

GAATATGAAA---TCCAATCCTGGAAATACAGCATCCCCCTTTTTTTTAC----------

-----------TACCCAAGGCTTCGATACATTTCGAAATAGAGAAATCTCTACCGGAGCG

GGTGCTATCAGGGAACAATTAGCCGATTTGGATTTGCGAATTGTTATCGATAATTCATCG

GTAGAATGGAAGGACTT---------------------------AGGAGACGAAGGGTCC

------ACTGGGAATGAATGGGAAGATAGAAAAATTGGACGAAGAAAGGATTTTTTAGTT

AGACGCATGGAATTAGCTAAACATTTTATTCGAACAAATGTAGAACCAGAGCGGATGGTT

TTGTGCCTATTACCGGTTCTTCCTCCCGAGTTGAGACCAATTATTCAGATAGATGGGGGT

AAACTAATGAGT---TCGGATATTAATGAACTCTATAGAAGAGTTATCTATCGGAACAAT

ACTCTTACCGATCTATTAAAAA--CAAGTAGATCTACGCC-------------AGGGGAA

TTAGTAATGTGTCAGGAGAAATTGGTGCAAGAAGCCGTAGATACACTTCTTGATAATGGA

ATCCGCGGACAACCAATGAGGGATGGCCATAATAAAGTGTACAAGTCATTTTCAGATGTA

ATTGAAGGCAAGGAGGGGAGATTTCGTGAGACTTTACTTGGTAAACGGGTCGATTATTCG

GGTCGTTCCGTCATTGTCGTAGGCCCTTCACTTTCATTACATCGATGCGGATTACCTCGA

GAAATAGCAATAGAGCTTTTCCAGACATTTGTAATTCGCGGTCTAATCAGACAACATCTT

GCTTCTAACATAGGAATTGCTAAAAGCAAAATTCGGGAAAAAGAACCGATCGTATGGGAA

ATACTTCAAGAAGTTATGCAGGGACATCCTGTATTGCTAAATAGAGCGCCCACCCTGCAT

AGATTAGGCATACAGGCGTTCCAACCCGTTTTAGTGGAGGGACGTGCTATTTGTTTACAT

CCATTAGTTTGTAAGGGATTCAATGCAGATTTTGATGGAGATCAAATGGCTGTTCATGTA

CCTTTATCTTTGGAAGCTCAAGCAGAGGCTCGTTTACTTATGTTTTCTCATATGAATCTC

TTGTCACCAGCTATTGGAGATCCCATTTCTGTACCAACTCAAGATATGCTTATTGGCCTC

TATGTATTAACGATGGGGAATCGTCGAGGTATTTTTGTAAATAGGTATAATCCA------

TGTAATCGCAGAAATTATCAAAATAAAACAGTTGACAATAATA---------ACTATAAA

------CATACGAAAGAGAAAAAACCCTATTTTTTGAGTTCCTATGATGCACTTGGAGCT

TATCAGCAGAAACGAATCAATTTACATAGTCCTTTGTGGCTCCGGTGGCGAC---TAGAT

CAACGTGTCATTG------GTTCA------AGAGAAGTTCCTATCGAAGTTCAATATGAA

TCTTTGGGTACCTATCAGGAGATTTATGGGCACTATCTAATAGTAAGAAGTGTAAAAAAA

GAAATACTTTGTATATACATTCGAACCACTGTTGGTCATATTTCTTTTTATCGAGAAATA

GAAGAATC----------CGTACAAGGGTTTTGTCGAG--CCTACTCATATG--------

--------------GTACCTAA------------ATGGAGGTACTTATG--GCAGAACGG

GC---------------CGATCTGGTCTATCACAATAAAGCGATAGATGGAACTGCCATG

AAACGACTTATTAGCAGATTAATAGATCACTTCGGAATGGCATATACATCACACATCCTG

GATCAAGTAAAGACTCTGGGTTTTCGGCAAGCCACTGCTACATCTATTTCATTAGGAATT

GATGATCTTTTAACAATACCTTCTAAAGGATGGCTAGTCCAAGATGCTGAACAACAAAGT

TTTATTTTGGAGAAACACCATTCTTATGGAAATGTACACGCGGTAGAAAAATTACGTCAA

TCCATTGAGATATGGTATGCTACAAGTGAATATTTGAGACAAGAAATGAATCCTAATTTT

CGGATGACTGATCCTTCTAATCCAGTCCATATAATGTCCTTTTCGGGAGCTAGAGGAAAT

GCATCTCAGGTGCACCAATTAGTAGGTATGAGAGGACTAATGTCGGATCCCCAGGGACAA

ATGATTGATTTACCCATCCAAAGCAATTTACGTGAAGGACTTTCTTTAACCGAATATATA

ATTTCCTGCTACGGAGCCCGCAAAGGAGTTGTAGATACTGCTGTACGAACATCAGATGCC

GGATACCTCACTCGTAGACTTGTTGAAGTAGTTCAACACATTGTTGTACGCAGAACAGAT

TGTGGCACTATCCGAGGTATTTCTGTGCAACC---------TCGAAA------CGGGATG

ACGGAAAGGATGTTTTTTCAAAC------ACTAATCGGTCGTGTATTAGCAGACGATGTA

TATATAGGTCTACGATGCATTGCCGCTCGAAATCAAGATATTGGGATTGGACTTGTCAAT

CGATTCATAACC------------------TTTCGAGCACAACCAGTATATATTCGAACT

CCCTTTACTTGTAGGAGTACCTCTTGGATCTGTCAATTATGTTATGGCCGGAGTCCTACT

CACGGCGACCTGGTCGAATTGGGGGAAGCGGTAGGTATTATTGCGGGACAATCAATTGGG

GAACCGGGGACTCAACTAACATTAAGAACCTTTCATACTGGTGGAGTATTCACAGGGGGT

ACTGCAGAACATGTACGAGCTCCTTCTAATGGAAAAATAAAATTCAATGAGGAGTTGGTT

CATCCCACACGTACACGTCACGGACATCCTGCTTTTCTATGTTCTATAG---ACCTGTAT

GTAACTGT---TGAGGGTCGGGATATTATACATAATGTCAATATTCCACCAAAAAGTTTG

ATTTTAGTTCAAAATGATCAATATGTAGAATCAGAACAAGTGATTGCCGAGATTCGCGCC

GGAACATCCACTTTTAATTTTAAAGAGAGGGTCCAGAAACATATTTATTCCGAATCAGCG

GGAGAAATGCACTGGAGTACCGATGTCTATCATGCACCTGAATATACATATGGTAATGTT

CATCTATTACCAAAAACAAGTCATTTATGGATATTATCCGGAGGGCCATACAGA------

TCCAGTATAG---TGTCTTCGTCGCTCCACAAGGATCAAGATCAAACGAATGCTCATTCT

TTTTCTGTTGAACGGCGA------TATATTTCTGACCTCTCAATGACTA---ATGGTCGA

GTGAGACACAAATTATTTAGTTCGGATCTTTCTGGTAAAAG---------GGGGGATAGG

ATTCTTGATTATTCA---AG---ACCCGA--TCAAATCATATCC------AAGGGTCATT

GGAATTTTATATACCCTTC------TATTCTCCACGAGAATTCAGATTTCTTAGCGAAGA

GGCGAAAAAATAGATTCATCATCCCGTTCCAATATGATC----AAGAAGGGGAGAAAGAA

CTAA----------TACCGCATT------CTGGTATTTCGATCGAAATACCCATAAACGG

TATGTTGCGTAGAAATAGTATTCTTGCTTATTTTGACGATACACGATACAGAAGAAGCA-

-----GTTCCGGAATTACTAAAT-------------------------------------

------------------------------------------------------------

------------------------------------------------------------

------------------------------------------------------------

------------------------------------------------------------

------------------------------------------------------------

------------------------------------------------------------

--------------------------------------------------ACGGGACCGT

------------------------------------------------------------

------------------------------AGAGATAGATTCAATCGTTAAAAAAGAGGA

TTTCATTGAGTATCGAGGAACAAAAGAATTTAGTTCGAAATAC------CAAATGAAAGT

AGATCGATTTTTTTTCATTCCCGAAGAAGTGCATATCTTACCTGGATCTTCGTCCATAAT

GGTCCGGAACAACAGTCTCATCGGAGTAGACACACGAATCACTTT------AAATATAAG

AAGCCGAGTGGGTGGATTGGTTCGAGTGGAGAGAAAAAAAAAAAATATTGAACTCAAAAT

TGTTTCTGGAGATATCCATTTTCCTGGAGAGACAGATAAGATATCCAGGCACAGCGGTAT

CTTGATACCGCCAGGAACGG---AAAAAAAAAACTCTAAGGAATCAAAAACAAAAT----

-----TGAAAAATTGGATCTATGTCCA---GCGGATCACGCCTACTAAGA------AAAA

GTATTTTGTTTTGGTCCGACCCGTAGCCACATATGAAATAGCAGATGGGATCAATTTAGC

AACGCTTTTCCCTCAGGATCTATTGCAGGAAAGGGATAATGTTCAACTTCGAGTTGTTAA

TTATATCCTTTATGGAAATGGCAAACCAATTCGAGGAATTTCTCACACAAGTATTCAATT

AGTTCGTACTTGCTTAGTATTGAATTGGGACCAAGAACAAAATGGTTCTAT---AGAAG-

--GTGTTCGTGCTTCCTTTGTTGAAGTAAGGGCAAACGATCTTATTAGAGATTTCATAAG

AATTGAGTT------ATGCAAGTCCGCTATTTTGTATACCGGAAAGAGGAAGGATATCGC

AGGTTCAGGATTGATCCACGATAATGTATCGGATCGCACAAATAT---CAATCCTGTTTA

TTTGAAGGATAAGATTCCATCATTTATTCAACATCAAGGGAC------TGTTGGTACGTT

GTTGAATCGAAATAAGGAATGCC------AATCTTTGATTCTTTTGTCATCATCTAATTG

TTTTCGAATTGGTCCATTTAATGGTTCAAAATATCACAATGTGCCAAAAGAATCAATT--

----AAAGAGGATCCTATAATTCCAATTA---------------------GAGATTCGCT

GGGTCTTTTAGGTACTACTGTACCAAAAATT------GCGAATTTTTTTTATTTATCTTA

CCATGTAATAACTCATAATCAT---------ATCTTGTTAACTAAATATTTGCTACG---

---TGACCATTTAAAACAGGCTT---------TCCAAGTAC------TTAGATATTGTTT

AATGGATGAAAATAGGAGAAT------TTATAATCCCGATCCGTGCAGTAACATCATTTT

TAATGCATTCGATTTGAATTGGCGCTTTCTCCATCACGA------TTATTCTGAAGAGAC

ATCCAC------AATACTTAGCCTTGGACAATTTGTTTGTGAAAATGTATGTCTATTTAA

ACACGGACC------ACAGAT---AAAATCGGGTCAAGTTATAATTGTTCATGTTGACTC

CTTTGTAATAAGAGCAGCCAAGCCGTATTTGGCCACTCCAGGAGCAACTGTTCATGGCCA

TTATGGAGAAATACTTTACGGAGGAGATACATTAATTACATTTATATATGAAAAATCAAG

ATCTGGTGATATAACGCAGGGTCTTCCAAAAGTAGAACAAGTGTTAGAAGTACGTTCAAT

TGATTCCATATCGACGAACCTCGAAAAAAGGGTTGAAGGTTGGAACGAACATATAACAAA

AATTCTTGGAATTCCCTGGGGATTCTTGATCGGCGCCGAGTTAACCATAGCGCAAAGTCG

TATCTCTTTGGTTAATAAGATCCAAAAAGTTTATCGATCTCAGGGGGTGCAGATTCATAA

TAAGCATATAGAGATTATTGTACGTCAAATAACATCAAAAGTGTTGGTTTCAGAAGATGG

AATGTCTAATGTTTTTTCACCGGGAGAACTTATCGGATTGTTGCGGGCGGAACGAACAGG

TCGCGCTTTGGAAGAAGCGATCTGTTATCGAGCAATCTTATTGGGAATAACAAGAGCGTC

TTTGAATACTCAAAGTTTCATATCCGAAGCGAGTTTTCAAGAAACTGCTCGAGTTTTGGC

AAAAGCGGCTCTACGGGGTCGTGTTGATTGGTTGAAAGGCCTGAAAGAGAACGTTGTTCT

AGGGGCGATGATACCCGTCGGTACGGGATTCAAAGCATTAG-TACACCGG---T-CAAG-

------AAAACCCAACAACATTCATTTGGAAATCAAAAAGAATAATCTATTTGA-GGGGC

AAGTGGGAGATGATATTTTGTTCTACCACAGAGAATTATTT-------G------GTTCT

TGCG-GC-----CCAAATAATTTCCATGAT---------------ACATCAGAACAGTCA

TTT---------------------------------ATGAAATTTCATGATTCCTAGATG

ACA---------------------------------------------AAAAGATATTGG

AACATCAATTTGGAAGAGATGATGGAA-GCAGG--------AGTGCATTTCGGTCACGGT

ACTAGGAAATGGAATCCTAGAATGGCACCTTACATTTCTGCAAAGCGTAAAGGTATTCAT

ATTACAAATCTTACTAGAACTGCTCGTTTTTTATCAGAAGCCTGTGATTTACTTTTTGAT

GCAGCAAGTGGGGGAAAACACTTCTTAATCGTTGGTACCAAAAAGAAAGCAGCTGATTCA

GTAGCATCGGCTGCAATAAGGGCTCGGTGTCATTATGTTAATAAAAAATGGCTTGGTGGT

ATGTCAACGAATTGGTCCACTACAGAAACGAGACTTCAAAATTTTAGGGACTTAAGAGCA

GAACAAAAGGCTGGGAAAATCGACC---GTCTCCCGAAAAGGGATGCAGCAATGTTGAAG

AGACAATTATCTACCTTGCAAACATATCTGGGTGGGATCAAATATATGACGGGTTTACCT

GATATTGTAATCATCGTTGATCAGCAAGAAGACTATACGGCTCTTCGAGAATGTGTCATT

TTAGGAATTCCGACTATTTGTTTAATCGATACAAATTCTGACCCGGATCTCGCAGACATT

TCGATTCCCGCCAACGATGACGCTATAGCTTCAATCCGATTGATTCTTAACAAATTAGTA

TTCGCAATTTGC----GAGGGTCGTTCTAGCTATATAAG------------AAATCGTTA

AATGGGACAAAAAATAAATCCACTTGGTTTCAGACTTGGTGCA--ACCCAAAGTCATCTT

TCCCTTTGGTTCGCACAACCAAAAAGTTATTCTATGGGTTTACAGGAAGATGAAAAAATA

CGGGAGTGTATCAAGAATTATGTACAAAAAAACCCGAGAC--------------------

-------------------------------------------------TGTCTTCAGGA

TTCGAA------GGAATAGCGCGTATAGAAATTAAAAAAAGAATCGATCTGATCCAGGTC

ATAATCTATATCGGATTCCCAAAT---TTATTCATAGAGGGTCGAACGCAAGGAATCAAA

GAATTACAGATGAGTGTACAAAAAAGATTAAATTCTGTGAATCAGAGACTCAACATTGCT

ATCGCAAGAATTTCAAAACCTTATGGGCAACCCAATATTCTTGCTGAATATATAGCTTTA

CAACTAAAAAATAGAGTTTCGTTTCGAAAAGCAATGAAAAAAGCTATTGAATTGACTGAA

CAAGCAGATACAAAAGGAATTCAAATTCAAATCGCAGGACGTATCGATGGAAAAGAAATT

GCACGTGTCGAATGGATAAGAGAAGGTAGGGTTCCCCTACAAACCATTCGAGCTAAAATT

GATCATTGTTTCCATAAAGTTCAAACTATCTATGGGGTATTAGGCATAAAAATTTGGATA

TTTGTAGACGAG---GAATAA------ATGTCTCGCTACCGAGGGCCTCGTTTAAAAAAA

ATACGCCGTCTGGGGGCTTTACCCGGACTAACTAGTAAAGGAACTAGACCCGGAAGTGAT

CTTAGAAATCAATTTC----GTTCTG--GTAAAAGATCCCAATATCGTATTCGTCTTGAA

GAAAAACAGAAATTGCGTTTTCATTATGGTCTGACAGAGCGACAATTACTTAGATATGTT

CATATCGCCGGAAAAGCCAAAGGATCAACAGGTCAGGTTTTACTACAACTACTTGAAATG

CGTTTGGATAACATCCTTTTTCGATTGGGTATGGCTCCTACGATTCCTGGAGCCCGGCAA

TTAGTTAA--CCAT-AGACATATTTTAG--TTAATGGTC--GTATAGT-AGATATACCAA

GTTATCGTTGCAAACCCA--GAGACATTA-TTACTAC--GAAGGATAAGC----AAAGAT

CGAAAGTTCTGATTCAAAATAATATGGATTCATCGACCCGCGAGGAATTGCCAAAACATT

TGACTCT---TGACTCATTCCAACATAAAGGATTAGTAAATCAAATCATAGATAG-TAAA

TGGGTCGGTTTGAAAATTAATGAGTTGTTAGTTGTGGAATATTATTCCCGCCAGACTTAA

ATGTCACGTCGAGGTACTGCAGA------AGAAAAAACTGCAAAATCCGATCCAATTTAT

CGTAATCGATTAGTTAACATGTTGGTTAACCGTATTCTGAAACACGGAAAAAAATCATTG

GCTTATCAAATTATCTATCGAGCCTTGAAAAAGATTCAACAAAAGACAGAAACAAATCCA

CTATCTGTTTTACGTCAAGCAATACGTGGAGTAACTCCCGATATAGCAGTAAAATCAAGA

CG---TGTAGGCGGATCGACTCATCAAGTTCCTATTGAAATAGGATCTACACAAGGAAAA

GCACTTGCCATTCGTTGGTTATTAGCGGCATCCCGAAAACGT---CCGGGTCGAAATATG

GCTTTCAAATTAAGTTCCGAATTAGTAGATGCTGCCAAAGGGAGTGGCGATGCCATACGC

AAAAAGGAAGAGACTCATAAAATGGCAGAGGCAAATAGAGCTTTTGCACATTTTCGTTAA

ATGGGTAAGGACACTATTGCCGATATAATAACTTCTATAAGAAATGCTCAAATGGCTAAA

AAAGGGACAGTTCGAATAGCATCTACGAATCTTACCGAAAACGTTGTTAAAATACTTCTG

CGAGAAGGTTTTATTGAAAACGTTAGAAAACATCGGGAAAGTAATAAAGATTTCTTGGTT

TTAACCCTGCGACAT------AGAAGGACTAGGAAAGG----------------------

------------------------------------------------------------

--------------------------------------GATATAT---------------

------------------------------------------------------------

------------------------------------------------------------

------------------------------------------------------------

------------AGAACTA------TTTTAAAGCGTATCAGCCGACCTGGTTTACGAATC

TATTCCAACTATCAAGGAATTCCCAAGATTTTAGGCGGAATAGGAATTGTAATTGTTTCT

ACTTCTAGAGGTATAATGACAGATCGGGAGGCTCGACTACAGGGAATTGGAGGAGAAATG

TTATGTTATATATGTTGAATGACAAAAACTATACCAAGAATTGGTTCACGTAGGAATGGA

CGTATTGGTTTACGT------------------------AAGACTGGACGTAGA------

------------------ATACCAAAAGGAATTATTCATGTTCAGGCAAGTTTCAACAAT

ACCATTGTGACTGTTACAGATGTTCGGGGTCGAGTAGTTTCTTGGTCCTCCGCCGGTACT

TGTGGATTCAAAGGCACAAGAAGAGGGACGCCGTTTGCGGCTCAAACCGCAGCGGGAAAT

GCTATTCGTACAGTAGTAGATCAGGGTATGCAACGAGCAGAAGTCATGATAAAAGGTCCT

GGGCTCGGAAGAGACGCAGCATTACGAGCCATTCGTAGAAGCGGCATATTATTAAATTTC

GTACGTGACGTGACCCCTATGCCACATAATGGATGTAGACCTCCTAAAAAAAGACGTGTG

TAGATGCCAACTATTAAACAACTTATTAGAAACACAAGACAGCCAATCCGAAATGTCACA

AAATCCCCCGCTCTTCGAGGATGTCCTCAGCGTCGAGGAACATGTACTAGGGTGTAT-AC

TATCACCCCCAAAAAACCAAACTCTGCCTTACGTAAAGTTGCCAGAGTACGATTAACCTC

TGGATTTGAAATCACTGCTTATATACCTGGTATTGGCCATAATTTACAAGAACATTCTGT

AGTCTTAGTAAGAGGTGGGAGGGTTAAGGATTTACCCGGTGTGAGATATCACATTGTTCG

AGGAACCCTAGATGCTGTCGGAGTAAAGGATCGTCAACAAGGGCGTTCTAGTGCGTTGTA

GATT----CTTATCCAAGACTTGTATCATT---TGATGATGCCATGTGAATCGCTAGAAA

CATGTGAAGTGTATGGCTAACCCAATAACGAAAGTTTCGTAAGGGGACTGGAGCAGGCTA

CCATGAGACAAAA-GATCTTCTTT-CTAAAGAGATTCGATTCGGAACTATT------ATA

TGTCCAAGGTCCAATATTGAAATCATTTCAGAGGTTTTCCCTTACTTTGTCCGTGTCAAC

AAACAATTCGAAATACCTCGACTTTTTTAGAACAGGTCCGAGTCAAATAGCAATGATTCG

AAGCACTTCTTT-----TTACACTCTTTCGGAAACCCAAGGACTCGATCGTATGGATATG

TAAAATACAGGATTTCCAATCCTAGCAGGA-AAAGGAGGGAAACGGATACTCAATTT---

---AAAGTGAGTAAACAGAATTCCATAC--------TCGATCTCAT-----AGATACATA

TAGAATTCTGCGGAAAGCCGTATTCGATGAAAGTCGTATGTACGGCTTGGAGGGAGATCT

TTCATATCTTTCGAGATCCACCCTACAATATGGGGTCAAAAAGCCAAAATAA--------

---------ATGGCAAGGAAAGGTTTGATTGAGAGGGAGAAGAAGAGGAAGAAATTGGAA

CAGAAATATCATTCGATTCGTGGATCCTCAAAAAAAGAAAT---AAGAAAAGTTCCATCG

TTGAGT------GACAAATGGGAAATTCATGGAAAATTGCAATCCCCACCACGTAATAGT

GCACCTGTACGTCTTCATCGACGTTGTTTTTTGACCGGAAGACCCAGAGCTAACTATAGG

GATTTTGGGTTATCCGGACACGTACTTCGAGAAATGGTTCAGGCATGTTTGTTGCCGGGC

GCAACAAGATCAAGTTGGTAAATGGAAAAGAAAAAAATAAATAAAAAAGAATTTTTTATG

GT---CAAAAATTCATTTATCT-CGCTTATTCCG------CAAGAAGAAAAAGAAGAA--

----AACCGGGGATCTGTTGAATTTCAAGTATTCAGTTTCACCAATAAGATACGGAGACT

TACTTCACACTTGGAATTACACAGAAAAGATTATTTATCCCAGAGAGGTTTACGAAAAAT

TCTAGGAAAACGTCAACGGCTGCTGGCTTATTTGTCAAAGAAAAATAGAGTACGTTATAA

TAAATTAATTGGTCAGTTGGATATTCGGGAGCCAAAAACTCGTTAAATGGTAAAACTTCG

TTTAAAACGATGCGGTAGAAAGCAACGTGCGACTTGAAGGACATGATCGGC--TGTGGAA

TATTACATCCACCATTTTCTATAGGAATGAAGATGCTCTTGGCTCGACATAGTTTGTTCT

GTTCCATTGGGA----CCCAAATTTTGTTGGGTTG-----------------TAA--AT-

----------------------------------------AGTACATGATGGAGCTCGAG

-CAGAA-AG-----GATTGA----TTTATT-TTTC-AGGGGGAAGAATCTAGGGTTAGCA

CC------AATCAATAAGTTGGAACAGCTTTGTAA---GTATATCTATACTA--------

-------------------AATAGAGAAATCGAAGGGTT----------CAAATTCGAAC

CCAACAAGTTTGAAATAAAAAAA-----------AAATTGTCGGAAT-TGGTAAAACTAT

TTCGATCAA-----AAGTGTATCAAAGTGGAATT----------TATTGTTCGTATG---

----ATTTTTCCATA-----------------------------TAAAGAAATAAAAAGG

GTATGTTGCTGCCATTTTGA------AACAATTAAGGATCACCGAAGTAATGTCTAAACC

CAATGATTC----------AAAGCAAAGATAAAGGATCCCGGAACAAGAAAACACAATTT

TCAA---TTGT------CTTAA----------------------CAATTGGATCCGAATG

AGAAAT--------------CAAA----ATGGATTCGAGACGAGACAAAG-AAAAGAGGT

TAGAGACGGCTCAAGAAA-------TG--------TCTAAAGAGTTCTTT----------

---TCAG----AATTACCCAACTTG-AGTTATGAGTACGAATGATATTTT-------TTT

TTTTGAAGGAAGAAGGTAAAAACGACTCAAATTTCAAATTAGAGTCTAATTACCGAT---

-------------TTTATGAATCCATTTGACA-----CTATATACAG-------------

------------------------------------------------------------

------------------AAGAGAAATTAGAA----------------------------

-------------------TCATTCTTTCTCGAGCCGTATGAGGAGAAAACCTCATATAC

GTTTCTA-----GGGGGGGTATTGTTTATATATATCTATCCCAATGAGCCATTTATCGAA

---TCGTTGCAATTGATGTTCGATCCCGAAGAGAGGGAAGAGATCTTTGTAAAGTGGGTT

TTTATGATCCGATAAAAAATCAAACTTATTTAAATGTTCCTGTTATTCTAAATTTTCTGG

AAAAGGGGGCTCAACCTACAGAAACTGTTCATGATATTTTAAAGAAGGCAGAGGTATTT-

---------AAGGAACTTCAAGG------------------------AAATCAAACGAAA

TGAATGGATAAA------------------------------------------------

---------TCCAAGCAAC-----------------------------------------

------------------------------------------------------------

-------------------TTTTTCGTAAATCCAAGCGATC-------------------

--TTTTCGTAGGCGTTTGCC------------------------CCCAATTGGATCGGGG

GATCGAATTGATTATAGAAACATGAGTTTAATTAGTCGATTTATTAGTGAACAGGGAAAA

ATATTATCTAGACGAGTGAATAGATTGACCTTGAAACAACAACGATTAATTACTATTGCT

ATAAAACAAGCCCGTATTTTATCTTCGTTACCTTTT-----------CTTAAT------A

ACGAGAAACAATTTGAAAGAACCGAGTCAATACCTAGA-CCTACCGGTCCTAGAAGCAGA

AATAAATAG---------------------------------------------------

----------------------------------------------------GTGACACG

TTC------------------------------------------------------ATT

AAAAAAAAATCCTTTTGTGGCCAATCATTTATTGGAAAAAATTGAGAAGCTCAACATG--

----------------------AAAGAGGAGAAAGAAATAATAGTAACTTGGTCCCGGGC

ATCTACCATTATACCCACAATGATCGGTCATACAATTGCTATTCATAATGGAAAGGAACA

CTTACCCATTTATATAACAGATCGTATGGTCGGTCACAAATTGGGAGAATTCGCACCTAC

TCTTACTTTCCGGGG------ACACGCGAGAAA---------------------------

------------------------------------CGATAATAAATCTCGTCGTTAAAT

GATT-------------------------------TTCAAATCATT---TCTACTAGGTA

A---------TCTTGGATCCTTATACA--------------TAAAGATAATAAATTCAGT

CGTTGTGGTCGGACTATATTATGGATTTTTGACA------ACATTCTCCATAGGGCCC--

--TCTTATT--------TCTTCCTTCTCCGAGCTCGGATTATGGAAGAAGGAACCGAGAG

GGAGGTATCAGCAACAACTGGTTTTATTACGGGACAGCTCATGATGTTCATATCGATCTA

TTATGCGCCTCTGCATCTAGCATTGGGTAGACCTCATACAATAACTGTCCTAGTTATACC

GTATCTTTT-------GTTTCAT------TTCTTCTCGAACAATCAAAAACAATTTTTTG

--ATTATGGAT---------CTAC---TACCAGAAATTCAATG---------CGTAATCT

CAGCA--------------------TTCAATGTGTA------------------------

------TTCCTAACTAATCTAATTTTTCAACTCTTCAACCATTTAATGTTACCAAGTTCA

ACGTTAGCGAGATTAGTCAACATTTATATGTTTCGATGCAACAACAAGATGTTATTTGTA

ACAAGTAGTTTTGTTGGTTGGTTAATTGGTCACATTTTATTCATGAAATGGGTTGGA---

------------TTGGTAGTATCCTGGATACGGCAAAATCATTCTATTCGATCTAATGTA

CTTATTCGATCTAATAAGTACCTTGGGTTAAAATTGAAAAGTGCTATAGCTCA-------

--AATCTTGAGTATT-ATCTTCTTTATCGCCTGTGTCAA--CTATTTAGCCAGACTACCG

GATCCTAGTGTCACT---------------------------------------------

------------------------AAGAAACTG------AACGAAACCTCAAAAACGGAA

ACGGAAGAAGAAGAAAGTAAAGAAAGTCAAAAAAG-------------------------

---------------------------------------TAAAGAAAGTGAGGA------

---------------------------------AGAAAGAGACG------TAGAAAAAGA

AACAA------------------------------CTTCCGAAACG------AAGGAGAC

T--------------AAACAG---GAACAAGAGGGATC--CACCG---------------

-AGCAAGACCCTTCCC---CTTATT------GGGAAGAAAA------------GG-----

--AGGATCCGGAC---AAA---------------ATAGATGAAACGGAAGAGATCCGAGT

GAATGGAAAGGAAAAAAAAAAGGATGAAACGGAAG--AGATCCG---------AGTGAAT

---------GGAAA----------GGAAAAAAAAAA------------------------

-----------------------------------------GGATGAATTCCGCTTT---

---------------CACTTTAAAGAGAC--------------AGACTAT-----AAACG

TAGCCCAGG------TTAC---------GAAAACTCT------TATCTTGATGGGTATCA

AGATAA------TT--------GGGACTTGCAA------AG-------------------

TGAA--------GAAGAAGAAGAAGAAGAAA------GTACTTTAGGGT-----------

TTGAAAAACCTCTTGT---------------------AACTTGTCTTTTCGA--------

-----------------CTATAAACGAT--GGAATCGACCTTTTAGATATATAAAAAA--

--------------------------CAAAACA----------------------TTCGA

------GAA---TGCTATAAGAGACGAAATGTCACAA-----------------------

---------------------------------TATTTTTTTTATACATGTCTGAA----

---TAATGGGAAAAAAA------------------AAATATCTTTTAC------------

--------------ATATCCACCTAGTTTAGCGACTTTTTCG---------GAAATAAT-

-AGAAAGA----------------------------AAGATGTCTTTG------------

TACACAACAAAAAAACTAACTG-----ATGACG----------------AC------TTG

TATAG------TCATTGGG----------------TTTCCAAC--------AATGAAGAG

AAAAAGAACAACTTA-AGTAATGAGTTGATAAACCGAA------------TAAAAGTTTT

AG---------------ACAAGGGATCCCTTGCTCCGGATGTGCTCGAAAAAAGG-----

---------ACGAGATTGTGT----------------GATGATGAGAATGAACAA--GAA

TGCTTACCTAAAAAGTATGATCC--TTTGTTGAATGGATCATATCGCGGAAGAATAAAGA

AATTAGAATC------------------------------AGAATCAACTAG-------G

AACGA---------TTCAATCATTTC---------------C------------GCAAA-

--------------------------AGGTTCACTAGAAAAAATTTGGAAAAATAAGATA

CACAGTCTTAT--------------------TACTAATGATTCTCG------------AG

AATTTGA-------------------------------ACATCAAATGGATCCGTT----

--TGACGGAGAATCTTTAT------------------------------CGGCATATATG

ACTCATTCCTTAACC------TCAATCAGTAAATTGTCTTTGGAATTAGTATCTATTTTC

CATTTTGAGGATCTT---------GCTTTATTGACAGAACAAAAAAGAA-----------

----TTGATTTTGAAA-------ATCAAACGAAACGGTTG----------------AAAT

TTCTATTCGA--------TGTAATTAC-----------AGCTGACGCAAATAATCAAA--

C---CATTGAAAAT-----------AAATTTAT------TGAAA-------T------A-

-----GAAAAGATCGACAAAAA-------GATTCCTAGA---------TGGGTATA----

-----------------CAAATTGATATCCGAG---GA--GGATTATTTGGCACAA---C

AAGA--------------------------------ACAACAGGA--AGAAGAAAA----

------------------------------------------------------------

------------TG---AGGA-------AGAAGCACCACTGG---ATTTTGGAATTC---

-------------------------GTTCGAGAA------------------AAGGCAGA

CG------------------TGTAGTAATTTAT-------------------ACTGATAA

AAATCAGAATCGGGATCAGGATCC-------------------CAATCC------TAAT-

------------------------------------------------------------

------------------------------------------------------------

-----------------------------------ACCGATAATACTACTACCGAGAACG

ATAAT-------------------------------------------------------

-----AGTGATACG---------------GGGGAA------GAAATAGCTTTGATACGTT

ACGCGCAACAATCAGATTTTAGTCGGGATCTGATCAATGGATCCATACGCGCTAAAAGAC

GTAAAATAGTTC---TTTGGGAAA-----------TGTTAC---AAGCAAATGCGCATTC

CCC------ACTTTTTTTAGAT----------CAAGTAGCAAAAATGGTTTCTTTT---G

ATCT---------CTTTGATGATCT---------TCGTGAAACAATGAATCT------TA

TTTTTAGGAATTGGATAACAAAGGAGC------CAGAATT---------ACAAATTTTG-

-----------GATTCTGAAGAGAAAGA-------------------GGACAGAGAAAAA

CTGA---------TAAA-------------GATAAGGGAGGAAG----------------

-----------------ATGA------ACGG---ATAATAATATCCGAAACTTGGGATAA

CGTTATGTGTGCCCAAGCAA-------------TAAGAGGCTGTATGCTAGTAACCCACT

CATTTATTAGAAAATACATTATATTGCCTTTATTAATAATAGCTAAAAACATTGGGCGTA

TATTATTATTTCAGCTCTCTGAGTGGGATGAGGATTTTAAGGACTGGAATAGAGAAATGC

ATGTTAAATGCACCTATAATGGTGTTCAATTATCAGAAACAGAATTTCCTCAAAACTGGT

TAAAGGATGGTATTCAGATAAAGATCCTATTTCCTTTTTCTCTAAAACCTTGGCGCGAAT

------CTA--AGGCAACACCC------TCTACTGGAGGTCTAATGAAAGAGAAAAAAAG

AAAAAATGACGATTTTT--------GTTTTTTAACAGTTTGGGGAATGGAAGCGGAACTC

CCTTTTGGT---CCCCCTCGAAACC----GACCTTCT-----TTTTTTAAACCCATTTTT

GAAGAACTTGA------------CACAAACA------------TTCGAAAGGTGGAAAAC

CAAA------GTTTT---GTTTTCAAAG---------AAAA---------------AACA

AAAGACT---------TTTTAAAGAAAAAAAC---------G---GGATGGGTTA-----

--------------------------------------CAAAAATCGTTTTAT-----TG

AAAAATA------AAATAAGGAACTTTTTT------------------------------

---------------------ACAAAAGTAAACCCCAATTTATTATTCGGATTGAA----

-----GAAAGTATAT---------GAACCAAGTGAGAATCAA-----AAAGATTCCATAA

T------------TAGTAATAAGA---------------TTACCC------AC-----GA

ATCGACTGTTCAAATTCCATCCAGCAATTGGACAAATTATTCACCGATAG----------

------------------AAAAAAAAA--------TGAAGGATCTGTCTGATAGGACAAT

CACAACCAGGAGT---------------------CAAA------TAGAA------CGCAT

CAC----AAA--------------AGACAAGCAAA------AAGGATTTCTAACTTCA--

-------GATATAAATATTCGTTCTAATGAGACAA---------GTTGTAATGCTAAAA-

-----GAACGGAATT-------GCAGAAA---------GATATTTTACGGAT--------

-AGCA---AAAA------------------------------------------------

----------------------------AAAGAAG---------------TATCCGATTC

ATCCGTAAA---TTACACTC-----------------TTTTGTGAAA-------------

---------TCTTTCATTGAAAGAATATACCTAGATA-----------------------

-------------------------------------TCTTTCTATGTACCATTAACA--

TTCCCCGAATTAATCTA------CAACT---------------TTTCTTTGAATCAATAA

AAAAG-------------------ATTCTCAATAAAT---CCATTTCT-----------A

ATGATGAAAGAAAT---------------AAAGA--------------------------

-------------------------AAAAATTGATGAAACAAATCAAAATAC--------

------AA------TTCACTTTATTTCTACT--------ATAAGAAATTCG---------

------------TTTTCTAATA---GTAATCTTAA------------------------T

AATAAATCAAAGATTT---------------------------ATTGGGATTTCTCTTTA

TTGTCCCAAGCATATGTATTATATAAATTATCACAAACCCAAGTGATTAAAAGGTATCAA

TTGAAATCTGTACTTCAATAT------CACAGAGCATATCCTTTTCTGAAGGATAGAATC

AAG------------GACCTTTTTGGGACACCAAGAATAGTTCATGCCAAATCAAGGCCT

AAGAAACTTCCTATTTCGAGAATA-AATGCATGGAAAAACTGGTTAAGGGGTCATTATCA

ATACAATTTATC-TCAGA--CTAGATGGTCTGAATTAGTACCGAAAAAATGGCGAGAGAA

ATTCAATCAAC---GTACGA------TTAAAA------ATAAATACTCAAGAAAATTGAA

TTCATCTGAAAAAGAAAAAAACCAACAAGCTCATTACGCG----AAAGAAAT------TT

CTTATGT------AGTGGATTCATTGT---------------------------CGAGTC

AAAAAGGAAAATT----GAAAAAAGACTACAGATATGATCTTCTATCACATAAATATATT

AATT---------CCAATTATGAGGATAG---------------------GGAGTACTCA

GATATTTCGAGATTAT----------------------CATTACAAATAAATGGG-----

------------------------------------------------------------

------GATCGAGAAATTCCATA------TGATTAC-AAT---------AGACAAGAATC

CGA------TT---ATGTACTAGTTGGTTTACCTATTAGTGATTATCTAGGAGAAGAATA

TTTTATTGGCGTAGATAAAA------ATTCGGA-----TAGAAA---ATATTT-TGATC-

GGAGGATTCTCCGTTTTG-A-----------CCTTAGAAAGGATCTTA------ATATTA

AGACCCAGATCAATAGGGATACAGAGACC---------------AACATGAATAAAAA--

-------CGGAACTAATAA--TTATCCAGCAATTG-GTAAGAAA-----AATCCTTTTTC

TC------TCCCGATTCATCAAGAAATCAATT------CATCCAAGAAAAAAAAACAAAA

ATT------TTTTGATTGGATG---GGAATGCGCGAAGAAATTTT---------ATATCG

TCCGATAT-----------------CGAATCTGGAGCCTTGGTTT-------------TT

CCCAGAATTTGTGCTACG------------------TTACGATGCATATAAGAGTAAACC

ACGGATCATATCAATCAAATCTCTTCTTTTAGATTCTCAGAAAGACGAGCGAAATGAAA-

-----------------------------TAATTAGTAAAACAAAAAACA----------

------TCAACAAAAAAAA-------------------TCAAAAAAAAGAT---------

---CTATCTAATCAA------AA--AAAACATCTTGAAGTAGAGAA---------TCAAG

AAAAAGAGGATTTTCGACAAGTAGATCTTAGACCCAATCCAACAA-------ATCAAA--

--GGGATCCTAT--------------------TGTATCAGAT------------ACACGA

AACC-------------------AACAAAAAGATATTG----------------------

-----------------AAGAGGA---------TTCCGTGGGATCAGGCGTTAAAAAACG

TAGAAAAAAAAAGAAATT---------------CAAGAG-TAAGAAGGAAGCAGAATTAG

ACTTTTTTCTAAAA-----AAATAT----------CTCCT--------TTTTC-------

---AATTGAGATG-------------GG---------ATGAGCTTTTGAAT---------

-AAAAAAA----------TGATGAC-----------------------------------

-TAATATTAAGGTATATTGTCTCCTGCTTAG-------ACTGAAA--AATCT-------A

AAGGAAATCGCAA-------------------------------TATC----CTCCATTG

AAAGA-----------------GGAGAGATGTGCCTGGATTTAATGCT------AAT---

---GCAAAAAGATCTATCTCTTAGGG----------------------------------

AATTGATA--------AAAAAAGGAAT------ATTC-----------------------

------ATTAT------CGAACCGATTCGTTTATCTAGAAAATGGGA-------------

-------------------------------------------------CGGAAAATTGA

TTATGTATCAAACCATAGGTATTTCATTG-------GTCGCTGAGA--------------

-ATAAGGATC------CAATTAATATAAGATGTCGAAAAAAAGGAT--------------

-----ATGCTGAT---GAGAATTCTTTCAATAAATCCGTTAGACAACAAGAAAAAATGCT

TGT---AGAT-AGAGACGAA---------------------------------AATGATT

ATGATTTGC------------TTGTTCCTGAAAATATTCTATCTCCTAGACGTCGTAGAG

AATTGAGAATCCTAATTTGTTTTAATTCCGGGAATG---AGAGTGCT------GTGGATG

GAAATTCAGTATTTTACAATAACAAGAATGTGG------AGAGCTGTAGAC-----AATT

TTTGGATGA-----------GGACAAGCATCT------TGATACAGATGC------AAAG

------AAATTTATGAAATTG------------------AAATTTTTTCTTTGGCCCAAT

TATCGATTAGAAGATTTAGCTTGTATGAATCGCTATTGGTTTGATACCACTAATGGCAGT

CGTTTCAGTATGTTAAGGATTCATATGTATCC-ACGATTCCTAATCAGTTGGTGGTAG--

-------------------------------------------------ATGAAACGACA

TCAAT------------------------TCAG---------AT----------------

------------------------------------------------------------

-TCTGGATTTTCGAATTGAGAGAGATATTGAGAGAGATCAAGAATTCGCCCCATTTCTTA

GATTCATGGACCAAATTCGATTCAGTGAGATCTTTGATTCACATTTTTTTCCACCAAGAA

CGTTTGATGAAACTCTTTGACCCCCGAATTTGGAGTATCCTACTGTCACGCGATTCAC--

----AGGGTTCAACAAGCAATCGATATTTCATTATCAAAGGTTTAGTACTTCTTGTAGTA

GCGGTCCTTATATCTCGTATTAACAATCGA---AATATGGTCGAAAGAAAAAATCTCTAT

TTGAGGGGGC---------------TTCTTCC---TATACCTATGAATTCCATTGGACCC

GGAAAT------------GAGACATTGGAAGAATCCTTTTGGTCTTCCGATATCAATAGG

TTGATTGTTTTGC------TCCTGTATCTTCCAAAAGGGAAAAAGACCTCTGAGAGTTGT

TTCATGGATCCGAAAGAGAGTCTTTGGCTTTTCCCAATAAATAAAATAAATCAAAAGTGT

ATCATGCCCGAATCTAACC------------GGGGTTCGCGGTGGTGGAGGAACCGGATC

GGAAAAAAGAGGGATTCTAGTTGTAAGATATCTACTAAAACCGTAGCTGGAATTGAGATC

TCATCCAAAGAGAAAGATCTAAAATATCTGGAGTTTCTTTTTTTATCCT------ATACG

GATGATCCGATCCGCAAG------------------------------------------

------------------------------------------------------------

------------------------------------------------------------

------------------------------------------------------------

------------------------------------------------------------

------------------------------------------------------------

------------------------------------------------------------

------------------------------------------------------------

------------------------------------------------------------

------GATCGGTTTTTTAGCAAGGTACAGAATGTATCGTCAAATATTCAATATGATTCC

ACAAGATCTATTTTCGTTCAAGTAACGGATTTTAGCCAATCGAAAGGA---TCTTCTGAT

CAATCCAGAGATCACTTCGATTCCATTAGTAAGGAGGATTCAGAATATCACACATTGATC

GATCAAACAG--------------------------------------------------

------------------------------------------------------------

------------------------------------------------------------

-------AGATTCCGCAAC---------TAAAAGGAAGATCGATTCCTGGGGATC---CT

TCCTTTCTTCAAACGGAACGAAC------AGAGATAGAATCAGATCGATTCCCGAAATGC

CTTTCTGGATCTTCCCCAATGTCCCCG---------CTATTCCCCGAACATGAGAAGCAG

ATGATTATTCATCGGCTTCCG------GAAGAAATCGACGAATTTCTTGGGAATCCTACA

AGATCAATTCGTTCTTTTTTCTCTGACAGATGGTCA------GAACTTCATATG---GGT

TCGAATCCTACTGACAGGTCCACTAGAGATCAGAA------------ATGGTTGAAGAAA

CAACAAGATG---TTTCTTTTGTCTCTTCCCGGCGATCAGA------------AAATAAA

GAAATGGTTGATATATTCAAGATAATTACTTATT------TACAAAATACCGTCTCAATT

CATCCT-----ATTTCATCAGATCCGGGATGTGATATG---GTTCCGAAGGATGAACCAG

AT---ATGGACAGTTCCAATAAGATTTCATTCTTGAACAAAAATCCATTTTTTGATTTAT

TTCATCTATTCCACGATCGGAACAAGGGGGGGTACACATTACACCACGA---TTTTGAAT

CAGAAGAGAGATTTCAAGAAATGGCAGATCTATTCACTCTATCAATAACCGAGCCGGATC

TGGTGTATCATAGGGGATTTGCCTTTTCGATTGAT---------------TCCTGCGGAT

TAGATCAAAAAAAATTAGTGA------ATGGAAAGGAAAAAACAAAGGATGAATCGAAAA

AGAAATCTTTATTGGTTCTACCTCCTCTTTTTTATGAAGAGAATGCCTTTTTTTATCGAA

GCATCAG-AAAAAAACCGGTCTGGATCCACTGCGGGAATGATTTG---------------

------------TTTGCTAGCAACAACATAATGGAGGCAGTCAATCAATATAGATTGATC

CGAAATCTGATTCAAATCCAATATAGCGCCTATGGGTACATAAGAAATGTATTGAATCGA

TTCTTTTTAATGAATAGATCCGATCGCAACTTCGAATATGGAATTCAAAGGGAT---CAA

ATAGGAAATGAT----ACTCTGAATCATATAACTATAATGAAATATACGATCAACCAACA

TTTATCGAATTTGAAAAAGAGTCAGAAGAAATGGTTTGATCCTCTT----ATTTCTCGAA

CCGAGAGATCCATGAATCGGGATCCTAATGCATATAGATACAAATGGTCCAACGGGAGTA

AGAATTTCCAGGAACATTTGGAACATTTCGTTTCTGAACAGAAGAACCGTTTT-------

--CAAGTAGTGTTTGATC------AATTACG-----------------TATTAATCAATA

TTCGATTAATT-GGTCCGAGGCTATCGACAAACAAGATTTGTCTAAGTCACTTCGTTTCT

TT--TTGTCCAAGTCA--CTT------------------------CTCTTTTTGTC----

---------------CAAGCCACTTCGTTTCTTTTTGTCCAAGTCACTTCCTTTGTTCTT

TGTGAGTATCGGGAATAGCCCCATTCATAGGTCCGAGATCCACATCTATGAATTGAAAGG

TCCGAATGAGCCG---CTCT---------------------GCAATCAGTTGTTAAAATC

AATAGGTGTTCAAATCATTCATTTGCATAAATTGAAACC------------------TGA

TCATGATA--CTTCCCAAAGATCGAAATTCTTGATCAATGGAGGAACAATATCACCATTT

TTGTTCAAT-AAGATACCAA------AGTG-GATGATTGACTCATTCCATACTAGAAAGA

ATCGCAGCAAATCCTTTGCTAACACGGATTCCTATTTCTCAATGATATCCCACGATCGAG

ACAATTGGCTGAATCCC-GTGAAACCATTTCATAGAAGTTCATTGATATCGTCTTTTTAT

AAAGCAAATCAACTTCGATTCTTGAATAATCCACATCACTTCTGGTTCTATTGTAACAAA

AGATTCCCCTTTTATGT---GGAAAAGACCCGTATCAATAATTATGATCGTACATATGGA

CAATTCCTCAATATCTTGTTCATTCGCA-ACAAAATATTTTCTTTGTGCGTC------GG

TAAAAAAAAACA---------------------TCTTTTA------CCGATTTATTCACA

GGTATCTGACATATTCATACCTAACGATTTTC------CACAAAGTGGTGACGAAACG--

----------TATAACTT---------GTACAAATCTTTCCGTTTTCCAATTCGACCCGA

TCCATTCGTTCGT------AGAGCTCTTTACTCGATCGCAGACATTTCTGGAACACCTCT

AACAGAGGAACAAATAGTCAATTTTGAAAGAACTTATTGTCAGCCTCTTTCAGATATGAA

TCTATCCGATTCAGAAGGGAAGAACTTGCATCAGTATCTCA------GTTTCAATTCAAA

CATTGGTTTGATTCACACTCCATGTTCTGAGAAAAATTTACCATACGTAA---------A

----------------GAGGAAA-------------------------------------

--AAACAGAGTCTTT----ATCTGAGGAAAAAACGGAGTC--------------------

------TTTATCTAAAGA------------------------------------------

--------AATGCGTTGAGAAAGGGCAGATGTATAGAACCTCTCAACAACGGGATAGTGC

TTTT---------TCAAAATGGAATCTGTTCCAAACATATATGCCATGGTTCCTTACTTC

GACAGGGTGCAAATATCTAACTTCCGTCCTTTTAGATATTTTTTCAGACCGATTGTCGAT

ACTAAGTAGTAGTCCAAAATTGGTATCCATTTTGAATTCTATTATGCA------------

TAGATCAGATATATCACGGCTAATT------AAAAAATGGTGGACGATTCTTCCACAAT-

----------GGAATCTGATAAGTGA----------------------------GATTTC

GAGTAAGTG------TTTACAGAA---TCTT--------CTTCTGTCCG-AAGAAATGAT

TCATCGAAATAATGAGTCACCCGTTTCAATGATATTGACACATCTGAGATCAACAA--AT

GCTCGGGAGTTCCTCTATTCAATCCTTTTCCTTCTTCTTGTTGCTGGATATCTCGTTCGT

ACGCATCTTCTCTTTGTTTCCCGAGTCTCTAATGAGTTACAGACAGAGTTAGAAAAGATC

AA----ATCTTTGAT---------GATTCCATCATAC-ATGATTG---------------

---------------------------AGTTG----------------------------

-------------------CAAAAACTTCTGTATAGGTATCCTACATCTACATCTAAATC

TGAATCTGAATCTGAATCTGAATATGAATATGAACTGAAT--------------------

----TCTTT--CTTGTTAAATAATCTCTTT------GCTCTGG-----------------

--------AACAATTAG-------------------------------------------

------------------------------------------------------------

--------------------------------------------ATTGGAAT--------

----------ATCGATCTCATAAGTATCATATCAAATACCA---------TCACTTTTT-

-------------CGAGAAATACGAGACATCTAAGTCGTA--------------------

------------------------------------------------------------

CAAG------------------------------TAAAGAGATCTA------TTCATTGA

TAAGAGAAAGAAAAAA--CGTGAACAGTGATTGGATTGAAGACAGAGT------------

-------------------------AGAATCCTGGGTTACGATCAATGATTTGA------

---------------TTGAT----------------------------------------

-----GAGGACGAAAGAGACTTGGCGGTTCAGTTCTCCACCTTAACGACCGAAAAAAGGA

TTGATCAAATTCTATGGAGTCTGACTCATAGTCATCCTGTATCAAAGAATGATCTCGATT

ATCAAATAATTGAACAACCGGGATTAATTTCCTTACGATACTTAGTTGACATTCATAAAA

AGGATCTAATGAATTATGAGTTCAATGGATCCTGTTTAGCAGAAAAACGGATATTCCTTG

CTAATTATCGGACAATCACTTATTC------ACAAACCCCAT------------------

------------CTCGTGGAAAACCCTTTTCACTCCGCTTAGACCTATCCCCCTCAAGGG

GTATTTTAGTGATAGGTTCTATAGGAACTGGACGATCACATTTGGTCAAATACCTAGCGA

CAAACTCCTATGTTCCTTTCATTACGGTATTTCCGGACAGGGGCCTGTCTGACAGGCCTA

TATATTGTTTTATGGATGAG---------------GATACCGATCCTGATGAGGATTATG

------------------------ACTATATTGATGATGATATCGATATCGATAGTGATA

ACTTTGATAC------GGAGCTGGAAACTATCGCGGATGTGGTAACTATGGATAATATGC

TGCTGAA---AGTAATCC------GATTTGATATCCTCGGTCAATTCGAATTAGCAAAAA

CAATGTCTCCTTGCATAATCTGGATTCCAAACATT----------CATGATCTGT-----

------------------------------------------------------------

---------------ATTTGAATGA---------------------------GTCGAATT

ACTTATCCC-----------TCGGTCTATTAACGAACTTTCTCTCCAGTCTCTACGGGG-

------ATTGTGAAA------CCACT------AGAAATATTCTTGTTTTTGCTTCGACTC

ATCTTCCCCAATTAGTGGATCCCGTTCTAATAGATCCGAAGAAATTCAATACATGCATTA

AGATACGAAGGCTTCTTAGTCCACAACAACGGAAGCACTTTTTCAATCTTTTATATACTA

GGGGATTTCACTCGGAAAATAAAATGTTCCATGCTAACGGATTCGGGTCCATAACCATGG

GTTCCAATGCACGAGATCTTGTAGCACTTACCAACGAGGTCCTATCAATTAGTATTACAC

AGAAGAAATCAATTCTAGAAACTAATACAATTAGATTAGCTCTTCATAGACAAACTTGGG

ATTTGCGATCC---------CATG---TAAGATCGGTTC---CGGATCATGGGATCCTTT

TCTATCAGATAGGAAGGGCTGTTGCACAAAATGTACTTCTAAGTAATTGCTGCACAGATC

CTATATCTATCTATATGAAGAAGAAATCATGTAATGAAGGGTTTTCTTATTTGTACAAAT

GGTACTACGAACTT---------GGAACGAGCATGAAGAAATTAACGATACTTCTTTATC

TTTTGAGTTGTTCTGCCGGATCGGTCGCTCAAGATCTTTGGT------CTCCACCCGGAC

CCGATGAAAACAGTTGGATCACTTCTGATAGATTCGTTGAGAATGATTCTGATCTAGTTG

ATAGCCTA---------------TTAGAAATAGAAGGC------GCTCTGGTAGGATCCT

GGC---------------------------------------------------------

---------------------TTCTTCGGTCCGAACCAAGGAATCCGTTAGATATGATGC

AAAAAAGATCTTGTTCTATCTTT------------------AAAAAAGACGAATCGGAGT

TTGCAGAAGGG------GAAGGAGCCCTCGCCCTCGACCCG------------------C

AAGAGATGCTTTTCTACAATCAAATAGTTTGGGCTCCTAGACTATGGCGCCCT------C

CTTGTGGCAAACTATTTGATTGT-------------------------------------

-----ATCGAATATTCTCAAG---------------------------------------

------------------------------------------------------------

------------AGGATGATG-------------------------------ATGATTGG

GAGTTCTGGCAGAGT----------------GGAA----CCAAGTATTACTGGATGCGAG

ATAG------------------CGAGGGAGGACAGGGCTTTTGGGTTTCTCAACTAAAAA

AATTCCTTTGGAACCCTGAGGCTGCGGATCCATTCCTTTTCCTATTCAAAGATCAGCCCT

TTGTCTCTGATTCTGATTTTTCATCATATCATCAACCAT------TCTCCAAT------T

TTTTGGTAGAGCTGATGCCTCC--------------------------------------

----------TTTC----------------------------------------------

------------------------------------------------------------

------------------------------------------------------------

------------------------------------------------------------

--------------AAAGCTTCTTCTGTATCTCTATATAAAGGCTGGGTCAACAAGAAGT

TGCAAGAAATG------TGCCTCGAATACTTGAGTGATCGCG---AGAGATGGCTTAGA-

-----ACCAATAGTTCATTATCTCATGGATCTTTCCGTTCTAATACTCTATCCGAGAGTT

ATCAGTATTTAGCAAATCTGTTCCTATC---TAACGCAACGCTATTGGATCAAATGACAA

AGACATTGTTGAGAAAGAGATGGCTTTTCCCGGATGAAATGAAAGATTTGATTCATGGAA

CAGGAGGTGGA-------------------ACAGAATTGACCGGGCGGTAG---------

--------ATGCCTAGATCTCGAATAAATGCAAATTTTATTGACAAGACCTCTACAATTG

TAGCCAATATCTTATTACGAATAATTCCGACCACTTCAGGAGAAAAAGAGGCATTTACTT

ATTACAGAGATGGTGCGATTTGATTC--TTTTTTTTTTTTTTTTTTTTTTTTAGCCCTCA

CC--------------------------CCAGTCTTAC--TAAAAAGAG-ACAGGATTCG

G------GATAT-----A-AAAAAAAGATGATGAAAAA----TAAACCTGCGCTTGGTGA

AGGTGAAGT-----TTGAAGATCGAACAATTCCTTCTATCGTGTATCCTCGATTGATGCA

GCCTCAGATACTTCAAT----TGTCGATTCTAGTATTGAGCAAAAGGTTACGCCTATA--

----GGT----TCTGT-ATTG-----CGGGTCAATCCTACTCCC-C----GAATACAAAT

AGGC-GGCATAG----GGGA-ATAAGCACTACACC------T-----AGGAATCAACAAC

ACGAAAACTTTGTTATA---------AATTACCTTTT---CCTTATCGGGATTGGAACTA

ACAA-------------GAATGGTTGGGACAACAAGCATCCATCTCGTTCGTACTTTGGA

TACCCGTAT-----AACCATCGAAGATCGTTGAAGTGACTAATTC--CT-GTAAATAGGG

GGCGTTGAGGACAAAGAAATTGTTGG----AGTTATCATTTCTATCT----AGCACCAAG

ACGGTTGGT--GT------------------TAAGAAAGGACCTCTTGCAGGAAGGATGG

CTAGAGATTTCGTGTAAAAA-TACCAGCCCCTGTATGTTCATAAAA--TAATGAGAATAT

TTCATTCTTT----TTCGATTCCATG-------------------------GATTATTCT

CCTCATTAC---TATGCACAGAA-GGGAGGAGCCATATGAGATGAAAATCTCACGTACGG

TTCTGGAACGGAGATTCTTTGA-ATAAAATAAACGAAAACGACCGTAACGGATGTCAGCC

CAATCCGAAGGAAATTATGCGGAAGCTTTACAGAATTATTATGAAGCTACGCGACCAGAA

ATTGATCCCTACGATCGAAGTTATATACTTTATAACATAGGCCTTATACACACAAGTAAC

GGAGAACATACGAAGGCTTTGGAATATTATTTCCGAGCACTAGAACGAAATCCATTCTTA

CCGCAAGCTTTTAATAATATGGCCGTGATCTGTCATTACGTGCGACTATCTCCACT----

--ATAG------------------------------------------------------

-------------------AAAG-------AAGGAAATAA-AGATCAAATCCGCTAGTA-

-----------------ACT-ACTTACTAGAAAC----------------AAAATAGGCG

TTCTA-CATATGCATTGTCCGAA--GCAACGATTTT----TATCAGCTGTAGCAAAGAAA

GATA------CTTCACGGTAGCC-AAAATAGGA--AGAAATAAGTAG-GCCTGTATACTA

TATTCTATGGAT-----AAAG--GATCGAATTGATAGAGGAAGCGCCGTAAAGATCAATT

AGCGAGGTT---TT-GGGCCGATACAA-T-----AAAAACTGCTTACT----TATCTCAT

GA-TACGAGATA---AAAGTTAGG----AATCAACTTATGTAATAGAGTTGATCCAC---

----TAAAGTACTGAGCAGCGGCGTAGCATCAGATCCCAAAGAT-AGTAAGTCCTTTCTT

TCTTAT--------GAAG---------------GAAAGTCTTTTTC-AAAGATTCTAT-A

TTAAT-----------------CAATTTCTATAT---------GAAACCGAGATAGTTAC

CT-TTCAGAAA-------ATTATAACGATAGAG------GTAGATACTTAT------GCT

TCATTTTTCTGAAGGTGGGAGAAAAGATAAAACTGA-TTATTCATC-----AAAA-TTAG

AGTTTCAAACTT-ATGTAATTAAC-----TTCTTCTGGTTA-AC--------CTAAG---

AACTGGGATAGAT------TTCTTAGAAATCTCAC------------AAATAGGGTAGAT

T-----AGGATGGGAC----GCCAAAAGAATTGATGGCGGAGCCGTATGAGATAGGAAAC

TCTCAAGTACGGTTCTAAGGGAAGGAATTGACCC-------ACCTATTCCGACCGGGGAG

AACAGGCCATTCGACAGGGCGATTCTGAAATTGCGGAGGCTTGGTCCGATCAAGCTGCTG

AGTATTGGAAACAAGCTATAGCTCTTACTCCAGGTAATTATATTGAAGCGCAGAATTGGT

TGAAGATCACAAGGCGGTTCGAA------TAAATGAACTGGCGATCAGAACATATATGGA

TAGAATTTATAACGGGGTCTCGAAAAACAAGTAATTTTGGCTGGGCCTGTATCCTTTTTT

TAGGTTCACTAGGATTCTTAGTGGTTGGAGCTTCCAGTTATCTTGGTAGGAATCTGATAT

CTGTATTCCCATCTCAGCAAATAGTTTTTTTTCCACAAGGGATCGTGATGTCTTTCTATG

GGATTGCAGGACTGTTCATTAGCTCCTATTTGTGGTGCACTATTTCGTGGAATGTAGGTA

GTGGTTATGATCGATTCGATAGAAAAGAAGGAATAGTGTGTATTTTTCGTTGGGGATTTC

CTGGAATAAATCGTCGCATTTTCCTTCGATTCTTTATAAGAGATATCCAGTCTATTAGAA

TAGAAGTTAAAGAGGGTCTTTATTCT---CGTCGTGTCCTTTATATGGAAATCAGAGGTC

AGGGAGCGATTCCCTTGACTCGTACTGATGAGAA---TTTGACTCCACGAGAAATTGAAC

AAAAAGCAGCTGATTCAGCCTATTTCTTGCGTGTCCCAATTGAGG------TATTTTGA-

------------------------------------------------------------

------------------------------------------------------------

--------------------------------

>Alstroemeria

ATGGAAAAA---TGGTGGGTCAATTCGACGTTGTTTAACGAAAAATTAGAACATAGGTGT

GGGCTAAGTAAATCAATGGATAGTTCTGATGCTATTGGACATACCAGTGGAAGTGATGAA

TCCGTTCTAAATGGTACGGGGAAAGACATTCCTAATTGGGGTAATAGTGACAGTTATAGT

TTCAGTAATGTTGATTA---------TTTATT---------TGATATCAGGAATATTTGG

AGTTTGATCTCTGATGATACCTTTTTAGTTAGGGATAGTAATGGTGACAGTTATTCTCTA

TATTTTGATATTGAAAATCATATTTTTGAGATTGACAATGATAGTTCT------------

------------------------------------------------------------

---------------------------------------------------TTTCTGAGT

GAACTAGAAAGTGTTTTTCTAATTAT----------------------------------

-----------------------------------TNTGAATAGTGGGTCTAAGAGTAAC

AATTACTACTA---TTATCCTTACATGTATCATACTCAATTTAGTTGGAATAA------T

CACATTAATAGTTGCATTGATAGTTATCTTAATTTTGAAGTC------AGTATTAATAGT

TCTATTTGGGGTCGTACCCACAATTACAGTGACAG------TTATATTTCTCGTTTTAT-

-----------------------------TTGTACTGAAAGTGTAAGTGATAGCGAAAGC

G---------------AGAGTTCTAATATAAAAACGAATAG---TAATGGCAACG-----

-ATTTCAATATAAGAGAAAGATCTAATGATTTCGATATTGATATAAGTCAAAAATACAGA

CATTTATGGGTTCAATGCGAAAATTGTTATGGATTAAACTATAAAAAATTTTTTAGGTCA

AAAATGAATATTTGTGAACAGTGTGGATATCATTTGAAAATGAGTAGTTCAGATAGAATC

GAACTTTCGATTGATCCGGGCACTTGGAATCCTATGGATGAAGATATTGTTTCT------

------------------ATGGACCCCATTGAATTTC-----------------------

-------------ATTCAGAGGAAGAGCCTTATAGAGATCGTATCGATTCTTATCAAAGA

AAGACGGGTTTAACTGAAGCTGTTCAAACAGGCATAGGTCAACTAAATGGTATTCCTATA

GCAATCGGGGTTATGGATTTTCAGTTCATGGGAGGTAGTATGGGATCCGCGGTCGGCGAG

AAAATTACCCGTTTGATCGAGTATGCTACTAATCGATCTCTGCCTGTCATTATTGTGTGT

GCTTCGGGAGGAGCACGCATGCAAGAAGGAAGTTTGAGCTTGATGCAAATGGCTAAAATA

TCTTCTGCTTCATATGATTATCAGTTAAATAAAAAGTTATTCTATGTATCAATTCTTACA

TCTCCTACAACCGGTGGAGTAACAGCCAGTTTTGGTATGTTGGGAGATGTTATTATTGCT

GAACCAAATGCCTACATTGCGTTTGCGGGTAAAAGAGTAATTGAACAAACATTGAATAAG

ACAGTACCCGAGGGTTCACAAGGGGCTGAGTATTTATTCCATAAGGGCTTATTCGACCCA

ATCGTACCGCGTAATCCTTTAAAAGGTGTTCTGGGTGAGTTATTTCAACTCCATGGTTTC

TTT----------------CCTTT------------------------------------

------------------------------------------------------------

------------------GAATCCAAGTTCAACAAATGAAAATTAA--------------

-ATGGCAACCCTTCGAGCCGACGAAATTAGTAACATTATCCGTGAACGTATTGAACAGTA

TAATAGAGAAGTCAAGATTGTAAATACTGGTACCGTACTTCAAGTAGGCGATGGAATTGC

TCGTATTCATGGTCTTGATGAAGTAATGGCGGGTGAATTAGTAGAATTTGAAGAGGGTAC

TATAGGGATTGCTCTGAATTTGGAATCCAATAATGTTGGTGTTGTATTAATGGGTGATGG

TTTGCTGATACAAGAGGGAAGTTCTGTAAAAGCAACAGGAAGAATTGCTCAAATACCTGT

GAGCGAGGCTTATTTGGGTCGTGTTATAAATGCCTTGGCTAAGCCTATTGATGGGAGAGG

GGAAATTTCAGCTTCTGAATCTCGGTTAATTGAATCTCCTGCCCCAGGTATTATTTCAAG

ACGTTCCGTATATGAGCCTCTTCAAACTGGGCTTATTGCTATTGATGCGATGATCCCTAT

AGGGCGTGGTCAGCGAGAATTAATTATTGGGGATAGACAGACTGGCAAAACAGCAGTAGC

TACAGATACGATTCTCAATCAAAAAGGGAAAAATGTAATATGTGTTTATGTAGCCATTGG

TCAAAAAGCGTCTTCTGTAGCTCAAGTAGTGACTACTTTCCAGGAACAGGAGGCAATGGA

ATATACCATTGTGGTAGCCGAAACCGCGGATTCACCTGCTACATTACAATACCTTGCTCC

TTATACAGGAGCGGCTCTGGCTGAATATTTTATGTACCGTGAACGGCATACTTTAATAAT

TTATGATGATCTCTCCAAACAGGCACAAGCTTATCGCCAAATGTCTCTTCTATTAAGAAG

ACCCCCCGGTCGTGAAGCTTATCCGGGGGATGTTTTTTATTTGCATTCACGTCTTTTGGA

AAGAGCCGCCAAATTAAGTTCTCGTTTAGGCGAAGGAAGTATGACCGCTTTACCCATAGT

TGAGACTCAATCTGGAGACGTCTCGGCTTATATTCCTACTAATGTCATTTCCATTACGGA

TGGACAAATATTCTTATCGGCCGATCTTTTCAATGCTGGAATCCGGCCCGCTATTAATGT

GGGTATTTCTGTTTCCAGAGTAGGATCCGCAGCTCAAATTAAAGCCATGAAACAAGTAGC

CGGCAAATTAAAATTGGAACTAGCCCAATTCGCGGAGTTAGAAGCCTTTGCGCAATTTGC

TTCTGATCTCGATAAGGCTACTCAGAATCAATTGGCCAGAGGCCAACGATTACGCGAATT

GCTCAAACAATCCCAATCAGAACCTCTCGGGGTGGAAGAACAGGTACTTACTATTTATAC

CGGAGCGAATGGGTATCTTGATTCGTTAGAAATTGGCCAGGTAAAGAAATTTCTTGTTCA

GTTACGCACCTACTTTAAAAATAATAAACCTGAATTCCAAGAAATTATATCTTCTACCAA

GACATTCACCAAAGAAGCGGAAGCCCTTTTGAAGGAAGTTATTCAGGAACAGATCGAACT

GTTTCTACTTCAGGAACAAACA---------------------TAAATGAGAATCAATCC

TACTAC------TTCTGGTC------CTGCGGTTTCTACACTTCAAGAAAAAAACCTAGG

GCGTATCGCTCAAATTATTGGCCCAGTACTGGATGTTGTTTTTCCCCCGGGCAAGATGCC

TAATATTTATAACGCTTTGGTAGTTAAGGGTCGAGATACTGTGGGCCAACAAATTAATGT

GACTTGTGAGGTACAACAATTATTAGGAAATAATCGAGTTAGAGCTGTTGCTATGAGTGC

TACAGACGGTCTGACGAGAGGGATGGAAGTGATTGATACAGGAGCTCCTTTAAGTGTTCC

AGTTGGTGGATCTACTCTCGGACGAATTTTCAACGTTCTTGGAGAGCCTGTTGATGATTT

GGGTCCTGTAGATACTCGCACAACATCTCCTATTCATAGATCTGCGCCTGCCTTTATACA

GTTAGATACGAAATTATCAATCTTTGAAACAGGAATTAAAGTGGTGGATCTTTTAGCTCC

CTATCGCCGCGGAGGAAAAATCGGACTATTTGGGGGAGCTGGGGTGGGTAAAACAGTACT

CATCATGGAATTGATCAACAACATCGCCAAGGCTCATGGGGGCGTATCCGTATTTGGCGG

AGTAGGCGAACGAACTCGCGAAGGAAATGACCTCTACATGGAAATGAAAGAATCTGGAGT

GATTAATGAAAAAAATATTACAGACTCCAAAGTAGCTCTAGTCTATGGTCAAATGAATGA

ACCGCCGGGAGCTCGTATGAGAGTTGGGTTGACTGCCCTAACCATGGCAGAATACTTCCG

GGATGTTAATGAGCAAGACGTGCTTCTATTCATCGATAATATCTTTCGTTTCGTCCAAGC

AGGATCAGAAGTATCTGCCTTATTAGGGAGAATGCCTTCCGCTGTGGGTTATCAACCTAC

CCTTAGTACAGAAATGGGTTCT---TTGCAAGAAAGAATTACTTCTACCAAAGAGGGATC

CATAACCTCGATCCAAGCAGTTTATGTACCTGCAGACGATTTGACCGACCCGGCTCCTGC

CACGACATTTGCACATTTAGATGCTACTACCGTACTATCGAGAGGATTAGCTGCCAAAGG

CATTTATCCAGCGGTGGATCCTTTAGATTCAACGTCAACTATGTTACAACCTCGGATCGT

TGGTGAGGAACATTATGAAATTGCGCAAAGAGTTAAGCAAACTTCACAACGTTACAAAGA

ACTTCAGGACATTATAGCTATCCTTGGGTTAGACGAATTATCCGAAGAGGATCGTTTAAC

TGTAGCAAGAGCACGAAAAATTGAGCGTTTCTTATCACAACCCTTCTTCGTGGCAGAAGT

ATTTACGGGTTCCCCCGGAAAATATGTGGGTCTCGCAGAAACCATTAGGGGATTCCAACT

CATCCTTTCCGGAGAATTAGACGGTCTTCCCGAGCAGGCTTTTTATTTGGTGGGTAACAT

CGATGAAGCTACCGCGAAAGCTATGAACTTAGAAGAGGAGAGCAAATCGAAGAAATGAAT

GACCTTAAATCTTTGTGTACTGACTCCTAATCGAATTATTTGGGATTCAGAAGTGAAAGA

AATCATTTTATCTACTAATAGCGGCCAAATTGGTGTATTACCAAATCACGCCCCTATTGC

CACGGCCGTAGATATAGGTCTTTTGAGAATACGTCTC---AACGACCAATGGTTAACTGT

GGCTCTAATGGGTGGTTTCGCCAGAATAGGTAATAACGAGATCACTATTTTAGGAAATGA

TGCGGAGATGAGTACTGACATTGATCCCCAAGAAGCTCAACAGGCTCTTGAAATAGCTGA

AGCTAACTTAAGCAGGGCTGAGGGTAAGAAACAAGCAATTGAGGCAAATCTAGCTCTCAG

ACGGGCTAGGACACGAGTAGAGGCCACCAATG---------CAATT------------TC

CTACTAGATGAAAAATGTAACCGATTTTTTTGTTTCCTTG---GGCTATTGGCCATCTGC

GGGGAGTTTCGGGTTTAATACCGATATTTTAGCAACAAATCCAATAAATCTAAGTGTAGT

ACTTGGTGTGTTAATTTTTTTTGGAAAGGGAGTGTGTGCGAGTTGTGTATTTCAAGAATA

-GGTTGGATCCAGCCAGCTGCACTTTA---------------------------------

------------------------------------------------------------

----------------------------TTTTTTATTTATTTT-----------------

------------------------------------------------------------

------------------------------------------------------------

------------------------------------------------------------

------------------------------------------------------------

------------------------------------------------------------

------------------------------------------------------------

------------------------------------------------------------

---------CTATTTTAAATAGAAATAGGA----A-------AAAGTGCATGATCTCG-A

CGAATTACTTCTGAATA----------AATTCAGA----AATC-----ATATGCAAGAAC

CATAGCATTTCGTGACTTATTGGT-----AAATCAACCTTGA-----TTCTCTATCAA--

---CCGATAATAATAATGCGCAACTTTTCACATGGTTAAAGCT-AAACTGTTTGAAGTCC

AGGC--ACAACAT-----GGTATTCTTTCTACCACTAAGTAAGTA------CCGAAAGAA

ATGG-----TAAAAAAA----------------AAGAGTTGGTAATTCATACGATATACA

ACACTCATATCGATAGAATGATT-TGAA----ACCATTTACCAG----------------

--AA---AAATGGGTCGTCTTGCCCTTTTT---TTTTCTAATGCCAAATTGACAACCTAT

GTAT----------AAAA--------AAGAGGAA--TTCTTTGGATTTTCATATTTGAAC

AAAAATGGA------------------------------------------------AAA

TAAAAAA-----------------------------------------------------

-ATCTATAATATTATTTCATTTCTA--------------------TTTTTCATAGAAATG

AAAT----------------------------------------------------AAAA

AACAAATAAAGAAACAACTTTGCTGACAATTATACATTTTTT--GTCTGGTCAGAAGAAT

CC-----------TCC--TACCCTAATATTCTGGTATT---------ATATTAGTTT---

-----------------------------------------CAATATCTTTGAATAGGAA

CAAAAAAG-------AGAGGGCAGGCTCATTACAT-----TAAAAGATATGGGAATA-CC

CATT------------AACTAAATAATTCTAATTGAGCGTGAGAGCCAAATGAATCGAAA

GATTCATGTTTGGTTCGGGAAGAGATCA-TGGGAGTTGTGAAAT-----TAATGGTAAGA

TAATCTACTTTCATTAAATGATTTATTAGATAATCGAAAACAGAGGATCTTGAGTACGAT

TCGAAATTCGGAAGAATTACGGAAAGGAGCCCTTGAGCAACTCGAAAGAGCTCGAGCTCG

CTTACGGAAAGTGGAAATTGAAGCAGATGAGTATCGAATGAATGGATACTCTGAGATAGA

ACGAGAAAAGGAAAATTTAATTAATGCTACTTGCGATAGTTTGGAACGATTAAAAAATTA

CAAAAATGAAACCCTTCATTTTGAACAACAAAGAGCGGTTAATCAGGTTCGACAACGAGT

TTTCCAACAAGCCTTACAAGGAGCTCTAGTAACTCTGAATAGTTTTT------TGAATAA

TGAGTTACATTTCCGTACCATCAGTGCTAATATCGGCGCTCTTGGGGCCATGAAA-----

GAAAAAATGGATTAGATGAATCCACTGATTTCTGCCGCTTCCGTTATTGCTGCTGGATTG

GCTGTAGGGCTTGCTTCTATTGGACCTGGAGTTGGTCAAGGTACTGCTGCGGGCCAGGCT

GTAGAAGGTATTGCGAGACAACCAGAGGCAGAGGGGAAAATACGAGGTACTTTATTGCTT

AGTCTAGCTTTTATGGAAGCTTTAACAATTTACGGGCTGGTCGTGGCCTTAGCACTTTTA

TTCGCAAATCCCTTTGTTTAAATGAACGTTATACCATGTTCCATTAAAACACTCAAGGGG

TTATACGATATATCGGGTGTCGAAGTAGGCCAACATTTATATTGGCAAATAGGAAGTTTA

CAAATCCATGCCCAAGTACTTATTACCTCTTGGTTCGTAATTGCTATCTTATTGGGTTCA

GCCATCATAGTTGTTCGGAATCCACAAACTATTCCGACCGACGGCCAGAATTTCTTCGAA

TATACCCTTGAATTTATTCGAGACTTGAGCAAAACTCAGATTGG---AGAAGAATATAGT

CCTTGGGTTCCCTTTATTGGAACTATGTTCCTTTTTATTTTTGTTTCTAACTGGTCAGGC

GCTCTTTTACCGTGGAAAATCATACAGTTACCTCATGGGGAGTTAGCTGCGCCCACGAAT

GATATAAATACTACTGTTGCTTTAGCTTTACCCACGTCAGCGGCATATTTTTATGCCGGT

CTTACCAAAAAGGGATTGGATTATTTCGGGAAATATATTCAACCGACTCCAATACTTTTA

CCAATTAACATCCTAGAAGATTTCACAAAACCCTTATCTCTTAGTTTTCGACTTTTCGGG

AATATATTGGCGGATGAATTAGTAGTTGTTGTTCTTGTTTCTTTAGTACCTTCAGTAGTT

CCTATACCTGTTATGTTTCTTGGATTATTTACAAGTGGTATTCAAGCTCTTATTTTTGCA

ACTTTAGCTGCGGCTTATATAGGCGAAGCCATGGAGGGTCATCATTGAATGATCTTTGCA

GCTTTAGAACATATATTAACCCATATCTTTTTTTCGATCATTTCAATTGTCATTATGGTT

CATTTGATGAACTTATTAATCCGCGAAATCGTAGGATTACGTGATTCCTCGGAAAAGGGT

ATGATAGCCACTTTTTTCTCTATAACAGGATTACTAGTTACTCGTTGGATTTATTTGAGA

CATTTTCCCTTAAGTAATTTATATGAGTCATTAATTTTCCTTTCGTGGGGTTTCTGCATT

ATTCATATGATTCCTAAGATACATAATCTTAAAAACG------ATT---TTCTTGCAATA

ACTGCGCCAAGTGCTATTTTTACTCAGGGTTTCGCCACGTGGGGTCTTTCAATTGAAATG

CATCAATCCGCAATATTGGTACCCGCTCTACAATCTCAGTGGTTAATGATGCACGTAAGT

ATGATGTTATTGAGCTATGCAGCTCTTTTATGCGGATCATTATTATCGGTCGCTCTTCTA

GTCATTACGTTTAGACAAAATATCTATATTTTTTC------CAAAAGC---AATAATTTC

TTAATT------AAGTCATTTTTC---------TTTGATGAGATTAAATACTTGTACTTG

AATGCAAA---AAGA---------CGTGTTTG---TACAAACACGTCTTTT---CTTTTA

TTTCGGAATTATTACAAATATCAATTGACCCAGCGTTTGGATTATTGGAGTTATCGTGTC

ATTAGTTTAGGGTTTACCTTTTTAACCATAGGAATTCTTTCTGGAGCAGTATGGGCTAAC

GAGGCATGGGGCTCCTATTGGAATTGGGACCCAAAAGAAACTTGGGCATTTATTACTTGG

ACCATATTCGCCATTTATTTACATACTCGAACAAATAAAAGTTTACAAGAT------CTG

AATTCAGCACTTGTGGCTTCTATAGGATTTCTTATAATTTGGATATGCTATTTTGGGGTA

AATCTATTAGGAATAGGTCTACATAGTTATGGTTCATTCAATTAGACGTTA-ATATTCAT

ATTAACGAACCACACGAAAAATACATAA--------------------------------

------------------------------------------------------------

------------------------------------------------------------

------------------------------------------------------------

----------ATGAAAAAAAAGAAAGCATTGACTTCCCTCCCCTATATTGTAGCTATAGT

ATTTTTGCCCTGGTGGGTTTCTTTCTCATTTGAGAAAAGTCTGGAACCTTGGGTTACTGA

TTGGTGGAATACCCGGCAATCAGAAATTTTTTTGAATAATATTCAAGAGAAAAATGTTCT

AGAAAGATTCATAGAATTAGAAGAACTTTCCCTGTTGGACGAAATGATAAAGGAGTACCC

GGAAACACATATACAAAAACTTCGTATAGGGATACACGAGGAAACGATACAAT-TGGTAA

AAACACAGAATAAATATTCTTTCCATATCATTTTGCATTTCTCGACAAATATAATATGTT

TCGCTATTCTAAGTGGTTATTTTATTCTGAGTAATGAAGAACTTATCATTCTGAATTCTT

GGGCTCAAGAATTCCTCCATAACTTGAGTGACACAATAAAAGCTTTTTCGATTCTTTTAG

TTACTGATTTATGGATCGGATTTCACTCGACCCATGGTTGGGAACTTATGATTGGTTCGG

TCTACAACGATTTTGGGTTGGCTCATAACGATCAAATTATATCTGGCCTTGTTTCCACTT

TTCCAGTTATTCTAGATACCATTGTGAAATATTGGATCTTCGATTATTTAAATCGTGTAT

CTCCCTCACTTGTAGTTATTTATCATTCAATGAATGA---ATGAATGCCCATTGGTGTTC

CAAAAGTACCTTTTCGGAATCCCGGAGAG---GAAGATGCGGTTTGGGTTGACATCTAGT

GCGACTTGTCGGACATATTTGGCCATATGGGATTCA-CCCGTTATCTC-----CCCCGAT

CGAGATATCCTCTGTTTCGCCCAA----------------CAACTATTT-------ATTG

ACTGAACTATCAAT----------CATTCGGAGCGTGAA--ATTCAATTAGATCAATTAA

TTTTGGGGATCAATATT----ATCAA-GCAGAAAATTTTATGGTTAACTTGGACTAATAA

AATAGAAAATAGAG-----TATCCAGGCTCCGTTCAGAAAATACCAAAT-GGTAATAT--

------------------------------------------------------------

------------------------------------------------------------

---------------------ATTAATATTATA---------------------------

----------------------------------------------------------TG

TATGATTTTGTATGATTT-GTATGATAAATATCATATAGTGTTTTTATCCT---------

--------------TAGGAGAGATCTC-----------AAACTTCT-----AATCAATGG

AATCTAA-----------CTACATCGAATAGT---TTGAGGGAAAGGAAAGCATAAAACG

AATTGAAA--------AAAAAAAGAAGTCGTA----------------------------

-GCAAAAAGGAGTGGTAC---TGAGATCATTTGCCCTATATGTGAAAATAGAATTTGGAC

ATATCTTTACCCGGAGTAG----AACATAAACCT-------------------AAAAGAG

TTGAAA-GGGCCCACTCAGGAACAAGAAAATGACATCGTGATTTGAATCTCGATGAAACA

ATA-CATCAATGGGAGATTAGTGCAAATTAGTGCAATAAGTTTAGTCCATTTTTTT----

-------------------------------TATAGGAAAGGGCACGTAAACTCATATAT

ATATATATATTTTTGAAAAATAGAAGAAAGG----CCCTTC-----------AAAAAAAC

GTT-----AAAAAAAGAAATAAAACTATAATCGACACATTGA--------TTTTTTTTCG

CTAATTTTTT----TGAACCATATGCATCCAAAGGTGCACGTATGGTTCCTAAGGGATAG

GATTTTGTCCTAATCAACCGACTTTATCGAGAAAGATTACTTTTTTTAGGCCAAGAAGTT

GATAGCGAAATCTCGAATCAACTTGTTGGCCTCATGGTATATCTCAGTATAGAAGATAAT

ACTAGAGATTTTTATTTGTTTATA--AACTCTCCAGGCGGATGGGTAATACCAGGAATAT

CCATTTATGATACTATGCAATTTGTGCCACCCGATGTACATACAATATGCATGGGATTAG

CTGCTTCAATGGGATCTTTCATTCTAATTGGAGG-------GGAAATTACCA---AACGT

CTAGCATTCC-CTCACGCTTGGCGCCAATGAGTTGT---TTTGATT--------TGAAAA

AAAAAAAGAGACTATGCC-TTCGCCATATCGATTATTCATATTCGA--------------

------------------------------------------------------------

-------------ATATGAATAAAATAATAAGTAATAATAGCATGGCACTTAAAATTCGA

TATGAACAAACTATTATTGTGTTTTCATAATTTTCATAACATGAGATTTTGTATCGAGAT

AGTAGTATGAAAC-AAAGGTTATTTCCGATTGAATGAATTTTAT-------GTGT----C

GGGAGTAAATTGAGTAGCGTCACAAACCATTTTTATTCCACACCAGAA---GCTTTTTTT

CTAATATTTATG---AAAGAAAAAGAGTACGAAAATGAAAAAAAAATCA-----------

---TTTTTTCCTTATTTGCTTGA---TCGTATCAAAAAGACACTTTGGGATTGCTAAATC

ACATACG---------------------AAATAAAGCAACAGAACCATCATAGTATTT--

-------------------TTTTGACTCTTACG-----AAAGGAAGGGTGGTAAATGGAT

CTTTTAACGAT----CTGAATCGGATGGATTCTATCTCTTCC-----------TTCAATG

AAAGGAAAAGGAGGGAGGATGAGTAAAGTCCATTGCAGAGCCGTATGCAATATGCAAAAG

ATGCCCGTACGGTTGTT-AATTCTATCTTTATTAGAATTATTCTTGTTATTTTTTCATTT

C------------TTTAGAAAAATCATGCG------AGATAGAAAAACCGTTCAT----G

GTGTTATATCAATATCATCAGATCAGGGTTATGATTCACCAACCTGCGAGTTCTTTTTAT

GAGGCACAAGCAGGAGAATTTATCCTGGAAGCGGAAGAACTACTGAAACTTCGC----GA

AACCCTCACAAAGGTTTATGTA--CAAAGAACGGGCAACCCTTTGTGGGTTGTATCCGAA

GATATGGAAAGAGATGTTTTTATGTCAGCAACAGAAGCCCAAGCTCATGGCATTGTTGAT

CTTGTAGCGATCTAAA------------ATGAAAATACTG-------GGGATTTCGTGTA

A-----------------------------------------------------------

------------------------------------------------------------

------------------------------------------------------------

------------------------------------------------------------

------------------------------------------------------------

------------------------------------------------------------

-----------------------------------------ATGGAAGAAT---------

---TACAAGGATATTTAG-----AAAAAGATAG----ATCTAGGCAGCAATGC------T

TTTTATATCCGTTTCTCTTTCAGGAGTATATTTACACGCTTGCTCATGGTCATGGTTTAA

ACGG------TTCAATTTTTTACGAACCCGTAGAAATTTTTGGTT------ATGACAATA

AATCTAGTTCAGTACTTGTGAAACGTTTAATTACTCGAATGTATCAACAGAATTATTTGA

TTTATTCGGTTAATGGTTCTAACC------AAAATCGATTCATCGGGCGCAACCATTATT

TTTATTCTCA---GTTTTTTTCTCAAATGGTATCAGAAGGTTTTGCAGCCATTGCGGAAA

TTCCATTCTCGCCGCGATTAGTATTTTCTCCC---GAAGAAAAAG---AAATACCAAAAT

CTCA------GAATTTACGATCTATTCATTCAATATTTCCCTTTCTAGAGGACAAATTAT

CGCATTTTCATTATGCGTCAGATATATTAATACCCTACTCTATCCATATCGAAACCCTAG

TTCAAATCCT------TCAATGCTGGGTCCAGGATGTTCCCTCTTTGCATTTCTTGCGAT

TCTTTCTCCATGAATATGATAATTGGAATAGTTTT---ATTAC------TCCGAATA---

------AATCTAGTTACGCT------ATTTCAAAAGAAAATAAAAGATTATTTTGGT---

TCTTGTATAGTTCTTATGCATTTGAATTCGAATTTTTATTCATTTTTCTTCGTAAAC---

AATCCTATTATTTACAATCAATATCTTCTGGACCCTTTCTTGAGCGAACCCATTTTTTAT

GGAAAAATGGAAC---------------------ACCTTA---------CAGATGTATGT

TGT---AATTCTTCTCAGAATCA--GAAGGCCCTA------TGGTTCTTCATAGATCCCT

TCATGCATTATATTCGATATCAAAGAAAAGTGATTTTGGCTTCAAGGGGTACTCATCTTC

TGATCAAGACATGGAAATGTTACCTTGTCAATTTCTGGCAATATTATTTGCATTTTTGGT

CTCACCCGCACAGGATCCATATCAACCAATTATCAAACCATTCCTTCTATTTTTTGGGAT

ATCTTTCAAGTGTACTAA-----TAAATCCCTCGACGGTCAAAAACCAAACGTTAGAGAA

TTCATTTCTGATAGATACTGTTACTAAG------AAATTCGATACCATAGTCCCCGTAAT

TCCTCTTATTGGATCATTGTCTAAAGCTAAATTTTGTACCGTATCGGGGCATCCCAGTAG

TAAGGCAATATGGACTGATTTATCAGATTCTGATATTGTTGATCGATTTGGTCGAATATG

TAGAAATCTTTCTCATTATTACAGTGGATCCTCGAGAAAACAAAGTTTGTATCAAATAAA

GTATATACTTCGGTTTTCGTGTGCTAGAACTTTGGCCCGTAAACATAAAAGTACGATACG

CACTCTTGTGCAAAGATTAGGTTTCGAATTCTTAGAAAAATTCTTTACAGAAGAAGAACA

AGTT--------CTTTCTTTGATC-----TTCCA---AAAAGGAGCAATCCCTTTTCCTT

TATATAGATTACATA---GAGAAAGTATTTGGTATTTGGATATTAGCCGTATCAATGACT

TGGCGAATC----------------ATTTAGATGGGTCATGAGGCCTTCTAATTCTAAAT

CGAATAGAAATGATCCATAA----------------------------------------

------------------------------------------------------------

------------------------------------------------------------

------------------------------------------------------------

------------------------------------------------------------

------------------------------------------------------------

------------------------------------------------------------

------------------------------------------------------------

------------------------------------------------------------

------------------------------------------------------------

------------------------------------------------------------

------------------------------------------------------------

------------------------------------------------------------

------------------------------------------------------------

------------------------------------------------------------

------------------------------------------------------------

------------------------------------------------------------

------------------------------------------------------------

------------------------------------------------------------

------------------------------------------------------------

------------------------------------------------------------

------------------------------------------------------------

------------------------------------------------------------

------------------------------------------------------------

------------------------------------------------------------

------------------------------------------------------------

------------------------------------------------------------

------------------------------------------------------------

------------------------------------------------------------

------------------------------------------------------------

------------------------------------------------------------

------------------------------------------------------------

------------------------------------------------------------

------------------------------------------------------------

------------------------------------------------------------

------------------------------------------------------------

------------------------------------------------------------

------------------------------------------------------------

------------------------------------------------------------

------------------------------------------------------------

------------------------------------------------------------

------------------------------------------------------------

------------------------------------------------------------

------------------------------------------------------------

------------------------------------------------------------

------------------------------------------------------------

------------------------------------------------------------

------------------------------------------------------------

------------------------------------------------------------

------------------------------------------------------------

------------------------------------------------------------

------------------------------------------------------------

------------------------------------------------------------

------------------------------------------------------------

------------------------------------------------------------

------------------------------------------------------------

------------------------------------------------------------

------------------------------------------------------------

------------------------------------------------------------

------------------------------------------------------------

------------------------------------------------------------

------------------------------------------------------------

------------------------------------------------------------

------------------------------------------------------------

------------------------------------------------------------

------------------------------------------------------------

------------------------------------------------------------

------------------------------------------------------------

------------------------------------------------------------

------------------------------------------------------------

------------------------------------------------------------

------------------------------------------------------------

------------------------------------------------------------

------------------------------------------------------------

------------------------------------------------------------

------------------------------------------------------------

------------------------------------------------------------

------------------------------------------------------------

------------------------------------------------------------

------------------------------------------------------------

------------------------------------------------------------

------------------------------------------------------------

------------------------------------------------------------

------------------------------------------------------------

------------------------------------------------------------

------ATGTTTCTGCTTCACGAATAC--GATATTTTCTGGGCATTTCTAATA-----AT

ATCAAGTGTTATTCCTATCTTAGCATTTGTAATTTCCGGAGTTTTAGCACCAATTAGGGA

AGGACCGGAGAAGCTCTCTAGTTATGAA-TCGGGTATAGAA-CCTATGGGGGATGCTTGG

T-TACAATTCCGAATTCGCTATTACATGTTTGCTCTAGTTTTTGTTGTTTT--TGATGTT

GAAACGGTCTTTCTTTATCCATGGGCAATGAGTTTCGATGTATTGGGTGTATCCGTATTT

ATAGAAGCTTTAA--TTTTCGTGCTTATCTCAATTGTTGGTTCAGTTTATGCATGGCGAA

AAGGAGCATTAGAATGGTCTTAA---ACGAGTTATTTTCC------TTGGTTAACAATAC

TTGTTGTTTTGCCGATATTCGCGGGTTCTTCCATTTTTTTTCTCCCTCATCGGGGAAATA

AGGTCTTCCGCTGGTATACTATATGCATATGCATAT------TAGAACTCCTTATAACGA

CCTA-----------TGTATTCTGTTATAATTT-CCAATTTGACGATCCACTAATTCAAT

TGGAAGAGGATTTTAAATGGATAAATATTTTTGATTTTCACTGGAAATTAGGGGTCGATG

GACTTTCCATAGGACCCATTTTACTGACAGGATTTATCACTACTTTAGCTACTTTAGCGG

CTTGGCCAGTTACTCGAAATTCACGATTGTTCTATTTCCTAATGTTAGCAATGTACAGCG

GTCAAATAGGATCATTTTCTTCTCGAGACCTTTTACTTTTTTTCATTATGTGGGAGTTAG

AATTAATTCC-TGTTTACTTACTTTTATCCATGTGGGGGGGAAAGAAACGCCTGTACTCT

GCTACAAAGTTTATTTTGTACACTGCGGGGGGTTCCATTTTTCTTTTAATAGGAGTTCCA

GGTATGGGTTTA------TATGGTTCTAATGGGCCGACATTAGATTTTGAAAAATTAGCT

AATCAATCATATCCTGCGGTATTGGAAATAATATTATATATTGGCTTCCTTATTGTTTAT

GCTGTCAAATCACCGATTATCCCCTTACATACATGGTTACCAGATACCCATGGAGAAGCG

CATTACAGTACATGTATGCTTCTAGCCGGAATTTTATTAAAAATGGGAGCATACGGATTG

ATTCGGGTTAATATGGAATTATTCCCCCACGCTCATTCTTTATTTTCCCCGTGGTTAGTG

ATAGTCGGAACGATGCAAATAATCTATGCAGCTTTAATCTCTTTCGGACAGCGCAATTTA

AAAAAGAGAATAGCCTATTCCTCCGTATCTCACATGGGTTTCATAATTATAGGAATTGGT

TCTATAACCGACATGGGACTAAACGGAGCCATTTTACAAATACTTTCCCATGGATTTATT

GGTGCTGCACTTTTTTTCTTGGCTGGAACAAGTTGTGATAGAATACATCTTGTTTATCTC

GACGAAATGGGGGGGATATCT-------------ATTCCAATGCCAAAAATATTTACTAT

GTTTAGTGGCTTCTCGATGGCTTCTCTTGCATTGCCGGGAATGAGTGGTTTTGTTGCTGA

ATTAGTAGTATTTTTTGGAATAATTGCCAGCTCAAAATATCTTTTAATGTCAAAAGTGTT

AATTACTTTTGTAATGGCAATTGGAATGATATTAACTCCTATTTATTTATTATCTATG--

------TTACGCCAGATGTTCTACGGATACAGGCTATTCCATGTTCCAAACTTTCATTTT

ATGGACTCCGGGCCACGCGAACTATTTGTTTCAATCTGTATC-TTTCTACCCATAATC-G

GGATCGGTATTTAT---CCTGATT-TTGTTCTCTTGTTATCAGTTGACAAAGTACAAGTT

ATCTTATCTAATTTCTATTATAAATAGATGATGTTCGAGCATGTACTTTTTTTAAGTGTC

TATTTATTTTCTATCGGTATCTATGGATTAATCACAAGTCGAAACATGGTTCGAGCACTT

ATGTGCCTTGAGCTTATACTAAATTCGGTTAATATCAATCTCGTAACATTTTCTGATTTT

TTTGATAGTCGTCAATTAAA----------------------------------------

-----------------------------------GGGAGACATTTTCTCCATTTTTCTC

ATAGCTATTGCAGCCGCCGAAGCGGCCATTGGGCTAGCTATTGTTTCGTCCATCCATCAA

AACAGAAAATCAACTCGTATCAATCAATCGAATTTGTTGAATAATTAGATGGAACATATA

TATAAATATGCATGGATAATACCCCTTCTTCCACTTCCAGTTGCTATGTCAATAACATTT

GGACTTCTTCTTATTCCTACAGCAACAAAAAATATTCGGCGTATATGGGCTTTTACTAGT

GTTTTACTGTTAAGTATAACTATGAGTTTTTCGATCAATCTATCCATTCAGCAAGTAAAT

GGAAGTTTTATCCATCAAAATCTATGGTCTTGGACCATCAATAATGATTTTTCCTTAGAG

TTCGGATACTTGATCGATCCACTTACTTCTATTATGTCAATACTAATTACTACTGTTGGA

ATCATGGTTCTTATTTATAGTGACAATTATATGTCTCACGATCAAGGATATTTGAGATTT

TTTGCTTATATTAGTTTTTTCAATACTTCTATGTTGGGATTAGTTACTAGTTCCAATTTG

ATACAAATTTATATTTTTTGGGAATTAGTGGGAATGTGTTCCTATTTATTAATAGGTTTT

TGGTTTTCACGACCAATTGCGGCAAGTGCTTGCCAAAAAGCCTTTGTAACTAACCGTGTG

GGGGACTTTGGTTTATTATTAGGAATCTTAGGTTTTTATTGGATAACGGGCAGTTTAGAA

TTTCGGGATTTGTTCGAAATAGTTAATAACTTAATC---C------GTAATAATGGGGTC

AATTCCTTCTCTGTAACTCTGTGTGCTTCTTTATTATTTGTCGGTGCAATTGCTAAATCC

GCACAATTCCCCCTTCACGTATGGTTACCTGACGCCATGGAGGGGCCCACTCCCATTTCA

GCTCTTATCCACGCGGCTACCATGGTAGCCGCGGGAATTTTTCTTGTAGCTCGGCTTCTC

CCTCTTTTCATAGTCATACCTCACATAATGAATCTCATTTCTTTAATAGGCATAATAACA

GTACTATTAGGAGCTACCTTAGCCCTTGCTCAAAGAGACATAAAAAGAAGTTTAGCCTAT

TCTACAATGTCTCAATTGGGTTATATTATGTTAGCTCTAGGTATCGGTTCTTATCGAGCT

GCTTTATTCCATTTGATCACTCATGCCTATTCTAAAGCTTTATTGTTTTTGGGATCCGGG

TCGATTATTCATGCAATGGAACCCATTGTTGGATATTCACCGGATAAAAGTCAGAATATG

ATTCTTATGGGTGGGTTAAAAAGATATGTTCCAATTACAAAAATTGCCTTTTTATTAGGT

ACACTTTCTCTTTGTGGTATTCCGCCTCTTGCTTGTTTTTGGTCCAAGGATGAAATTCTT

AATGATAGCTGGTTGTATTCACCAATTTTTGCAATTATAGCTTATTGCACAGCAGGATTA

ACCGCATTTTATATGTTTCGGGTGTATTTACTTACTTTTGATGGGCATTTGCGTGTTCAT

TTTCAACATTATAGTAGCACTATAAATAGCTCATTTTATTCAA------TATCTCTATGG

GGAAAAGAAACATCCGGAGAAGTCAATAGAAAT----TTACTTTTAT----CAAA-----

--------AAT--GAATAAAAAGG---------TCTCCTTT------------TTTTCA-

-----AAAGATATATATCC----A--ATTCATGATTATTTCAAAAATAGA----------

ATGCGATACT---TGAGTACTTACTTTGGAAATAAATATAAATATACTTACACGTATCCT

CATGAATCGGACAATACTATGCTATTTCCTATTCTGGCATTGGTATTATTTACTTTGTTC

ATTGGATTAATAGGAATCCATTTCAATCAA---------------GGGATAGTGGATTTG

GATATATTATCCAAATGGTTAACTCCATCGGCA------AACTTTTTTCATCGAAATTCG

AATTCTTCTGTGGATTGGTATGAGTTTTTTACAAATGCAATTTTTTCAGTAAGTATAGCC

CTTTTTGGACTATTTATA-GCATCTATTTTATATGGGTCTATTTATTCGCCTTTTCAGAA

TTTGGACTTAATTAATTTTTTTGTAAAGAGGAGCCCTAA---AAGATT------TTTATT

GGACCAAATGAAAAATGTGGTATACAATTGGTCATATAATCGCGGTTACATAGATATTTG

TTATACTAGGGTCTTAACAATGGGGATAAGAGGATTAGCCGATCTGACTCGGTTTTTTGA

TAGACGTATAATTGATGGAATTACAAACGGAGTTGGGGTTTCAAGTTTCTTTATAGGGGA

GGGGGTCAAATAT---ATGGGGGGTGGACGAATTTCGTCTTATCTATTCTTATATTTATC

TTCTGTATCAATATTTATCTTCTGTATCAATATTTTTATTATTTTATTATTAGACTTAAA

TTTTCTAAATAATGATTCCCTATCAAAATTAAACGGATTTTTTTGAATGGATTTACCTGG

GCCAATACATGATATTCTTGTAGTATTTCTGGGATCGGTTCTTATATTAGGGGGCCTAGG

GGTAGTATTATTTACCAATCCAATTTATTCTGCCTTTTCATTGGGATTGG---TTCTTGT

TTGTATATCCTT-ATTCTATATTCTATCGAATTCGTATTTTGTAGCTGCTGCACAACTCC

TTATTTATGTAGGAGCCATAAATGTCTTAATCCTGTTTGCTGTAATGTTCATGAATAGTT

CAGAATATTCCAACGATTCCCATCCCTGGACGGTTGGGGATGGAGTCACTTCATTGGTTT

GTACAAG---------TATTCTTTTTTCATTAATTACT-ACTATCCCGGATACCTCATGG

TACGGAATTCTTTGGGCTACAAGATCAAACCAGATTCTAGAACAGGACCTAATAAGTAAC

GTTCAACAGATTGGGATTCATTTAGCAACAGATTTTTATCTTCCCTTTGAACTCATTTCT

ATAATTCTTTTAGTTTCCTTAATAGGTGCAATTACTATGGCTCGCCAA------------

TAA---------------------ATGACTATGTCGGCTACAAG------AAAAGACCTC

ATGATAGTCAATATGGGCCCGCACCACCCATCGATGCATGGTGTTCTTCGACTGATCGTT

ACTCTCGACGGTGAAGATGTTATTGACTGTGAACCCGTATTGGGCTATTTACACAGAGGG

ATGGAAAAAATAGCAGAAAACCGAACAATTATACAATATCTGCCTTATGTAACACGTTGG

GATTATTTAGCTACTATGTTTACAGAGGCAATAACAGTAAATGCACCAGAACAATTGGGA

AATATTCAAGTACCCAAAAGGGCCAGCTATATCAGAGTAATTATGCTGGAGCTTAGTCGT

ATAGCTTCTCATTTGTTATGGCTTGGACCTTTCATGGCGGATATCGGTGCACAAACTCCC

TTTTTCTATATTTTAAGAGAGAGAGAATTGTTATATGATCTATTCGAAGCTGCCACAGGT

ATGCGAATGATGCATAATTATTTCCGTATCGGAGGAGTAGCTGCTGATTTACCTCATGGC

TGGCTAGATAAATGCTTAGATTTCTGCGATTATTTTTTAGCGGGGGTTGGTGAATATGAA

AAACTTATTACGAGGAATCCCATTTTTTTGGAACGGGTTGAGGGAGTGGGTGTTATTGGT

GGAGAGGAAGCCATAAATTGGGGTTTATCCGGACCAATGTTACGAGCTTCCGGGATCCAA

TGGGATCTTCGTAAAATCGATCATTA--TGAATGTTACAATGACTTCGACTGGGAAGTCC

AATGGCAAAAAGAAGGAGATTCATTAGCTCGTTATTTAGTACGGATCGGCGAAATGAGAG

AATCCATAAAAATCATTCAACAGGCTTTAGAAGGAATTCCCGGGGGGCCCTATGAGAATT

TAGAAGTTCGGCGC------TTTGCTAGAGCAAAGAATGCCGAAT----GGAATGATTTT

GAATATAGATTCATTAGCAA--AAAAC-------CTTCGCCTAATTTGGAATTGTTGAAA

CAAGA---ACTTTATGTAAGGGTAGAAGCCCCAAAAGGAGAATTAGGAATTTATTCGATA

GGCGATAATAGTGTTTTCCCCTGGAGATGGAAAATTCGTCCACCCGGTTTCAT-------

---CAATTTGCAAATTCTTCCCCAGCTAATTAAAAGAATGAAATTGGCTGATATTATGAC

GATACTAGGTAGTATAGATATCATTATGGGAGAAGTTGATCGTTGAATGTTTCCTATGGT

GACCGGGTTCATGAATTACGGTCAACAAACAATACGAGCCGCAAGGTACATTGGTCAAAG

TTTCATAATTACCTTATCACACACAAATCGTTTACCTGTAACTATTCAATATCCTTATGA

AAAATTGATCACATTAGAGCGTTTCCGGGGTCGAATTCACTTTGAATTTGATAAATGTAT

TGCCTGTGAAGTATGTGTTCGTGTATGCCCCATAGATCTACCCGTTGTAGATTGGAGATT

TGAAAGAGATATTAAAAAG---AAACAATTGCTTAATTATAGTATTGATTTTGGTGTCTG

TATATTTTGTGGTAACTGTGTCGAATATTGTCCAACAAATTGTTTATCAATGACTGAAGA

ATATGAGCTTTCTACTTATGACCGTCACGAATTGAATTATAATCAAATTGCTTTGGGTCG

GTTACCAATGTCAGTAATTGGAGATTACACAATTCAAACTGTTATGAATT------CAAC

TAAAATC---AACATAGACGGAGAGAAATCCATT---GATTCAAGAACGATTACCAATTA

TTAA------------AT------------------------------------------

---GCAGGGTCGTTTATCTGATTGGCTAGTCAAGCATGAACTAGTTCATAGATCTTTGGG

CTTCGATTATCAAGGAACAGAGACTTTACAAATAAAAACAGAGGATTGGGACTCCATTGC

TGTCATTTTATATGTATATGGTTACAATTATTTACGCTCCCAATGCGCCTATGATGTAGC

ACCGGGCGGATTTTTAGCTAGTGTGTATCATCTTACGAGAATACAGTATGGTATAGATAA

ACCGGAAGAGGTATGCATAAAAGTATTTGTCTCAAGGAGTAATCCTAGAATCCCCTCTGT

TTTCTGGATTTGGAAAGGTGCTGATTTTCAAGAACGGGAATCTTATGATATGTTGGGAAT

CTCTTATTATAATCATCCACGGCTTAAACGTATCTTGATGCCGGAAAGTTGGATAGGCTG

GCCCTTACGTAAGGACTATATCACCCCAAATTTCTATGAAATACAAGATGCTCATTGA--

------------------------------------------------------------

-------------------------------------------------ATGGTCTTAAC

G---GAATATTCAGACAATCAGAATAAAAAGAAAGGAAAAGAGTACATTGAAACAGTCAT

GAATTTGATTGAGTTCCCGTTACTTGACCAAACAACACCCCATTCCGTTATTTCAACTAC

ATCAAATGATCTTTCCAATTGGTCAAGACTCTCCAGTTTATGGCCACTTCTCTACGGTAC

TAGTTGCTGTTTCATTGAATTTGCTTCATTAATAGGCTCGCGATTCGACTTTGATCGTTA

TGGATTGGTGCCAAGATCGAGCCCTA-GGCAAGCAGACCTCATTTTAACAGCCGGCACAG

TAACAATGAAGATGGCTCCTTCTTTAGTGAGATTATATGAGCAAATGCCCGAACCAAAAT

ATGTCATTGCTATGGGAGCCTGTACTATTACAGGAGGGATGTTCAGTACTGATTCTTATA

GTACTGTTCGGGGAGTCGATAAGCTAATTCCTGTGGATGTCTATTTGCCGGGCTGCCCGC

CTAAACCGGAGGCAGTTATAGATGCTATAACGAAACTTCGTAAGAAGATATCTCGAGAGA

TATCTCAAGATAGGACTGTGT------CTCAACAGGAAAATCGGTGTT----------TT

ACTATCAATCACAAGTTTTATGTTCGACGCAGTACTCAGACTGGAAATTACGATCAAGGA

CTGCTCTATCAATCATC---------------ATCCACTTCAGAGATACTTTCTGAAA--

----------------------GA------TTTTTTCAACCCAAAAATTTAGTATCTTCC

CACGAATTAGTGAAT--------CAA-GAAAGTTCTTTTGTGCAGAATAA----------

---------------------------------ATGC---ACAATAGAAATACTTTATCT

TGGGTAAAGGAACAGATGACCCGAGCCATTTATGTATCGATCATGATATATGTAATAACC

TGGGCATCTATTTCAAATGCATATCCCATTTTTGCGCAGCAGGGTTATGAAAATCCACGA

GAAGCGACTGGACGAATTGTATGTGCCAATTGCCATTTAGCTAATAAGCCCGTGGATATT

GAAGTTCCGCAAACTGTGCTTCCTGATACTGTATTTGAAGCAGTTGTTCGAATCCCTTAT

GATATGCAACTGAAACAAGTTCTTGCTAATGGTAAAAGGGGGGCTTTGAATGTGGGGGCT

GTTCTTATATTACCCGAGGGATTTGAATTAGCTCCCCCGGATCGTATTTCTCCCGAGGTG

AAAGAAAAGATG---GGAAATCTATCCTTTCAGAATTATCGTCCCAATAAAAAAAATATT

CTTGTAATAGGCCCCGTTGCCGGTAAGAAATATAGTGAAATTGTCTTTCCCATTCTTTCT

CCCGACCCTGCTACGAAGAAAGACGTTCACTTCTTAAAATATCCCATATATGTAGGCGGG

AATAGAGGGAGGGGTCAGATTTATCCTGACGGGAGCAAGAGTAACAATACAGTCTATAAT

GCTACATCAACAGGAATAGTAAGCAGAATAGTACGTAAAGAAAAG---GGGGGATATGAA

ATAACCATAGTTGATGCATCGGATGGGCATCAAACGGTTGATATTATACCTCCGGGACCA

GAACTTCTTGTTTCGGAGGGCGAATCCATCAAGCTTGATCAACCATTAACAAGCAATCCT

AATGTGGGAGGGTTTGGTCAGGGAG-ATGCA--GAAATAGTGCTTCAAG-ACCCATTACG

TG-TCCAAGGTCTTTTGTTCTTCTTGGCATCTGTTATTTTGGCACAAATCTTTTTGGTTC

TTAAAAA-GAAACAGTTTGAAAAGGTTCAATTGTACG-AAATGAATTTCTAG--------

------------------------------------------------------------

------------------------------------------------------------

------------------------------------------------------------

------------------------------------------------------------

------------------------------------------------------------

------------------------------------------------------------

------------------------------------------------------------

------------------------------------------------------------

------------------------------------------------------------

------------------------------------------------------------

------------------------------------------------------------

------------------------------------------------------------

------------------------------------------------------------

------------------------------------------------------------

------------------------------------------------------------

------------------------------------------------------------

------------------------------------------------------------

------------------------------------------------------------

------------------------------------------------------------

------------------------------------------------------------

------------------------------------------------------------

------------------------------------------------------------

------------------------------------------------------------

------------------------------------------------------------

------------------------------------------------------------

------------------------------------------------------------

------------------------------------------------------------

------------------------------------------------------------

------------------------------------------------------------

------------------------------------------------------------

------------------------------------------------------------

------------------------------------------------------------

------------------------------------------------------------

------------------------------------------------------------

------------------------------------------------------------

------------------------------------------------------------

------------------------------------------------------------

------------------------------------------------------------

------------------------------------------------------------

------------------------------------------------------------

------------------------------------------------------------

------------------------------------------------------------

------------------------------------------------------------

------------------------------------------------------------

------------------------------------------------------------

------------------------------------------------------------

------------------------------------------------------------

------------------------------------------------------------

------------------------------------------------------------

------------------------------------------------------------

------------------------------------------------------------

------------------------------------------------------------

------------------------------------------------------------

-----ATGATTGAAGTTTTTCTATTTGGAATCGTTTTAGGTCTAATTCCTATTACTTTGG

CAGGATTATTCGTAACTGCATATTTACAATATAGACGCGGTGATCAATTGGATCTTTGA-

--ATGCCCACTATAACTAGTTATTTCGGTTTTCTACTGGCTGCTTCAACTATAACCCCAG

CTCTATTGATTGGTTTGAACAAGATACGACTTATTTGAATGGATATAGTAAGTCTTGCTT

GGGCTGCTTTAATGGTAGTCTTTACATTTTCCCTTTCACTCGTAGTATGGGGAAGGAGTG

GGCTCTAGATGATTATTCGTTCGCCGGAACCAGAAGTGAAAATTGTTGTAGATAGGGATC

CCGTCAAAACGTCTTTCGAGGAATGGGCTAGACCCGGCCATTTTTCACGAACAATAGCTA

A---GGGCCCTGATACTACCACTTGGATCTGGAACCTACATGCTGATGCACACGATTTTG

ATAGTCATACCAGTGATTTGGAGGAGATCTCTCGAAAAGTATTTAGTGCTCATTTCGGTC

AACTCTCCATTATCTTTATTTGGCTGAGTGGCATGTATTTCCATGGTGCCCGTTTTTCCA

ATTATGAAGCATGGCTAAGTGATCCTACTCACATTAGGCCCAGTGCGCAGGTAGTTTGGC

CAATAGTGGGTCAAGAAATATTGAATAGTGATGTGGGCGGGGGTTTCCGAGGAATACAAA

TAACCTCCGGTTTTTTTCAGATTTGGCGAGCATCCGGAATAACTAGTGAATTACAACTCT

ATTGTACTGCAATTGGTGCATTGGTCTTTGCATCGCTAATGCTTTTTGCCGGTTGGTTTC

ATTATCATAAAGCCGCCCCAAAATTGGCTTGGTTCCAAGATGTAGAATCCATGTTAAATC

ACCATTTAGCGGGGTTACTAGGACTTGGGTCTCTTTCTTGGGCGGGACACCAAATCCATG

TATCTTTGCCAATTAACCAATTTCTTGATGCTGGAGTTGATCCTAAAGAGATACCACTTC

CTCATGAATTTATCTTGAATCGGGATCTTTTGGCTCAACTTTATCCCAGTTTTTCCGAAG

GAGCAACTCCCTTTTTTACCTTGAATTGGTCAAAATATGCGGAATTTCTGAGTTTTCGCG

GAGGATTAGATCCAATAACAGGGGGTCTATGGCTGAGCGATATTGCACACCATCATTTAG

CTATTGCAATTCTTTTCCTGATCGCCGGTCATATGTATAGGACCAACTGGGGCATTGGGC

ATGGCCTTAAAGACATTTTAGAGGCTCATAAAGGCCCATTTACGGGCCAGGGCCATAAGG

GACTTTATGAAATCC-TAACAACG--TCATGGCATGCTCAATTATCTCTTAACCTGGCTA

TGTTAGGCTCTTTAACCATTGTTGTAGCCCACCATATGTATTCCATGCCCCCTTATCCAT

ACTTAGCTATTGACTATGGTACACAACTTTCGTTGTTCACACATCACATGTGGATCGGTG

GATTTCTTATAGTTGGTGCTGCTGCGCACGCAGCCATTTTTATGGTAAGAGACTACGATC

CAACTACTCGATACAACGATCTGTTAGATCGTGTCCTTAGGCACCGCGATGCAATCATAT

CACATCTTAACTGGGCATGTATATTTCTAGGTTTTCACAGTTTTGGCTTGTATATTCATA

ATGATACCATGAGCGCTTTAGGTCGTCCCCAAGATATGTTTTCAGATACCGCTATACAAT

TGCAACCCATCTTTGCTCAATGGATACAAAACACCCACGCTTTAGCGCCCGGCTTAACAG

CCCCTGGTGCAACAACAAGTACCAGCTTAACTTGGGGGGGTAGTGAGTTAGTAGCAGTAG

GCGGCAAAGTCGCTTTGTTGCCTATTCCATTGGGAACCGCGGATTTTTTGGTCCATCACA

TTCATGCATTTACGATCCATGTAACTGTATTGATACTACTGAAAGGGGTTCTATTTGCTC

GCAGTTCCCGTTTGATACCCGATAAAGCAAATCTGGGCTTTCGTTTCCCCTGTGATGGGC

CTGGAAGAGGGGGGACATGTCAAGTATCCGCCTGGGATCATGTCTTCTTAGGTTTATTCT

GGATGTACAATGCAATTTCGGTAGTCATTTTCCATTTCAGTTGGAAAATGCAATCAGATG

TTTGGGGTACTATAAGTGATCAAGGAGTAGTAACTCATATAACAGGAGAAAACTTTGCAC

AGAGTTCCATTACTATTAATGGGTGGCTCCGAGATTTCTTATGGGCACAGGCATCTCAGG

TAATTCAGTCTTATGGTTCTTCATTATCTGCCTATGGTCTTTTTTTCTTAGGTGCTCATT

TTGTCTGGGCTTTCAGTTTAATGTTTCTATTCAGCGGCCGTGGTTATTGGCAAGAACTCA

TTGAATCCATCGTTTGGGCTCATAACAAATTAAAAGTTGCTCCTGCTACTCAGCCTAGAG

CCTTGAGCATTGTACAAGGACGTGCTGTAGGAGTAACCCATTACCTTCTGGGTGGAATTG

CCACAACATGGGCATTCTTCTTAGCAAGAATTATTGCAGTAGGATAAATGGCATTAAGAT

TTCCAAGGTTTAGCCAAGGCTTAGCTCAGGACCCCACTACTCGTCGTATTTGGTTTGGTA

TTGCTACCGCACATGACTTCGAGAGTCATGATGATATTACTGAGGAACGTCTTTATCAGA

ACATTTTTGCTTCTCACTTTGGGCAGTTAGCAATAATCTTTCTGTGGACGTCCGGGAATC

TGTTTCATGTAGCTTGGCAAGGAAATTTTGAGTCATGGATACAGGACCCCTTACATGTAA

GGCCTATTGCTCATGCAATTTGGGATCCTCATTTTGGTCAACCAGCCGTAGAAGCCTTTA

CTCGAGGAGGCGCTATCGGTCCAGTGAATATCGCCTATTCTGGTGTTTATCAGTGGTGGT

ATACAATTGGATTACGTACCAATGAAGATCTTTATACGGGAGCTCTTTTTCTATTATT--

----TCTTTCTGCTATATCCTTAATTGCAGGTTGGTTACACCTACAGCCAAAGTGGAAAC

CTAGCGTTTCGTGGTTCAAAAATGCCGAATCTCGTCTCAATCATCATTTGTCAGGACTTT

TCGGAGTAAGTTCCCTAGCCTGGACAGGACATTTAGTTCATGTCGCTATTCCCGGATCC-

--AGGGGAGAGTACGTCAGATGGAATAATTTCTTAGAGGTATTACCGCACCCCCAAGGGC

TTGGACCACTTCTTACGGGTCAATGGAATCTTTATGCGCAAAACCCCGATTCCAGTGGTC

ATTTATTTGGTACCACCCAAGGGGCGGGAACTGCCATTCTAACCCTTCTTGGGGGATTCC

ATCCACAAACGCAAAGTTTATGGCTGACCGATATGGCTCATCATCATTTAGCTATTGCAA

TTCTTTTCCTGATCGCCGGT-CATATGTATAGAACAAACTTCGGGATTGGACACAGCATC

AAAGATCTTTTAGAAGCGCATATTCCTCCGGGGGGTCAATTAGGGCGTGGGCATAAGGGT

CTTTATGACACAATCAATAATTCGATTCATTTTCAATTAGGTCTTGCCCTAGCCTCTTTA

GGGGTTATTACTTCTTTAGTAGCTCAACACATGTACTCTTTACCTGCTTATGCATTCATA

GCACAAGACTTTACTACTCAAGCTGCATTATATACTCATCACCAATATATTGCGGGGTTC

ATCATGACAGGAGCCTTTGCGCATGGCGCTATATTTTTCATTAGAGATTACAATCCGGAA

CAGAACGAGGATAATGTATTGGCAAGAATGTTAGACCATAAAGAAGCTATCATATCTCAT

TTAAGTTGGGCTAGTCTGTTTCTAGGGTTCCATACCTTGGGCCTTTATGTTCATAACGAC

GTCATGCTCGCTTTTGGTACTCCGGAAAAACAAATCTTGATCGAACCTATATTTGCCCAA

TGGATCCAATCCGCTCACGGTAAGACTTCATACGGGTTCGATGTACTCTTATCTTCAACG

AATGGACCCGCATTCAATGCAGGCCGAAGCATATGGTTGCCTGGCTGGTTGAATGCTATT

AATGAGAATAGTAATTCACTCTTCTTAACAATAGGTCCAGGAGATTTCTTGGTTCA-TCA

TGCTATTGCTCTAGGTTTGCATACAACCACATTGATTTTAGTAAAAGGCGCTTTGGATGC

ACGTGGTTCTAAGTTAATGCCAGATAAAAAGGATTTCGGTTATAGTTTTCCTTGCGACGG

CCCGGGGCGCGGCGGTACTTGTGATATTTC-CGCTT--GGGACGCATTTTATTTGGCAGT

TTT-CTGGATGTTAAATACCATTGGATG-GGTTACTTTTT-ATTGGCATTGGAAACATAT

CACATTATGGCAGGGTAACGTTTCACAATTTAACGAATCCTCCACTTATTTAATGGGGTG

GTTAAGAGATTATTTATGGTTAAATTCTTCACAACTTATCAATGGGTATAACCCTTTTGG

TATGAATAGTTTATCAGTATGGGCATGGATGTTCTTATTTGGACATCTTGTTTGGGCTAC

TGGATTTATGTTCTTAATTTCTTGGCGCGGATATTGGCAGGAATTGATTGAAACTTTAGC

GTGGGCTCATGAACGCACACCTTTGGCTAATTTGATTCGATGGAGAGATAAGCCAGTGGC

CCTTTCCATTGTGCAAGCAAGATTGGTTGGATTAGCTCACTTTTCTGTAGGTTATATATT

CACTTACGCAGCTTTCTTGATTGCCTCTACATCAGGAAAATTTGGTTAAATGTCACATTC

AGTCAAAATTTATGATACATGTATAGGATGTACTCAATGTGTACGAGCCTGCCCCACGGA

TGTATTGGAAATGATACCTTGGGACGGGTGTAAAGCTAAGCAAATTGCTTCTGCACCAAG

AACTGAGGATTGTGTAGGTTGTAAAAGATGTGAATCCGCTTGCCCAACGGATTTTTTAAG

TGTACGCGTTTATTTATGGCATGAAACAACTCGAAGCATGGGTCTATCTTATTGAATGAC

AGATCTTAA---------------------------------------------------

---------CTTACCCTCTATTTTTGTGCCTGCTGTAGGCCTAGTATTTCCAGCAATTGC

AATAGCTTCTTTATTTCTTTATGTCCAAAAAAATAAGA----------------------

-------------------------------------------TTGTCTAGATGCGAGAT

ATAAAAACATATCTCTCCACGGCACCCGTACTAACTACTCTATGGTTTGGTTCTTTAGCG

GGTCTATTGATAGAGATTAATCGTTTATTCTCAGATG------CTTTGTCATTCCCCTTT

TTC------------------TAGATGACTGCAATTTTAGAGAGACGCGAAAGCACAAGT

TTATGGGGTCGCTTCTGCAACTGGATAACCAGCACCGAAAACCGTCTTTACATTGGATGG

TTCGGCGTTTTGATGATCCCTACCTTATTGACCGCAACTTCTGTATTTATTATCGCCTTC

ATTGCTGCTCCTCCAGTAGATATTGATGGTATTCGTGAGCCTGTTTCCGGATCTCTACTT

TATGGAAACAATATTATCTCTGGTGCCATTATTCCTACTTCTGCAGCTATCGGTTTGCAT

TTTTACCCGATATGGGAAGCAGCATCTGTTGATGAGTGGTTATACAATGGTGGTCCTTAT

GAGCTAATTGTTCTACACTTCTTACTTGGCGTAGCTTGTTACATGGGTCGTGAGTGGGAA

CTTAGTTTCCGTTTAGGTATGCGTCCTTGGATTGCTGTTGCATATTCAGCTCCCGTTGCA

GCTGCTACTGCTGTTTTCTTGATCTATCCTATTGGTCAAGGAAGTTTTTCTGATGGTATG

CCTTTAGGAATTTCTGGTACTTTCAATTTCATGATCGTATTCCAGGCAGAGCACAACATC

CTTATGCATCCATTTCACATGTTAGGCGTAGCTGGTGTATTCGGCGGCTCCCTATTCAGT

GCTATGCATGGTTCCTTGGTAACCTCTAGTTTGATCAGGGAAACCACTGAAAACGAGTCT

GCTAATGAAGGTTACAGATTTGGTCAAGAGGAAGAAACTTATAATATCGTAGCTGCTCAT

GGTTATTTTGGCCGATTGATCTTCCAATATGCTAGTTTCAACAACTCTCGTTCCTTACAT

TTCTTCTTGGCTGCTTGGCCTGTAGTGGGTATCTGGTTTACCGCTTTAGGTATTAGTACT

ATGGCTTTCAACTTAAATGGTTTTAATTTCAACCAATCTGTAGTTGATAGTCAAGGTCGT

GTCATTAATACTTGGGCTGATATCATCAACCGTGCTAACCTTGGTATGGAAGTAATGCAT

GAACGTAATGCTCATAACTTCCCGCTAGACCTAGCTGCTGTTGAAGCTCCGTCTACAAAT

GGATAAATGGGTTTACCTTGGTATCGTGTTCATACTGTCGTCTTGAATGATCCCGGTCGA

TTGCTTTCTGTCCACATAATGCATACAGCTCTAGTTTCTGGTTGGGCCGGTTCGATGGCT

CTATACGAATTAGCGGTTTTTGATCCCTCTGACCCTGTTCTTGATCCAATGTGGAGACAG

GGTATGTTCGTTATACCCTTCATGACTCGTTTAGGAATAACCAATTCTTGGGGGGGTTGG

AGTATTTCAGGAGGAACTATAACGAATCCGGGTATTTGGAGTTATGAAGGTGTGGCGGGG

GCACATATTATGTTCTCCGGCTTGTGCTTCTTGGCAGCTATTTGGCATTGGGTGTATTGG

GACCTAGAAATATTCTCCGATGAACGTACAGGAAAACCCTCTTTGGATTTGCCCAAGATC

TTTGGAATTCATTTATTTCTTTCAGGATTGGCTTGCTTTGGCTTTGGCGCATTTCATGTA

ACAGGCTTGTATGGTCCTGGAATATGGGTGTCCGATCCTTATGGGCTAACTGGAAGAGTA

CAATCTGTAAATCCAGCGTGGGGCGCTGAAGGCTTTGATCCTTTT-GTTCCGGGA--GGG

ATAGCCTCTCATCATATTGCAGCGGGTACATTGGGCATATTGGCAGGCCTATTCCATCTT

AGTGTTCGTCCGCCTCAACGTCTATACAAAGGATTACGTATGGGCAATATTGAAACTGTA

CTTTCCAGTAGTATCGCTGCTGTTTTTTTTGCGGCTTTCGTTGTTGCCGGAACTATGTGG

TATGGTTCGGCAACTACCCCAATCGAATTATTTGGTCCTACTCGTTATCAGTGGGATCAG

GGATACTTCCAGCAAGAAATCTATCGAAGAGTCGGTGCTGGACTAGCCGAAAATCTGAGT

TTATCCGGGGCTTGGTCTAAAATTCCCGAAAAATTAGCTTTTTATGATTACATTGGCAAT

AATCCGGCAAAAGGGGGATTATTCAGAGCGGGCTCAATGGATAACGGAGATGGAATAGCT

GTTGGATGGTTAGGACACCCCCTCTTTAGAGATAAAGAGGGTCGCGAGCTTTTTGTACGT

CGTATGCCCACTTTTTTTGAAACATTTCCGGTAGTTTTGGTAGATGGAGATGGGATTGTG

AGAGCTGATGTTCCTTTTAGAAGGGCAGAATCAAAGTATAGTGTTGAACAAGTAGGTGTA

ACTGTTGAATTCTATGGTGGTGAACTTAACGGAGTCAGTTATAGTGATCCTGCTACTGTG

AAAAAATACGCTAGACGTGCCCAATTAGGGGAAATTTTTGAATTAGACCGGGCTACTTTG

AAATCCGATGGTGTTTTTCGTAGCAGTCCAAGGGGCTGGTTCACTTTTGGGCATGCTACA

TTTG-CTTTGCTCTTC--TTTTTCGGACATATTTGGCACGGCGCTAGAACCTTGTTCAGG

GATGTTTTTGCTGGTATTGATCCAGATTTGGATGCTCAAGTGGAATTTGGAGCATTCCAA

AAACTCGGGGATCCAACTACAAGGAGACAAGCAGTCTGAATGAAAACCTTATATTCCCTG

AGGAGGTTCTACCCCGTGGAAACGCTCTTTAATGGAACTTTAGCTTTAGCTGGTCGTGAC

CAAGAAACCACCGGGTTCGCTTGGTGGGCCGGGAATGCCAGACTTATCAATTTGTCCGGT

AAACTACTTGGGGCTCACGTAGCCCATGCCGGGTTAATCGTATTCTGGGCCGGAGCAATG

AACCTATTTGAAGTGGCTCATTTTGTACCAGAGAAACCCATGTATGAACAAGGATTGATT

TTACTTCCCCACTTAGCTACTCTAGGATGGGGGGTAGGTCCGGGGGGGGAGGGTAATAGA

CACCTTCCCATACTTTGTATCTGGAGTACTTCACTTAATTTCTTCCGCAGTCTTAGGCTT

TGGCGGTATTTATCATGCGCTTCTTGGGCCTGAGACTCTTGAAGAATCTTTTCCATTTTT

CGGTTATGTATGGAAAGATAGAAATAAGATGACTACAATTTTAGGTATTCACTTAATTTT

GTTAGGTATAGGTGCTTTTCTTCTAG---TACTTAAAGCTCTTTATTTTGGAGGCGTATA

TGATACCTGGGCTCCGGGAGGGG---GCGATGTAAGAAAAATTACCAACTTGACTCTTAG

CCCAAGCGTTATATTTGGTTATTTACTAAAATCTCCTTTTGGGGGAGAAGGATGGATTGT

TAGTGTGGACGATTTAGAAGATATAATCGGAGGACATGTATGGTTAGGGTTCATTTGTAT

ATTTGGCGGAATTTGGCATATCTTAACCAAACCCTTTGCATGGGCTCGCCGCGCATTGGT

ATGGTCCGGAGAGGCTTACCTGTCTTATAGTTTAGGCGCTTTAGCTGTATTTGGTTTCAT

TGCGTGTTGTTTCGTCTGGTTTAATAATACTGCTTATCCTAGTGAGTTTTATGGGCCCAC

TGGGCCAGAAGCTTCTCAAGCTCAAGCATTTACTTTTCTAGTTAGAGACCAACGCCTTGG

GGCTAACGTGGGATCTGCCCAAGGACCTACTGGTTTAGGTAAATATCTAATGCGTTCCCC

CACTGGAGAGGTTATTTTTGGAGGGGAGACTATGCGTTTTTGGGATCTCCGCGCTCCTTG

GTTGGAACCTCTAAGGGGTCCTAATGGGTTGGACTTGAATAGACTGAAAAAAGACATACA

ACCTTGGCAAGAACGACGTTCGGCAGAATATATGACTCATGCCCCCCTAGGTTCTTTAAA

TTCTGTGGGTGGTGTAGCTACCGAGATTAATGCAGTCAATTACGTCTCTCCTCGAAGTTG

GTTAGCTACCTCTCATTTTGTTCTAGGATTCTTCTTGTTTGTGGGGCATTTGTGGCATGC

GGGAAGGGCTCGTGCGGCTGCAGCAGGATTTGAAAAAGGAATCGATCGTGATTTGGAACC

TGTTCTTTCCATGACCCCTCTTAG----ATGACTATAGCCCTTGGTAGATTTACCAAAGA

AGAAAAGGATTTATTTGATAGTATGGATGATTGGTTACGGCGGGACCGTTTTGTTTTTGT

AGGTTGGTCTGGCCTGTTGCTCTTTCCTTGTGCTTATTTCGCTTTAGGGGGTTGGTTCAC

AGGTACAACTTTTGTAACTTCATGGTATACCCACGGATTGGCTAGTTCCTATTTGGAAGG

TTGCAATTTCTTAACCGCTGCAGTTTCTACCCCTGCGAATAGTTTAGCACATTCTTTGTT

GCTACTATGGGGCCCTGAAGCACAAGGGGATTTCACTCGTTGGTGTCAATTAGGTGGTTT

GTGGACTTTTGTTGCGCTCCATGGTGCTTTTGGACTAATAGGTTTCATGTTACGTCAATT

CGAACTTGCTCGATCTGTTCAATTGCGGCCTTATAACGCAATCGCATTCTCTGGTCCAAT

TGCTGTTTTTGTTTCTGTATTCCTTATTTATCCGCTGGGTCAGTCTGGTTGGTTCTTTGC

GCCAAGTTTTGGTGTAGCAGCTATATTTCGATTCATCCTATTTTTCCAAGGGTTTCATAA

TTGGACATTGAACCCATTTCATATGATGGGAGTTGCCGGAGTATTAGGCGCCGCTCTGCT

ATGCGCTATTCATGGCGCTACCGTAGAAAATACTTTATTTGAAGATGGTGACGGCGCAAA

TACATTCCGTGCTTTTAACCCAACCCAAGCCGAAGAAACTTATTCAATGGTCACTGCTAA

CCGTTTTTGGTCCCAAATCTTTGGGGTTGCTTTTTCCAATAAACGTTGGTTACATTTCTT

TATGCTATTTGTACCAGTAACCGGTTTATGGATGAGTGCTATTGGGGTAGTCGGTCTGGC

TCTGAACCTACGTGCCTATGACTTC-GTTTCGCAAGAAATCCGTGCAGCGGAAGATCCTG

AATTTGAGACTTTCTACACCAAAAATATTCTCTTAAACGAAGGCATTCGTGCTTGGATGG

CGGCTCAGGATCAGCCCCATGAAAACCTTATATTCCCTGAGGAGGTTCTACCCCGTGGAA

ACGCTCTTTAAATGTCTGGAAGCACGGGAGAACGTTCTTTTGCTGATATTATTACCAGTA

TTCGATACTGGGTCATTCATAGCATTACTATACCTTCCCTATTCATTGCGGGTTGGTTAT

TCGTCAGTACAGGTTTAGCTTACGATGTGTTTGGAAGTCCTCGGCCAAATGAATATTTCA

CAGAGAGCCGGCAGGGAATTCCATTAATAACCGGCCATTTTGATTCTTTGGAACAACTTG

ATGAATTTAGTAGATCCTTTTAGATGACCATAGATCGAACCTATCCAATTTTTACAGTGC

GATGGTTGGCTGTTCATGGACTAGCTGTACCTACCGTTTCTTTTTTGGGGTCAATATCAG

CGATGCAGTTCATCCAACGATAA---------ATGGCTACACAAACCGTTGAGGGTAGTT

CTAGATCTG------GACCAAGACGAACTACTGTAGGGAGTTTATTGAAACCATTGAATT

CGGAATATGGCAAAGTCGCTCCGGGTTGGGGAACTACACCACTTATGGGGGTTGCAATGG

CTCTATTTGCGATATTTCTATCTATTATTTTGGAAATTTATAATTCTTCTGTTTTACTGG

ATGGAATTTCCATGAAT------TAG------------------ATG-----------CT

TACTTTAAAACTCTTCGTTTATACAGTCGTGATATTCTTC---------------GTTTC

TCTATTCATCTTCGGATTCTTATCTAATGATCCAGGTCGCAATCCTGGACGTGAGGAA--

----TAAATGGCCGATACTA---------------------CTGGA---AGAATTCCTCT

TTGGCT---GATAGGTACTGT-AGCTGGTAT-TCTTGT-GATCG-GTTCAATAGGTATCT

T-------------CTTTTATGGCTCA-TATTCCGGGTTGGGCTCATCTCTGTAGATGCT

TAATA---TCTTTAGTTTAATCTGTATCTATCTTAATTCTGTCC---TTTATTTGAGTA-

-----GTCTTTTCTTCGCTAAATTGCCCGAAGCTTATGCCGTTTTTAATCCAATAGTAGA

CTTTATGCCCGTCATACCCCTACTATTTTTTCTCTTAGCCTTCGTTTGGCAGGCTGCTGT

AAGTTTTCGATGAATGACACAAT---------CAAACCCGAATGAACAAAGTGTTGAATT

GAATCGTACCAGTCTATACTGGGGTTTATTACTTA-TTTTTGTACTTGCTGTTTTATTTT

CCAATTATTTTTTCAATTGAATGGAAGTCAATATTCTCGCATTTATTGCTACTGCACTGT

TCATTCTAGTTCCTACCGCCTTTTTACTTATCATCTATGTAAAAACAGTCAGTCAAAATG

AT---------TAAATGGAAACAGCAACCCTAGTCGCCATCTCCATATCCGGTTTACTTG

TAAGCTTTACTGGGTACGCCTTATATACCGCTTTTGGGCAACCCTCTCAACAATTAAGAG

ATCCATTCGAAGAACACGGGGATTAA---------ATGGAAGCATTGGTTTATACATTCC

TCTTAGTCTCGACTTTAGGGATAATTTTTTTCGCTATCTTTTTTCGAGAACCACCTAAGG

T---------TCCGACTAAAAAAATGAAATGAATGACTATTGCTTTCCAATTAGCTGTTT

TTGCATTAATTGCGACTTCATCAATCTTACTGATTAGTGTACCCCTTGTATTTGCTTCTT

CTGATGGTTGGTCAAGTAATAAAAATGTTGTATTTTCCGGTACATCATTATGGATTGGTT

TAGTTTTTCTGGTAGCTATCCTTAATTCTCTCATCTCTTGA-------------------

--ATGTCACCACAAACAGAGACTAAAGCAAGTGCTGGATTCAAAGCCGGTGTTAAAGATT

ACAAATTGACTTATTATACTCCTGACTACGAAACTAAAGATACTGATATCTTGGCAGCAT

TCCGAATGACTCCTCAACCTGGAGTTCCGCCCGAAGAAGCAGGGGCTGCGGTAGCTGCCG

AATCTTCTACTGGTACATGGACAACTGTGTGGACTGATGGACTTACCAGTCTTGATCGTT

ACAAAGGACGATGCTACCACATTGAGACTGTTATTGGGGAGGAAAATCAATATATTGCTT

ATGTCGCTTATCCTTTAGACCTTTTTGAAGAAGGTTCCGTTACTAACATGTTTACTTCCA

TTGTGGGTAATGTATTTGGTTTTAAAGCCCTACGAGCTCTACGTTTGGAGGATCTGCGAA

TTCCCCCTGCTTATTCCAAAACTTTCCAAGGCCCGCCCCACGGTATCCAAGTTGAACGAG

ATAAATTGAACAAGTACGGTCGTCCCCTGTTGGGATGTACCATTAAACCAAAATTGGGAT

TATCCTCAAAGAACTACGGTCGGGCGGTTTATGAATGTCTACGTGGTGGACTTGATTTTA

CCAAGGATGATGAAAACGTGAACTCCCAACCATTTATGCGTTGGAGAGATCGTTTCTTAT

TTTGCGCCGAAGCGCTTTTTAAAGCGCAAGCCGAAACGGGCGAAATCAAAGGGCATTACT

TGAATGCGACTGCGGGTACATGTGAAGAAATGATAAAAAGGGCTGTATTTGCTAGAGAAT

TGGGAGTTCCTATCATAATGCATGACTATATAACCGGGGGATTCACTGCAAATACTAGCT

TGGCTTATTATTGCCGCGACAACGGCCTACTTCTTCACATTCACCGTGCAATGCATGCAG

TTATTGATAGACAGAAAAATCATGGTATGCATTTTCGTGTACTAGCGAAAGCATTACGTA

TGTCTGGCGGAGATCATATTCACGCTGGTACAGTAGTAGGTAAACTGGAAGGGGAACGTG

AGATGACTTTAGGTTTTGTTGATTTATTACGTGATGATTTTATTGAAAAAGACCGAAGTC

GTGGTATTTTTTTCACACAAGATTGGGTCTCTATGCCAGGCGTTTTGCCCGTGGCTTCAG

GGGGTATTCATGTTTGGCATATGCCTGCCCTGACCGAAATCTTTGGGGATGATTCTGTAC

TACAGTTCGGTGGAGGAACTTTAGGACATCCTTGGGGGAATGCGCCTGGTGCAGTAGCTA

ATCGAGTGGCTTTAGAAGCATGTGTACAAGCTCGTAATGAGGGGCGTGATCTTGCTCGTG

AAGGTAATGAAATTATCCGT-----------------------GAAGCCGCTG-------

-------AATGGAGCCCTGAACTAGCCGCCGCTTGTGAAGTATGGAAGGAGATCAAATTC

GA---GTTCGAGCCGGTAGATAAGGGGGAT---CAGCTAGCATAG---------------

------------------------------------------------------------

------------------------------------------------------------

------------------------------------------------------------

------------------------------------------------------------

------------------------------------------------------------

------------------------------------------------------------

------------------------------------------------------------

------------------------------------------------------------

------------------------------------------------------------

------------------------------------------------------------

------------------------------------------------------------

------------------------------------------------------------

------------------------------------------------------------

------------------------------------------------------------

------------------------------------------------------------

------------------------------------------------------------

------------------------------------------------------------

------------------------------------------------------------

------------------------------------------------------------

------------------------------------------------------------

------------------------------------------------------------

------------------------------------------------------------

------------------------------------------------------------

------------------------------------------------------------

------------------------------------------------------------

------------------------------------------------------------

---------ATGATTCAACCTCAAACCCTTTTGAATGTAGCAGATAACAGCGGAGCTCGG

AAATTGATGTGTATTCGAATCATAGGG---GCTGGCAATCACCGATATGCTCATATTGGC

GACGTTATTGTTGCTGTAATTAAAGAAGCAGTGCCCAATATGCCTCTAGAAAGATCAGAA

GTAATTAGGGCTGTAATTGTGCGTACGTGTAAAGAACTTAAACGTGACAACGGTATGATA

ATACGATACGATGACAATGCCGCGGTTGTTATTGATCAAGAAGGAAATCCAAAAGGAACC

CGAGTTTTTGGTGCGATTGCCCGGGAATTGAGGCAGTTGAATTTTACTAAAATAGTTTCA

TTAGCCCCCGAGGTATTATAA---------------------------------------

------------------------------------------------------------

------------------------------------------------------------

------------------------------------------------------------

------------------------------------------------------------

------------------------------------------------------------

------------------------------------------------------------

------------------------------------------------------------

------------------------------------------------------------

------------------------------------------------------------

------------------------------------------------------------

------------------------------------------------------------

------------------------------------------------------------

------------------------------------------------------------

------------------------------------------------------------

------------------------------------------------------------

------------------------------------------------------------

------------------------------------------------------------

------------------------------------------------------------

------------------------------------------------------------

------------------------------------------------------------

------------------------------------------------------------

------------------------------------------------------------

------------------------------------------------------------

------------------------------------------------------------

------------------------------------------------------------

------------------------------------------------------------

------------------------------------------------------------

------------------------------------------------------------

------------------------------------------------------------

------------------------------------------------------------

------------------------------------------------------------

------------------------------------------------------------

------------------------------------------------------------

------------------------------------------------------------

------------------------------------------------------------

------------------------------------------------------------

-----------------------------------------ATGACCAGAGTTAGACGAG

GATATACAGCTCGGAGGCGTAGAACCAAACTCCGTTTATTTGCATCAACCTTTAAAGGGG

CTCATTCAAGACTTACTCGAACGAGTACTCAACAGAAAATGAGAGCTTTGGTTTATGCTC

ATCGAGATAGAGGCAGGCAAAAGAGAAACTTTCGTCGTTTGTGGATCACTAGGATAAATG

CAGTAACTCGCGAGAATAAAATGGTATTCTATAGTTATAGTCGGTTAATGCATAACTTGT

ACAAGAGGCAATTGCTTCTTAATCGTAAAATACTTGCACAAATAGCTATATCAAATAAGA

ATTGTCTTTGCATGATTTCCAATAA------G-ATTAGC-AAATAA--------------

---------ATGATAAAGAGATTGC--------------GTTCGAATACAGAAG------

---------CCAAAG---------------------------------TTTTAGCTC---

-------------------AACATATACATATGTCTGTTTTCAAAGCACGAAGAGTAATT

GATCAGATTCGTGGGCGTTCCTACGAGGAAACACTCATGATACTGGAACTCATGCCTTAT

CGAGCATCTTATTCTATTTTGAAATTGGTTTATTCCGCAGCAGCAAATGCTAGTCATAAT

CTGGGCTTGAACGAAGCTGATTCATTCATTAGTAAGGCTGAAGTCAACGGGGGTACTGTC

GTAAAAAAGTTAAGGCCTCGGGCTCGAGGGCGCAGTTATACGATAAAAAGACCTACTTGT

CATATAACAATTGTATTGAAGG-------ATAAAT--------CGAAATTTAAT------

----------------------GAAATGAATTTTAGATAA-----------ATGGATGGA

ATCAAATATGCAGTATTTACAGAAAAAAGTATTCGGTTATTGGGGAACAATCAATATACT

TCTAATGTCGAATCAGGATCAACTAGGACAGAAATAAAGCATTGGGTCGAACTCTTCTTT

GGTGTCAAGGTAATAGCTATGAATAGTCATCGAC------TCCCGGGAAAGGGTAGAAGA

ACGGGACCTATTATGGGACATACAATGCATTACAAACGTATGATCATTACGCTTCAACCG

GGTTATTCTATTCCACCTCG-TATAGAGAAAAGAACTTAA--------------------

------------------------------------------------------------

------------------------------------------------------------

------------------------------------------------------------

------------------------------------------------ATGGCCAAGGGT

AAAGATGTTAGAGTAAGAGTTATTTTAGAATGTACTA---GTTGTGCCCG------AAAC

GATGTCAAT------AAAGAATCGCCTGGCATCTCTAGATATATTACTCAAAAGAATCGA

CACAATACACCCAATCGATTAGAATTGAGAAAATTTTGTCGCTATTGTCACAAACATATG

ATTCATGGAGAAATAAAGAAA------TAGATGAAAATAAGGGCTTCTGTTCGTAAAATT

TGTGAAAAGTGTCGCCTAATCCGTAGGCGCGGGCGGATTATAGTGATTTGTTCCAATCCG

AGACATAAACAAAGGCAAGGATAAATGGTTCGAGAGGAAGTAGTAAGATCCACTCGAACA

CTACAGTGGAAGTGTGTTGAATCAAGAGTAGACAGTAAGCGTCTTTATTATGGTCGTTTT

A-TTCTGTCC--CCGCTTATGAAAGGTCAAGCCG-ATACCATA-GGTATT-GCCGTGCGA

AGGG--CTTTACTTGGAG-AAATGGAAGGAA--CATGTATC----ACACGCGCAAAATCT

----GAGAAGGTACCACATGAATATTC--------GACGATAGTAGGTATTGAAGAATCC

GTGCACGAAATTTTAATAAATTTGAGAGAAATTGTATTGAGAAGTAATCTCTATGGGATT

ATAGATGCATCTATTTGCATCAGGGGTCCTAGATACGTAACTGCTCAAGATATTATCTCA

CCACCTTCTGTAGAAATAGTTGACACAACACAGCATATAGCTAAACTAACGGAACCAATT

GATTTGCGTATTGAATTACAAATCCATAGGGATCGCGGATATCGTATGAAATCCACAAAT

AATTCTCAAGATGGAAGTTA----------CCCTATAGATGCTGTATCCATGCCTGTTCG

AAATGCAAATTATAGTATTCATTCTTATGGGAATGGAAATG------AAAAACAAGAGAT

ACTTTTTCTAGAAATATGGACGAATGGAAGTTTAACTCCTAAGGAAGCACTTTATGAAGC

TTCTCGTAATTTGATTGATTTATTTATTCCTTTTCTACATGCGGAGGAAGAGGAGGGCAT

------TAATTTCAAGGAAAGTAAAAATAGGTT---TACTCCACCCCCTTTTACCTTTAA

AGACGGATTAACT------AATCTAAAGAAAAATAAAAAAGTAATTCCATTGAAATGTAT

TTTTATTGACCAATTAGAATTGCCTTCCA-GGACCTATAATTGTCTCAAAAGGTCCAATA

TACATACATTATTGGACCTTTTGAGTAACAG------TCAAGAAGACCTTATGAGCATTG

AATATTTTCGCATAGAAGATGTAAAGCGGATATCGGACACCTTA-CAGAAGCATTTTGC-

---AATCGATTTA--CCTAAGAATA------AGTTTTTATTT---TAA------------

---------------------------------------------------ATGTTCCGT

AA---TGGAAATGAGGGAATGTTCACAATACCTGGATTTAGTCAGATCCAATTTGAGGGA

TTTTGTAGGTTCATGAATCAGGGCTTGACGGAAGAATTTCATAAGTTTCCAAAAATTGAA

GATACAGATCAAGAAATTGAATTTCAATTATTTGCAGAAAGATATCAATTGGTAGAACCC

TTGATAAAAGAAAGAGATGCTGTGTATGAATCACTCACATATTCTTCGGAATTATATGTA

CCCGCGGGATTAATTTGGAAAACGGGTAGAGATATGCAAG--AACAAACCGTTTTTATTG

GAAATATTCCTCTAATGAATTCCCTGGGAACCTCTATAGTAAATGGAATATACAGAATTG

TAATCAATCAAGTATTGCAAAGTCCCGGGATTTACTACCGTTCAGAATTGGACCATAACG

GAAT---TTCTGTCTATACCGGGACTATAATATCAGATTGGGAGGGGAGGTTGGAATTAG

AAATTGATAAAAAGGCAAGGATATGGGCCCGCGTGAGTAGGAAACAAAAAATATCTATTC

TAGTTCTATCATCAGCTATGGGTTCGAATCTAAGAGAAATTCTAGATAATGTTTGTTACC

CTGAAATTTTC------------TTGTCTTTCCCGAACGATAAGGAGAAA-------CAA

AAGA-----TTGGGTCAA-AAGAAAATGCTATTTTGGAGTTTTATCAGCAATTTGCTT--

-----GTGTA--GGCGGAGATCCGGTATTTTCTGAGTCCTTATGTAAGGAATTGCAAAAG

AAATTTTTTCAACAAAGATGTGAATTAGGAAGGATTGGTCGACGAAATATGAACCGGAGA

CTAAATCTTGATATACCTCAGAACAATACATTTTTGTTACCACGAGATGTATTAGCTGCT

GCGGATCATTTGATCCGAATGAAATTTGGAATGGGCGCACT------TGACGATATGAAT

CATTTGAAAAATAAACGTATTCGTTCTGTAGCAGATCTATTACAAGATCAATTCGGATTG

GCTCTTGTTCGTTTAGAAAATACGGTTCGAGGAACTATATGTGGAGCA------------

---ATCCGGCATAAATTGATACCGACTCCTCACAATTTGATAACTTCAACTTC-------

-----ATTAACAACTACTTATGAATCGTTTTTTGGTCTACACCCCTTATCTCAAGTTTTT

GATCGAACTAATCCATTGACACAAATCGTTCATGGGCGAAAATTGAGTTATTTGGGCCCT

GGGGGATTGACGGGGCGAACTGCAAGTTTTCGGATACGAGATATACATCCTAGCCATTAC

GGGCGTATTTGCCCAATTGACACGTCCGAAGGAATCAATGTTGGTCTTATTGGATCCTTA

GCTATTCATGCGAGGATTGGCCCG---TGGGGATCTATAGATAGCCCATTTTATGAAATA

T---CTGCGAGATC------AAAAGAGAC---ACAGATGATTTATTTATCACC------A

AGTAGAGATGAATATTAT------ATGGTAGCAGCAGGAAATTCTTTGGCCTTGAATCGG

---AGTATTCAGGAAGAGCAGGTTGTTCCAGCTCGATATCGTCAAGAATTCCTGACTATT

GCGTGGGAACAGATTCATCTTAGAAGCATTTTCCCTTTCCAATATTTTTCTATTGGAGCT

TCCCTTATTCCTTTTATTGAACATAATGATGCAAATCGGGCTTTAATGAGTTCTAATATG

CAGCGCCAAGCAGTTCCGCTTTCTCGATCCGAGAAGTGCATTGTTGGAACTGGACTGGAA

CGCCAAACAGCACTAGATTCGGGGCTTTCAGTTATAGCCGAACGTGAGGGAAAGGTCATT

TATACTGATACTCACAAGATCATTTTCTCGAGTAATGGAGACACGAGAAATATTCCATTA

GTTATATATCAACGTTCCAACAAAAATACTTGTATGCATCAAAAACCCCGGGTTCTGCGG

GGTAAATACCTTAAAAAGGGACAAATTTTAGCGGATGGTGCGGCTACAGTTGGAGGGGAA

CTCGCTTTAGGAAAAAACGTATTAGTAGCCTATATGCCATGGGAAGGTTACAATTTTGAA

GACGCAGTACTAATTAGCGAACGTCTAGTATATGAAGATATTTATACTTCTTTTCACATC

CGAAAATATGAAATTCAGACTCATGTGACAAGCCAAGGCC---CTGAAAGAATTACTAAA

GAAATACCGCATTTAGAGGCCCATTTATTCCGCAATTTAGATAGAAATGGAATTGTTATG

CTGGGATCTTGGATAGAAGCAGGTGATATTTTAGTAGGTAAATTAACACCTCAAACAGCG

AACGAATCGTCGTATGCTCCAGAGGATAGATTATTACGAGCCATACTTGGTATTCAGGTA

TCCACGGCAAAAGAAACTTCTTTAAAACTACCTATAGGCGGAAGGGGCCGAGTTATTGAT

GTGAGATGGATCCA----------------------------GAAAAAA-----------

---------------------GGGGGTTCCAGTTA-------------------------

--------------------TAATCCAGAAATGATTCGTGTATATATTTTACAGAACCGT

GAAATCAAAGTAGGTGATAAAGTAGCTGGAAGACATGGGAATAAGGGTATCATTTCAAAA

ATTTTTCCTAGACAAGATATGCCTTATTTGCAAGATGGAACACCTGTTGATATGGTCTTC

AACCCATTAGGAGTACCCTCACGAATGAATGTGGGACAGATTTTTGAATGCTCGCTCGGG

TTAGCGGGGGATTTGCTCAAGAGACATTATAGAATAGCGCCTTTTGATGAGAGATATGAG

CAAGAGGCTTCTAGAAAACTAGTGTTTCCTGAATTATATGAAGCTAGTCAACAAACAAAA

AATCCATGGGTTTTTGAACCCGAGTATCCAGGAAAAAGCAGAATATTTGATGGAAGAACA

GGAGATCCTTTTGAACAACCTGTTCTAATAGGAAAGTCCTATATCCTGAAATTAATTCAT

CAAGTTGATGATAAAATCCATGGGCGTTCCAGTGGGCATTACGCACTTGTTACACAACAA

CCTCTTAGAGGAAGGGCTAAGCAAGGGGGACAGCGAGTAGGAGAAATGGAAGTTTGGGCT

TTGGAGGGATTTGGCGTTGCTCATATTTTACAAGAGATGCTTACTTATAAATCTGATCAT

ATTAGAGCTCGTCAAGAAGTACTTGGTGCTACGATCATTGGAGGAACAATACCTAACCCT

GAGGATGCTCCAGAATCTTTTCGATTGCTCGTTCGAGAACTACGATCCTTAGCTCTGGAA

CTGAATCATTTCCTTGTATCTGAGAAGAATTTTCAGATTAATAGGAAGGAAGCT------

---TGA---------------------ATGATCGATC---GGTATAAACACCAACAACTT

CGAATTGGGCTAGTTTCTCCTCAACAAATAAGGGCCTGGGCTAACAAAATCCTACCTAAT

GGAGAGATAATTGGAGAAGTGACAAAACCCTATACTTTTCATTACAAAACCAATAAACCG

GAAAAAGATGGATTGTTTTGCGAAAGAATCTCTGGGCCTATAAAAAGTGGAATTTGTGCT

TGTGGAAATTA-----------------------TCGAGTAATCGGAGCTGAAAAAG---

-AAGTCCCGAAATTTTGTGAACAATGCGGGGTGGGATTTGTTGATTCTCGGATACGAAGA

TATCAAATGGGATACATCGAACTCGCATGTCCAGTAACTCATGTGTGGTATTTGAAACGT

CTTCCTAGTTATATCGCGAATCTTTTAGATAAACCCCTTAAAGAGTTAGA------AGGT

CTAGTGTACTGCGATGTGTGATTTGATCG-AAATTTTCATTTTACAGATTCGTAATAAGA

AACTGTCATCCCATTCAATCTGGTT---GGGATGTCCCCGACTCTGACATGTGTCTTGGG

AGGAGTAACATGAAGCTCAGAACTATGGGTGTATTCAATACTTC----G-AACTGAAAGG

GGAGTTGATCCATGGTCGAT---T-CTGTAACAGATAAATAA-ATAGGAATTGCTAGTTA

TACCTCGTAGA-------------AAAAAAAAGATTTTTGTGGAATT------CGCCATT

CCATCCCTTCT----CTTTTATATAGAAATAAAATTCTGTTCAAGCAAGC--AA----A-

---TATGGTATGGTTACAGAAGTCTATTTATCGCATATATG-----CTTCAAGGGGCATC

ATG-GCATA-------ACCATTGAGGTG-AGTAGGG---------ACCT--AAAAGATCG

AACAAAACGATATATAG-----ACAAGTAAATCCCTTACGAGTCCCGAAGTATTCCTTT-

----AAGGGGATTCATCATTTGAGGGGAAGTAGACTACTCAATA-----ATTTCATATTT

CCATTTT------TGTCGGAAAGTCAAAATGAAAAATTAAGTAATTCAAAAAGTCGTTC-

-A-AATCAAAAAATG-AATCA-ATGAAA----GATTGGTC----TTTACTGGGAAAACTT

GAGTAAGGAGTAG-------TTTTT-----------GTAGGGTTTTATCAAATAAAGTTT

CAAAACAAAATTAAGAACCCCTTTCTTTTATTTAGTGTAACTGCTTGAGCCGGATGAGAG

GAAACCTTCACGTCCGATTTTTA-AGGGGGGAATCCCAGTCTATGGGAACCTATCTCGAT

TTTTCTTTTGCTAGGCCCATAACTAAAAAACCCACTTTCTTACGATTAAGAGGTTCATTC

GAATACGAAA---TCCAATCCTGGAAATACAGCATCCCGCTTTTTTTTAC----------

-----------TACTCAAGGTTTTGACACATTTCGAAATAGAGAAATCTCTACGGGAGCA

AGTGCTATCATAGAGCAATTAGCCGATTCGGATTTGCGAATTATTATAGATAATTCATTA

GTAGAATGGAAGGAATT---------------------------AGGAGACGGAGGGTCT

------ACTGGAAATGAGTGGGAAGATAGAAAAATTAGAAGAAGAAGGGATTTTTTGGTT

AGACGTATGGAATTAGCCAAACATTTTATTCGAACAAATGTAGAACCAGAACGGATGGTT

TTGCGCCTATTACCAGTTCTTCCTCCAGAGCTGAGACCTATCATTCAGATAGATGGGGGT

AAACTAATGAGT---TCAGATATTAATGAACTCTATAGAAGAGTTATCTATCGGAACAAT

ACGCTTACCGATCTATTAACAA--CAAGTAGATCGGCGCC-------------GGGCCAA

TTAGTAATGTGTCAGGAGAAATTGGTACAAGAAGCCGTGGATACACTTCTGGATAATGGG

ATCCGGGGACAGCCTATGAGGGATGGTCATAATAAAGTTTACAAGTCATTTTCAGATGTA

ATTGAAGGCAAAGAGGGGAGATTTCGCGAGACTCTGCTTGGTAAACGAGTCGATTATTCG

GGACGTTCCGTCATTGTCGTGGGTCCTTTGCTTTCATTACATCAATGTGGATTACCTCGA

GAAATAGCAATAGAGCTTTTCCAAACATTTGTCATTCGCGGTCTAATCAGACAACATGTT

GCTTCTAACATAGGGATTGCTAAAAGCAAAATTCGGGAAAGAGAACCAATTGTATGGGAA

ATACTTCAAGAAGTTATGCAGGGGCATCCTATATTGTTAAATAGAGCACCTACCCTGCAT

AGATTAGGCATACAGGCGTTCCAACCCATTTTAGTGGAGGGACGCGCTATCTGTTTACAC

CCCTTAGTTTGTAAGGGCTTTAATGCAGACTTTGACGGGGATCAAATGGCTGTTCATGTA

CCTTTATCTTTGGAGGCTCAAGCAGAGGCCCGTTTCCTTATGTTTTCTCATATTAACCTC

CTTTCTCCAGCTATTGGAGATCCTATTTCCGTACCAACTCAAGATATGCTTATAGGACTC

TATCTATTAATGATCGGGAATCGTCGAGGTATTTGTGCAAATAGATATAATCCA------

TATAATTGCGGAAACTATAAAAATAAAAGAGTTGAGAATAAAG---------ACTATAAC

------TATACAAAAGAGAAAGAGCCGTCTTTTTGTAGTTCGTATGATGCGCTTGGAGCT

TATCGACAGAAACGAATCAATTTAGATAGTCCTTTGTGGCTACGGTGGCGAT---TAGAT

CAACGCGTCATTGGTTCAAGTTCA------AGGGAAGTTCCCATCGAAGTTCAATACGAA

TCTTTGGGTACGTATCATGAGATTTATGGGCACTATCTAATAGTGGGAAGCGTAAAAAAA

GAAATCTGTTGTGTATACATTCGAACCACTGTTGGTCATATTTCTTTTTATCGAGAAATA

GAAGAGGC----------CATACAAGGGTTTTATCGGG--CCTACTCATACA--------

--------------CTATCTAA------------------------ATG--GCAGAACGG

GT---------------CGATCTGGTCTTTCACAATAAAGTGATAGATGGAACTGCTATG

AAACGACTTATTAGCAGATTAATAGATCATTTCGGAATGGCATATACATCACATATTCTG

GATCAAGTGAAGACTTTGGGTTTCCAGCAAGCCACTTCTACATCTATTTCATTAGGAATT

GATGATCTTTTAACAATACCGTCTAAGGGATGGTTAGTTCGAGACGCTGAACAACAAAGT

TTTATTTTGGAGAAATACCATCATTATGGGAATGTAAACGCGGTAGAAAAATTACGTCAA

TCCATTGAGATATGGTATGCTACAAGTGAATATTTAAGACACGAAATGAATCCTAATTTT

CGGATGACCGATCCTTCTAATCCAGTCCATCTAATGTCCTTTTCGGGAGCTAGAGGAAAT

GCATCTCAGGTGCACCAATTAGTGGGTATGAGAGGATTAATGTCGGATCCCCAAGGACAA

ATGATTGATTTACCCATTCAAAGTAATTTACGCGAAGGACTTTCTTTGACAGAGTATATA

ATTTCCTGTTACGGAGCCCGCAAAGGGGTTGTGGATACTGCTGTACGAACATCAGATGCC

GGATATCTCACGCGTAGACTTGTTGAAGTAGTTCAACACATTATTATACGTAGAACGGAT

TGTGGTACTATCCAAGGCATTTCCATAAGTCC---------TCGAAA------TGATATG

ACGGAAAAAGTTTTTGTCCAAAC------ATTAATGGGTCGTGTATTGGCAGACGATATC

TATCTGGGTCTACGATGTATTGCCGCTCGAAATCAAGATATTGGGGTTGGGCTTGTCAAT

CGATTCATCACC------------------TTTCGAGCACAACGAATATATATTCGAACA

CCCTTTACTTGCAGAAGTACATCTTGGATCTGTCAATTATGTTATGGTCGGAGTCCCACT

CGCGGTGACTTGGTCGAATTAGGGGAAGCTGTAGGTATTATTGCGGGTCAATCAATTGGG

GAACCGGGCACTCAACTAACATTAAGAACCTTTCATACCGGTGGAGTATTCACGGGTGGT

ACTGCCAAACATGTGCGAGCTCCTTCTAATGGAAAAATAAAATTCAATGAGGGTTTGGTT

CATCCCACGCGTACCCGTCACGGGCACCCTGCTTTTTTATGTTCTATAG---ACTTGTAT

GTAACTAT---TGAGAGTCGGGATATTATACATAATGTGAATATTCCACCAAAGAGTTTG

ATTTTAGTTCAAAATGATCAATATGTGGAATCAGAACAAGTGATTGCTGAGATTCGTTCT

GGAACGCCCACTTTTCATTTTAAAGAGAGGGTTCGAAAACATATTTTTTCTGAATCAGAG

GGAGAAATGCACTGGAGTACCGATGTCTACCATGCACCCGAATATACATATGGTAATGTT

CATCTATTACCCAAAACAAGCCATTTATGGATATTAGCAGGAGGTCCCCGCGAG------

TCTAGTATAG---TGTCTTTCTCGCTCCACACAGATCAGGATCAAATGAATGTTCATTCG

TTTTCTGGCGAAGGCAAA------TATATCTCTGACCCTTCAATGACTA---ATGATCGG

ATAAGGCAGAAATTTTT------GGATACTTTTAGTAAAAA---------AGATAGAGAA

ATTCTTGCCTATTCA---AT---ATCGGA--TCGAATCATATCCAAAGGTAAAGGTCATT

GGAATTTCCTAAATCCTTC------TATGCTCCCAGAGAATTCTTATTTTTTGGCGAAAA

GGCGAAGAAATAGATTCATCATCCCAATACAATATGATC----AAGAGCGGGGGAAAGAG

CCGA----------TAACTCGTT------TTGGTATTTCAATTGAAATACCCATAAATGG

TATTTTGCGTGAAAATAGCATTCTTGCTTATTTTGATGACCCACGATACAGAAGAAACA-

-----GTTCGGGAATTACTAAAT-------------------------------------

------------------------------------------------------------

------------------------------------------------------------

------------------------------------------------------------

------------------------------------------------------------

------------------------------------------------------------

------------------------------------------------------------

--------------------------------------------------ATGGGATCGT

------------------------------------------------------------

------------------------------AGAGGTAGATTCCGTCGTAAAAAAAGAGGA

TTTGATTGAGTATCGAGGAGCAAAAGAATTTAGTCTGAAAAAC------CAAATGAGAGT

AGATCGGTTTTTTTTCATTCCCGAAGAAGTGCATATTTTACCCGGATCCTCGCACATAAT

GGTTCGGAACAATACTATCATTGGAGTAGATACACGACTTGCTTT------AAATACAAG

AAGCCGAGTAGGCGGATTAGTCCGAGTGGAGAGAAAAAAAAAA-GTATTGAACTGAAAAT

CTTTTCTGGAGATATTCATTTTCCCGGGGAAACGGATAAGATATCCAGACACAGCGGTAT

TTTGGTACCACCAAGCACGA---GAAAAAAAAATCCTAAGGAATCCAAAA---AAT----

-----GGAAAAATTGGATCTATGTTCA---ACGGATCACACCTACCAAAA------AAAA

GTATTTTGTTTCGGTTCGGTCCGTAGTCACATATGAAATAGCTGATGGAATAAATTTAGC

AACACTTTTACCCCAGGATCTCTTGCAAGAAAAGGATAATGTCCAACTTAGAGTTGTCAA

TTATATCCTTTATGGATATGGCAAGTCAATTCGAGGAATATATCACACAAGTATTCAATT

AGTTCGTACTTGCTTAGTATTGAATTGGGACCAAGAGCAAAACGGTTCTCT---GAAAG-

--AAGTTCATGCTTCCTTTGTTGAGATAAGGGCAGATGGTCTAATTCGTGATTTCATCAA

AATGGAGTT------AACCAAGTCCACTATTTTGTATAGTGGAAAAAGGTATCATACGGT

CGGTTCAGGATTGATTTCCGAAAAGGAATTAGATCGCACCAATAT---CAACCCTTTTCA

GTCCAAGGCGAAGATTCAATCACTTACCCAACATCAAGGAACTATTAGTGTTGGTACGTT

ATTGAATCAAAATAAAGAATGCC------AATCTTTCATAATTTTGTCATCATCCAATTG

TCTTCGAATGGATCCATTCAATGATTTGAAATATAATAATGTGACAAAGAAGGG------

---------GGATTCCCTAATTTCAATTA---------------------GGGGTTTGTT

GTGTCCCTTGGGTACTATTGTACCTAAAATA------GCCCA---TTTTTATTCATTTTA

CCATTTAGTAACCCATAATCAG---------ATCTTGTTAAAGAAATCTTTGTTACT---

---TGACAATTTTAAACAGACTTTCCAAATGCTTCAAGCAC------TAAAATACTGTTT

AATGGATGAAAATGGGCGGGTTTATAATTATAATCCCGATCCGTGCAGTAACATCATTTT

GCATCCATTCTATTTGAATTGGTGTTTTCTCCATCACGA------TTCTTGTGAAGATGC

ATTCAC------AAGAATTCACCTTGGACAATTTATTTGTGAAAATGTGTGTCTATTCAA

ATACGAACC------ACACATAAAAAAATCCGGTCAAATTATAATTATTCATATTGACTC

CTTAGTTATAAGATTGGCTAAGCCCTATTTGGCTACCCCGGGAGCAACCGTTCATGGCCA

TTATGGGGGAATCCTTTCTGAAGGGGAGACATTAGTTACATTTATATATGAAAAATCAAG

ATCTGGTGACATAACGCAGGGTCTTCCAAAAGTGGAACAAGTCTTAGAAGTCCGTTCAAT

TGATTCAATATCGATGAACCTAGAAAAGAGAGTTGAAAGTTGGAACGAACGTATACCAAG

AATTCTTGGAATCCCCTGGGGATTCTTGATTGGGGCTCAGCTAACCATAGCCCAAAGTCG

TATCTCTTTGGTTAATAAGATTCAAAGGGTTTATCGCTCTCAAGGGGTACAGATCCATAA

TAGACATATAGAAATCATTGTACGTCAAATAACATCAAAAGTATTGGTTTCAGAAAATGG

AATGTCCAATGTTTTTT-GCCTGGAGAATTAATTGGATTGTTGCGAGCGGAACGAGCAGG

GCGTGCTTTAGACGAAGGAGTCGGTTATCGGGCAATCTTATTGGGAATAACGAGGGCATC

TCTGAATACTCAAAGTTTTATATCTGAGGCGAGTTTTCAAGAAACCGCTCGAGTTTTAAC

AAAGGCTGCTTTACGGGGTCGCATTGATTGGTTGAAAGGTCTTAAAGAGAATGTTGTTTT

GGGGGGGATTATACCTGTTGGTACCGGGTTCAAAAAATTAG-TACACCGTTCAT-CAAG-

------ACAAGACAAAAACATTTATTTGGAAATCAAAAGGAAGAATCTATTTGA-ATTGG

AAACGAGA---GATATCTTGTTACACCATAGAGAATTATTTTAGAGAATTATTTTGTTCT

TTCG-CC-----CCAAACAATTTCCATGAG---------------GCATCAGAACAATCA

TTT---------------------------------ACGCGATTTAATGATTCCTAAATG

ACA---------------------------------------------AGAAGATATTGG

AACATCAATTTGGAAGAGATGATGGAA-GCAGG--------AGTTCATTTTGGCCATGGT

ACTCGGAAATGGAATCCTAGAATGGGCCCTTACATCTCTGCAAAGCGCAAAGGTATTCAT

ATTATAAATCTTACTAGAACGGCTCGTTTTTTATCAGAAGCCTGTGATTTAGTTTTTGAT

ACAGCAAGTAAAGGAAAAAACTTTTTAATTGTCGGTACCAAAAAGAAAACGGCAGATTTA

GTAGCATCAGCTGCAATAGGGGCTCGGTGTCATTATGTTAATAAAAAATGGCTCGGTGGT

ATGTTAACAAATTGGTCCACTACGGAAACGAGGCTTCATAAGTTCAGGGACTTAAGAGCG

GAACAAAAGATGGGGAAACTCAACC---ATCTCCCAAAAAGAGATGCAGCAATGTTGAAG

AGAAAATTATCTACCTTGCAAGCATATCTGGGTGGAATCAAATATATGACGGGATTACCT

GATATTGTAATCATTGTTAATCAGCAAGAAGAATATACGGCTCTTCGAGAATGCGCCATT

TTAGGGATTCCGACCATTTGTTTAATCGATACAAATTGTGACCCCGAGATCGGGGATATT

TCGATTCCAGCCAACGACGATGCTATAGCTTCAATTCGATTCATTCTTAACAAATTAGTA

TTCGCAATTTGC----GAGGGTCGTTCTAGCTATATAAG------------AAATCGTTG

AATGGGACAAAAAATAAATCCACTTGGTTTCAGACTTGGTACA--ACCCAAAATCATCAT

TCCTTTTGGTTCGCACAACCAAAACATTTTTCTGTGGGTTTACAAGAAGATGAAAAAATA

CGGAATTGTATCAAGAACTATGTACAAAAAAATCAGAGAG--------------------

-------------------------------------------------CATCCTCAGGT

TTTGAA------GGAATTGCACGTATAGGAATTCAAAAAAGAATTGATTTGATCCAAGTC

ATAATCCATATTGGATTCCCAAAT---TTATTAATAGAGGGCCGAACACGAGGAATCGAA

GAATTACAGATGAATGTACAAAAGGAGTTTCACTCCGTGAACCGAAGACTCAACATTGCC

ATCACAAGAGTTGAAAAACCTTATGGACAACCTAATATTCTTGCAGAATATATAGCTTTA

CAATTAAAAAATAGAGTTTCGTTTCGAAAGGCAATGAAAAAAGCCATTGAATTAACTGAA

CAAACGGATACAAAAGGAATTCAAGTGCAAATTGCGGGACGCATCGATGGAAAAGAAATT

GCACGTGTCGAATGGATCAGAGAGGGTAGGGTTCCCCTACAAACAATTCGCGCTAAAATT

GATTATTGTTCCTATACAACTCGAACTATCTATGGAGTATTAGGCATAAAAATTTGGATA

TTTGTAGACGAT---AAATAA------ATGTCTCGTTACCGAGGACCTCGTTTCAAAAAA

ATACGCCGTTTGGGGGCTTTACCAGGACTAACTAATAAAATCCCTAGATCCAGAAATGAT

CTTAAAAACCAATTGC----GTTCGG--GGAAAAGATCGCAATATCGTATTCGTCTAGAA

GAAAAACAGAAATTGCGTTTTCATTATGGTCTCACAGAGCGACAATTACTTAGATATGTG

CATATCGCCGGAAAAGCTAAAGGGTCAACGGGTCAAGTATTACTACAATTACTTGAGATG

CGTTTGGATAACACCCTTTTTAGATTGGGTATGGCTTCGACCATTCCCGGAGCCAGGCAA

TTAGTTAA--CCAT-AGACATATTTTAG--TTAATGGTC--GTGTAGT-GGATATACCAA

GTTATCGTTGCAAACCCC--GAGATATTA-TTACTAC--GAAGGATAAAC----AAAGAT

CGAAAGCTCTGATTCAAAATTATATTGCGTCATCCCCCCGCGAGGAATTGCCAAAACATT

TGACTAT---TGACCCATTCCAATATAAAGGATGGGTAAATCAAATAATAGATAG-TAAA

TGGATCGGTTTAAAAATAAATGAGTTGTTAGTCGTAGAATATTATTCCCGTCAGACTTGA

ATGTCACGTCGAGGTACTGCAAA------AGAAAAAACTGCAAAATCCGATCCAATTTAT

CGTAATCGATTAGTTAACATGTTGGTTAACCGTATTCTGAAACACGGAAAAAAATCATTG

GCTTATCAAATTATCTATCGAGCCTTGAAAAAGATTCAACAAAAGACAGAAACAAATCCA

CTATCTGTTTTACGCCAAGCAATACGTGGAGTCACTCCCGATATAGCAGTAAAAGCAAGA

CG---TGTAGGCGGATCGACTCACCAAGTTCCTATTGAAATAGGATCTACACAAGGAAAA

GCACTTGCCATTCGTTGGTTATTAAGGGCATCCCGAAAACGT---CCGGGTCGAAATATG

GCTTTCAAATTAAGTTCCGAATTAGTAGATGCTGCCAAGGGGAGTGGCGATGCCATACGC

AAAAAGGAAGAGACTCATAGAATGGCAGAGGCAAATAGAGCTTTTGCACATTTTCGTTAA

ATGGGTAAGGATACTATTGCCGATATAATAACTTCTATAAGAAATGCTGACATGGAAAAA

AAAGGAACAGTTCGAATAGCATCTACTAATATCATCGAAAACATTGTGAAAATACTTCTA

CGAGAAGGTTTTATTGAAAACGTTCGAAAACATCAGGAAAGTCACAAATATTTCTTGGTT

TCAACCCTGCGACAT------AGAAGGACTAGAAAAGG----------------------

------------------------------------------------------------

--------------------------------------CATATAT---------------

------------------------------------------------------------

------------------------------------------------------------

------------------------------------------------------------

------------AGAACTA------TTTTAAAGCGTATCAGCCGACCCGGTCTACGAATC

TATTCCAACTATCAACGAATTCCTAAGATTTTAGGCGGAATGGGGGTTGTAATTCTTTCT

ACTTCTCGCGGTATAATGACAGATCGAGAAGCTCGACTAGAACGAATTGGTGGAGAAATT

TTGTGTTATATATGGTGAATGACAAAACCTATATCAAGACTTGGTTCACGTAAGAATAGA

CGCATTAATTCACGC------------------------AAGAATGGACGAAGA------

------------------ATACCAAAAGGGGTTATTCATGTTCAAGCGAGTTTCAACAAT

ACCATTGTAACTGTTACAGATGTACGAGGTCGGGTGCTTTCTTGGGCCTCTGCCGGTACT

TCCGGATTCAAAGGCACAAGAAGAGGGACACCCTATGCTGCTCAAGCAGCAGCGGCAAAT

GCTATTCGTACAGTAGTTGATCAGGGTATGCAACGAGCAGAAGTTATGATAAAAGGCCCC

GGTCTCGGAAGAGATGCAGCATTACGAGCCATACGTAGAAGCGGTATACTATTAAGTTTC

GTACGTGATGTAACACCTATGCCACATAATGGATGCCGACCCCCTAAAAAAAGACGTGTG

TAGATGCCAACTATTAAACAACTTATTAGAAACACAAGACAGCCAATAAGAAATGTCACG

AAATCTCCCGCTCTTCGAGGGTGTCCTCAGCGTCGAGGAACATGTACTAGGGTGTAT-AC

TATCACCCCCAAAAAACCAAACTCTGCCTTACGTAAAGTTGCCAGAGTACGATTAACCTC

TGGATTTGAAATCACTGCTTATATACCTGGTATTGGCCATAATTTACAAGAACATTCTGT

AGTATTAGTAAGAGGAGGAAGGGTTAAGGATTTACCCGGTGTGAGATATCACATTGTTCG

AGGAACCCTAGATGCTGTCGGAGTAAAGGATCGTCAACAAGGGCGTTCTAGTGCGTTGTA

G-----------------------------------------------------------

------------------------------------------------------------

------------------------------------------------------------

------------------------------------------------------------

------------------------------------------------------------

------------------------------------------------------------

------------------------------------------------------------

------------------------------------------------------------

------------------------------------------------------------

------------------------------------------------------------

---------ATGGCAAGAAAAAGTTTGATTCAGAGGGAGAAGAAGCGGCAGAAATTGGAA

CAGAAATATCATTTGATTCGTCGCTCCTTAAAAAAAGAAAT---AAGCAAAGTTCCATCG

TTGAGT------GATAAATGGGAAATTCATCGAAAATTGCAATCCCTACCACGCAATAGT

GCACCTATACGTATCCATCGGCGTTGTTTTGTGACTGGAAGACCTAGAGCTAACTATCGA

GACTTTGGGCTATCCGGACACATACTTCGAGAAATGGTTCATGCATGTTTGTTACCGGGC

GCAACAAGATCGAGTTGGTAA------------------------------------ATG

GT---CCAAAATTCATTCATTT-CAGTTATTCTA------CAAGAAGAAAAAGAAGAA--

----ACAAAGGGGTCTGTTGAATTTCAAGTATTCAGTTTCACCAATAAGATACGGAGGCT

TACTTCCCATTTGGAATTAAACAAAAAAGATTTTTTATCACAAAGAGGCCTAAAAAGAAT

TCTGGGAAAACGTCAAAGGCTGTTGGCTTATTTGTCAAATAAAAATAGAGTGCGTTATAA

GAAATTAATAAGTCAATTGAATATTCGGGAGCCAAAAACTCGTTAAATGGTAAAACTTCG

TTTGAAACGATGTGGTAGAAAGCAACGCGCGACTTGAAGGACGTGATCTGC--CGTGGAT

TCTTACATCCACCATTTTCTATAAGAATGAAGATGCTCCCGGCTCGACGTAGTTTGTTCT

GTTCCACCGGGA---ATCTAA-------TAGATTCC------------AATCTAA--AT-

----------------------------------------AGTACATGATGAAGCTCGAG

-TAGAA-AA-----GATGGA----TTTATT-TTTC-AGGAGGAAGAATCTAGGGTTAGTA

AC------AATTAATAAGTCGTACCAACTTTGTAA---GTATATCCTC------------

-------------------AATATATAGATCGAAAAGTG----------AAAATTTCAAT

AA-----GTTTGAAATAGAA--------AAGTAATATTTGTCGGAAT-TGATAACACTTT

TTTGATCAAAAGTCAAGTGTATCATAAAGAAATC----------AATCGTTCATATT---

----TCTTTACTATAGAATAGAAAAGAATAGAAAGAAAAAGAAATAATAATAAACAAAAG

GTATGTTGCTGCCTCTTTGAAT----AAGGAGTAAGGATCACCGAAGTAATGTCTAAACC

CAACGATTT-------CACAAAACAAAGATAAAGGATTCCGGAACAAGGAAACACTATTT

TCAA---TTGT------CTTAA----------------------CAATTAGATCAGAATG

AGGAAT--------------AAAA----ATAGATTCGAGACGAGACAAAC-AAAAGAGGT

TAGAGACGACTCAAGAAA-------TGAATG----TTTAAGGA---TTTCCCTTTGAACT

CTTTCGG----AATTATTCCACTTG-AGTTATGAGTACGAATGAGATTTCTTT----TTT

CCTGCAATGCAAGGAGGAACAAAAAAACCA--CTTTAATCATAGTCTAATTCATTAT---

-------------TTTGTGGATCCGTTTACCA-----TTATA------------------

------------------------------------------------------------

------------------TACAGAAATTAGAA----------------------------

-------------------TCTTTTCTTCTCGAGCCGTATGAGGAGAAAACCTCCTATAC

GTTTCTA-----AGGGGGGCATTGTTTATCTACATCTATCCCAATGGGCCATCTATCGAA

---TCGTTGCAATTGATGTTCGATCTCGAAGAGAAGGAAGAGATCTTCGAAAAGTGGGTT

TTTACGATCCGATAAAGAATCAAACTTATTCAAATGTTCCCGCCATTCTGTATTTCCTTG

AAAACGGCGCTCAACCTACTATAACTGTTCATGATATTTTAAGGAAGGCAGAATTGTTTA

AATTTAAAGAAGTAGCTTCGAGT-------------------------------------

TAAATGGATAAA------------------------------------------------

---------TCCAAGCAAC-----------------------------------------

------------------------------------------------------------

-------------------CTTTTCGTAAATCCAAGCTCTC-------------------

--TTTTCGCAGACGTTTGCC------------------------CCCAATCGGGTCGGGG

GATCGAATTGATTATAGAAACATGAGTTTAATTAGTCGATTTATTAGTGAACAAGGAAAA

ATCTTATCCAGGCGGGTAAATAGATTGACCTTGAAACAACAACGATTAATTACTATTGCT

ATAAAACAAGCTCGTATTTTATCTTTATTACCCTTT-----------CTTAAT------A

ATGAGAAACAATTTGAGAGAGCCGAGTTGATCCCTAGA-AGTACTGGTCCCAGAACCAGA

AATAAATAG---------------------------------------------------

----------------------------------------------------GTGACACG

TTC------------------------------------------------------ACT

AAAAAAAAATCCTTTTGTGGCTAATCATTTATCGAGAAAAATCGAAAAACTCAACATG--

----------------------CGGGAGGGGAAAGAAATAATAGTAACTTGGTCTAGGGC

ATCTACCATTATACCCACAATGATCGGCCATACAATCGGTATTCATACAGGAAAAGAACA

TTTACCTATTTATATAACAGATCGTATGGTGGGTCATAAATTGGGCGAATTCGCGCCTAC

TCTAACCTCCGCAAG------ACATGCAAAAAA---------------------------

------------------------------------CGATAATAAATCTCGTCGTTAAAT

GATT-------------------------------TTGAAATCGTT---TCTCCTAGGTA

A---------TCTATTATCCTTATGCA--------------TGAAGATAATAAATTCGGC

CATTGTGGTCGGGCTCTATTATGGATTTCTGACC------ACATTTTCCATAGGGCCC--

--TCTTATC--------TCTTCCTTCTCCGAGCTCGGGTTATGGAAGAAGGAACCGAGAA

GGAGGTATCAGCAACAACTGGTTTTATTACGGGACAGCTCGTGATGTTCATATCGATCTA

TTATGCGCCTCTGCATCTAGCATTGGGTAGACCTCATACAATAACTGTCCTAGTTCTACC

GTATCTTTT-------GTTTCAT------TTCTTCTGGAACAATCACAAAAAGATTTTTG

--ATTATGGATCTACTAGATCTAC---TACCAGAAATTCAATG---------CGTAATCT

CAGCA--------------------TTCCATGTGTA------------------------

------TTCCTGAATAATCTCATTTTTCAATTATTCAACCATTTCATTTTACCAAGCTCA

ACATTAACTAGATTAGTCAACATTTATATGTTTCGATGCAACAACAAGATGTTATTTGTA

ACAAGTAGTTTTGTTGGTTGGTTAATTGGTCACATTTTATTCATGAAATGGGTTGGA---

------------TTGGTATTATTCTGGATACGGGAAAATCATTCTATTCGATCTAA----

--------------TAAGTACCTTGTGGCAGAATTGAGAAATTCTATGGCTCG-------

--AATCTTTAGTATT-CTCTTATTTATCACCTGTGTCTA--CTATTTAGGCAGAATGCCG

TCGCCTATTTTCACT---------------------------------------------

------------------------AAGAAACTG------AAAGAAACCTCAGAAACAG--

----------------------------AAGAAGG-------------------------

---------------------------------------GGAAGAAGGTGAAGA------

---------------------------------AGAAAGCGATG------TAGAAGC---

---AA------------------------------CTTCCAAAACG------AAGGAGAC

T--------------AAACAG---GAACAAGGGGGATC--CACCG---------------

-AAGAAGACCCTTCCC---TTTGCT------CGGAAGAAAA------------GG-----

--AGGATCCGGAC---AAA---------------ATA-----------------------

----------------------GATAAAACGGAAG--AGATCCG---------GGTGAAT

---------GGAAA----------GGAAAAAACAAA------------------------

-----------------------------------------GGATGAATTCCACTTT---

---------------CACTTTAAAGAGAC--------------ATGTTAT-----AAAGA

TAGCCCAGT------TTAC---------GAAAATTAT------TATCTTGATGGGTATCA

AGATAA------TG--------GGAAATTGGGA------AGA----------------CT

TAAA--------AAAGAAAAAAACGAAAAGA------TCCATTTCCGCC-----------

TTGAAAAACCTCTTGT---------------------AACTTTTCTTTTCGA--------

-----------------TTATAAACGAT--GGAATCGTCCATTGCGATATATAAAAAA--

--------------------------CGGTCGA----------------------TTTGA

------AAA---TGCCGTACGAAATGAAATGTCACAA-----------------------

---------------------------------TATTTTTTTGATATATGTCCAAG----

---TGATGGAAAACAAA------------------TAATATCTTTTAC------------

--------------ATATCCGCCTAGCTTATCAAGCTTTTTG---------GAAATGAT-

-AGAAGGA----------------------------AAAATTTCTTTG------------

TACACAACCGAAAAACTATGCC-----ATGAGG----------------GC------CTG

TCCCA------TTATTGGG----------------TTTATACC--------AATGAACAA

AAAAAATATAACTTG-AAAAATGAATTGATAAATCGAA------------TAAAAGTTAT

AG---------AAAAAGAAAAGGGATCTCTTGTTCTAGATATACTTGAAAAAAGA-----

---------ATCAGATTATAC----------------AATGATGAGAATGAACGA--GAA

TGCTTGCCAAAAATCTATGATCC--TTTTTTCAGCGGGCCCTATCGCGGGACAATTAAAA

AATTCTACTC------------------AAACACAAATATAAACACAAATAG-------A

AATGA---------TTTTATCACTTT---------------------------TGTAGA-

--------------------------AGATTCCATCAAAATGTTTTGGATAAATAAAATT

CATAGTTTACTTTTTAATGA-----------TTATAACGATTCCCG------------AG

AATTTGA-------------------------------ACATCAAAAAAATCCGTTTAAT

TTTGATAGGGAATCATTATTTAGAAAATTTAAGTCTAATAATAAAATAATAACTTCTTCT

AATAATCCCGGCACC------TCAACGAATCAATTTTATTTAAAGTCCGAACCCCGTTTT

CATTTGGTAAAATCT---------TCTTTTTTAATAGAAACAGAACAAA-----------

----AACGGTTTGAAA-------GTCAAGCAAAATTTTTG----------------AAGT

TTGTATTCGA--------TATGATTAC-----------AACTGATCAAAATGATCAAAAT

AAATCAACAAAAAA-----------AAAATTAT------TGGAA-------T------A-

-----GAAGAAATCTGTAAAAA-------GCTTTCTCGA---------TGGTCATA----

-----------------CAAATTGA---CCGAT---GA--------GTTG----------

--GA--------------------------------GGAACAGGA--TCAAGAAAG----

------------------------------------------------------------

------------TG---AGGA-------AGAATCAACGGAAG---ATCCCGGGATTC---

-------------------------GTTCAAGAA------------------AAGCCAAA

CG------------------TGTGGTAATTTAT-------------------ACTGAAAA

TGATCAGA------------ATAC-------------------CGATTC------TCTT-

------------------------------------------------------------

------------------------------------------------------------

-----------------------------------ACT------AGTACTGATAATACTA

GTAAT-------------------------------------------------------

-----AGCGATCAA---------------GCAGAA------GAGGTGGCTTTGATACGTT

ACTCACAACAATCGGATTTTCGTCGAGATCTAATCAAAGGGTCCATGCGCGCTCAAAGAC

GCAAAACGGTTA---CTTCGGAAA-----------TGTTTC---AAACAAATGTGCATTC

CCC------GCTTTTTTTTGAT----------CGAATAGACAAAACATTTTTTTTT---T

CTTC---------TTTTGATA---T---------TTCTGAAAAAAAAAATCA------AA

TTTTGAGTAATTGGATTGGTAGAGAAG------CAGAATT---------ACAAATTTCT-

-----------GATCTTGAGGAAGAAGA-------------------GGCAAAAGAAA--

-------------ATGA-------------GAAAGATGAACAAA----------------

-----------------ATGA------ACGA---ATAACAATATCAGAAACTTGGGATAC

CATTATATTTGCTCAAGCAA-------------TAAGGGGTTCGATGTTAGTAACCCAAT

CGTTTCTTAGAAAATACATTGTATTACCTTTATTGATAATAGTGAAAAATGTTGGTCGTA

TGTTATTATTACAATTTCCCGAGTGGTACGAGGATTTGAGGGAATGGAATAGAGAAATTC

ACATTAAATGCACCCATAATGGTGTTCAATTATCTGAAACAGAATTCCCCAAAAATTGGT

TAACAGACGGGATTCAGATAAAGATTCTATTTCCCTTTTGTCTAAAACCTTGGCGAAAAT

CAAAATTAA--AAGTACAATCCGCTAATTCTAATAGAGATTCAACGAAC-----------

-AAAAAAGAAAATTTTT--------GTTTTTTAACAATATGGGGAATGGAAGCGGAACTT

CCCTTTGGT---TCTCCCCGAAAAC----GATCTTCG-----TTTTTTGAACCTATCGGT

AAAGAATTCAA------------ACGAAAAT------------TTAGAAAAGTGAAAAAG

ATAT------TTTTTCTAGTTTTAAGAATTTTAAAAGAAAG---------------AAGA

AAGGGGTCGCTGCAAATTTCAAAAGAAAAAAACAAAAAAACA---AGATGGGTCA-----

--------------------------------------TAAAAAGAGTTCTAG-----CT

ATAAAAC------GAATAATGAAAGAATTT------------------------------

---------------------GAAACAGTGAATCC---------ATTTGAGTCGAGGAAA

GC---CAAACTATAC---------GAACTAACTACAAATTTA-----AAATATTACAAAA

T------------ATATAATAAAA---------------TTAACC------AT-----GA

ATCGACCACTCTAATTCGATCTATGAATTGGATAAATTATCCACTCATAG----------

------------------AAAAAAAAA--------TTAAAAACCTTTCCGATAGGATAAT

CACAATCAGGAAT---------------------CAAA------TAAAA------GGAAT

TAC----AAA--------------AGAAAAGAAAA------AAAGAATTCTAAC------

---------------------------------------------TTATGATGTGAAAA-

-----GATTGGACTC-------GCATAAA---------TACATTTGGCGGAT--------

-ATTC---CAAA------------------------------------------------

----------------------------AAAACAA---------------TATTCGATTA

ATACGTAAA---TTTCATTA-----------------TTTTATGAAA-------------

---------TCTTTCGTTGAAAAAGTATACAAGGATA-----------------------

-------------------------------------TCTTCCTATGTACCATTAACA--

TTCTCAGGATTCATGGA------CAACT---------------TTTTTTTGAATCAACAA

AAAAA-------------------ATTATCAATAAAT---CCATTTAC-----------A

GCGATGAAACAAAT---------------CAACA--------------------------

-------------------------AGGAATTGATGAAACAAATCAAAATAC--------

------AA------TGAACTTTTTTTCGACT--------ATAAATAAGTCA---------

------------TTATTGAATA---------------------------------CTAAT

AATAATTTTAATCCTT---------------------------ATTATGACTTATCTTCC

CTGTCACAAACATATGTATTTTATAAATTATCACAAACCAAATTAGTAAATAAGTATCAT

TTGGGATCTGCACTTCCAAAT------TATGGAACCCATCATTGTCTAAGGGATAAAATA

AAG------------AATTATTGTATAACACGAGAAATATTTGATTACGGATCAAGGCAT

AAGAAAATTCGAAAGTCTGGAATG-AATGAATGGAAAAACTGGTTAAAGGGTCATTATCA

ATATAATCTATC-CCAGA--GTAACTGGTCCCAATTAGTACCGATAAAATGGCGAAATAG

AGTCAATCAACGTCGTACGA------TTCAAA------ATGAAGACCCAATGAAATTTGA

TTCATATA-AAAAGAAAAAGACCAATTAATTCATTACGTGAAAAAAAAAAAA------TT

CTTATTT------AGTGGATTTATTGA---------------------------CGAATC

AAAAAGAAAATCT----TAAAAAACACTACAGATATAATCTTTTATTACATAAATCTATG

---------------AATTATGGGGATAA---------------------GGAGGACTCA

TATATTTATGGACCAA----------------------CACTACAGGTAAAGATG-----

------------------------------------------------------------

------AACCGAGAAATTCCACA------TAATTTC-AAT---------ATACCGAAATC

CCACTCATTTG---ATGTATTGGTAAGTGCAGCCATTAGTGATTATCTAGAGGCGAAATA

T------GGTACGAATAAAA------ATCTGCA-----TAGAAA---ATATCT-TGATT-

GTAGAATTCTCCGTTTTT-G-----------TTTTAGACAGAATATTG------ATATTG

AGACTTGGACAAATACGCGTATAGATGCCAAGATTAATAATAATAAGATTAATAAAAATA

TTAAGTCGGAAACTAAGAA--GTATAAAAAAATGG-AAAAGAAA-----GGTATTC----

-----------CGACTTATCAAGAAATCAACA------CATCCTATAAAAAAA---AAAA

CTT------TTTTGATTGGATG---GGAATGAATCAAGAAAAGTTATATCG---TAATCG

TACTATAT-----------------CATATCTAAAATATCGGCTC-------------TT

ACCAGAACTTGTAATACT------------------CTTTGATATATATAGGATTAAACC

ATGTATTATACCAATACAATTACTTGTTTTCTATTTTTATA---------GAAATGAAA-

-----------------------------ATATTAGTCAAAATAAAAGCA----------

------TCAACATAAATAA-------------------AAAAAAGAA------TCCTCAT

ATATCATATAATCAA------AA--AAAATATCTTGCATTAAGAAA---------TTCTA

ACCAAGAAAA-------------------------ACATCAACAACAG---GACCAAA--

--GAGGCC---T--------------------TGGGCAAGAT------------TTGCGA

AAA--------------------AAAAAAAAGATGTTC----------------------

-----------------AAGAAAA---------TTATTCGCAATCAAACATTAAAAAACG

TGGAAAGAAAAAAAAAC----------------CAAGAA-AAATAAAGAAGCAGAACTAG

ATTTCTTCCTGAAA-----AAATAT----------TTCCT--------TTTTC-------

---AATTAAGATG-------------GA---------ATGACCCCTTGAAT---------

-CAAACAA----------TGATCAA-----------------------------------

-TAATATCAAGGTCTATTGTCTCCTACTTAG-------ACTGACA--AATCC-------A

AAGGAAATTTCTA-------------------------------TATC----TTCGATTC

AAAGA-----------------GGAGAGATGCATCTAGATGTAATGCT------GAT---

---TCAGAAAGATCTCGCTCTTACAG----------------------------------

AATTGATA--------AAAAAAGGAAT------ATTG-----------------------

------ATTAT------CGAACCCATTCGTCCATCCATAAAAAGGGA-------------

-------------------------------------------------TAGACAATTTT

TGATATATAAAACCATAAGTATTTCATTG-------GTTGATACAA--------------

-GTAAAGATCAAACCAAAACTAATAAAAAATACGCAAAAAATTTAT--------------

-----ATGTTGATAAGAAGAAGAATTTTGACAGAGCCATTGTACAACATAGCAATACACT

TGT---GAAT-ATAGAACAA---------------------------------AGCCATT

ATGATTTCC------------TTGTTCCAGAAAATATTCTACCTCCAAAACGTCGGAGAG

AATTGGGAATTTTAATTTGTTTTAATTCCGGAAATTCCGGAAATGTT------GTGGATA

AAAATCCAATATTTTGTAACGAAAACAAGATAA------AAAATTGTCGAC-----AATT

TTGGAATGA-----------GGACAAGCATCT------TAATGCGAACGC------AAAC

------AAATTTATAAAGTTC------------------AAATTGTTTCTTTGGCCCAAT

TATCGATTAGAAGATTTAGCTTGTATGAATCGCTATTGGTTTAATACCAATAACGGTAGC

CGTTTCAGCATGTTAAGGATACATATGTATCC-ACAATTCAGAATTAGTTAA--------

-------------------------------------------------ATGAAAGGACA

TCAAT------------------------TCAA---------AT----------------

------------------------------------------------------------

-CCTGGATCTTCGAATTGAGAGAGATATTGAGAGAGATCAAGAATTCTCACTATTTCTTA

GATTCATGGATCAAATTCGATTCAGTGGGATCTTTCACTCACATTTTTTTCCACCAAGAA

CGTTTTATGAAACTCTTTGACCCCCGAATTTGGAGTATCCTACTTTCACGTGATTCAC--

----AGGGTTCAAGAAGCAATCGATATTTCACGATCAAAGGTGTAGTACTGCTTGTAGTA

GCGGTCCTTATATCTCGTATTAACAATCGA---AAGATGGTTGAAAGAAAAAATCTCTAT

TTGATGGGGC---------------TTCTTCC---TATACCTATGAATTCCATTGGACCC

AGAAAT------------GGGACATTGGAAGAATCCTTTTGGTCTTCCAATATCAATAGG

TTGATTGTTTCGC------TCCTGTATCTTCCAAAAGGGAAAAACATTTCTGAGAGTTGT

TTCATGGATCCGCAAGAGAGCACTTGGGTTCTCCCAATAAATA---------AAAAGTGT

ATCATGCCTGAATCTAACC------------GGGGTTCGCGGTGGTGGAGGAACCGGATC

GGAAAAAAGAGGGATTCTAGTTGTAAGGTATCTAATGAAACCGTAGCTGGAATTGAGATC

TCATTCAAAGAGAAAGATAGCAAATATCTGGAGTTTCTTTTTTTATCCT------ATACG

GATGATCCGACCCACAAGGACCGTGATTGGGAAT---TGTTTGATCGTCTTTCTCCGAGG

ACGAAGCGAAACATAATCAACTTGAATTCGGGACAGCTATTCGAAGTCTTAGGGAAAGAC

TTGATTTGTTATCTCATGTCTGCTTTTTGCGAAAAAAGACCAATTGAGGGGTGGGATTTC

TTCAAGAAACAAGGAGCTGAGGCAACTATTCAATCCAATGATATTGGGCGTGTTTCCCA-

-----TC-----------------------------------------------------

------------------------------------------------------------

-TCTTCTCG------AGAAACAAGTGGGGTATTTCTTTGCAAAATTGTGCTCAATATCAT

ATGTGGCAATTCCGCCAGGATCTCTTCGTTAGTTGGGGAAAGAATCAGCACGAATCGGAT

TTGTTGAGGAACGTATCGAGAGAGAATTGGATTTGGTTAGAC---AATGTGTGGTTGGTA

AACAAGGATCGGTTTTTTAGCAAGGTACGGAATGTATTGTCAAATATTCAATATGATTCC

ACAAGATCCATTTTCGTTCAAGTAACGGATTCTAGCCAATTGAAAGGA---TCTTCTGAT

CAATCCAGAGATCATTTCGATTCCATTAGAAATGAGGATCCCGAATATCACACACTGATC

GATCAAAGAG--------------------------------------------------

------------------------------------------------------------

------------------------------------------------------------

-------AGATTCAGCAAC---------TAAAAGAAAGATCGATTCTTTGGGATC---CT

TCCTTTCTTCCAACGGAAGGAAC------AGAGATAGAATCAGATCGATTCCCGAAATGC

CTTTTTGGATCTTCCCCAATGTCCCCG---------CTATTCACGGAAGGTGAGAAGCAG

ATGAATAATCATCTGCTTCCG------GAAGAAATCGAAGAATTTCTTGGGAATCCTGCA

AGATCAATTCGTTCTTTTTTCTCTGACAGATGGTCA------GAACTTCATCTG---GGT

TCGAATCCTATTGAGGGGTCCACTAGGGATCAGAA------------ATTGTTGAAGAAA

AAACAAGATC---TTTCTTTTGTTCCTTCCAGGCGATCGGA------------AAATAAA

GAAATGGTTGATATATTCAAGATAATTACGTATT------TACAAAATACCGTCTCAATT

CATCCT-----ATTTCCTCAGATCCGGGATCTGATATG---GTTTCGAAGGATGAACCAG

AT---ATGGACAGTTCCAATAAGATTTCATTCTTGAACAAAAATCCATTTTTTGATTTCT

TTCATCTATTCCATGACCGGAACAAAGGGGGATACACGTTACACCATGA---TTTTGACT

CAGAAGAGAGATTTCCAGAAATGGCAGATCTATTCATTCTATCAATAACCGGGCCGGATC

TGGTGTATCATAGGGGATTTTTCTTTTCTATTGAT---------------TCCTACGGGT

TAGATCAAAAAAAATTCTCGA------ATAAGGTATTCAACCCCAGAGATGAATCGAAAA

AGAAATCTTTATTGATTCTACCTCCTCTTTTTTATGAAGAGAATGAATCTTTTTATCGAA

GGATCAG-AAAAGAGTCGGTCCGGATCTACTGTGGGAATGATTTGGAAGATCCAAAACGA

AAAACAGCGGTATTTGCTAGCAACAACATAATGGAGGCAGTCAATCAATATAGATTGATC

CGAAATCTAATTCAAATCCAATATAGCACCTATGGGTACATAAGAAATGTATCGAATCGA

TTCTTTTTAATGAATAGATCCGATCGCAACCTCGAATATGGAATTCAAAGGGATCAACAA

ATAGGAAATGAT----ACTCTGAATCATATAACTATAATGAAATATAGGATCAACCAACA

TTTATCGAATTTGAAAAAGAGTCAGAAGAAATGGTTTGATCCTCTT----ATTTCTCGAA

CCGAGAGATCCATGAATCGGGATCCTAATGCATATAGATACGAATGGTCCAATGGGAGCA

AGAATCTCCAGGAACATTTGGAACATTTCATTTCTGAACAGAAGAACCGTTTT-------

--CAAGTAGTGTTCGATC------GATTACG-----------------TATTAATCAATA

TTCGATTGATT-GGTCCGAGGCTATCGATAAAGAAGATTTGTCCAAGTCACTTC---TCT

TT--TTGTCCAAGTCA--CTT------------------------CTCTTTTTGTC----

---------------CAAGTCACTTC---CCTTTTTGTCCAAGTTACTTCCCCTTTTCTT

TGTGAGTATCGGGAATATCCCCATTCATAGGTCCGAGATCCACATCTATGAATTGAAAGG

TCCG------CAA---CTCT---------------------GCAATGAGTTGTTAGAATC

AATAGGTGTTCAAATCGTTCATTTGAATAAATTGAAACCC------TTCTTATTGGATGA

TCATGATA--CTTCCCAAAGACCGAAATTCTT---------AGGAATAATATTACCATTT

TTGTTCAAA-AAGATACCAA------AGTG-GATGATTGACTCATTCCATACTAGAAATA

ATCGCAGGAAATCCTTTG------CAGATTCCTATTTCTCAATAATATCCCACGATCGAG

ACAATTGGCTGAATCCC-GTGAAACCATTTCATAGAAGTTCATTGATATCTTCTTTTTAT

AAAGCAAATCGGCTTCGATTCTTGAATGATCCACATCACTTCTGGTTCTATTGTAACAAA

AGATTTCCTTTTTATGT---GAAAAAGACCCGTATCAATAATTATGATCTTACATATGGG

CAATTCCTCAATATCTTGTTCATTCGCA-ACAAAATATTTTCTTTGTGCGTC------GA

TAAAAAAAAACATATTTTTTTGGAGAGAGAGACTATTTCA------CCAATTGAGTCACG

GGTATCTGACATATTCATACCTAACGATTTTC------CACAAAGTGGTAACGAAACG--

----------TATAACTT---------GTACAAATCTTTCCATTTTCCAATTCGATCCGA

TCCATTCGTTCGT------AGAGCTATTTACTCGATCGCAGACATTTCTGCAACACCTCT

AACAGAGGAACAAATAGTCAA---------AACTTATTGTCAGCCTCTTTCAGATATGAA

TCTATCTGATTCAGAAGGGAAGAACTTGTATCAGTATCTCG------GTTTCCATTCAAA

CATGAGTTTGATTCACACTCCATGTTCTGAGAAAGATTTTCCATCCGGAA----------

------------------------------------------------------------

----------------------AGAGGAAAAAACGGAGTC--------------------

------TTTGTCTAAAGA------------------------------------------

--------AATGCGTTGAGAAACGGCAGATGTATAGAGCCTTTCAACGA---GATAGTGC

TTTTTTAAATCTCCCAAAATGGGATCTGTTCCAAACATATATGCCATGGTTCCTGACTTC

GACAGGGTACAAATATCTAAATTTCACCCTTTTAGATACTTTTTCAGACCCATTGCCGAT

ACTAAGTAGCAGTCCAAAATTTGTATCCATTTTTCATGATATTATGCA------------

TGGATCAGATATATCATGGCCAATTCCTCAGAA------------GATTCTTCCGCAAT-

----------GGACTCTGATAAGTGA----------------------------GATTTC

GAGTAAGTG------TTTACAGAA---TCTT--------CTTCTGTCCG-AAGAAATGAT

TCATCGAAATAATGAGTCACCCGCTCCATTGATATGGACACATCTGAGATCAAAAA--AT

GCTCGGGAGTTCCTCTATTCAATCCTTTTCCTTCTTCTTGTTGCTGGATATCTCATTCGT

ATACATCTTCTCTCTGTTTCCCGAGCCTCCAGTGAGTTACAGACAGAGTTAGAAAAGATC

AA----GTCTTTGAT---------GATTCCATCATAC-ATGATTG---------------

---------------------------AGTTG----------------------------

-------------------CAAAAACTTCTGGATAGGTATCCTACATCTGAACTGAAT--

------------------------------------------------------------

----TCTTT--CTGGTTAAAGAATCTCTTTCTAGTTGCTCTGG-----------------

--------AACAATTAGGA-------------------------GATTCTCTGGAAGAAA

TATGGGGTTCTGCTTCTGGTGGCAACATGCTATGGGGTGGGGGT----------------

----TCCGCTTATGGGGTCAAATCAATGCGTTCTAAGAAGAAATATTTGAATATCAATC-

--------TCATCGATCTCATAAGTATCATACCAAATCCCATCAATCGAATCACTTTTT-

-------------CGAGAAACACGAGACATCTAAGTCGTA--------------------

------------------------------------------------------------

CAAG------------------------------TAAAGGGATCTA------TTCATTGA

TAAGAAAAAGAAAAAA--CGTGAACGGTGATTGGATTGATGATAGAAT------------

-------------------------AGAATCCTGGGTCGCGAACAATGATTCGA------

---------------TTGAT----------------------------------------

-----GATGAAGAAAGAGAATTCTTGGTTCAGTTCTCCACCTTAACGACAGAAAAAAGGA

TTGATCAAATTCTATTGAGTCTGACCCATAGTGATCATTTATCAAAAAATGACTCTGGTT

ATCAAATGATTGAACAACCGGGATCAATTTACTTACGATACTTAGTTGACATTCATAAAA

AGTATCTAATGAATTATGAGTTCAATAGATCCTGTTTAGCAGAAAGGCGGATATTCCTTG

CTCATTATCAGACAATCACTTATTC------ACAAACCCCTTGTGGGGCTAATAGTTTTC

ATTTCCCAT---CTCATGGAAAACCCTTTTCGCTCCGCTTAGCCCTATCCCCTTCTAGGG

GTATTTTAGTGATAGGTTCTATAGGAACTGGACGATCCTATTTGGTCAAATACCTAGCGA

CAAACTCCTATGTTCCTTTCATTGCGGTATTTCCAAACAAGTTCCTGGATGACAAGTCTA

AAGATTATCTTAT------------------------TGACCATCTTGATGATAGTGACG

A------------------TGACGATGTCGATATTGATGATAGTGACGATATTGATGATG

ACCTTGATAC------GGAGCTGTTAACTATGACGAATGTGCTAACTATGTATATGATGA

TGCCGAA---AATAGACCGATTTGAATTTGATATCACCCTTCAATTCGAATTAGCAAAAG

CAATGTCTCCTTGCATAATATGGATTCCAAACATT----------CATGATCTGT-----

------------------------------------------------------------

---------------ATGTCAATGA---------------------------GTCGAATT

ACTTATCCC-----------TCGGTCTATTAGAGAACTATCTCTCCG---------GGG-

------ATTGTGAAAGATGTTCCACT------AGAAATATTCTTGTTATTGCTTCGACTC

ATATTCCCCAAAAAGTGGATCCCGCTCTAATAGCTCCGAATAAATTAAATACATGCATTA

AGATACGAAGGCTTCTTATTCCACAACAACGAAAGCACTTTTTCATTCTTTCATATACTA

GGGGATTTCACTTGGAAAAGAAAATGTTCCATACTAAGGGATTCGGGTCCATAACCATGG

GTTCCAATGCACGAGATCTTGTAGCACTTATCAATGAGGCCCTATCAATTAGTATTACAC

AGAAGAAATCAATTATAGAAACTAATACAATTAGATCAGCTCTTCATAGACAAACTTGGG

ATTTGCGATCC---------CAGG---TAAGACCGGTTC---AGGATCATGAGATCCTTT

TCTATCAGATAGGAAGGGCTGTTGCACAAAATGTACTTCTAAGTAATTGCCCCATAGATC

CCATATCTATCTATATGAAGAAGAAATCATGTAAGGAAGGGGATTCTTATTTGTACAAAT

GGTACTTCGAACTT---------GGAACGAGCATGAAGAAATTAACGATACTTCTTTATC

TTTTGAGTTGTTCTGCCGGATCGGTCGCTCAAGGTCTTTGGT------CTTCACCCGGGC

CCGATGAAAAAAATGGGATC---------AGCTTCGTTGAGAATGATTCTGATCTAGTTC

ATGGTCTA---------------TTAGAAGTAGAAGGC------GCTCTGGTGGGATCCT

CAC------GGACAGAAAAAGATTGCAGTCAGTT---T------------------GATA

ATAATCGAGTG---ACATTACTTCTTCGGCCCGAACCAAGGAATCAGTTAGATATGATGC

GGAATGGATTTTGTTCTATCGTTGATCAGAGATTTCTATATGAAAAAGACGAATCGGAGT

TTGAAGAAGCGGAAGGAGAAGGAGCCCTCGACCCGCAACAG------------------A

TAGAGGAGGATTTATTCAATCACATAGTTTGGGCTCCTAGAATATGGCGC---------C

CTTGTGGCAATCTATTTGATTGT------ATCGAAAGGCCCACTGAATTGGGATTTCCCT

ATTGGGCCGGGTCATTTCGGGGCAAGCGGATCGTTTATCATAAAG---------------

------------------------------------------------------------

------------AGGATGAGCTTCAAGAGA----------------------ATGATTCG

GAGTTCTTGCAGAGT----------------GGAA----CCACGGAGTACCAGACACGAG

ATAGATC------------TTCCAA---AGAACAAGGCT------TTTTTCGAATAAGCC

AATTCATTTGGGACC------CTGCGGATCCATTCTTTTTCCTATTCAAAGATCCGCCCC

TTGTCTCTG---------TGTTTTCCCGTCGAGAATTCT------TTGCAGATGAAGAGA

TGTCAAAGGGGCTTATTAC-----------------------------------------

----------TTCCC---------------------------------------------

------------------------------------------------------------

------------------------------------------------------------

------------------------------------------------------------

------------AAACAGATCCTCCTACATCTATATATAAACACTGGTTCATCAAGAATA

CGCAAGAAAAG------CACTTCGAATTGTTGATTCATCGCC---AGAGATGGCTTAGA-

-----ACCAATAGTTCATTATCTAATGGATCTTTCCGTTCTAATACTCTATCCGAGAGTT

ATCAGTATTTATCAAATCTGTTCCTATC---TAACGGAACGCTATTGGATCAAATGACAA

AGACATTGTTGAGAAAGAGATGGCTTTTCCCGGATGAAATGAAACGTTTGATTCATGTAA

CAGGAGAAAGGTTTCTTATTAC----TTAG------------------------------

--------ATGCCTAGATCTCAGAGAAATGGAAATTTTATTGATAAGACCTCTTCAATTG

TAGCCAATATCTTATTACGAATAATTCCGACAACTTCCGGGGAAAAAAGGGCATTTACCT

ATTACAGAGATGGTGCGATTTGAT----T---------------TTCTTTTTAG-----C

CC--------------------------CTAGT---------------------GGGTTT

G------GATAG----AA-AGAAAGAAATTATG-GAAA----TAAACCTACGCCTGGTGA

AGGTGAAGT-----TTGGAGATAGAACAATTCCTTCCGTCGTGTATTCTCGATTGATGCA

GCCTCATATGCTTTCAT----TATCGATTTGAGTATTGAGCGAAAGGTTACACCTATA--

----GGT----TCTGG-ATAG-----CGCGTCAATCCTACCCCA-T----TAGTACCAAT

AGGT-AGCATAG----GGGA-AGAAGCACTACTCC------T-----AGAAATCAACAAC

GCGAAAACTTTGTT-----TGTTATAAATTACCCCTTT--CCTTATCGGTATCGGGACTA

CCAA-------------GAATGGTTGGGACAACAAACATCCATCTCGTTCGTACTTCGGA

TATCCATAT-----AACCATCAAAGATTGTTGAAGTGGCTAATTC--CT-GGAAATAGGA

GGCGTTGAGAACAAAGAAATTGTTGG----AGTTACCCCTTTTATCT----AGTATTAAT

ACGATTGGT--GT------------------TAAGAAAAAACCTCTTTCGGGAAGGCTGG

CTAGAGATTTTTAGTAAAAA-TACTAGCCCCTGTCAGTTCATA-------ATGAAAATAG

TGAAAT--------TTGGATTCTATA-------------------------GATTATTTC

CCTTAGTAC---TATGCACAGAA-GGGAGGAGCCGTATGAGATGAAAATCTCACGTACGG

TTCTGGAGCGGAGATTCTTTGA-ATAGAAA------GAACGACCGTAACGGATGTCGGCT

CAGTCCGAAGGAAATTATGCAGAAGCTTTACAGAACTATTATGAAGCTACGCGACTAGAA

ATTGATCCCTATGATCGAAGTTATATACTCTATAACATAGGTCTTATACACACAAGCAAC

GGGGAGCATACGAAGGCTTTGGAATATTATTTCCGAGCACTAGAACGAAATCCATTCTTA

CCACAAGCTTTTAATAATATGGCCGTGATCTGTCATTACGTGCGACTATCTCCATT----

--ATAG------------------------------------------------------

-------------------AAAG-------AA-GAAAAAA-AGAGCAAATCCGCTAGTA-

-----------------AAT-ACTGG-------A----------------AAAATGGGCT

TTCTA-CATATGCATCGTCGAAA--GCAACGATTTT----TATCAGCTGTAGCGAGAAAA

GAAA------CTTCATGAGAACC-AAAATATGA--AGAAATGGGTAC-GCCCA-----TA

TACTCTATGGATAAAAAAAAG--GATCGAATTGATAGAGAAAGCACCGTAAAGATCAATT

AACGAGCTA---TT-GGATCGATACAGTT-----AAGAACTGCTTACT----TATGTCAT

GA-TATGATATA---AAAGTTAGG----AATCCACTTATGTAATAGAGTTGATCCAC---

----TAAGGTATTAAGCAGCGGTGTAGCATCAGATCCCAAAGAT-AGTAAGTTCTTT-TT

TCTTGTGGAGGTACGGAG---------------GAAAGTCTTTTTCAAAAGAGTCTAT-A

TGAA----------------------------AT---------GAAAACGAGATAGTTAC

CT-TTCAGAAA-------ATTCTAACGATGTAG------GGGA-----TAT------TCC

TCATTTTTCTAAAGGTGGGAGAAAAGATAGAACTGA-TTATTGATC-----ACAA-TTAG

AGTTTAAAACTTAATGTAATTAAC-----TTCTTCTGGTTA-AC--------CCAAG-AA

AAACGGGATAGGC------TTATGAGAGATCTAATTCAG-TTA----GGATATGATAGAT

TA--ATAAGATGGAACGCAAGCAAAAAGAATCGACTGCTGAGCCGTATGAGGTAGGAAAC

TCTCAAGTACGGTTCTAAGGGAAGGAATTGATCT-------ACCTATTCCGACCGGGGAG

AACAGGCCATTCTACAGGGTGACTCTGAAATTGCGGAGGCTTGGTTCGATCAAGCCGCTG

AGTATTGGAAACAAGCGATAGCGCTGACTCCGGGTAATTATATTGAAGCACATAATTGGT

TGAAGATCACGAGACGTTTCGAATTCAAATAAATGAACTGGCGATCAGAACATATATGGA

TAGAACTTATAACCGGGTCTCGAAAAACAGGTAATTTTTGTTGGGCCTGTATCCTTTTTT

TAGGTTCATTAGGGTTCTTAGTGGTTGGAACTTCCAGTTATCTTGGTAAAAATCTAATAT

CCGTATTTCCATCTCAGCAAATCATTTTTTTTCCACAAGGGATCGTGATGTCTTTCTATG

GGATCGCAGGTTTATTCATTAGCTCCTATTTGTGGTGCACAATTTCGTGGAATGTGGGTA

GTGGTTATGACCGATTTGATAGAAAAGAAGGAATAGTTTGTATTTTTCGTTGGGGATTTC

CTGGAATAAATCGTCGCATTTTCCTCCGTTTCCTTATGAAAGATATCAAATCCATCAGAA

TGGAGGTTAAAGAGGGTCTTTATCCT---CGTCGTGTCCTTTATATGGAAATAAGAGGCC

AAGGAGCCATTCCCTTAACCCATACTGATAAGAA---TTTTACTCCACGAGAAATGGAAC

AAAAAGCCGCCGAATTGGCTTATTTCTTGCGAGTACCAATTGAAG------TATTTTGA-

------------------------------------------------------------

------------------------------------------------------------

--------------------------------

>Amborella

ATGGAAAAA---TGTGGGTTCAATTCGATGTTGTCTAATGAAGAGTTAGAGCACAAGTGT

GAACTAAGTAAATCGATGGACAGTCTTGGTCTTATTGGAAATACCGGTGCAGGTGGAGGC

CCTACTCTAA--------------------------------------------------

----------------------------TATT---------CGATGTCAGGGACATTTTG

AGTTTGATCTATGATGACACTTTTAAGCTTAAGGATAGGAATGGTGACAGCTATTCCGTA

TATTTTGATATTGACAATAAGATCTTTGAGATTGACGATGATAGTGACTCTCTGAGTGGA

CTAGATATTGAAAATCAGAATCAGATCTTCTTTGATATTTACAATGATAGTGACTCTCTG

AGTGAACTAGATAGTGAAAATAAGAACTTTGAGATTCACAATGAGAGTGACTTTATGAGT

AAACTAGAAAGTTCTTTTTTTAGTTATCTCACTTATAGTTGTCTCAAACGAAGAAGTTCT

TTTTCTAGTTATCCTACTTCTCTCACTTCTAGTAATCTCAATAGTGGATCTAAGAGTTAC

AACCCCTACTA---TGATCCTTACATGCATGACACTAGATATAGTTGGAATAATGACATT

AACATTAATAGTTGCATTGACAGTTATATTCGTTGTGAAATC---------------GAT

TCCATTTCAAGTGGTAGCGACAATTGCAGTGACAG------TTATATTTATAGTTACAT-

-----------------------------CTGTAGTGAGGGGGTAAGTTATAGTGACAAT

G---------------GGAGTTCTAGTATAAGAACTAGAACTAGTACTAGTAGTGGCAGT

TCTTACCATATAAGAGGAAGATCTAATAATTTC------GAGAAAAATAAAAAATTAAAA

AAATTATGGGTTCAATGCGAGAATTGTTATGCATTGAATTATAATAAATTATTTAGGTCT

AAAATGAATGTTTGTGAACAATGTGGATATCACTTGAAAATGAGTAGTTCAGATAGAATC

GAACTCTCGATTGATCCAGGCACTTGGCATCCTATGGATGAAGATATGGTCTCC------

------------------ACGGATCCTATTGAATTTC-----------------------

-------------ATTCGGAGGAGGAACCTTATAGAGATCGCATCGATTCTTATCAAAGA

CAAGCAGGTTTAACCGATGCTGTTCAAACAGGCATAGGTCAACTAGAAGGTATTCCGATA

GCAATTGGAGTTATGGATTTTCAGTTCATGGGAGGTAGTATGGGATCCGTAGTAGGCGAG

AAAATAACTCGTTTGATCGAGTATGCTATTGATAGATCTCTCCCAGTTGTTATTGTGTGT

GCTTCTGGAGGAGCACGCATGCAAGAAGGAGGTTTGAGCTTGATGCAAATGGCTAAAATA

TCTTCCGCTTCATATAATTATCAATCCAATAAAAAGTTATTCTATGTATCAATCCTTACA

TCTCCTACAACCGGTGGAGTGACTGCGAGTTTCGGTATGTTGGGAGATATCATTATTGCC

GAACCCAATGCCTACGTAGCATTTGCAGGCAAAAGAGTAATTGAAGAAACATTAAATAAG

AAAATACCTGATGGTTTGCAAGTAGCTGAGTATTCATTCCATAAGGGCTTATTCGATTCA

ATCGTACCACGTAATCCTTTAAAAGGTGTTCCAAGTGAGTTATTTCAGCTCCACGGTTTC

TTTTCTTCTACTTTCCATCCCCTT------------------------------------

------------------------------------------------------------

------------------AAATCAAATAAAGTAAAGCGCTAA------------------

-ATGGTAACCATTCGAGCTGACGAAATTAGTAATATTATCCGTGAACGGATTGAGCAATA

TAATAGAGAAGTAAAGATTGTCAATACTGGCACTGTACTTCAAGTGGGCGACGGCATTGC

TCGTATTCATGGTCTTGATGAAGTAATGGCGGGCGAATTAGTAGAATTTGAAGAGAGTAC

AATAGGCATTGCTCCAAATTTGGAATCCAATAATGTTGGTGTTGTATTAATGGGTGATGG

TTTAATGATACAAGAGGGCAGTTCTGTAAAAGCAACAGGAAGAATTGCTCAGATACCAGT

GAGTGAGGCTTATTTAGGTCGTGTTATAAATGCTCTGGCTAAACCTATTGATGGGAGAGG

TGAAATTTCATCTTCCGAATCTCGGTTAATTGAATCCCCCGCACCAGGTATTATTTCGAG

ACGTTCCGTATATGAACCTCTTCAAACAGGTCTTATTGCTATTGATTCCATGATTCCTAT

AGGACGCGGTCAGCGAGAATTAATTATTGGGGACAGACAGACCGGTAAAACAGCCGTAGC

TACAGATACTATTCTGAATCAAAAGGGGCAAAATGTAATATGTGTTTATGTAGCTATTGG

TCAAAAAGCATCTTCCGTGGCTCAGGTAGTGACTACTTTCCAGGAACAAGGAGCAATGGA

ATACACTATTGTGGTAGCTGAAACGGCGGATTCACCTGCTACATTACAATACCTCGCTCC

TTATACAGGAGCAGCCTTGGCTGAATATTTTATGTACCGTGAACGACATACTTTAATAAT

TCATGATGATCCCTCTAAACAAGCACAAGCTTATCGCCAAATGTCTCTTCTATTAAGAAG

ACCGCCTGGTCGCGAAGCTTATCCAGGAGATGTTTTTTATTTGCATTCCCGACTTTTGGA

AAGAGCCGCTAAATCAAGTTCTCGTTTAGGCGAAGGAAGTATGACTGCTTTACCAATAGT

TGAGACTCAATCTGGGGACGTTTCGGCTTATATTCCCACTAATGTAATTTCCATTACAGA

TGGACAAATCTTCTTATCTGCCGATCTATTCAATGCTGGAATCCGGCCTGCTATTAATGT

GGGTATTTCCGTTTCCAGAGTAGGATCCGCAGCTCAAATTAAAGCTATGAAACAAGTAGC

TGGCAAATCAAAATTGGAACTGGCTCAATTTGCAGAGTTAGAAGCCTTTGCACAATTCGC

TTCTGATCTCGATAAAGCTACTCAGAATCAATTGGCAAGAGGTCAACGATTGCGCGAGTT

GCTCAAACAATCCCAAGCAGCCCCTCTCGCGGTGGAAGAACAGGTAGTTACTATTTATAC

AGGAGCGAATGGGTATCTTGATCCATTAGAAATTGGACAGGTGAAGAAATTTCTCGTTCA

GTTACGTACTTATTTAAGAACAAATAAGCCTCAGTTCCAAGAAATCATATCTTCTACCCG

GACGTTCACCGAGGAAGCAGAAGCCATTCTGAAAGAAGCTATTCAGGAACAGATAGAACT

GTTTCTATTTCAGGAACAAACA---------------------TAAATGAGAATCAATCC

TACTACTTCTGGTTCTGGTT------CCGGGGCTTCTACACTTGCGGAAAAAAGCGTGGG

ACGTATCGCTCAAATCATTGGTCCGGTACTGGATGTAGCTTTTCCCCCGGGCAAGATGCC

CAATATTTACAACTCTTTGGTAGTTAAGGGTCGAGATACTGCCGGTCAGCAAATTAATGT

GACTTGTGAGGTACAACAATTATTAGGAAATAATCGAGTTAGAACGGTAGCTATGAGTGC

TACAGATGGTCTAATGAGAGGAATGGAAGTGATTGACACGGGAGCTCCTCTAAGTGTTCC

GGTTGGCGAAGCTACTCTCGGACGAATTTTCAACGTTCTTGGAGAACCAGTTGATAATTT

AGGTCCTGTAGATACTCGCACAACATCTCCTATTCATAGATCTGCGCCTGCCTTTATACA

GTTAGATACCAAATTATCAATCTTTGAAACAGGCATTAAAGTGGTGGATCTTTTAGCTCC

TTATCGTCGTGGAGGAAAAATCGGGCTATTCGGGGGAGCCGGAGTGGGTAAAACAGTACT

CATCATGGAATTGATCAATAACATTGCCAAAGCTCACGGAGGTGTATCTGTATTTGGCGG

AGTAGGAGAACGTACTCGTGAAGGAAATGATCTTTACATGGAAATGAAAGAATCCGGAGT

GATTAATGAACAAAATATTGCAGAATCAAAAGTCGCTCTAGTCTATGGTCAGATGAATGA

ACCGCCGGGAGCTCGGATGAGAGTTGGTTTAACTGCCCTAACAATGGCGGAATATTTCCG

TGATGTTAATGAGCAAGACGTACTTCTATTTATTGACAATATCTCTCGCTTCGTTCAAGC

AGGATCAGAAGTCTCTGCCTTATTAGGCAGAATGCCTTCTGCCGTGGGTTATCAACCTAC

CCTTAGTACAGAAATGGGTTCT---TTGCAAGAAAGAATTACTTCTACTAAAGAGGGATC

CATAACTTCCATTCAAGCAGTTTATGTACCTGCAGACGATTTGACCGACCCTGCTCCTGC

CACGACATTTGCACATTTAGATGCTACTACCGTACTATCAAGAGGATTAGCCGCCAAAGG

TATCTATCCAGCAGTAGATCCTTTAGATTCAACGTCAACTATGCTCCAACCTCGGATCGT

TGGCGAGGAACATTACGAAACTGCGCAAAGAGTTAAGCAAACTTTACAACGTTACAAAGA

ACTTCAAGACATTATAGCTATTCTTGGACCAGATGAATTATCCGAAGAGGATCGTTTAAC

CGTAGCAAGAGCACGAAAAATTGAGCGTTTCTTATCACAACCCTTCTTCGTAGCAGAGGT

ATTTACTGGTTCTCCAGGAAAATATGTTGGTCTCGCAGAAACAATTAGGGGGTTTCAACT

GATCCTTTCCGGAGAATTAGATGGTCTTCCCGAACAGGCTTTTTATCTGGTAGGTAACAT

CGATGAAGCTACCGCGAAGGCTATTAACTTAGAAGTAGAGAGCAAATTAAAGAAATGAAT

GACCCTAAATCTTTGTGTACTGACTCCTAATCGAATCATTTGGGATTCAGAAGTGAAAGA

AATTATTTTATCCACTAATAGTGGCCAAATTGGTGTATTACCAAATCACGCACCTATTGC

AACGGCTGTAGATATAGGTATTCTCAAAATACGCCTC---CAGGAGCAATGGTTAACAAT

GGCTCTGATGGGTGGTTTCGCCAGAATAGGCAATAATGAAATAACCATATTAGTAAATGA

TGCGGAGAAGGGTAGTGACATTGATCCGCAAGAAGCTCAGCAAACTCTTGAAATAGCTGA

AGCTAACCTGAGTAGAGCTGAGGGCAAGAGACAAACAATTGAGGCGAATTTAGCTCTTAG

ACGAGCTAGGACACGAGTAGAGGCTATCAATG---------CTATT------------TC

GTCCTAGATGAAAAATGTAACCGACTCTTTCGTTTCCTTG---GGCCACTGGCCATCTGC

CGGAAGTTTCGGATTTAATACCGATATTTTTGCAACAAATCCAATAAATCTAAGTGTGGT

GCTTGGTGTGTTGATTTTTTTCGGAAAGGGAGTGTGTGCGAGTTGTTTATTTCAAGAATA

-GGTTGGATCCTACCAGCTGCACTTTACTCATTCTCATTTCTAATTTCTATTAGAATTAT

ATATATTTTATTTCTTTACTATACTATAACTATAAAAGAAAAAGAAAACTATAAAATAAA

AGAAAATCTATAAAATCTCATTTTCATATTTTTTTTTTCTTTTATACATTTTACATAATT

AATTCTAATTTTATATTTATATATTATTTCTAATCTAATCTAAAATTAGAATAATTCTAA

AATGATAAAAATAAAAATGAAATAAAATAGAATTAAAATTTAAATTCAATTTAAATATCT

AAAATTAAAATATAGAATATAGAATAGAAATTCTTATATTTTATATTATTAGAATTAGAA

GAAATATTCTAATAACTAAATATTTCTATCTCTAGAACAGAAATTCGATTCTACTAAATT

CTAAATTAAAAGAAATTAAATTAAAATAGTAATAATTTAAATAATAAAAATTACTTATAC

TTATATACTTTATACTTATATACTTCTATTTTATTTAATAATAATAATTATATACTAATA

ATTTAATAATAATAATTATATACTAATAATTTAATAATTATAATAATCATATTATAAATG

ATGTTATAAGTTATAAAATAATAAATAAAAAAAAAAAGAATTAAGGTGCATGATCCCG-A

CGAATTACTTCTGAATA----------AATAAAGA----AATC-----ATATGTAGGAAC

TATAGCATTTCGTGACTCATTGGT-----AAATTCACTTTGA-----TTCTCTATCAACC

AATCCAATAATG-TAGGACCC-----TTTACATGGTTAAAGTT-AAA--GTTTGAAGTCC

AGGC--ACAACGG-----GGTACTCTTTCTACCACTGCATTCGTA--------GAGATGG

TT-------TCAAAACG----------------AAGAGTTGGTAATTTATCCGATATAGA

ACACTCATATCGATAAAATAATTGTGA-----ACCATTCACTGT----------------

--AA-AAAAAATGGGCACTCCGACTTTTT----TTATCCAATGCTGAATTGACGACCTAT

GTAG----------------------AATAAAAA--TTCTTTGGATT--------TAAAG

AT----------------------------------------------------------

------------------------------------------------------------

------------------------------------------------------------

----------------------------------------------------------AA

TCAAATAAAAGAAACGACTTTCCTGACAGTT-------------ACCTGGTCGGAAGAGC

CC-----------TCC--GGGGAT-----CCTGGTCTT---------GTATCAGTTT---

-----------------------------------------CGATATCTTAGAAGACGAA

GAG------------AGAGGGTAGGCTCATT----------AAAAGATACGGGAGTGTCC

CATA---------------------A--GTAACTGAGCGTGAGAGCCAAATGAATTGAAA

GATTCATGTTCGGTTCGGGAAGAGATCA-TAGAAGTTGTGGAAT-----TGATATGGAGA

CAATCTACTTTCATTAAGTGATTTATTAGATAATCGAAAACAGAGAATCTTGAGTACGAT

TCGCAATTCAGAAGAACTACGTGGGGGAGCCATTGAGCAGCTTGAAAAAGCCCGGGCTCG

CTTACGGAAAGTGGAAATTGAAGCAGATGAGTTTCGAGTGAATGGATACTCTGAGATAGA

ACGAGAAAAAAGTAATTTGATTAATGCTGCTTATGAAAATTTAGAACGATTAGAAAATTA

CAAAAATGAAAGCATTCATTTTGAACAACAAAGAGCGATGAATCAGGTCCGACAGCGAGT

TTTCCAACAAGCCTTGCAAGGAGCTCTAGAAACTCTGAATAGTTATT------TGAACAG

CGAGTTACATTTACGCACCATCAGTGCTAATATTGGCATGCTCGGGACCATGAAA-----

AATATAACTGATTAGATGAATCCCCTGATTTCTGCTGCTTCCGTTATTGCTGCTGGATTG

GCCGTAGGACTTGCTTCTATTGGACCCGGAGTCGGTCAAGGTACTGCTGCGGGTCAAGCC

GTAGAGGGTATCGCGAGACAACCAGAAGCAGAGGGTAAAATACGAGGTACTTTATTGCTT

AGCCTGGCTTTTATGGAAGCTTTAACAATTTACGGACTGGTCGTGGCATTAGCGCTTTTA

TTTGCGAATCCCTTTGTTTAAATGAGTGTTCTACCATGTTCCATCAACACACTAAAAGGG

ATATACGATCTATCCGGTGTGGAAGTAGGCCAACATTTCTATTGGCAAATTGGGGGTTTC

CAAGTCCATGCCCAAGTACTTATAACTTCTTGGGTCGTAATTGCTATCTTATTAGGTTCA

GCCACTATAGTTGTTCGGAATCCACAAACCATTCCGACCGACGGTCAGAATTTCTTCGAA

TATGTGCTTGAATTCATTCGAGACTTGACCAAAACTCAGATTGGAGAAGAAGAATATGGT

CCCTGGGTTCCCTTTATTGGAACTATGTTCCTATTTATTTTTGTTTCTAACTGGTCCGGC

GCTCTTTTACCTCGGAAAATCATAGAGTTACCTCAGGGGGAGTTAGCTGCACCTACAAAT

GATATAAATACTACTGTTGCTTTAGCTTTACCCACGTCAGTGGCATATTTCTATGCAGGT

ATTAGCAAGAAAGGATTGGGTTATTTCGGTAAATATATTCAACCAACTCCCATACTTTTA

CCAATTAATATCTTAGAAGATTTCACAAAACCTTTATCACTTAGTTTTCGACTTTTTGGG

AATATATTGGCTGATGAATTAGTAGTTGTTGTTCTTGTTTCTTTAGTCCCTTCATTGGTT

CCTATACCTGTCATGTTCCTCGGATTATTCACAAGCGGTATTCAAGCTTTGATTTTTGCA

ACTTTAGCCGCGGCTTATATAGGCGAATCCATGGAGGGTCATCATTGAATGATATTCGCG

ACTTTAGAACATATATTAACTCACATCTCTTTTTCAATAATTTCAATTGTCATTACGACT

CATTTGATGACC---TTCGCCCGTGAAATCGCAGGACTATCTGATTTGTCAGAAAAAGGC

ATGATAGCTGTTTTTTTGCGTATAACAGGATTATTAGTCACTCGTTGGATTTATTCAGGA

CATTTACCATTAAGTAATTTATATGAATCATTAATTTTTCTTTCATGGGGTTTCTCTCTT

ATTCATATGATTTCTAAAATACAGAACCATAAGAATT------TTT---TAAGTTCAATA

ACCGCGCCAAGTGCTATTTTAACCCAAGGCTTTGTCACTTCGGGTCTTTTAACTGAAATG

CATCAATCCGCAATACTAGTACCTGCTCTACAATCCCATTGGTTAATAATGCACGTAAGT

ATGATGTTGTTGAGCTATGCAGCTCTCTTATGTGGATCCTTATTATCAATAGCTCTTCTA

GTCATTACATTTAGAAAAAAGCTAGATATTATTTT---------------------CTTA

TTAATT------AGGTTATTCTCT---------TTTGGTGAGATCCAA------TATTTG

AATGAAAA---AAGA---------AGTATTTT---GCAAAACACTTCTTTT------TCA

TTTAGAAATTACCACAAATATCAACTTACTCAGCAATTAGATCATTGGAGTTATCGTGTT

ATTGGTATAGGATTTACCCTTTTAACTTTAGGTATTCTTTCAGGGGCGGTATGGGCTAAT

GAGGCATGGGGATCCTATTGGAATTGGGACCCCAAGGAGACTTGGGCTTTTATTACTTGG

ACTATATCTGCGATTTATTTACATACTAGAACAAATAAAAACTTGCAAAGT------GCG

AATTCAGCAATTGTGGCTTCGATAGGATTTCTTATCATTTGGATATGCTATTTTGGGGTC

AATCTATTAGGAATAGGCCTACATAGCTATGGTTCATTC------GCATTA-------AG

G------------------------TAA--------------------------------

------------------------------------------------------------

------------------------------------------------------------

------------------------------------------------------------

----------GTGTCCAAAAAGAAAGCATTGACTTCCCTGCCATATCTTGCATCTATAGT

TTTTCTGCCCTGGGGAATTTCTCTATCATTTAATAAAAGTCTGGAACCTTGGGTTACAAA

TTGGTGGAATACCAGCCAATACGAAACTCTTTTGAATGAAATTCAAGAAAATAACGTTCT

TGAAAGATTCATAGAATTAGAAGAACTATTCTTGTTGGACGAAATGATAAGGGATTATCC

GGAGACACATATACAAAAGCTTCGTATAGGAATCCACAAAGAAACGATAGAAT-TGGTGA

AAATGTACAATCAAGATTATATCCATATAATTTCGCATTTCTCGACAAATATAATGAGTT

TCGCTATTCTAAGTGCTTATTCTATTCTGGGTAATGAAGAACTTGTCACTCTTAATTCTT

GGGTTCAGGAATTCTTCTATAACTTAAGTGACACAATAAAAGCTTTTTCTATTCTTTTAC

TAACCGATTCATGTATCGGATTTCATTCGCCCCATGGTTGGGAACTCATGATTGGTTCGG

TCTACAAAGATTTTGGATTTAGTCATAACGATCAAATTATATCCGGTCTCGTTTCCACTT

TTCCGGTTATTCTAGATACTATTTTGAAATATTGGATCTTCCATCATTTAAATCGTATAT

CTCCTTCACTTGTAGTTATTTATCATTCAATGAATGA---ATAAATGCCCGTTGGTGTTC

CAAAAGTACCTTTTCGGAGTCCTGGAGAG---GAAGACGCAACTTGGGTTGACATATAGT

GCGACTTGTCTGACATATTGGGTCATATGGAATTCC-CCCGTTCTCTC-----CCCCGAT

CGAGATATCCTCTCTTTCGCCCAA----------------GAAAG-----------ATTG

ATTGAACCATCAAT----------AATTCGGAGCGTGAA--GTGCAATTA-ATCCTTTTG

TTTTGGGGATAGATATT----ATCAA-TTAGAATAATCTATGGTTGGCTTGGACCAAT--

-----AAAATAGAGTAGCCTATCCAGGCTCCGTTCAGAAAATATCAAATTGGTATTTG--

------------------------------------------------------------

------------------------------------------------------------

------------------------------------------------------------

--------------------------------------------GTAGTAGTAATATATG

TATGAT----TACTACTTAATATCATAAT---CATAAATTATTACTATCCATATC-----

--------------GATCAGTGATCCC-----------AAACTCCC-----AACTTATCG

AGTAATAGCAT--AA---ATACATCGAATAT----TTGGTGGAAGACATGAAGCAAATTG

A---------------AAAAAAAAAAGTTGAATCTAATACTAATAAAATCTAATACTTAC

TAATAAAAGAGGTGGTAA---TAGGATCGTTTGTCCTATATGTGCAAATAGAATTCGGGC

ATATCTTTACCCGGAGTAG----AGCATAAACCTAAAAG--------------ACAAGAA

TTGAAG-AGGCCCATTCAGGAACAAGAAAATTACACCGTGATTTTGATCTCGATGAAAGA

ATA-CATCAATAAGAG----------AGTGATTCAATAAGTTCAGTGAATTCTTTTTTTT

TTTTTCT------------------------TTTAAGGGGGTGAACCAAAACTC------

--------ATGTTTCTTGAAAAAGGAAGAAAAAGCCCCTTCGGGAGG-----AAAACCCT

GCTAC---GAAAAAAGGAATAGGTCTGTAATCGGCACATTGATTCTTTTTTTTTTTTTTT

CGCAATTTTT-----GAACCGTATGCATCGAAAGGTGCGTGTACGATTCCTAAGGGATAC

AATTTGATCCTAATCAACCGACTTCATCGAGAAAGATTACTTTTTTTAGGCCAAGAGGTT

GAAGGTGAGATATCGAATCAAATTGTTGGTCTCATGGTATATCTCAGTATAGAGGATGAT

ACCAGGGATCTCTATTTGTTTATA--AACTCTCCCGGCGGATGGGTAATACCAGGAATAG

CTATTTATGACGCTATGCAATTTGTACCGCCAGACGTACATACGATATGCATGGGATTAG

CCGCTTCAATGGGATCTTTCGTCCTGGCCGGAGG-------AGAAATTACCA---AACGT

CTAGCATTCC-CTCACGCTTGGCGCCAATGAGTTTTTATTTATATTATTTATATTTAGTA

GAAAAAAAAGACTATGCC-TTCGCCATATGAAATATGAAAAAAAAAAATCAAAAATCAAT

CATAAATGAATATAAAATTCCAAAAGAAATAGAAATAAAATCTAATATAAAACATAGAAT

ATTAATATTAAAGAAGATAAAGAAGAAAGAAGTAATAATAGCATGGCACTTCTAATTCGA

TATGAACAAACCCTCGTTGTTT--------TTTTTATAACATGA--TTATGTATCGAAAT

AGTAGTATGAAA--AAAAGTTATTCCCAAT----TTGATCTTAT-------GTAT----C

GGGGGTACATT--TCAGCGTCACAAACCTTTTTGCTCCTACACCAGA----AGTCTCTTT

CGTATTTTTATG----AAAGAAAAGAGGGATAAAATGAAAGAAAAATTC--------TTC

TTTTTCTCTTTTTTTTTATTTGA---TCGGATCAGAAAGAAACTTTGGGATTGCTGAATG

AAAAC-----------------------TTCAAAAGCAACCGAACCATCATAGTAT----

-------------------TTTTGACTCCTCCG-----AGGAAAAAGGTGGTAAATGAAT

CATCTAAAGAT----CTGGGTCTAATAGATTCTATTTGTCTC-----------TTTCTC-

------------GGATAGAAGAGTCGATTCCATTGCAGAGCCGTATGCAACGCACAAA--

--GCCTGTACGGTTGTTCAATTCTATCTTTTTTTTTGTTATTCTTTTTCTTTATTCATTC

CC-----------TTCTGTCCGATTATGCGA-----ATATAGAAGAACCGCTCAC----A

ATGTCATATTAATC-------ATCAGGGTTATGATCCACCAACCCGCTAGTTCTTTTTAT

GAGGCACCAGCAGGAGAATTTATCCTGGAAGCGGGGGAGCTACTGAAACTTCGC----GA

AACCATCACAAGCGTTTATGTA--CAAAGAACAGGCAACCCTTTATGGGTTGTATCCGAA

GACATGGAAAGGGATGTTTTTATGTCAGCAACAGAAGCCCAAGCTCATGGCATTGTTGAT

CTTATAGCAGTCGAAA------------AT------ACCT-------GGGATTTCCCGTA

A-----------------ATGAAAGAACA-------------------------------

-----AAAATTAATTCAT---GAAGGTTTAATTACTGAATCACTTCCCAATGGCATGTTC

CAAGTT------CGCTTAGATAATGGAGACCTGGTTCTAGGTTATGTTTCAGGAAGGATC

CGACGTAGTTTTATACGGATATTACCAGGCGATAGAGTGAAAATTGAAGTAAGTCGTTAT

GATTCAACCAGGGGACGTATTA--TTTATAGGCTTC------------------------

------------------------------------------------------------

------GCAACAAAGATTCGAATGAT------------TAGATGGAGGAAT---------

---TACGAGGATATTTAG-----AAATAGATAG----ACCCCATCAACAACGC------T

TCCTATATCCGCTTCTATTCCAGGAGTCTATCTATGCACTTGCTCATAATCATGGTTTAA

ATGG------ATCAATTCTTTACGAACCCATGGAAGATTTAGGTC------ATGACAAGA

AATCCAGTTCACTAAATGTAAAACGTTTAATTATTCAAATGTATCAACAGAATCATTTCA

TCATTTCATTTAATGATTCTAACC------AAAATAGATTTCTTGGGCATAGCAGGAA--

-------------TTCTTATTTTCAAATGGTATCAGAGGGTTTTGCAGTAATTGTGGAAA

TTCCATTCTCGATGCGATTAGTATCTTCCCTA---GAAA---------AAGGATCTGAAT

CTCA------TAATTTCCAATCTATCCATTCAATATTTCCGTTTCTAGAGGACAAACTTT

CATATTTAAATTATGTGTTAGATATACTAATACCCCACCCAATCCATCTGGAAATCTTGG

TTCAAAGCCT------TCGCCAATGGATACGAGACCTTCCTTCTTTGCATTTATTGCGGT

TTTTTCTCCACGAACATCAGAATTGGAATAGTTTC---ATTACTACAAATACAAAGA---

------AATGCAGTTCTTTT------TTTTCAAGAGAGAATCAAAGATTGTTTTTGT---

TCCTGTACAATTTTCATGTATATCAATTTGAATCCCTTTTCGTTTTCCTACGTAAAC---

AATTTTTTCATTTACGGTCGATATCTTTTGGATCTTTTCTTGAACGAACACA-TTTCTAT

GGAAAAATAGAAA---------------------ATTTTG---------TAGTATCCATG

CGT---AATCATTC------TCA--GAATATTTTA------TGGTTGTTCAAGGACCTTT

TCATGCATTATGTTAGATATAAAGGAAAATCAATTATGGCTTCAAGGGGTACTTATCTCC

TTATGAATAAATGGAAATCTCACCTTGTCAATTTCTGGCAATTCCGTTTTTACTTCTGGT

CTCAACCGGGCAGAATCCATATAAATGAATTATCCAATCATTCCTTCTATTTCCCGGGCT

ATCTTTCAGGTCTACGAT-----TAAATCCCTCGATGGTAAGGAGTGAAATGCTAGAGAA

TTCATTTATGATAGATGCTGTTATCAAG------AGATTCGATACAGTAGTCCCAACTAT

TTTTCTTATTGGATCCTTGGCTAAAGTAAAATTATGTAACGTATCAGGGCATCCTATTAG

TAAGTCGGTCTGGGCCGATTCGTCAGATTCTGATATTCTCGATCAATTTGGGCGGATATG

CAGAAATCTCTCTCATTATCACAGTGGGTCCTACAAAAAACACAGTTTGTGTCGAATAAA

ATATATACTTCGACTTTCGTGTGCTAGAACTTTGGCTCGTAAACATAAAAGCACGGTACG

CGCAATTTTGAAAAGATTAGGTTCGGAATTCTTGGACGAATTCCTTACGGAGGAACAAGA

GATT--------CTTTCCTTGATT-----TTTCC---------AAAAACCCCTTTC----

-----------CATA---GTGGACGAATTTGGTATTTGGATATTATCCGTATTCATAGTC

TGGCCAATC------------------------------------------ATTGA----

--------------------------------------------------ATGATAATTG

ATACGAC------AGAAGTACAAGCTATCAATTCTTTTTCCAGATTGGA------ATCGT

CAAAAGAGGTCTATGGTCTGATATGGCTATTTATCCCTATTTTTACTCCTGTATCAGGGA

TCACAATAGGGGTATTGGTTATTGTGTGGCTAGAAAGAGAAATATCTGCAGGGATACAAC

AACGCATTGGACCTGAATACGCTGGTCCTTTGGGGATTCTTCAAGCTATAGCAGATGGGA

CCAAATTACTTTTCAAAGAGGATCTTCTCCCATCTAGAGGAGATATTCGTTTATTCAGTA

TCGGGCCTTCTGTAGTGGTCATATCAATTTTACTAAGTCATTTGGTAATTCCTTTCGGTT

ATCGCCTTGTTCTCGCCGATCTAAGTATAGGTGTTTCTCTATGGATCGCAATTTCCAGTA

TTGCTCCTATTGGACTTCTTATGTCAGGATATGCATCAAATAATAAATATTCTTTTTCAG

GTGGTCTACGAGCTGCTGCTCAATCTATTAGTTATGAAATACCGTTAACTCTGTGTGTGT

TATCAATATCTCTACGTGCGATTCGTTAGAACATTAACTTTTACGTCTTTTCTTTTTTTA

GTATTTTAGTATTAGAATCTAATATTAGGTTATAGATAGGTATATAAAGAAAGTATAGGA

TCAGAGGAAAGAACGAATAAAGAATATATATATATATATATTCTACTCTACGTTATTGAT

TCTACGTTATTGATTCAATGATTCCGATTGATGAGCTAAACCAG------ATAGTTATAT

GAGTGAAACAAAACTGCTTTGCTTATGGATTTGTAGTAAGAAAATGGAGTCTCATTCCCT

AT-GTACAAGAGGAA--------CCAACATAAG-----CAGTGGAAACTGTTTACCCCAA

GCCCAAGATTAGTTGATTAGTCATCGTATCGTGGCTTGAAGCGGGA----GCAAAAGATC

AACCA--TATGGAGTTTCTCCTATTCTATAGTATTT------------------ACCTAT

TACCATAGCGGAGATCAATCAAAAGTGAGTGGACGGTTAGAAACA-CTAAGGTGCA----

-------CAAAGGGTTAGTAATGTAGATAATGTAACGTATCCAAAAATG-----GATTTC

TGCATAAGGAGAAGTAAAAGGAATCAGAAT-----GAGGGTTTGACGTTGGTAGAAATGA

TCAAGCAGTACTTCCTTATGATCCCGATCCAGAGTAT-ATTCCTATCCACTAGTTAAAGA

AG---AACTATCAGG-AACGAAGTAATCCTTTATCTTTAT-----------ATAGTCCCC

----TCTGAGAAAGAAGAATAGGAACGAAGGGAATGCAATAGAAAAATGAA---------

-----------------TACTAAGAGATCTTTCTTT------------------------

-----CTTTTTTCTTTTCTCTATTAATAATTCATC----CTCATCCGGATGGAATTATCA

TGACTTATCAATTACATGCCTCATGAACTAGCTTGTTCCCTTGTACTATACT-TATTTTT

CTTCGTAATCGAAAGATGAAAGATATGGGTCGAAATATCTCTTGAGACAACGAGTATTTT

AT------TGAAAGATCGAGTTATTACTGA-ACAACAAA--------------TAATGGG

TAAAGGATAAGATAGATT-CAGAAGCACCCCCTTTTTTT-------------TTATAGCG

GACAGAATCCCATTGGTCTAATTCC-----GGACATTCCGATCCATCTCTACTCTACGGA

T------------------------------------------------ATGCCGAGGCA

AGACTAAGGTTTCAGTCCTTAGATTTATGT----GACCATCGAGGAGCCGTATGAAACCG

AGGTATCATGTACGGTTCTGGAATAGAGATGATAACAGTGATGTTATCATCGACTATGAT

TATCTAACAGTTCAAGTACGGTTGATATAGTGGAGGCACAGTATAAATATGGTTTTTGGG

GGTGGAATCTGTGGCGTCAACCTATAGG-GTTTCTAGCTTTTCTAATTT--CTTCCTTAG

CGGAATGTGAGAGGTTGCCCTTTGATTTACCAGAAGCGGAGGAAGAATTAGTAGCGGGTT

ATCAAACCGAATATTCAGGTATTAAATTTGGTTTATTTTATCTTGCTTCTTACCTAAATC

TACTAGTTTCTTCATTATTTGTAACGGTTCTTTATTTGGGTGGGTGGAATCTTTCTATTC

CATACATAGCAATTCCTGAGCTTTTCAGA---------ATAAATAGAATTGGTGGAGTTT

TTGGAACAACAATTAGTATCTTTTTTACATTAGCTAAAGCTTATTTGTTCCTGTTCATTC

CTATCACAACAAGATGGACTTTACCTAGGATGAGAATGGACCAACTATTAAATCTTGGTT

GGAAATTTCTTTTACCTATTGCTCTAGGTAATCTATTACTGACAACTTCTTCCCAACTTT

TTTCGCTGTAA-------ATGATCTGGCATGTACAGAATGAAAACTTCATTCTCGATTCT

ACGAGAATTTTTATGAAAGCCTTTCATTTGCTTCTCTTCCATGGAAGTTTCATTTTCCCA

GAATGTATCCTAATTTTTGGCCTAATTCTTCTTCTAATGATCGATTCAACCTCTGATCAA

AAAGATAGACCTTGGTTCTATTTCATCTCTTCAACAAGTTTAGTAATGAGCATAACGGCC

CTATTGTTCCGATGGAGAGAAGAACCTATGATTAGCTTTTCGGGGAATTTCCAAACGAAC

AATTTCAACGAAATCTTTCAATTTCTTATTTTACTATGTTCAACTCTATGTATTCCTCTA

TCAGTAGAGTACATTGAATGTACAGAAATGGCTATAACAGAGTTTCTGTTATTCGTATTA

ACAGCTACTCTAGGAGGAATGTTTTTATGTGGTGCTAACGATTTAATAACTATCTTTGTA

GCTCCAGAATGTTTCAGTTTATGCTCCTA-----CCTATTATCTG-------G-ATATAC

CAAGAG---AGATGTACGG--TCTAATGA-GGCTACTACGAAATATTTACTCATGGGTGG

GGCAAGCTCTT-CTATTCTGGTTCATGGTTTCTCTTGGCTATACGGCTCATCCGGGGGAG

AGAT-CGAG-CTTCAAGAAATAGTGAATGGTCTTATCAATACACAAATGTATAACTCCCC

GGGAATTTCGATTGCGCTTCTATCCATCACTGTAGGAATTGGGTTCAAGCTTTCCCCAGC

CCCTTTTCATCAATGGACTCCTGACGTATACGAAGGAGTGCGGTTCGTTCGACAAATTCC

TACCTCTATATCTATCTCTATCTCAGAGATGTTTGGATTTTTCAAAACTCCATGGACATG

CAGAAGAGAAATGCTATCCCCACTCGGACCAAGACATAACTTTTACCAAAAGAAAAGTTG

ATTGTGATCTTTTTGT----------TCAAATAACAATTAAGG----TGAAGCAGGGTCA

GGAACAACGAATC-TCTTTAT-GATAAACAGATCCATTTTGCAAGCTCGTTATTA--CGG

GTAGTTCCTACAAAGGATCGGACTAATG-ACGTATACAATACTTGAATTCTCG-ATGTAG

ATGCTACATAGTTGGTTCTCAT-CCTTCAGAGACTACGAATGTAATAGGAG---CATCCG

TCGACAAAAAGATCGCC------------CTAAGATGAT---------------------

-CATCC--------CATGGCTATTGAGAACGAATCAAATCAGATGGTTCTATTTCTCAAT

C-TTTCTGACTTGCTCCTACGG--AACCCGGGTCGAAAAGATTGAAAAAAAATCAGTCAT

TCACAACCACTGATGAAGGATTCCTCGAAAAGTTAAGGATTAGTATAGTAATCCTTTTTA

GAAATCG------------AATGGATTCGGTCTTATACATACGCGAGGAAGGTAATCAAA

AAAGAAAGAAGAAC----------------------------------------------

------------------------------------------------------------

-------------------TC-ATCTTCTTTCTTTTATCACTTAGGAGCCGTGCGAGATG

AAAGTCTCATGCACGGTTTTGAATGAGAGAAAGAAGTGAGG-AATCCTC-TTTTCGACTC

TGACTCTCCCACTCCA-GTCGTTGCTTTTCTTTCTGTTACTTCGAAAGTAGCTGCTTCAG

CTTTAGCCACTCGAATTTTCGATATTCCTTTCTATTTCTCATCGAACGAATGGCATCTTC

TTCTTGAAATCCTAGCTATTCTTAGCATGATATTGGGGAATCTCATTGCTATTACTCAAA

CAAGCATGAAACGTATGCTTGCATATTCGTCCATAGGTCAAATCGGATATGTAATTATTG

GAATAATTGTTGGAGACTCAAATGATGGATATGCAAGCATGATAACTTATATGCTGTTCT

ATATCTCCATGAATCTAGGAACTTTTGCTCGCATTGTATCATTTGGTCCACGTACCGGAA

CTGATAACATTCGGGATTATGCAGGATTATACACGAAAGATCC-TTTTTTGGCTCTCTCT

TCAGCCCTATGTCTCCTATCCCTAGGAGGTATTCCTCCATTAGCAGGTTTTTTCGGAAAA

CTCCAT---------CTATTCTGGTGTGGATGGCAGGCGGGCCTATATTTCTTGGTTTCG

ATAGGACTCCTTACGAGCGTTGTTTCTATCTACTATTATCTAAAAAT-------AATCAA

GTTATTAATGA--CTGGACGAAACAAAGAAATAACCCCTCACGTGCGAAATTATAGAAGA

TCTC------CTTTAAGATCAAACAATTCCATCGAATTGAGTATGATTGTATGTGTAATA

GCATCTACTATACCAGGAATATCAATGAACCCAATTATTGCAATTGCTCAGGATACCTTC

TTTTAAATGTTTCTGCTTCACGAATAT--GATATTTTCTGGGCATTTCTAATG-----AT

ATCAAGTGTTATTCCTATTTTGGCATTTCTAATTTCTGGAGTTTTAGCCCCGATTAGTCA

AGGACCAGAAAAGGTCTCTAGTTATGAA-TCAGGTATAGAA-CCAATGGGGGATGCTTGG

A-TACAATTCCGAATCCGCTATTACATGTTTGCCCTAGTTTTTGTTGTTTT--TGATGTC

GAAACCGTTTTTCTTTATCCATGGGCCATGAGTTTCGATGTATTGGGCGTATCCGTATTT

ATAGAAGCTTTAA--TTTTCGTGCTTATCCCAATCGTTGGTTCAGTTTATGCATGGCGAA

AAGGAGCATTGGAATGGTCTTAG---ACGAATAATTTTCC------TTGGCTAACAGCGA

TTCTTCTTTTGCCCATATCCGCGGGTTCTTCAATTCTGTTTATACCTCAGAGAGGAAATA

AAGCGGTTCGGTGGTATACTATATGTATATGCTTAT------TAGAACTTCTCCTAATGA

CCTA-----------TGTATTTTATTATAATTT-CCAATTGGACGATCCATTAATCCAAT

TGGAAGAGGATTGTAACTGGATAAATCTTTTTGATTTTCACTGGAGACTAGGAATTGATG

GACTTTCCATAGGTCCCATTTTATTGACGGGATTCATCACTACTTTAGCTACTTCAGCGG

CTTGGCCAGTTACTCGAAATTCGCGATTATTTCATTTCCTGATGTTAGCAATGTATAGCG

GTCAAATAGGATCATTTTCTTCTCGGGACCTTTTACTTTTTTTCATCATGTGGGAGTTAG

AATTAATTCC-TGTTTACCTACTTTTATCGATGTGGGGAGGAAAAAAACGTCTGTATTCA

GCTACAAAATTCATTTTGTACACCGCAGGAGGTTCCATTTTTCTCTTAATAGGAGTTCTG

GGTATGGGCCTA------TATAGTTCCAATCAACCCATGTTGAATTTTGAAACATCAGCC

AATCAATCGTATCCCGTGGGATTGGAAATCCTATTCTATTTCGGCTTCCTTATTGCTTAT

GCTGCCAAACCACCGATTATACCCCTACATACATGGTTACCAGATACTCATGGAGAGGCG

CATTACAGTACATGTATGCTTCTGGCTGGAATCTTATTAAAAATGGGAGCATATGGGTTG

GTTCGAATCAATATGGAATTATTACCCCATGCTCATTCTCTATTTTCTCCTTGGTTGGTA

ATAGTGGGAACCCTTCAAATAATCTATGCAGCTTCAACTTCTCTTGGCCAACGCAATTTA

AAAAAGAGAATAGCCTATTCCTCCGTATCTCATATGGGTTCCACAATCATAGGAATTGGT

TCCATGACCGATACGGCACTCAATGGAGCCATTTTACAAATAATTTCTCATGGATTTATT

GGTGCTGCACTTTTTTTCTTGGCAGGAACAAGTTACGATAGAATACGGTTTCTTTATCTC

GACGAAATGGGAGGAATAGCT-------------ATACCAATGCCAAAAATATTTACTAT

GTTCAGTATCTTTTCGATGGCCTCTCTCGCATCGCCAGGAATGAGTGGTTTTGTTGCAGA

ATTTTTGGTCTTTTTGGGAATAATTACCAGCCCAAAATATCTTTTCATCCCCAAAATACT

AATTACTGTTGTAATGGCAATTGGAATGATATTAACTCCCATTTATTTATTATCTATG--

------TCACGCCAGATGTTCTATGGATACAAACTATTCAATGTTCCAAATTCTTACTTT

GTAGATTCTGGACCGCGAGAAATATTTATTTTAATCTCTATC-CTTCTACCTGTAATG-G

GTATTGGTATTTAT---CCTGATT-TCGTTCTTTCACTATCAGTTGATAAGGTAGAAGCT

ATTTTAGCAAATTACTTTAATGGATAGATGATGTTCGAGTATGCACTTGTTTTAAGTAGT

TATTTATTTTCTATGGGTATCTATGGATTAATCACAAGTCGAAACATGGTTAGAGCACTT

ATGTGTCTTGAGCTTATACTGAATGCTGTTAATATGAATCTCGTAACATTTTCTGATTTA

TTTGATAGTCGACAATTAAA----------------------------------------

-----------------------------------GGGAGACATTTTCTCGATCTTCGTT

ATAGCTATTGCAGCCGCTGAAGCAGCTATTGGCCCAGCTATTGTTTCATCAATCCATCGT

AACAGAAAATCCACTCGTATCAATCAATCAAATTTGTTGAAGAAATAGATGGAACGTACA

TATCAATATGCATGGATCATACCCTTCCTTACGCTTGCAGTTCCTATTTTGATAGGATTG

GGACTCCTGCTTGTTCCCCCGGCAACAAAAAGTATTCGTCGGATATGGGCTTTTCCCAGT

GTTTTATTGTTAAGTATAGTTATGGTTTTTTCTACCAATTTGTCTATTCAGCAAATAAAT

GGTAATTCGATTTATCAATATCTATGGTCCTGGACCATCAATAGTGATTCTTCCTTAGAG

TTCGGCTATTTGATCGATTCACTTACTTCTATTATGTCAATATTAATTGCTACTGTTGGA

ATGATGGTTCTTATCTATAGTGATAATTATATGTCTCATGATAAGGGATATTTAAGATTT

TTTGCTTATATGAGTTTTTTCAATACTTCAATGTTGGGGTTAGTTATTAGTCCCAATTTG

ATACAAATCTATATTTTCTGGGAACTTGTGGGAATGTGTTCGTATCTATCAATAGGTTTT

TGGTTTACTCGACCAAGTGCAGCAAATGCTTGTCAAAAAGCGTTTGTAACTAATCGTGTA

GGGGATTTTGGTTTATTATTAGGAATCTTAGGTCTTTATTGGATAGCGGGTAGTTTCGAA

TTTCGGGATTTGTTTGATATATTCAATGATTCAATCGATAATATCGATAATAATGTGTTG

AATTCCTCTTTTGCTATTCTGTGCGCCTCCTTATTATTCCTGGGTGCAGTTGCTAAATCC

GCACAATTCCCCCTTCATGTATGGTTACCTGATGCCATGGAGGGACCAACTCCTATTTCG

GCTCTTATACATGCTGCTACTATGGTAGCAGCAGGAATTTTTCTTGTAGCTCGGCTTCTT

CCTCTTTTGACAGTTATACCCTACATAATGAATCTTATTTCTTTGATAGGTGTAATAACA

GTACTATTAGGAGCCACTTTGGCTCTTGCTCAAAAGGACATTAAAAGAAGTTTAGCTTAT

TCTACGATGTCTCAACTGGGTTATATTATGTTGGCTTCAGGTATAGGTTCTTATCGAGCC

GCTTTATTCCATTCAATCACTCATGCCTATTCGAAAGCATTATTGTTTTTAGGATCTGGA

TCAATTATTCATTCTATGGAACCTATTCTTGGGTATTCTCCAGATAAAAGCCAGAACATG

GTTTTTATGGGTGGTTTAAGTAAATATGTGCCGATAACAAAGGCTACTTTTTTGTTAGGT

ACGCTCTCCCTTTGTGGTATTCCACCCCTTGCTTGTTTCTGGTCCAAAGATGAAATTCTT

AATAATAGTTGGTTGTATTCGCCAATTTTCGCGATAATAGCTTCTTCTACAACAGGATTA

ACTGCATTTTATATGTTTCGTATGTATTTACTTACTTTTGAAGGGCATTTACGCGTTCAT

TTTAAAGGTGA---------TACAAATAGTTCCTTGTATTCAA------TATCTATATGG

GGGAAAGAAGGACCAGAAGTTTTTAGCAGAAAT----TTGATTTTAT----TATC---AA

TGAATAATAATCAAAATGAAAAAG---------TTTCTTCT------------TTTTTGA

ATAAGAATAAGATATATCA----G--ATTGATAGAGATGTAATAAA---A----------

ATGCGATCCT---TTAGCACTCATTTTGTCAAAAAAGAA------ACCTTTCCCTATCCC

CACGAATCGGACAATACTATGTTATTCCCCTTACTTTTATTGGCCATATTGACTTTGTTC

GTTGGGTCCGTAGGAATTCGTTTCGGTCAA---------------GGAGTAACGGACTTT

GACGTGTTATCAAAATGGTTAATTCCATCAATATCAATGGACCTTTTTCATGAGTATTTG

AATCCTTCGGCGGATTGGTATGAATTTGCACAAAATGCAATTTATTCAGTCAGTATAGCC

TTTTTTGGAATATTAATA-GCCAATCTTTTATATGGGTCTGTTCATTCATCTTTCCAGAA

TTTGGACTTAATCAACTCATTTGCAAAGATATATACTAAGATAAGAAT------CTTTTC

GGACCAAGCACTAAATGTCATATACAATTGGTCATATAATCGCGGTTACATAGATCTTTA

TTATGCAACAATCTTAACTAGAGGTATACGAGGATTAGCTGAATCCACTCATTTTTTTGA

TCAACGGATAATTGATGGAGTCACGAATGCAGTTGGTATTACAAATTTCTTTGTAGGAGA

AGCGATCAAATAT---ATGGCGGGTGGAAGAATCTCTTCTTACCTCTTTTTTTCGTTATC

CTCTTTATCAATAGCC----------CTTATTT------TAGTATATTTTT---ATTTGT

ATTTTTGA--------------------------------------ATGGATTTACTTGG

ACCAATACATGATATTCTTTTGGTATCTTTGGGCTCAGGTATTATATTAGGAAGTTTAGG

AGTAGTATTACTTACCAATCCTATTTATTCTGCCTTTTCATCGGGATTGG---TTCTTGT

TCGTATATCCTT-ATTCCATATTCTATCAAACTCCTATTTTGTAGCTGCTGCACAGCTCC

TTATTTACGTAGGAGCCATAAATGTCTTAATCATATTTGCTGTGATGTTCATGAATGGTT

CAGAATATTACAATAATTTCCATCTTTGGACCGTTGGAGATGGGATCTCTTCAGTAGTTT

GTACAAG---------TATTCTTTTTTCACTAATTGCT-ACTATTCTAGATACGTCATGG

TACGGGATTATTTGGACTACAAGATCAAATCAGATCATAGAGCAGGACCTTACAAGTAAC

GTACAACAAATTGGGATTCATTTATCAACTGATTTTTTTCTTCCATTTGAACTCATTTCT

ATAATTCTTTTAGTTGCCTTGGTAGGGGCAATTGCTATGGCTCGTGCGGAA---------

TAA---------------------ATGACTGTACCAGCTACAAG------AAACGACCTC

ATGATAGTCAATATGGGTCCTCACCATCCATCAATGCATGGTGTTCTTCGACTCATAGTT

ACTCTAGACGGTGAAGACGTTATTGACTGTGAACCCATATTAGGTTATTTACACAGAGGG

ATGGAAAAAATTGCAGAAAATCGAACAATCATACAATATCTTCCTTATGTAACACGTTGG

GATTATTTAGCTACTATGTTCACAGAGGCAATAACAGTGAATGCACCGGAAGAGTTGGGA

AATATTCAAGTACCTAGAAGAGCCAGCTATATCAGAGTAATTATGTTGGAGCTCAGTCGT

ATAGCTTCTCATTTGTTATGGCTTGGACCTTTTATGGCTGATATCGGTGCACAGACTCCT

TTCTTCTATATTTTCAGAGAGAGAGAATTGCTATATGATCTATTTGAAACTGCCACAGGA

ATGCGAATGATGCATAATTTTTTCCGTATCGGAGGAGTAGCTGCTGATTTACCTCATGGC

TGGATAGATAAATGTTTGGATTTCTGTGATTATTTCTTACCAGAAGTTGCTGAATATCAA

AAACTTATTACACGTAATCCCATCTTTTTGGAACGAGTTGAAGGAGTGGGCTTTATTGCT

GAAGAGGAAGCAATAAATTGGGGTTTATCAGGACCAATGTTACGAGCTTCCGGAATACCA

TGGGATCTTCGTAAAGTCGATCATTA--TGAGTGTTACGATGAATTTGATTGGGAAGTCC

AATGGCAAAAAGAAGGAGACTCATTAGCTCGTTATTTAGTACGAATCAGTGAAATGATAG

AATCCGTCAAAATTATTCAACAGGCTCTAGAAGGAATTCCGGGGGGGCCCTATGAAAATT

TAGAAGCCCGACGC------TTTGATAGAGTAAGAAATATAGAAT----GGAATGATTTT

GAATATCGATTTATTAGTAA--AAAAC-------CTTCTCCCACTTTTGAATTGTCGAAA

CAAGA---ACTCTATGTGAGAGTAGAAGCTCCAAAAGGGGAATTAGGGATTTTTCTAATA

GGAGATAATAGTGTTTTTCCCTGGAGATGGAAAATTCGTCCACCCGGTCTTAT-------

---CAATTTGCAAATTCTTCCTCAGCTAGTTAAAAGAATGAAATTGGCTGATATCATGAC

GATACTAGGTAGTATAGATATCATTATGGGAGAAGTTGATCGTTGAATGTTCCCTATGGT

GACTGGGTTCATGAATTATGGTCAACAAGCAGTGCGAGCTGCAAGATATATTGGTCAAAG

TTTCATGATTACCTTATCTCACGCGAATCGTTTACCTGTAACCATTCAGTATCCTTATGA

AAAATCGATCACATCAGAGCGTTTCCGGGGGCGAATCCACTTTGAGTTCGATAAATGCAT

TGCTTGTGAAGTATGCGTTCGTGTATGCCCGATAGATCTCCCCGTTGTTGATTGGAGATT

CGAAACAGATATTAGAAAA---AAACGATTGCTTAATTATAGTATTGATTTTGGAATCTG

TATATTTTGTGGTAACTGCGTCGAGTATTGCCCCACAAACTGTTTATCCATGACTGAAGA

ATATGAACTTTCCACCTATGATCGTCACGAATTGAATTATAATCAAATTGCTTTGGGTCG

GTTACCAATACCTGTAGTTGGAGATTACACAATTCAAACAATTATGAATT------CGAA

TCAAACA---AAAATAGCTATGAATAAACCCCTT---GATTCAAGAACAATTACCAATTA

CTAG------------AT------------------------------------------

---GCAGGGTCGTTTATCTGCTTGGCTAGTCAAGCATGAGCTAGTTCATAGATCTTTGGG

CTTCGATTACCAAGGAATAGAAACTTTACAAATTAAATCCGAAGATTGGTACTCCATTGC

TGTCATTTCATATGTATATGGTTACAATTACCTACGTTCTCAGTGTGCTTATGATGTAGC

ACCAGGAGGATTATTAGCTAGTGTGTATCATCTTACGAGAATACAATATGGTGTGGATCA

ACCAGAAGAGGTATGTATAAAAGTATTTGCCCAAAGGAGGAATCCTAGAATCCCGTCTGT

TTTCTGGATCTGGAAAAGTTCTGATTTTCAAGAACGGGAATCTTATGATATGTTGGGAAT

CTATTATGATAACCACCCACGCCTGAAACGTATCTTGATGCCTGAAAGTTGGATTGGTTG

GCCCCTACGCAAGGATTATATTGCCCCTAATTTCTATGAGATACAGGATGCTCATTGA--

------------------------------------------------------------

------------------------------------------------------------

----------------------------------------------------------AT

GAATTCTATGGAGTTTCCGTTACTTGACCGAACAACCCCAAATTCAGTTATTTCAACTAC

ACCGAATGATCTTTCGAATTGGTCAAGACTCTCCAGTTTATGGCCGCTTCTATATGGTAC

CAGTTGCTGCTTCATTGAATTTGCTTCATTAATTGGCTCGCGATTCGACTTTGATCGTTA

TGGACTGGTACCAAGATCGAGTCCTA-GGCAAGCCGACCTTATTTTAACAGCCGGCACAG

TGACAATGAAAATGGCTCCTTCTTTAGTAAGATTATATGAACAAATGCCTGAACCAAAAT

ATGTCATTGCTATGGGAGCCTGTACTATTACAGGAGGAATGTTCAGTACCGATTCTTATA

GTACCGTTCGAGGAGTCGATAAGTTAATTCCTGTGGATGTCTATTTGCCGGGTTGCCCAC

CTAAACCAGAAGCAATTATAGACGCTATAACGAAACTTCGTAAGAAGGTATCTCGAGAAA

TCTATGAAGATAGAATCGCGT------CCCAACAGGAAGATCGATGTT----------TT

ACTACCAATCACAAGTTTCGGGTTGGGCGCAGTATTCATACCGGAAATTACGATCAAGAG

TTACTCTACCAATCGCC---------------ATCTACTTCAGAGATACTTTCTGAAA--

----------------------CA------CTTTTCAAATACAAGAGTTCACTATCTTCT

CACGAATTAGTGAAT--------TAG----------------------------------

---------------------------------ATGC---AAAATAGAAATACTTTTTCT

TGGGTAAAGGAACAGATGACTCGATCCATTTTTGTATCGATGATGATATATATAATAACT

CGGGCATCTATTTCAAATGCATATCCTATTTTTGCGCAGCAGGGTTATGAAAATCCGCGA

GAAGCAACTGGACGTATTGTATGTGCCAATTGTCATTTAGCTAATAAGCCTGTGGATATT

GAGGTTCCACAAGCTGTGCTTCCTGATACTGTATTTGAAGCAGTTGTTAGAATCCCCTAT

GATATGCAACTGAAACAAGTTCTTGCTAATGGTAAAAAGGGAGGTTTGAATGTAGGAGCT

GTTCTCATTTTACCCGATGGATTTGAATTAGCTCCTCCTGATCGTATTTCTCCCGCGATG

AAAGAAAAGATA---GGTAATTTATCTTTCCAGAGTTATCGCCCCACTAAAAAAAATATT

CTTGTGATAGGTCCTGTTCCTGGTCAGAAATATAATGAAATTCTCTTTCCAATCCTTTCC

CCGGATCCCGCTACTAAGAAAGAAGTTCACTTCTTGAAATATCCCATATATGTGGGTGGG

AATAGAGGAAGGGGTCAGATTTATCCCGATGGGAGCAAGAGTAACAATACAGTTTATAAT

GCCTCAGCATCAGGGATAATAAGTAGAATAATACGTAAAGAAAAG---GGTGGATATGAA

ATAACCATAGCTAATGCATTGGATGGGCGTCAAGTGGTTGATATTATACCTCCAGGACCG

GAACTTCTTGTTTCCGAAGGTGAATCTATCAAACTTGATCAACCATTAACGAGTAATCCC

AATGTGGGTGGATTTGGTCAGGGGG-ATGCA--GAAATAGTACTTCAAG-ATCCATTACG

TA-TCCAAGGTCTTTTTCTCTTTTTGGCATCTGTTATTCTGGCACAAATCTTTTTGGTTC

TTAAAAA-GAAACAGTTTGAGAAGGTTCAATTGGCTG-AAATGAATTTCTAGATGAGTGT

GTGACTTGTTATAA-TTGATCCTATTGTATAGTACAGAGAGTGGATCTGTCGTC---TCG

ATAGAGATGGTTCT-ATCTCGTCGGATA-------TTCAG----TCTAGTATCTGAAACA

CGG-----ACTCTTTATT--CTATTATATGGAATAGATAAATAAAAATTTGAACTATGAT

TCATACTTATTTT--TTTATTCAGACCTCGTGACCGGGCTCAAAAAAAATTCTCAAAGAT

CTA-------------------TTTCATAAATCGAACGATTCCTTTTTTTTATTCAGAAT

CATG-----CTTACTTCAACTAAAGGACAAA-----TATTTCTCTGGATTT---TTAGTC

ATT----ATA--TCTTTGAAT----------AAATGATGATCAAAC-----GGTTCCT-A

CTCAGAGAACCTTTTGTTTTTGAGGGACTTT-------GGTGAATCATCGTGGTTTTAAT

ATGAATCTAAGGTTTCAAT-----TGATTCGTAGGGTCTCAACAAGAGAATTCCTATCAA

TTGTATGGTACTTCCTT--------------------------------AAGTAAATCCG

TATTACGCACAAAAAAATA---------------------AAATAGGGTAAGAGAACATT

CAAGAGGCC----T-GTAACG----------ATCGACAT---------AAAGACAAGACG

ACGCGCCAACTTGA------------------TATTTTGGCATTATACATCACCAATCAC

AAAGAAGAGATTCTGGATTTTAGCTC-----TTTCGTATCTTC-------ATGGAAGATT

GAATCAA-----GTAGCTAA-------AAAGTTTCAAATTT---------------CAAT

ATTCGTTG-CAACCAGTATTGG------GGTGTTTTCGCTTGAGCCGTACGAGACGAAAT

TTTCATATACGGTTCTCAGAGGGGG-AGTCTCTTTGGTTTACCTATCTCAATAAAGTATA

TGATTGGTTCGAGGAGCGTCTCGAGATTCAGGCGATTGCGGATGATATAACTAGTAAATA

TGTTCCTCCTCATGTCAATATATTTCATTGTCTAGGGGGAATCACACTTACTTGTTTTTT

AGTACAAGTAGCTACGGGTTTTGCTATGACTTTTTACTATCGTCCAACTGTTACAGAGGC

TTTTGCCTCTGTTCAATATATAATGACTGAAGTAAACTTTGGTTGGTTAATCCGATCAGT

TCATCGATGGTCGGCAAGTATGATGGTTCTAATGATGATCCTGCACGTATTTCGTGTTTA

TCTCACAGGCGGATTTAAGAAACCTCGCGAATTAACTTGGGTTACGGGTGTGGTTCTGGC

TGTATTGACTGCATCTTTTGGTGTAACTGGTTATTCCTTACCTCGGGACCAAATTGGTTA

TTGGGCAGTCAAAATCGTGACCGGCGTGCCTGAGGCTATCCCTATAATAGGATCGCCCTT

GGTAGAGTTATTACGTGGAAGTGCTAGTGTGGGCCAATCTACCTTGACCCGTTTTTATAG

TTTACACACTTTTGTATTACCTCTTCTTACTGCCGTATTTATGTTAATGCATTTTCCAAT

GATACGTAAGCAAGGTATTTCGGGTCCTTTATAGATGGGAGTGTTTGACTTGAACTA-TT

GATTGGGCCGTGCAGATATA-TGAATTTATCTGCCACATTGAAATTCACAACC-----AA

ATGTGTCTCTG--TCCAAC-----CACCG-----CGTAAATTCCCC------------CT

ACAGAGGACAGGCTGGTTCGCTTGAGGAGAATATTTTCTATGATCAAAGCCGA--AT-CA

TGTCATGCATGAGCAGGCTCCGTAAGATCCATTAGAATAA-----GTGAAAGTGATTTGA

CATGAGTCAGATTGTGTTCTATCTATTTCACTTACCT--------------------ATA

A-------------------------------------------------CCTATAGTAT

GGAAATGCATTCATTTCCCCTGCATTGATCCT--CGATCTATGATACTATCGGAGTGAAA

CAAGAGATCTAAGGAAGAACAGAGGTTAGACTTT----------ATTAGTAACAAGTAAA

TCCTTTG----TATGTACGAAAAGAAGATTCGAGA----TATTGTGGGGATAAAGATC--

-----AATCACAAGGCATGAGACGATCCAAAAAGCG-CTTGGTAATGATC-AAAATTAAA

ATTGCAAGCCTACTTGGGTATTGAGCATTTACTTGTAA-----GAACTGAATTCCTTGTA

ATGGA-----TA-------GTTGCAACTCCGTATAATTGAATCCGGGAAATCTCTT----

-------CTTACATAAAGTCAT-------------------------ATATATGTGATGT

GGTGGATATC------------------------------TATGATTTTATTTTACCATT

TCATTTGATTCTTTTGTTGCTCGAGCCGGACGATGAAAAATTATCATGTCCGGTTCTTTC

GGGGGATGGATCCA-------TAAGAATTCACCTATCCCAATAACAAAGAAACCTGACTT

GAATGATCCTGTATTAAGAGCTAAATTGGCTAAAGGGATGGGACATAATTATTACGGAGA

ACCCGCATGGCCTAATGATCTTTTATATATCTTTCCAGTAGTAATTTTAGGTACTATTGC

ATGTAACGTGGGTCTAGCTGTTCTAGAACCTTCAATGATTGGTGAACCAGCAGATCCATT

TGCAACTCCTTTGGAAATATTACCCGAATGGTACTTTTTTCCCGTATT------------

------------TCAAATACTTCGTACAGTACCTAATAAGTTATTGGGTGTCCTTTTAAT

GGTTTCGGTACCAACGGGATTATTGACAGTACCTTTTTTGGAGAATGTTAATAAATTCCA

AAATCCATTTCGCCGTCCAGTAGCTACAACAGTCTTTTTGTTCGGTACCGCACTAAGTCT

TTGGTTAGGTATTGGAGCAACATTACCTATTGATAAATCCCTAACTTTA-GGTCTTTTTT

AA----------------------------------------------------------

-----ATGATTGAAGTTTTTCCATTTGGAATCGTGTTAGGTCTAATTCCTATTACTTTGG

CCGGATTATTTGTAACTGCATATTTACAATACAGACGCGGGGATCAGTTGGATCTTTGA-

------------------------------------------------------------

--------------------------------------ATGGATATAGTAAGTCTCGCTT

GGGCTGCTTTAATGGTAGTTTTTACATTTTCCCTTTCACTAGTAGTATGGGGAAGAAGTG

GACTCTAGATGATTATTCGTTCGCCGGAATCAGAAGTGAAGATTATGGTGGATAGGGATC

CCATAAAAACTTCTTTCGAGGAATGGGCCAGACCCGGCCATTTCTCAAGGACAATAGCTA

A---AGGCCCCAATACTACCACTTGGATCTGGAACCTACATGCTGATGCTCACGATTTCG

ATAGCCATACCAGTGATTTGGAGGAAATCTCTCGAAAAGTATTTAGTGCTCATTTCGGTC

AACTATCCATCATCTTTCTTTGGTTGAGTGGTATGTACTTCCATGGTGCCCGTTTTTCCA

ATTATGAAGCATGGTTGAGCGATCCTACTCACATCGCACCCAGTGCCCAGGTGGTTTGGC

CAATAGTTGGTCAAGAAATCCTGAATGGCGATGTAGGTGGAGGTTTCCGAGGAATACAAA

TAACCTCTGGTTTTTTTCAGATTTGGCGAGCATCTGGAATAACTAGTGAATTACAACTCT

ATTGTACCGCAATTGGTGCTTTGGTCTTTGCAGCGTTAATGCTTTTTGCCGGTTGGTTCC

ATTATCACAAGGCTGCTCCAAAATTGGCTTGGTTCCAAGATGTAGAATCCATGTTGAACC

ACCACTTAGCAGGGTTACTAGGACTTGGGTCTCTTTCTTGGGCGGGACACCAAGTACATG

TATCTTTACCCATTAACCAATTTCTAGACGCGGGAGTGGATCCTAAAGAGATACCACTTC

CTCATGAATTTATCTTGAATCGGGATCTTTTGGCTCAACTTTATCCCAGTTTTGCCGAGG

GATCAACCCCATTTTTTACCTTGAATTGGTCAAAATACGCGGAATTTCTTACTTTTCGCG

GAGGATTAGATCCAGTAACAGGGGGTCTATGGCTGACCGATATTGCACACCATCATTTGG

CTATTGCAATTATTTTCCTGATAGCTGGTCACATGTATAGGACCAACTGGGGAATTGGTC

ATGGCCTAAAAGAGATTTTAGAGGCTCATAAAGGTCCATTTACAGGCGAGGGTCATAAAG

GTCTCTATGAGATCC-TAACAACG--TCATGGCATGCTCAATTAGCTCTTAACCTAGCTA

TGTTAGGCTCTTTAACCATTGTTGTAGCTCACCATATGTATTCTATGCCCCCCTATCCAT

ACCTAGCTATTGACTATGGTACACAACTCTCGTTGTTCACACATCACATGTGGATCGGTG

GATTTCTCATAGTTGGCGCTGCTGCACATGCAGCTATTTTTATGGTAAGAGACTATGACC

CAACTACTCGATACAACGACCTTTTAGATCGTGTCCTTAGGCACCGTGATGCAATCATAT

CACACCTCAACTGGGCATGTATATTTCTAGGCTTTCATAGTTTTGGCCTGTATATTCATA

ATGATACCATGAGCGCTTTGGGGCGTCCTCAAGATATGTTTTCAGATACCGCTATACAAT

TACAACCCATCTTTGCTCAATGGATACAAAACACTCATGCTTTAGCACCTGGTGCAACAG

CTCCTGGTGCAACAACAAGTACCAGTTTGACTTGGGGAGGTGGTGATTTAGTAGCAGTAG

GTGGAAAAGTAGCTTTGTTACCTATTCCACTAGGAACCGCAGATTTTTTGGTACACCACA

TTCATGCATTTACGATCCATGTGACCGTATTGATACTACTGAAAGGCGTTCTATTTGCTC

GTAGCTCCCGTTTGATACCTGATAAAGCAAATCTGGGTTTTCGTTTCCCTTGTGACGGAC

CCGGAAGAGGGGGAACATGTCAAGTATCCGCCTGGGATCATGTCTTCTTAGGTCTATTCT

GGATGTACAATGCGATTTCGGTAGTAATATTCCATTTCAGTTGGAAAATGCAGTCGGATG

TTTGGGGTAGTATAAGCAATCAAGGGGTGGTAACTCATATCACGGGAGGAAACTTTGCGC

AGAGTTCCATTACTATTAACGGATGGCTCCGGGATTTTCTATGGGCACAGGCCTCTCAGG

TGATCCAGTCTTATGGTTCTTCATTATCCGCATATGGCCTGTTTTTCCTAGGTGCTCATT

TTGTTTGGGCCTTCAGTTTAATGTTTCTATTCAGCGGCCGCGGTTATTGGCAAGAACTTA

TTGAATCCATCGTTTGGGCTCATAACAAATTAAAAGTTGCTCCTGCTACTCAGCCTAGAG

CCTTGAGCATTGTCCAAGGACGTGCTGTAGGAGTAACCCATTACCTTCTGGGTGGAATTG

CCACAACATGGGCATTCTTCTTAGCAAGAATTATTGCAGTAGGATAAATGGCATTAAGAT

TTCCAAGGTTTAGCCAAGGCTTAGCTCAGGACCCCACTACTCGTCGTATTTGGTTTGGTA

TTGCCACCGCACATGACTTCGAGAGTCATGATGATATCACTGAGGAAGGTCTTTATCAGA

ATATTTTTGCTTCTCACTTCGGGCAGTTAGCAATAATCTTCCTGTGGACTTCCGGGAATC

TGTTTCATGTGGCTTGGCAAGGAAATTTTGAGTCATGGGTACAGGACCCTTTACATGTAA

GACCTATTGCTCATGCAATTTGGGATCCTCATTTTGGTCAACCAGCTGTAGAAGCCTTTA

CTCGAGGGGGTGCTCTCGGTCCGGTGAATATCGCTTATTCCGGTCTCTATCAGTGGTGGT

ATACAATCGGCTTGCGCACCAATGAAGATCTTTATACTGGAGCTCTTTTTCTATTATT--

----TCTTTCTGCGATATCTTTAATAGCGGGTTGGTTACATCTACAACCCAAATGGAAGC

CAAGCGTTTCGTGGTTCAAAAATGCTGAATCTCGTCTTAATCATCATTTGTCAGGACTGT

TCGGAGTAAGTTCCTTGGCTTGGACAGGACATTTAGTTCATGTCGCTATTCCCGGATCC-

--AGGGGGGAATACGTCAGATGGAATAATTTCCTAGACGTATTACCATCTCCTCAAGGCT

TGGGGCCACTTTTTACAGGACAGTGGAATCTTTATGCCCAAAACCCCGATTCCAGTGGTC

ATTTATTCGGTACCTCCCAAGGAGCGGGAACTGCCATTCTAACCCTTCTCGGGGGATTCC

ATCCTCAAACTCAAAGCTTATGGCTGACCGATATTGCTCATCATCATTTAGCTATTGCAT

TTGTTTTTCTCGTTGCTGGT-CATATGTATAGAACTAACTTCGGGATCGGTCATAGTATA

AAAGATCTTTTAGAAGCACATACTCCTCCGGGGGGTCGATTGGGACGGGCACATAAAGGT

CTTTATGACACAATCAATAATTCCATTCATTTTCAATTAGGTCTTGCTCTAGCCTCTTTG

GGGGTTATTACTTCCTTGGTAGCTCAACACATGTATTCTTTACCTCCTTATGCATTCATA

GCACAAGACTTTACTACTCAAGCTGCGTTATATACTCATCACCAATACATCGCAGGCTTC

ATCATGACAGGGGCCTTTGCTCATGGAGCTATATTCTTCATTAGAGATTACAATCCGGAA

CAGAACGAGGATAATGTATTGGCAAGAATGTTAGACCATAAAGAAGCTATAAAATCTCAT

TTAAGTTGGGCCAGCTTGTTCCTGGGTTTCCATACTTTGGGACTCTATGTTCATAACGAC

GTCATGCTTGCTTTTGGTACTCCAGAAAAACAAATCTTGATCGAACCTATATTTGCCCAA

TGGATACAATCCGCTCATGGTAAGACTTCATATGGATTTGATGTACTCTTATCTTCAACG

AACGGCCCAGCATTCAATGCTGGTCGAAGCTTATGGTTACCTGGCTGGTTGAATGCTATT

AATGAGAATGGTAATTCACTATTCCTAACAATAGGTCCTGGAGACTTCTTGGTTCA-TCA

TGCTATTGCTCTAGGTTTGCATACAACTACATTGATCTTAGTAAAAGGTGCTTTAGATGC

ACGTGGTTCCAAGTTCATGCCAGATAAGAAAGATTTTGGTTATAGTTTCCCTTGCGATGG

TCCAGGACGAGGCGGTACTTGTGATATTTC-GGCTT--GGGATGCATTTTATTTGGCGGT

TTT-CTGGATGTTAAATACCATTGGATG-GGTTACTTTTT-ATTGGCATTGGAAGCATAT

CACTTTATGGCAGGGTAATCCTTCACAATTTAATGAATCTTCCACTTATTTGATGGGATG

GTTAAGAGATTATCTATGGTTAAACTCTTCACAACTTATCAATGGATATAACCCTTTCGG

TACAAATAGTTTATCTGTCTGGGCGTGGATGTTCCTATTTGGGCATCTGGTTTGGGCTAC

TGGATTCATGTTTTTAATTTCCTGGCGAGGATATTGGCAGGAATTGATTGAAACTCTAGC

ATGGGCTCATGAACGCACACCTTTGGCTAATTTGATTCGATGGAGGGATAAACCTGTGGC

TCTTTCCATTGTTCAAGCAAGACTGGTTGGCTTAGCCCACTTTTCTGTAGGTTATATATT

TACTTATGCAGCTTTCTTAATTGCCTCTACATCGGGCAAATTTGGTTAAATGTCACATTC

AGTAAAGATTTATGATACATGTATAGGGTGTACACAATGCGTACGAGCCTGCCCCACGGA

TGTATTGGAAATGATACCTTGGGACGGATGTAAAGCTAAACAAATAGCTTCTGCTCCAAG

AACAGAGGACTGTGTAGGTTGTAAGAGATGTGAATCCGCCTGTCCAACGGATTTCTTGAG

TGTACGTGTTTATTTATGGCATGAGACCACTCGTAGCATGGGTCTAGCTTATTGA-----

------------------------------------------------------------

------------------------------------------------------------

------------------------------------------------------------

---------------------------------------------------ATGCGAGAT

ATAAAAACATATCTCTCTGTGGCGCCCGTGTTAACTACTCTATGGTTCGGGGCTTTAGCA

GGTCTATTGATAGAGATTAATCGTTTCTTCCCAGATG------CGCTGGCATTCCCCTTT

TTT------------------TGA----CTGC-----TAGAGAGACGCGAAAGCACAAGC

TTATGGGGGCGCTTCTGCAACTGGATAACCAGCACCGAAAACCGTCTTTACATTGGGTGG

TTCGGTGTTTTGATGATCCCTACCTTATTGACCGCAACTTCTGTATTTATTATCGCCTTC

ATTGCGGCTCCTCCAGTAGATATTGATGGGATCCGTGAACCTGTTTCTGGTTCTCTACTT

TATGGAAACAATATTCTTTCTGGTGCCATTATTCCAACCTCTGCAGCTATAGGTTTGCAT

TTTTACCCAATATGGGAAGCGGCATCCGTTGATGAATGGTTATACAATGGTGGTCCTTAT

GAGTTAATTGTCCTACACTTCTTACTTAGTGTAGCTTGTTACATGGGTCGTGAGTGGGAA

CTTAGTTTCCGTCTGGGTATGCGCCCTTGGATTGCTGTTGCATATTCAGCTCCTGTTGCA

GCTGCTACTGCTGTTTTCTTGATCTACCCTATTGGTCAAGGAAGTTTCTCAGATGGTATG

CCTCTAGGAATATCTGGTATTTTCAACTTGATGATTGTATTCCAGGCGGAGCACAACATC

CTTATGCACCCATTTCACATGTTAGGCGTAGCTGGTGTATTCGGCGGCTCCCTATTCAGT

GCTATGCATGGTTCCTTGGTAACCTCTAGTTTGATCAGGGAAACCACTGAAAATGAGTCT

GCTAATGCAGGTTACAGATTCGGTCAAGAGGAAGAAACCTATAATATCGTAGCTGCTCAT

GGTTATTTTGGTCGATTGATCTTCCAATATGCTAGTTTCAACAATTCTCGTTCCTTACAT

TTCTTCCTAGCTGCTTGGCCCGTAGTAGGTATTTGGTTCACTGCTTTGGGTATTAGCACT

ATGGCTTTCAACCTAAATGGTTTCAATTTCAACCAATCCGTAGTTGACAGTCAAGGTCGT

GTCATCAACACTTGGGCTGATATAATCAACCGTGCTAACCTTGGTATGGAAGTTATGCAT

GAACGTAATGCTCACAATTTCCCTCTAGACTTAGCTGCTGTTGAAGCTCCATCTACAAAT

GGATAAATGGGTTTGCCTTGGTATCGTGTTCATACCGTTGTCTTGAATGATCCCGGTCGG

TTGCTTTCTGTCCATATAATGCATACAGCTCTAGTTTCTGGTTGGGCCGGTTCGATGGCT

CTCTATGAATTAGCTGTTTTTGATCCCTCTGATCCCATTCTTGATCCAATGTGGAGACAA

GGTATGTTCGTTATACCCTTCATGACCCGTTTAGGAATAACAAATTCATGGGGCGGGTGG

AGTATCACAGGAGGAACTGTAACGAATCCGGGTATTTGGAGTTACGAAGGTGTGGCCGGG

GCACATATTGTGTTTTCTGGTTTGTGCTTTTTGGCATCTATCTGGCATTGGGTGTATTGG

GATCTAGAAATATTCTGTGATGAACGCACAGGAAAACCCTCTTTGGATTTGCCCAAAATC

TTTGGAATTCATTTATTTCTCTCAGGGGTGGCTTGCTTTGGGTTTGGCGCATTTCATGTC

ACAGGTTTGTATGGTCCTGGAATATGGGTGTCTGATCCTTATGGACTAACGGGAAAAGTG

CAATCTGTAAATCCGGCATGGGGCGCTGAAGGTTTTGATCCTTTT-GTTCCGGGG--GGA

ATAGCTTCTCATCATATCGCAGCCGGGACATTGGGTATACTAGCAGGTCTATTCCATCTT

AGTGTCCGCCCACCCCAACGTCTCTACAAAGGATTACGTATGGGTAATATTGAAACTGTT

CTTTCCAGTAGTATCGCTGCTGTATTTTTTGCAGCTTTCATTGTTGCTGGAACTATGTGG

TACGGTTCAGCAACTACTCCGATTGAATTATTTGGTCCCACTCGTTATCAGTGGGATCAA

GGATACTTTCAGCAAGAAATATATCGAAGAGTTGGTACCGGGCTGGCCGAAAATCTAAGT

TTATCGGAAGCTTGGTCCAAAATTCCTGATAAACTAGCTTTTTATGATTACATCGGTAAT

AATCCGGCGAAAGGAGGATTATTCAGAGCAGGCTCAATGGACAACGGAGATGGAATAGCT

GTTGGGTGGTTAGGACACCCGATCTTTAGAGATAAGGAAGGGCATGAACTCTTTGTACGT

CGTATGCCTACTTTTTTTGAAACATTTCCAGTAGTTTTGGTAGATGGAGACGGAATTGTG

AGAGCTGATGTTCCTTTTAGAAGGGCAGAATCAAAGTACAGTGTCGAACAAGTAGGTGTA

ACTGTTGAGTTCTATGGTGGTGAACTCAATAGAGTAAGTTATAGCGATCCTGCTACTGTG

AAAAAATATGCTAGACGTGCCCAATTGGGTGAGATTTTTGAATTAGATCGAGCTACTTTG

AAATCCGATGGTGTTTTTCGTAGCAGTCCAAGGGGTTGGTTCACTTTTGGACATGCTTCG

TTTG-CTTTGCTCTTC--TTTTTCGGACACATTTGGCATGGTGCTAGAACCTTGTTTAGA

GATGTTTTTGCTGGTATCGACCCGGATTTGGATGCCCAAGTAGAATTTGGAGCATTCCAA

AAAATAGGAGATCCAACTACAAGGAGACAAATAGTCTGAATGAAAACCTTATATTCCCTG

AGGAGGTTCTACCCCGTGGAAACGCTCTTTAATGGAACTTTAGCTTTAGCTGGTCGTGAC

CAAGAAACCACAGGTTTTGCTTGGTGGGCCGGGAATGCCCGACTTATCAATTTGTCTGGT

AAATTACTCGGGGCTCACGTGGCCCATGCCGGATTAATCGTATTCTGGGCGGGAGCAATG

AATCTATTTGAAGTGGCTCATTTTGTACCAGAGAAACCTATGTATGAACAAGGATTAATT

TTACTTCCCCATCTAGCTACTCTAGGTTGGGGGGTAGGTCCTGGGGGGGA-AGTTATAGA

CACCTTTCCATACTTTGTATCTGGAGTACTTCATTTAATTTCCTCTGCAGTCCTAGGCTT

TGGTGGTATTTATCATGCACTTCTTGGACCTGAGACTCTTGAAGAATCCTTTCCATTCTT

CGGTTATGTATGGAAAGATAGAAATAAAATGACCACTATTTTGGGTATTCACTTAATTTT

ATTAGGTGTAGGCGCTTTTCTCCTAG---TGTTCAAGGCTCTTTATTTTGGAGGCGTATA

TGATACCTGGGCCCCCGGTGGCG---GAGATGTAAGAAAAATAACCAACCTGACACTTAG

CCCAAGTGTTATATTTGGTTATTTGCTAAAATCTCCTTTTGGGGGAGAAGGATGGATTGT

TAGTGTGGATGATTTAGAAGATATAATTGGAGGACACGTATGGTTAGGTTTCATTTGTAT

ACTTGGTGGAATATGGCATATCTTAACCAAACCTTTTGCGTGGGCTCGCCGCGCATTTGT

ATGGTCTGGAGAAGCTTACTTGTCTTATAGTTTGGGGGCTTTATCTATCTTTGGTTTCAT

TGCTTGCTGTTTCGTCTGGTTCAATAATACGGCTTATCCTAGTGAGTTTTACGGTCCTAC

CGGCCCAGAAGCTTCTCAAGCTCAAGCGTTTACTTTTCTAGTTAGAGACCAACGTCTTGG

GGCTAACGTAGGATCTGCTCAAGGACCCACCGGTTTAGGTAAATATCTAATGCGTTCTCC

AACTGGGGAGGTCATTTTTGGAGGAGAAACTATGCGCTTCTGGGATCTCCGTGCTCCCTG

GTTGGAACCACTAAGGGGTCCCAACGGTTTGGACTTGAGTAGGCTGAAAAAAGACATACA

GCCCTGGCAAGAACGACGTTCGGCGGAATATATGACTCATGCTCCTTTAGGTTCTTTAAA

TTCCGTGGGCGGCGTAGCTACAGAGATCAATGCAGTCAATTATGTCTCTCCCAGAAGTTG

GTTAGCTACCTCTCATTTTGTTCTAGGATTTTTCCTATTCGTGGGTCATTTGTGGCATGC

AGGAAGGGCCCGTGCAGCTGCAGCAGGATTTGAAAAAGGAATCGATCGTGATTTTGAACC

TGTTCTTTCCATGACTCCTCTTAACTGAATGACTATAGCCCTTGGTAGGTTTTCCAAAGA

GGAAAATGATTTATTTGATATTATGGATGACTGGCTACGGAGGGACCGCTTCGTTTTTGT

AGGTTGGTCTGGTCTATTGCTTTTTCCTTGTGCCTATTTTGCTTTAGGAGGGTGGTTCAC

AGGTACAACCTTTGTAACTTCATGGTACACCCATGGATTAGCTAGCTCCTATTTGGAAGG

ATGCAATTTTTTAACCGCTGCAGTTTCTACTCCTGCTAATAGTTTAGCACATTCTTTGTT

GTTACTGTGGGGTCCTGAAGCACAAGGAGATTTTACTCGTTGGTGTCAATTGGGGGGTCT

GTGGACTTTTGTTGCTCTCCATGGTGCTTTCGGACTAATAGGTTTCATGTTGCGTCAATT

CGAACTTGCTCGATCTGTTCAATTGCGACCTTATAACGCAATCGCATTCTCCGCTCCAAT

TGCTGTTTTTGTTTCTGTATTCCTGATTTATCCACTAGGTCAGTCTGGTTGGTTCTTTGC

ACCGAGTTTCGGTGTAGCAGCTATATTTCGATTCATTCTCTTCTTCCAAGGATTTCATAA

TTGGACGTTGAATCCATTTCATATGATGGGAGTTGCTGGAGTATTGGGTGCTGCTCTGCT

ATGCGCTATTCATGGTGCTACCGTAGAAAATACTTTATTTGAAGATGGTGATGGTGCAAA

TACATTCCGTGCTTTTAACCCAACTCAAGCTGAAGAGACTTATTCGATGGTCACTGCTAA

CCGCTTTTGGTCCCAAATCTTTGGGGTTGCTTTTTCCAATAAACGTTGGTTACATTTCTT

TATGTTATTTGTACCAGTAACCGGTTTATGGATGAGTGCTCTTGGAGTAGTCGGTCTGGC

TTTGAACCTACGTGCCTATGACTTC-GTTTCCCAAGAAATCCGTGCAGCGGAAGATCCTG

AATTTGAGACTTTCTATACCAAAAATATTCTCTTAAACGAAGGTATTCGTGCTTGGATGG

CGGCTCAGGATCAGCCCCATGAAAACCTTATATTCCCTGAGGAGGTTCTACCCCGTGGAA

ACGCTCTTTAAATGTCTGGAAGTACGGGAGAACGTTCTTTTGCTGATATTATTACCAGTA

TTCGATACTGGGTCATTCATAGCATTACTATACCTTCCCTATTCATTGCAGGTTGGTTAT

TCGTCAGCACAGGCTTAGCTTACGATGTGTTTGGAAGCCCTCGACCGAACGAGTATTTCA

CAGAGAGCCGACAAGGGATTCCATTAATAACTGGCCGTTTCGATTCATTGGAACAACTTG

ATGAATTTAGTAGATCCTTTTAGATGACCATAGATAGAACCTATCCGATTTTTACAGTGA

GATGGTTGGCTGTTCACGGACTAGCTGTACCTACAGTTTTTTTCTTGGGGTCAATATCAG

CAATGCAGTTCATCCAACGATAA---------ATGGTTACACAAAGCGTGGAGGATAGTT

CCAGATCTG------GTCCAAGACGGACTATTGTAGGGGATTTATTGAAACCGTTGAATT

CAGAATATGGTAAAGTAGCTCCTGGATGGGGAACTACCCCCTTTATGGGGGTCGCAATGG

CTCTATTTGCAATATTTCTATCAATTATTTTGGAGATTTATAATTCTTCCGTTTTACTGG

ATGGAATCTCAGTGAGT------TAG------------------ATG-----------CT

TACTCTCAAACTGTTCGTTTACACAGTAGTGATATTCTTT---------------GTTTC

TCTATTCATTTTTGGATTCCTATCTAATGACCCAGGACGTAATCCTGGGCGTGACGAA--

----TAAATGCGAGATATAAAAACATATCTCTCTGTGGCGCCCGTG---TTAACTACTCT

ATGGTT------CGGGGCTTT-AGCAGGTCT-----ATTGATAGAGATTAATCGTTTCTT

CCCAGATG------CGCTGGCATTCCCCTTTTTTTGA------------------ATGCC

TAATA---TCTTCAGTTTGATCTGTATCTGTCTTCATTCTGCCC---TTCATTCGAGTG-

-----GTTTTTTCTTCGCGAAACTGCCCGAGGCCTATGCTGTTTTCAATCCAATCGTAGA

TGTTATGCCGGTCATACCTTTGTTCTTTTTTCTCTTAGCCTTTGTTTGGCAAGCTGCTGC

AAGTTTTCGATGAACGACACAAT---------CAAACCCAAACGAACAAAATGTTGAATT

GAATCGTACCAGTCTCTACTGGGGGTTATTACTCA-TTTTTGTACTTGCTGTTTCATTTT

CCAATTATTTCTTCAATTGA----------------------------------------

------------------------------------------------------------

--------------ATGGAAACTGCAACCCTAGTTGCCATCTCCATATCTGGTTCACTTG

TAAGCTTTACTGGGTATGCCTTATATACCGCCTTTGGGCAACCCTCTCAACAACTAAGAG

ATCCATTCGAGGAACATGGGGACTAA---------ATGGAAGCACTGGTTTATACATTCC

TGTTGGTATCGACTCTAGGGATAATTTTTTTCGCTATCTTTTTTCGAGAACCGCCTAAGG

T---------TCCGAATAAAAAAATAAAATGAATGACTATTGCTTTCCAATTGGCTGTTT

TTGCATTAATTGCTACTTCATCAATCTTACTGATTAGTGTACCCGTTGTATTTGCTTCTT

CTGATGGTTGGTCAAGTAACAAAAATATTGTATTTTCGGGTACATCATTATGGATTGGAT

TAGTCTTTTTGGTAGCTATCCTTAATTCTCTCATCTCTTGA-------------------

--ATGTCACCAAAAACAGAGACTAAAGCAAGCGCTGGATTCAAAGCCGGTGTTAAAGATT

ACAGATTGACTTATTATACTCCTGACTATGAAACCCTAGCTACTGATATCTTGGCAGCAT

TCCGAGTAACTCCTCAACCTGGAGTTCCACCTGAGGAAGCAGGAGCTGCAGTAGCTGCCG

AATCTTCCACTGGTACATGGACAACTGTGTGGACCGATGGACTTACCAGCCTTGATCGTT

ACAAAGGTCGATGCTACCACATCGAACCCGTTGCTGGGGAAGAAAATCAATATATTGCTT

ATGTAGCTTATCCTTTGGACCTTTTTGAGGAAGGTTCTGTTACTAACATGTTTACTTCCA

TCGTAGGTAATGTATTTGGGTTCAAAGCCCTACGAGCTCTACGTCTAGAGGATCTGCGAA

TTCCTCCTGCTTATTCCAAAAGTTTCCAAGGCCCACCTCATGGCATCCAAGTCGAGAGAG

ATAAATTGAACAAGTATGGTCGTCCCCTATTAGGATGTACTATTAAACCAAAATTGGGGT

TATCTGCCAAAAACTACGGTAGAGCGGTTTATGAATGTCTCCGTGGTGGGCTTGATTTTA

CCAAGGATGATGAGAATGTGAACTCCCAACCATTTATGCGTTGGAGGGACCGTTTCTTAT

TTTGTGCCGAAGCTCTTTATAAAGCACAGGCAGAAACAGGTGAAATCAAAGGACATTACT

TGAATGCTACTGCAGGTACATGCGAAGAAATGATGAAAAGGGCCGTATTTGCCAGAGAAT

TGGGAGTTCCTATCGTAATGCATGACTACTTAACAGGAGGATTCACTGCAAATACTAGTT

TGGCCCATTATTGCCGAGACAATGGCCTACTTCTTCACATTCATCGCGCAATGCATGCAG

TTATTGATAGACAAAGGAATCATGGTATACACTTTCGTGTACTAGCTAAAGCATTGCGTA

TGTCTGGTGGGGATCATATTCACGCTGGTACCGTAGTAGGTAAACTGGAAGGGGAACGGG

ATGTCACTTTGGGTTTTGTTGATCTACTACGTGATGATTTTATTGAAAAAGACCGAAGTC

GCGGTATTTATTTCACTCAAGATTGGGTATCTATGCCAGGTGTTCTGCCCGTGGCTTCAG

GTGGTATTCACGTTTGGCATATGCCTGCCCTGACCGAGATCTTTGGGGATGATTCCGTAT

TACAGTTCGGCGGAGGAACTCTGGGACACCCTTGGGGAAATGCACCCGGTGCAGTAGCTA

ATCGGGTGGCTTTAGAAGCGTGCGTACAAGCTCGTAATGAGGGACGTGATCTTGCTCGTG

AAGGTAATGAAGTTATCCGT-----------------------GAAGCTAGCA-------

-------GGTGGAGCCCAGAACTAGCTGCTGCTTGTGAGGTATGGAAGGAGATCAAATTC

GA---ATTCGAAGCAATGGATGTCTTG---------------TAAACGGCGATACATTTA

TACAAAACTTCTACCCCGAGCACACGCAATGGAGCCGTA---------GACAGTCAAGTG

AAATCCAATCCACGAAAGAATTTGATCTATGGACAGCATCGTTGTGGTAAAGGTCGTAAT

GCCAGAGGAATCATTACCGCAGGGCATAGAGGGGGGGGTCAT-AAGCGTCTATACCGTAA

AATCGATTTTCGACGGAATGAAAAAGACATATCTGGTAGAATCGTAACCATAGAATACGA

CCCTAATCGAAATGCATACATTTGTCTCATACACTATGGGGATGGTGAGAAGAGATATAT

TTTACATCCCAGAGGGGCTATAATTGGAGATACCATTGTTTCTGGTACAGAAGTTCCTAT

ATCAATGGGAAATGCCCTACCTTTGAGTGCGGTTTGAACCATTGATTTACGTAATTGGAA

GTAACCAATTAGGTTTACGACGAAACCTAGAAATCGATCACTGATCCAATTTGAG-----

---CGGGATAGACCTCAACAGAAAACTGAAGAGTAACGGCAGCAGGTGATTGAGTTCAGT

GGTTCCTCATATAAAATGATTGACTCTAGAGATATGGTAATATGGAGAAGAC-----TAA

----ATTGTTTGAAGCACGGACAGAACCGGA-AGCGCCCCTTGTTTCAAAGAGAGGGGGA

CGGGTTATTCACATTTCATTTGATGGTCAGAGGCAAATTGAAAGCTAAGCAATGGTAATT

CT-------------AAAGATCCCCCGGGGGA-----AG---------------------

----------AGATGTCTCCTACGTTACCCGTAATATG-----TGGAAGTGTCGACGTAA

TTTCATAGAGTCATTCGGTC--TGAGTGCTACATGAAGAACATAAGCCAGATGACGGAAT

GGGGAGACCTAGGATGTATAAAATCGTAGGATGAGTGATTCGACAGATTTGGATTC----

-CTATATATCTACTCGTGTGGTACTTCATC---ATACGATTCATATAAGATCCACCTGTC

TAGATATCATCATATACATCCAGAAAGCCGTATGCTTTGGAAGAAGCTTGTACAGTTTGG

GAAGGGGTTTTTATTGATCAAAAAGAAGAATCTACTTCAACCGATATGCCCTTAGGCACG

GCCATACATAATATAGAAATAACACTTGGAAAGGGTGGACAATTAGCTAGAGCAGCAGGT

GCTGTAGCGAAACTGATTGCAAAAGAGGGTAAATCGGCCACATTAAGATTACCATCTGGG

GAGGTTCGTTTGATATCCAAAAACTGCTCAGCAACAGTCGGACAAGTGGGTAATGTTGGG

GTGAACCAGAAAATTTTGGGTAGAGCCGGATCTAAATGTTGGCTAGGTAAGCGTCCTGTA

GTAAGAGGAGTAGTTATGAACCCTGTAGACCATCCCCATGGGGGTGGTGAAGGGAGGGCC

CCAATTGGTAGAAAAAAACCCACAACCCCTTGGGGTTATCCCGCGCTTGGAAGAAGAAGT

AGGAAAAAGAAGAAATATAGTGATAGTTTCATTCTTCGTCGTCG--TAAG----------

------TAAATGATTCAAGCTCAAACCTATTTGAATGTAGCGGACAACAGCGGGGCTCGA

GAATTGATGTGTATTCGAATCGTAGGA---GCTAGTAACCCCCGATATGCTCATATTGGT

GACGTTATTGTTGCTGTAATCAAAGAAGCAGTACCCAATATGCCCCTGGAAAGATCAGAA

GTGATCAGAGCTGTAATTGTACGTACATGTAAAGAACTCAAACGTGACAACGGTATAATA

ATACGATATGATGACAATGCAGCAGTTGTTATTGATCCAGAGGGAAATCCAAAAGGAACT

CGAGTCTTTGGTTCGATCGCTGGGGAATTGAGACATTTGAATTTCACTAAGATAGTATCA

TTAGCTCCCGAGGTATTCTAAATGCTTAGTGTGTGACTCGTTGAGATCCGGTTTTTTTTG

GAGTTGGGACTAAAATAAAATTATGGAATATAGAAA----------------GACCGACT

CCTAGTATGAA-------------------------------------------------

-----------------------------------CTAATAACCAACTCATTGCTTCG--

---TATTCTTAAGATCCAAAGAAGCATTCATTCACTATATGATTAT--------------

----------------------------------------CTATTTTATAGTTGCAGCAA

TGGAAATCCTTTCTAT---------GTACAAATAAATCACTATTGATTGTG--CATTGTT

AAACGAAAATAGA-GGGATATG--GATA-AAAGGAAGAATGAGAGAAAGAGAAGAAGA--

-----------------ACGATATATTATTCCAA--TA--TGTATGGTCTATGAATCATC

TCACATAAAGAAAGGCAGTGTGACAAAGCATCA-ATAC----------------------

---------------------------------------CGATTGGTCGATAAAAATATT

AAAAAT------------------------------------------------------

------------------TTAAATAAAATAATTAGATTAATTTGGTTCCTAGGAGAAATA

A---TAAAGAGCATT-GAGTTGATACAGACTGAGAAGATTGACTCGGGAACA--------

----AATTGAAAGCTCCATTGCAAAGGCAAGGCCTAGACA-TTAAT---GGAGAAGCTAT

GGGAACGACGGAACCAGTGACTGCATAAGATTCTATTGAA----AACGAATCCTACTGAT

TCATTGGATGGGATGGCGGAACAAACCAAG-AACCCTTTTCTTTTTTCTGAGAGGT-CAT

AGGTTGACCTGAACCTATGAA-TGAAGCAGATATT---GAAAGAGTAAATATTCGCCCGC

GAAATCCTTGTTGG------------ATTGGAAAATGTTGGAC-----------AATTCG

GATTCGGTAGGATTCTTTCTTTATTAATTA---------------------TAAGAATTC

TATTT--------------------------------------------TCTTATACTTA

ATAATATAAAAATTAAATTCTA----AGTTAATATAAGAATTAAATG-------------

------------AAATTAGAAGTACGTATATCTATAGATAATGTTATCTTTTAGAATAGA

ATCCTTTTTAGAATTCT---------------------ATATTCTATTATTCTATCTATA

TAGAAATAGAACAGATATCTGAATCTAATT------------------------------

------------------------------------------------------------

---CGTACGTTAGAAATTTAATCTGAATGAATCCGATGATTCAATGTATTATTCAT----

TAAATACATGTATATATAGATTGAA-----------------------------------

------------------------------------------------------------

-----------TCGTTTA-ATTCGCAAGGAGCTGGATGAGAAAAAACTCTCATGTCCGGT

TCTGCAGTAGAGATGG-----GATTAAAGC--ACTACCATCAACTATAACCCTAAAAGAA

CCAGATTCCGTAAACAACATAGAGGAAGAATGAAGGGAGTATCTTCT---CGGGGCAATC

ATATTTGTTTCGGTAGATACGCTCTTCAGGCACTTGAACCTGCTTGGATCACATCTAGAC

AAATAGAAGCGGGTAGACGAGCAATGACACGATACGCTCGTCGCGGCGGAAAAATATGGG

TACGCGTATTCCCCGACAAACCCATTACAGTAAGACCCGCGGAAACACGTATGGGTTCGG

GAAAGGGATCTCCTGAATATTGGGTTTCTGTAATTAAACCGGGTCGAATACTTCATGAAA

TGGGCGGAGTGCCAGAAACTGTCGCCAGAGCAGCTATGGAAATAGCCGCGTGCAAAATGC

CTATACGAACTCAATTCATTA---GTGCGAAA------TAGATGACCAGAGTTAGACGCG

GATATATAGCTCGCAGACGTCGAACAAAAATTCGTTTATTTGCATCGACCTTTCGAGGGG

CCCATTCAAGACTTACTCGAACTGCTACTCAACAGAAAATGAGGGCTTTGGTTTCCATTT

ATCGGGATAGAGGTAGGCAAAAGAGAGAATTTCGTCGCTTGTGGATCGCTCGGATAAATG

CAGCAACTCACGAAAATGG---GGTATCC------TATAGTCGATTAATAAACGATCTGT

ACAAGAGGCAGTTGCTTCTTAATCGTAAAATACCTGCACAAATAGCTAGATACAATCTAA

ATTCCCTTTACATGATTTCCAATGA------A-ATCATA-AAAGAAAAAAGGGATTAA--

---------ATGATAAATAAAA-----------------TCTCAGGTACAGAAA------

---------TACGAG---------------------------------CATTAGCTA---

-------------------AACATATATGTATGTCCCCTCACAAAGCGCGAAGAGTTATT

GATCAGATTCGCGGTTGCTCTTATGAACAAACACTTATGATACTAGAACTCATGCCGTAT

CGAGTATGTTATCCCGTTTTCAAATTGGTTTACTCTGCAGCGGCAAATGCTAGTCACAAT

ATGGGTTTGAACGAAGCTGATTCATTCATTAGTAAAGCGGAAGTCCATGGAGGTGCCATG

ATGAAAAAGTTCAAACCTAAAGCTAGGGGACGCAGTTATCCGATAAAAAGACCCACTTGT

CATATAACTATTGTTTTAAAGG-------AAAGAA-----------AAAAGAGT------

---------------------CCCCTTGTAGTTTGA---------------ATGGATGGA

ATCAAATATGCAGTATTTACAGAAAAAAGTATTCGGTTATTGGGGAACAATCAATATACT

TCTAATGTCGAATCAGGATCAACTAGGGCAGAAATAAAGCATTGGATCGAACTCTTCTTT

GGTGTCAAGGTAATAGCTATGAATAGTCATCGACTCCCGGTCCCGGGAAAGGGTAGAAGA

ATGGGACCTCTTATGGGACATAGAATGCATTACAGACGTATGATCATTACGCTTCAACCG

GGTTATTCTATTCCACCTCT-TATAGAGAAAAGGACTTAAATGGCAGTCCCAAAGAAACG

TACTTCTATGTCAAAAAAGCATATTCGTAGAAATTATTGGAA--GAGAAAGGGATATAAA

G-CCGCGGTAAAAGCTTTTTCT-------TTAGGTAAATCTG------TTTCTACCGG--

----------GCATTCAAAAAGTTTTT-TTGTGCAACAAACAA---CAACAAAAACAAAC

AAA------------------------------------------TAAATGGCCAAAGGT

AAAGATGCTAGAGTAAGAGTTATTTTGGAATGTACCA---GTTGTACTCG------AAAT

GGTGCTAATGCTAAGAAGGAATCACCGGGCATTTCCAGATATATTACTGAAAAGAATCGA

CACAATACACCTAGTCGATTGGAATTGAAGAAATTCTGTCCTTATTGTTCGAAGCATATG

ATTCACGCGGAGATAAAGAAA------TAGATGAAAATAAGAGCCTCTGTTCGTAAGATT

TGTGAAAAATGTCGACTGATTCGTAGGCGAGGACGAATTATAGTAATTTGTTCCAATCCG

AGACATAAACAGAGACAGGGGTAAATGGTTCGAAAGGAAGTACCAGTATCCAATCGGACA

CTACAGTGGAAGTGTGTTGAATCAAAAGCAGACAGTAAGCGTCTTTATTATGGTCGTTTC

G-TTCTATCT--CCGCTTATGAAAGGTCAAGCTG-ATACGATA-GGTACC-GCGATGCGA

AGGT--CTTTACTTGGAG-AAATAGAAGGAA--CATGTATC----ACATGTGCAAAATCT

----GAGAGGGTACCACATGAATATTC--------TACAATAGTAGGTATTGAGGAATCA

GTACATGAAATTTTAATGAATTTGAAAGAAATTATAGTTAGAAGCGATCTGTACGGGACT

CGTGATGCATTTATCTGTGTTAGGGGTCCTAAATATGTAACTGCTCAAGATATAATCTCA

CCACCTTCTGTGAAAATAGTTGATACTACACAGCATATAGCTAGTATCACCGAACCAATT

AATTTTTGTATTGGATTAGAAATCGAGAGAAATCGCGGATATCGTACGAGAACCACCAA-

-----CCAAGATGCAAGTTA----------TCCTATAGATGCTGTATCTATGCCTGTTCG

AAATGCGAATTATAGTATTCAATGTTATGGAAATGAGAATG------AAAAACAAGAGAT

ACTTTTTCTAGAGATATGGACAAATGGAAGTTTAACTCCTAAAGAGGCACTTTACGAAGC

TTCCCGTAATTTGATCGATTTATTTATTCCTTTTCTACATGCGGAGGAGCGGGA---CCT

------CCATTTAGATGACGATCAAAACATATT---TACTGCGCCTTTTTTCCCCTTTCA

TGAGGGGTTGGCTTATATAGGGATAGGGGAAAGCAAAAAAAGAATTGCATTGAAACGTAT

TTTTATTGACCAATTAGAATTATCTCCCA-GGACCTATAACTGCCTCAAAAGGTCCAATA

TCCATACATTATTGGACCTTTTGAGTAAGAG------TCAAGAAGATCTTAGGAAAATTC

AACATTTTCGTGTAGAGGATGTCAAACGGGTATTGGACATTCTC-AAGAAACGTTTTGCA

ATGAATTTATTTA--CCTAA----------------------------------------

---------------------------------------------------ATGCTACGG

GA---TGGAAATGAGGGAATGTCTACAATACCTGGATTTAGTCAGATACAATTTGAAGGA

TTTTGTAGGTTCGTTGATCAGGGCTTGGCAGAAGAACTTCATAAGTTTCCAAAAATTGAA

GATACAGATCAAGAAATTGAATTTCAATTACTTGTGGAAACATATCAATTGGCGGAACCC

TTGATAAAAGAAAGAGATGCTGTGTATGAATCACTCACACATTCCTCTGAATTATATGTA

CCCGCGGGGTTAATTTGGAAAGTTGGTAGAGATATGCAAG--AACAAACCGTGTTTATTG

GAAACATTCCTCTAATGAATTCTCTGGGAACCTTTATAGTAAATGGAATATACAGAATTG

TGATCAATCAAATATTGCAAAGCCCCGGTATTTATTACAGTTCAGAATTGGACCATAACG

GGAT---TTCTGTTTATACCGGCACCATAATATCAGATCGGGGAGGAAGATCAGAATTAG

AGATTGATAGAAAAGCAAGGATATGGGCCCGTGTGAGCAGGAAACAAAAAATTTCTATTC

TAGTTCTACCATCAGCTATGGGTTCGAATCTAAGAGAAATTCTAGACAATGTTTGCTACC

CCGAAATTTTA------------TTGTATTTCCCGAATGAGAAAGAGAAA-------AAG

AAAA-----TTGGGTCGA-AGGAAAATGCTATTTTGGAGTTTTATCAACAATTTTCTT--

-----GTGTA--GGCGGGGATCCGGTATTTTCTGAGTCCCTCTGTAAGGAATTACAAAAG

AGATTTTTTCAACAAAGATGTGAATTAGGAAGGATTGGTCGACAAAATATGAACCAGAGA

TTGAATATTGATATACCCCAGAACAATACATTTTTATTACCACGAGACGTATTGGCTGCT

ACAGATCATTTGATCGGAATGAAATTTGGAATGGGTACACT------TGACGATATGAAT

CACTTGAAAAATAAACGTATTCGTTCTGTAGCAGATCTGTTACAGGATCAATTCGGATTG

GCTCTGGTTCGTTTGGAAAATGTGGTTCGAGGAACTATATGTGGAGCA------------

---ATCAGGCATAAATTTATACCGACTCCTCAAAACTTGGTAACTTCAACTCC-------

-----ATTAACAACCACTTATGAATCGTTTTTCGGTCTACACCCTTTATCTCAAGTTTTG

GATCGAACTAATCCATTGACACAGATAGTTCATGGGCGAAAATCAAGCTATTTGGGTCCC

GGAGGATTAACAGGGCGAACTGCTAGTTTTCGCATACGAGATATCCATCCTAGTCACTAT

GGACGTATTTGCCCAATTGACACGTCCGAAGGAATCAATGTTGGACTTATCGGATCCTTA

GCTATTCATGCAAGGATCGGCGAT---TGGGGATCTATACGAAGTCCGTTTTATGAAATA

T---CTGAGAGATC------AAAAGAGGA---ACAGATGGTTTATTTATCACC------A

AGGAGAGATGAATACTATATGGTAATGGTAGCAGCAGGAAATTCTTTGGCCTTGAATCAG

---GATATTCAAGACGAACAGGTCGTTCCAGCCCGATACCGTCAAGAATTCGTGACTATT

GCATGGGAACATATTGATCTTCGAAGCATTTATCCCCTCCAATATTTTTCTATTGGAGCT

TCCCTCATTCCGTTTATCGAACACAATGATGCGAATCGAGCTTTAATGAGTTCTAATATG

CAGCGTCAAGCAGTTCCGCTTTCTCGGTCCGAGAAATGCATTGTTGGAACTGGGTTGGAG

CGCCAAGCGGCTTTAGATTCGGGGGGTTCAGCTATAGCCCAACACGAGGGAAAGGTCATT

TATACCGATACTGAAAAGATCCTTTTATCAGGGAATGGAGACACTATAAGCATTCCATTG

CTTATGTATCAGCGTTCCAACAAAAATACTTGTATGCATCAAAAACCTCAGGTTCATCGG

GATAAATATGTTAAAAAGGGACAAGTCTTGGCGGATGGTGCGGCTACAGTTGGTGGGGAA

CTCGCTTTGGGAAAAAACGTATTAGTAGCTCATATGCCATGGGAAGGCTACAATTTTGAA

GATGCAGTACTAATTAGCGAACGTCTGGTATATGGAGATATTTATACTTCGTTTCACATC

CGGAAATATGAGATTCAGACTCATGTGACAAGCCACGGCC---CTGAAAAGATCACTAAC

GAAATACCTCATTTAGAAGCTCATTTGCTCCGTAATTTAGACAGAAATGGAATTGTGATG

CTCGGATCTTGGGTGGAAACGGGTGATGTTTTAGTAGGTAAATTGACGCCTCAGACAGCG

AAAGAATCATCGTATGCCCCGGAAGATAGATTATTACGAGTCATACTTGGAATTCAAGTA

TCCACTGCAAAGGAAACTTGTCTAAAACTACCTATAGGTGGAAGAGGTCGAGTTATTGAT

GTGAGATGGATCCA----------------------------GAAAAAA-----------

---------------------GGGGCTTCCAGTTA-------------------------

--------------------TAATCCAGAAAAAATTCGTGTATATATTTCACAGAAACGT

GAAATCAAAGTGGGTGATAAAGTCGCCGGAAGACATGGCAATAAGGGTATCATTTCCAAA

ATTTTGCCTAGACAAGATATGCCTTATTTGCAAGATGGAACACCAGTTGATATGGTCTTC

AACCCATTAGGAGTACCTTCACGAATGAATGTGGGACAGATCTTTGAATGCTCGCTGGGG

TTAGCGGGAGATCTGCTGGACAGACATTATAGAATAACACCCTTTGATGAGAGATATGAG

CAAGAGGCTTCGAGAAAACTAGTGTTTCCTGAATTATATGAAGCCAGTAAGCGAACAGCA

AATCCATGGGTATTTGAACCCGAGTATCCTGGAAAAAGCAGAATATTTGATGGAAGAACA

GGAGATCCTTTTGAACAACCTGTTATAATAGGAAAGTCCTATATGCTGAAATTAATTCAT

CAAGTAGATGATAAAATCCATGGACGTTCCAGTGGACATTATGCACTTGTTACACAACAA

CCCCTTAGAGGAAGGGCCAAGCAAGGGGGACAACGAGTAGGAGAAATGGAAGTCTGGGCT

CTAGAGGGATTTGGTGTTGCTCATATTTTACAAGAGATGCTTACTTATAAATCCGACCAT

ATTAGAGCTCGTCAGGAACTACTTGGTACCACGATCGTTGGAGGAACAATACCTAAGCCT

GAGGGTGCTCCAGAATCTTTTCGATTGCTCGTTCGAGAACTACGATCTTTGGCTCTGGAA

CTGAAGCATTTCCTTGTATCTGAGAAGAACTTCCAGATTAATAGGAAGGAAGCT------

---TGA---------------------ATGATCGACC---GATATAAACATCAACAACTT

CGAATTGGATTAGTTTCTCCTCAACAAATAACTGCTTGGGCCAACAAAATCCTACCTAAT

GGAGAGATGGTTGGAGAAGTGACAAAACCCTATACTTTTCATTACAAAAGCAATAAACCG

GAAAAAGATGGATTGTTTTGTGAAAGAATCTTTGGGCCTATCAAAAGTGGAATTTGTGCT

TGTGGAAATTA-----------------------CCGAGTCATCGGGGATGAAAAAG---

-AAGACCCGAAATTTTGTGAACAATGCGGAGTCGAATCTGTTGATTCTCGGATACGAAGA

TATCAAATGGGATACATCAAACTGGCATGTCCAGTGACTCATGTGTGGTATTTGAAACGT

CTTCCCAGTTATATCGCGAATCTTTCAGATAGACCTCTTAAAGAATTAGA------AGGC

CTGGTATACTGCGATGTGTGATTTGATCA-AAATTACAATTCTACAGGTTCAGAATGAAA

AACTGTCATCCCGTTCAATCCAATT---GGGATGCCTCCAGCTCTGACATGTCTCTTAGG

AGAAGTAACATGAAGCTCAGAATTATGGGTCTATTCAATACTTCC-----AAATGAAAGA

GGAATTGATCCATGGTCGAT---T-CCGTAGCGGATCAATCA-ATAGGGAATTCTAGTTG

TACCTCGTA---------------AAAAAAGCGTCTTTCGTGAAACTAGAACCAGCCATT

GTATTTCTTTT---------AGTTTAGAAAGAGGTTCTGTTCAAATAAGCAGA-------

---TATTACATGGTTACAGGAGTCTATCCATCGCATATATGCTTCACTTCAAGGGACACC

ATG-GCATA-------ACCGTCGAGGCGAAGTAGGG---------ACCT--AAAAGATCG

AATGGAATGATACATAG-----ACAAGTAAATCCCTTATGAATTCCAAAGTCTTCTTTTA

ATTTAAGAGAATTCATCATTCGAAGGGAAGGAGACTACTCAATA-----ATTCCACTTT-

-------------------------GTCGTAATTGAATGAGTCATTGAGGA---------

-------GAAGAGGTGAATCA-ATGAAATATGGATTGGCC----CTCACCGAG--AACTT

GAGTAAGGAGTAG----------------------TTTTGGGATTTATCTAGAGTAAAAT

TTGGGAGT----GAGAACCCC-----TTTATTTGGTACAACTACTTGAGCCGGATGAGAG

GAAACCTTCACGTCCGATTTTGAGGGGGGG-------ATTCTATAGGAACTTATCCCAAT

TTCTCTTTTGCTAGGCCCATAGCTAAAAAACCCACTTTCTTACGATTACGAGGTTCATTC

GAATATGAAA---TCCAATCCCGGAAATACAGCATCCCACTTTTTTTTAC----------

-----------TACCCAATGCTTCAATCTATTTAGAAATAGAGAAATATCTACTGGAGCA

GGTGCTATCAGAGAACAATTAGCCGACCCGGATTTGCGAATTATTACAGATCGTTCATTG

GTAGAATGGAAGGAATT---------------------------GGGGGAAGAAAGATCC

------GCTGAGAATGAATGGGAAGATAAAAAAATCGTAAGAAGAAAGGATTTTTTGGTT

AGACGCATGGAATTGGCTAAGCATCTTCTTAGAACAAATGTAGAACCAGAACGGATGGTT

TTGTGCCTATTACCAGTTCTTCCTCCCGAGTTGAGGCCAATCATTCAGATAGATGGGGGT

AAACCAATGAGT---TCGGATATTAATGAACTCTATAGAAGAGTTATCTATCGGAATAAT

ACTCTTATTGATCCATTAACAA--CAAGTAGATCTACGCC-------------AGGGGAA

TCAGTGATGTGTCAGGAAAAATTAGTACAAGAAGCCGTAGATACACTTCTTGATAATGGG

ATCCGCGGACAACCAATGAGGGACGGTCACAATAAAGTTTACAAGTCTTTTTCAGATGTA

ATTGAAGGCAAAGAGGGAAGATTTCGCGAGACTCTACTTGGTAAACGGGTCGATTATTCG

GGTCGTTCTGTCATTGTGGTGGGCCCTTCGCTTTCATTACATCGATGTGGATTACCCCGA

GAAATAGCAATAGAGCTCTTTCAGACATTTGTAATTCGTGGTCTAATCAGACAACATGTT

GCTTCCAACATAGGGATTGCTAAAAGTAAAATTCGGGAAAAAGAACCAATTGTATGGGAA

ATACTTCAAAAGGTTATGGAAGGACATCCTGTATTGTTGAATAGAGCACCTACTTTGCAT

AGATTAGGCATACAGGCGTTCCAACCTATTCTAGTGGAGGGACGTGCTATTTGTTTACAT

CCATTAGTTCGTAAGGGATTCAATGCAGACTTCGATGGGGATCAGATGGCTGTTCATGTA

CCTTTGTCTTTGGAAGCTCAAGCGGAAGCCCGTTTACTTATGTTTTCTCATATGAATCTC

TTGTCTCCGGCTATTGGAGATCCTATTTCTGTACCAACTCAAGATATGCTTATTGGCCTC

TATGTACTAACGATTGGGAATCGCCGAGGTATTTGTACAAATAGGTATAATCCA------

TGTAACTACAGAAACTATCAAAATGAAATAGTTGACGATAATA---------ACTATAAG

------TATACAAAAGAGAAAGAACCTTATTTTTGTAGTTCCTACGATGCGCTTGGAGCT

TATCGTCAGAAACGAATCGACTTATACAGTCCTTTGTGGCTCCGGTGGCGGC---TAGAT

CAATGCGTGATTG------CCTCAATAAATAGAGAAGTTCCCATTGAAGTTCAATATGAA

TCTTTGGGTATCTACCATGAGATTCACGAGCACTATCGAATAGTAAAAAGCGTAAAAAAA

GAAATCGTTTGTATATACATTCGAACCACTGTTGGTCATATTTCTTTTTATCGAGAAATA

GAAGAAGC----------TATACAAGGGTTTTGTCGGA--CCTATTGA------------

----------------------------------------------ATG--GCAGAACGG

GC---------------CGATCTGGTCTTTCACAATAAAGCGATAGATGGAACTGCCATG

AAACGACTTATTAGTAGATTAATAGATCATTTTGGAATGGCATATACATCACACATTCTG

GATCAAGTAAAGACTCTGGGTTTCCGGCAAGCCACTCTGACATCAATTTCATTAGGAATT

GATGATCTTTTAACAACGCCTTCTAAGGGATGGTTAGTACAAGATGCGGAACAACAAAGT

TTGATTTTGGAAAAGCACCATCATTATGGGAATGTACACGCAGTAGAAAAATTACGCCAA

TCAATTGAGATATGGTATGCTACAAGTGAATATTTGAGACAAGAAATGAATCCAAATTTC

AGGATGACTGATCCTTTTAATCCAGTCTATATAATGTCCTTTTCGGGAGCTAGAGGAAAT

GCATCTCAGGTCCACCAATTAGTAGGTATGAGAGGATTAATGTCGGATCCCCAAGGACAA

ATGATTGATTTGCCCATTCAAAGCAATTTACGCGAAGGGCTTTCTTTAACGGAATATACC

ATTTCCTGTTACGGAGCCCGCAAAGGAGTTGTGGATACTGCTGTACGAACATCAGATGCT

GGATACCTCACGCGTAGACTTGTTGAAGTAGTTCAACACATTGTTGTACGTAGAACGGAC

TGTGGCACTATCCGAGGTATTTCCGTAAGTCC---------TCGAAATGGGGTTGGGGTG

ACGGAAAGGATTTTTATCCAAAC------ACTAATTGGTCGCGTATTAGCGAACGATGTA

TATATGGGTCTGCGATGCATTGCGACTCGAAATCAAGATATTGGGATTGGACTTGTCAAT

CGATTCATAACC------------------TCTCGGGCACAACCAATATATATTCGAAGC

CCCTTCACTTGCAGGAGTACATCTTGGATCTGTCAATTATGTTATGGTCGGAGTACCACT

CACGGTAACCTGGTCGAATTAGGAGAAGCTGTAGGTATTATTGCGGGTCAATCAATTGGA

GAACCGGGAACTCAACTAACATTAAGAACTTTTCATACCGGTGGAGTATTCACAGGCGGT

ATCGCAGAACATGTACGAGCTCCTTCTAATGGAAAAATTAAATTCAATGAGGATTTAGTT

CATCCCACACGTACACGTCATGGACATCCCGCTTTTCTATGCCATATAG---ACCTGCAT

GTAACTAT---TGAGAGTCAGGATATTATACATAAAGTGAATATTCCACCAAAAAGTTTT

CTTTTGGTTCAAAACGATCAATATGTAGAATCAGAACAAGTGATTGCTGAGATTCGCGCT

GGAACATCCACTTTA------AAGGAGAGGGTTCAAAAACATATTTATTCTGACTCAGAG

GGAGAGATGCACTGGAGTACCGATGTGTACCATACACCTGAATATACGCATGGTAATGTT

CATCTCTTACCCAAAACAAGTCATTTATGGGTATTATCAGGAAGTCCATGCAGA------

TCCAGTATAG---TGCCTTTTCCACTCCACAAGGATCAAGATCAAATGAATGTTCAATCT

CTTTATGTCGAAGAGAGA------TATATTTCTGACCTCTCGATGAATA---ATGATCGA

GTGAGACACAAATTGTTTGGGTGGGAT--------CAAAAA---------AGGGGGAGGG

TTTCTT-ATTATTCA---GG---ACCTGA--TCGAATCATATCC------AACAGGAATT

GGGATTCCATATATCCTCT------TATTCTCCACGAGAATTCTGATTTATTGGCGAAGA

GGCGAAGAAATAGATTCATCATCCCATTCCAATATGATC----AAGAACAGGAGAAGGAA

CTAA----------GGCCTC---------CTGGTATCGTGATTAAAATACCCATAAAAGG

TATTTTACGTAGAAATAGTATTCTTGCCTATTTCGACGATCCCCGATACAGAAGAAGTA-

-----GTTCAGGAATTGCCAAAT-------------------------------------

------------------------------------------------------------

------------------------------------------------------------

------------------------------------------------------------

------------------------------------------------------------

------------------------------------------------------------

------------------------------------------------------------

--------------------------------------------------ATGGGACCAT

------------------------------------------------------------

------------------------------AGAAGTCGATTCGATAATAAAAAAAGAAGA

TTTAATTGAATATCGAAAGACAAGAGAGTTTGGACCAAAATACCAAATCCAAATCAAAGT

AAATCGATTTTTTTTCATTCCTGAAGAAGTGCATATCTTACCTAGATCTTCGTCCATAAT

GGTACGGAATAATAGTATCATTGGAGTAGATACACGAATCACTTT------GAATATAAG

AAGTCAAGTAGGCGGCTTGGTCCGAGTGGAGAAAAAAAAAAAAAGGATTGAATTGAAAAT

ATCTTCTGGAGATATCCATTTTCCTGGAGAGACGGATAATATAGCCCGGTACAGCGGCAT

CTTGATACCACCTGGAAGGG---TAAAAAA------CACGGAATCAAAAT----------

-----TCAAAAATTGGATCTATGTCCA---ACGGATCACACCTATCAAGA------AAAA

GTATTTTGTTTCAGTCCGACCCGTAGTGACATATGAAATCGCCGATGGGATAAATCTAGC

AACGTTTTTTCCCCAGGATCTGTTGCAGGAAAAGAATAATTTGCAACTACGAGTTGTCAA

TTATATTCTTTATGGAGATGGCAAACCCATTCGAGGAATTTTTCACACAAGCATTCAATT

AGTTCGGACTTGCTTAGTATTGAATTGGGATCAAGACCGGGCTGGTTCTATAGAAGAAGA

AGAGGCTTATACTTCCCTCGCTGAAGTAAGGGTAAACGATCTGATTCGAAATTTTATAAG

AATTGATTT------GGTGAAGTCTCCTATTTCGTCTACTGGAAAAGAGAATGATATGGC

GGGTTCAGGATTGATTCCTAATAATGGATCAGATCGCATCAATAC---CAATCCTTTTTT

TTCCAAGGCGAAGACTCAATCACTTAGCCAACATCAAGGAAC------TATTCGTACATT

GCTGAATAGAAATAAGGAAGGCCAGGGCGAATCTCTTATGGTTTTGTCATCATCCAATTG

TTCTCGAATTGGTCCATTGAATGGTTCGAAATATCACAATGTGACAAAAGAATCAATT--

----CAAGAGGATCCCATGATTTCAATTA---------------------GGAATTCGTT

GGGTCCGTTAGGGAC---TGTACCTAACATT------TTGAA---TTTTTCCTCATCTTA

TCATTCAATAACTCATAATGAG---------ATCTTGTTCAATAAATATTTACTACC---

---TGACAATTCAAGAGAGACTT---------TCCTAGTAC------CTAAATCTTATTT

CATGGATGAAAATAGAAGGAT------TTATAATCTTGATCCATGCAGTAACATCATTTT

GACTCCATTCAATTTGAATTGGTGCTTTCTCCATCACGA------TTATTGGGAAGATAC

ATCAAC------AATAATTAGCCTTGGACAGTTTCTTTGTGAAAATATATGTATATCCAA

AGACGGACC------ATGCGT---AAAATCTGGTCAAATTATAATTGTTCATGTTGACTC

TTTAGTAATAAGATTAGCTAAGTACCATTTGGCTACCCGCGGAGCAACCGTTCATGGTCA

TTATGGAGAAATCCTTTACGAGGGAGATACATTAGTTACTTTTATATATGAAAAATCAAG

ATCTGGTGATATAACTCAAGGTCTTCCAAAAGTTGAACAAGTGTTAGAAGTTCGTTCGAT

TGATTCAATATCGATGAACCTAGAAAAGAGGGTTGAAGGCTGGAACGAACGCATAACAGG

ATTTCTTGGAATTCCCTGGGAATTCTTTATTAGTGCTCAGCTAACCATAGTGCAAAGTCG

TATCTCTTTGGTTAATAAGATCCAAAAGGTTTATCGATCCCAGGGGGTGCAGATCCATAA

TAGGCATATAGAAACTATTGTACGACAAATAACATCAAAAGTCTTGGTTTCAGAAGATGG

AATGTCTAATGTTTTTTCACCCAGAGAACTAATCGGATTGTTGCGAGCAGAAAGAATAGG

TCGTGCTTTGGAAGACGATATATGTTACCGAGCTATTCTATTGGGAATAACGAGGGCATC

TCTGAATACTCAAAGTTTCATATCAGAAGCGAGTTTTCAAGAAACCACTCGGGTTTTAGC

GAAAGCCGCTCTCCGAAGTCGTATTGATTGGTTGAAAGGCCTGAAAGAGAATGTTGTTCT

GGGGGGGATGATACCTGTTGGTACCGGATTCAAAGGATTCG-TTCACCAC---T-CCAG-

------GGAACACAACAACATTTCTTTGGAAATAAAAAAGAAGAATCTATTCGA-TGGGA

AGATGAGA---GATATTTTGTTCTACCACAGAGAATTTTGT-------------GGTTCG

TGCA-TC-----CCAAAGAATTTTCATGAT---------------ACATCAGAACAATAA

---------------------------------------------------------ATG

GCA---------------------------------------------AGAAGATATTGG

GACATCAATTTGGAAGAGATGATGGGA-GCAGG--------GGTTCATTTTGGTCATGGT

ACTAGGAAATGGAATCCTAGAATGGCACCTTATATCTCTGGAAAGCGTAAAGGCATTCAC

ATTACAAATCTTACTAGAACGGCTCGTCCTTTATCAGAAGCCTGCGATTTAGTTTTTGAT

GCAGCAAGTAGAGGAAAGCACTTCTTAATAGTAGGTACGAAAGATAAAGCAGCCGATTCA

GTAGCATCAGCTGCAATACGGGCTCGGTGTCATTATGTCAATAAAAAATGGCTTGGTGGT

ATGTCAACGAATTGGTCCACTACAGAAACAAGACTTCATAAGTTCAGGGACTTGAGAGCA

GAACAAAAGATGGGTCGATTTAAGC---GTCTCCCGAAAAGAGATGCGGCAATGTTGAAG

AGACAATTATCTCACTTGCAAACATATCTGGGCGGGATCAAATATATGACGGGGTTACCC

GATATTGTAATCATCGTGGATCACCAAGAAGAATATACGGCTATTAGAGAATGCCTCACT

TTGGGTATTCCGACGATTTGTTTAATCGATACAAATTGTGATCCGGATCTCGCAGATATT

CCGATTCCAGCCAACGATGACGCTATAGCTTCAATCCAATTGATTCTTAACAAATTAGTA

TTGGCAATTCGT----GAGGGTCATTATAGCTATCTAGG------------AAGTCATTG

AATGGGACAGAAAATAAATCCACTTGGTTTCAGACTTGGCACA--ACCCAAAGTCATCGT

TCCCTTTGGTTCGCACAACCAAAGGATTATTCGAGGAATCTACAAGAAGATGAAAAAATA

CGGGATTGTATCAAGAATTATGTACAAAAACACATGAGAA--------------------

-------------------------------------------------TATCCTCAGGG

TTCCAA------GGAATTGCACGTCTAGGGATTCAAAAAAGAATCGATCTCATCCAGGTC

ACAATCTATATTGGGTCCTCCAAT---TTGTTAATTGAGGGCCCAACCCGAGGAATCGAA

GAATTACGAACAGATGTACAAAAAAAGTTAAATTCTATGAACCGGAGACTCAACATTACT

ATCACAAGAATTGCAAGACCCTATGAACAACCTAATATTCTTGCAGAATATATTGCTCTG

CAATTAAAAAATAGAGTTTCATTTCGAAAAGCAATGAAAAAAGCTATTGAATTAGCTGAA

CAAGCAAATACAAAGGGGATTCGGGTACAAATTGCGGGGCGTCTGAACGGAAAAGAGATT

GCCCGTGTCGAATGGATCAGAGAAGGCAGGGTTCCCCTACACACTATTCGAGCTAAAATT

GATTATTGTTCTTATATGGTTCAAACTATCTACGGGGTATTAGGGATCAAAATTTGGATA

TTTGTAGATGAA---GAATAA------ATGTCTCGTTACCGAGGACCTCGTTTCAAAAAA

ATACGCCGTCTGGGGGCTTTACCGGGACTAACTAGTAAAAGACCTAGATCTGGAAGTGAT

CTTAGAAACCAATCCC----GTTCTG--GAAAAAGATCTCAATATCGTATTCGTTTAGAA

GAAAAACAGAAATTGCGTTTTCATTATGGTCTGACGGAGAGACAATTACTTAGATATGTT

CGTATCGCCGGAAAAGCCAAAGGGTCAACAGGACAGGTTTTACTGCAACTACTCGAGATG

CGTTTGGATAACATTCTTTTTCGATTGGGTATGGCTTCGACCATTCCTGGAGCCAGACAA

TTAGTTAA--CCAT-AGACATATTTTAG--TTAATGGTC--GTCTAGT-AGATATACCAA

GTTATCGCTGCAAACCCG--GAGATTTTG-TTACTAC--GAGGGAGAAAG----AAAGAT

CCAGAGCTCTGATTCAAAATCATATGAATTCATCCCCCAACGAGGAGTTGCCAAAACATT

TGACTCT---TTACTCGTTGCAGTATAAAGGATTAGTAAATCAAATAATAGATAG-TAAA

TGGATCGGTTTGAAAATCAATGAATTGTTAGTCGTAGAATATTATTCCCGTCAGACTTAA

ATGTCACGTCGAGGTACTGCAGA------AGAAAAAACTGCAAAATCCGATCCAATTTAT

CGTAATCGATTAGTTAACATGTTGGTTAACCGTATTCTGAAACACGGAAAAAAATCATTG

GCTTATCAAATTATCTATCGAGCCTTGAAAAAGATTCAACAAAAGACAGAAACAAATCCA

CTATCTGTTTTACGTCAAGCAATACGTGGAGTAACTCCCGATATAGCAGTAAAAGCAAGA

CG---TGTAGGCGGATCGACTCATCAAGTTCCCATTGAAATAGGATCTACACAAGGAAAA

GCACTTGCCATTCGTTGGTTATTAGGGGCATCCCGAAAACGT---CCGGGTCGAAATATG

GCTTTCAAATTAAGTTCCGAATTAGTGGATGCTGCCAAAGGGAACGGCGATGCCATACGC

AAAAAGGAAGAGACTCATAGAATGGCAGAGGCCAATAGAGCTTTTGCACATTTTCGTTAA

ATGGGTAGGGACACTATTGCCAATATAATAACTTGTATAAGAAATGCCGACATGGATAAA

AAAAGAATGGTTCGAATAGCATCTACCAATATAACCGAAAACATTGTTAAAATAATTCTA

CAAGAAGGTTTTATTGAAAACGTTAGGAAACATCGGGAAAACAACAAAAATTTCCTGGTT

TTAACCTTGCGACAT------AAAAGGAATAGGAAAGG----------------------

------------------------------------------------------------

--------------------------------------GACATAT---------------

------------------------------------------------------------

------------------------------------------------------------

------------------------------------------------------------

------------AGAAAGA------CTTTAAAACGTATAAGCAGATCCGGTCTACGAATC

TATTCCAACTATCAACGAATTCCTAGGATTTCAGGTGGAATGGGGGTTGTAATTCTTTCT

ACTTCTCGAGGTATAATGACAGATCGAGAGGCTCGACGAGAAGGAATTGGAGGAGAGATT

TTATGTTATATATGGTGAATGAAAAAACCTATACCAAGAATTGGTTCGCGTAGAAATGGA

CGTATTGGTTCACGT------------------------AAGAATGGACGTAGA------

------------------ATACCCAAAGGAGTTATTCATGTTCAAGCGAGTTTCAATAAT

ACCATTGTGACTGTTACAGATGTACGAGGGCGGGTGGTTTCTTGGTGCTCCGCGGGTACC

TGTGGATTCAGAGGCACAAGAAGAGGAACACCGTTTGCTGCTCAAACCGCAGCAACAAAT

GCTATTCGTACGGTAGTGGATCAGGGTATGCAACGAGCAGAAGTTATGATAAAGGGACCT

GGTCTCGGAAGAGATGCAGCATTACGAGCCATTCGCAGAAGTGGTGTACTATTAAGTTTC

GTACGTGACGTAACTCCCATGCCACATAATGGATGTAGACCCCCTAAAAAAAGACGCGTG

TAGATGCCAACTATTAAACAACTTATTAGAAACACAAGACAGCCAATCAGAAATGTTACG

AAATCTCCCGCTCTTCGAGGATGCCCTCAGCGTAGAGGAACATGTACTAGGGTGTAT-AC

TATCACCCCCAAAAAACCAAACTCTGCCTTACGTAAAGTTGCCAGAGTACGATTAACCTC

TGGATTTGAAATCACTGCTTATATACCTGGTATTGGCCATAATTCACAAGAACATTCTGT

AGTATTAGTAAGAGGAGGGAGGGTTAAGGATTTACCCGGTGTGAGATATCACATTGTTCG

AGGAACCCTAGATGCTGTCGGAGTAAAGGATCGTCAACAAGGGCGTTCTAGTGCGTTGTA

GATT----CTTATCCAAGACTTGTATCATT---TGATGATGCCATGTGAATCGCTAGAAA

CATGTGAAGTATATGGCTAACCCAATAACGAAAGTTTCGTAAGGGGACTGGAGCAGGCTA

CCATGAGACAAAA-GATCTTCTTT-CTAAAGA-----GATTCGGAACTATT------ATA

TGTCCAAGGTCCAATATTGAAATCATTTCAGAGGTTTTCCCTTACTTTGTCCGTGTCAAC

AAACGATTTGAAATACCTCGACTTTTTCAGAACAGGTCCGAGTCAAATAGCAATGATTCG

AAGCACTTCTTT-----TTACACTCTTTCGGAAACCCAAGGACTCGATCGTATGGATATG

TAAAATACAGGATTTCCAATCATAGCAAGA-AAAGGAGGGAAACGGATACTCAATCT---

---AAAGTGAGTAAACAGAATTCCATAC--------TCGATCCCAT-----AGATACATA

TAGAATTCTGTGGAAAGCCGTATTCGATGAAAGTCGTATGTACGGCTTGGAGGGAGATCT

TTCATATCTTTCGAGATCCACCCTACAATATGGGGTCAAAAAGCCAAAATAA--------

---------ATGGCAAGGAAAAGTCTGATTCAGAGGGAGAGGAAGAGGCAGAAATTGGAA

CAGAAATATCATTTGATTCGTCGATCCTTAAAAAAAGAAAT---AAGCAAAACTCCATCG

TTGAGT------GACAAATGGAAAATTCATGGAAAATTGCAATCCCCACCACGTAATAGT

GCACCTATACGTCTTCATCGACGTTGTTTTTCGACCGGAAGACCTAGAGCTAACTATCGA

GACTTTGGGCTATCCGGACACGTACTCCGCGAGATGGTTCATGCATGTTTGTTGCCGGGG

GCAACAAGATCGAGCTGGTAA------------------------------------ATG

GT---CATAAATTCGTTTATAT-CATCTATTTCA---------------AAAGAAGAA--

----AAAAAGGGGTCTGTTGAATTTCAAGTATTCTGTTTCACCGATAAGATACGGAAACT

TACTTCACATTTGGAACTGCACAAAAAGGACTTTTTATCCCAAAGAGGTATGCGTAAAAT

TCTGGGAAAACGTCAACGGATGCTGGCTTATTTGTCAAATAAAAATAAAGTACGTTATAA

GAAATTAATCGGTCAGTTGAATATTCGGGAGCCAAAGACTCGTTAAATGGTAAAACTTCG

TTTGAAACGATGTGGTAGAAAGCAACGTGCGACTTGAACGACATGATCGGC--TGTGGAT

TA----ATCCACCATTTTATATATGAATGAAGATGCTCTTGACTCAACATCCTTTGTTCT

GTTCCACGAGGATAGGACCCGCTCTTATTGGGTTGTAATTTAA-----TAAGTAA--AT-

----------------------------------------AGTACATGATGGAGCTCGAG

-CAGAA-AGTCAAATATTGA----TTCATT-GATC-AGGGGTCAGGATCTAGGGTTAGTC

CC------AATCAATAAGTTGGAACAACTTCGTAA---ATAGATCTTC------------

-------------------------------GAAAGGGGGT--------CAAATTCTAAC

AA-----GTTTCAAATAAAAAAG---------CAAAGTTGTTGAAAT-TGCCGAAACTCT

TTCGATCAA-----AAGTGTATTACGGGGAAATC----------AATCATTCGTATG---

----ATTCTTCAACGT----------------------------AAAGAAATACCAAAAG

GCATGTTGCTGTCGTTTCGG------AACGATTAAGGATCACCGAAGTAATGTCTAAACC

TAATGATTC---------------AAAGATATGGGATCTCAGAACAAGGAAATGCCATTT

TCAA---TTCTTATTGTCTCAA----------------------CAACTAG---------

----------------------------AATGATCTTAGATGAGACAAAG-GAAAAAGGT

TAGAGACGTCTCAATAAA-------TAAATAAATGTTGAAGGG---TCTCCCTTCGAGAT

CTTTCAG----AATTACCCAACTTG-AGTTATGAGTACGAATGGTAGTATTTTTTTTTTA

GTTTTTAGAAAGGATGGCGAAAAAAGACGA--CTCAAATCATAATCCAATTGATGAT---

-------------TTTATGGATGCATTTGCCATGCCATAATACAA---------------

------------------------------------------------------------

------------------TACATAAATTGAAT----------------------------

-------------------TCCTTTTTTCTCGAGCCGTACGAGGAGAAAACTTCCTATAC

GTTTCTA-----GGGGGGGTGTTATT------CATCTATCCCAATGAGTCATTTATCGAA

---TCGTTGCAATTGATGTTAGATCCCGAAGAGACGGAAGAGCTCTTCGGAAAGTGGGTT

TTTACGATCCAATAAAGAATCAAACTTATTCAAACGTTCCTGCTATTCTATATTTCCTTG

AAAAAGGAGCTCAGCCTACAGTAACTGTTCATGATATTTCAAAGAAGGCGGAGGTATTTA

AA----------------------------------------------------------

TAAATGGATAAA------------------------------------------------

---------TCCAAGCAAC-----------------------------------------

------------------------------------------------------------

-------------------CTTTTCGTAAATCCAAGCGATC-------------------

--TTTTCGTAGGCGTTTGCC------------------------CCCAATTGGATCGGGG

GATCGAATTGATTATAGAAACATGAGTTTAATTGGTCGATTTATTAGCGAACAAGGAAAA

ATATTATCCAGACGAGTGAATAGATTGACCTTAAAACAACAACGATTAATTACTATTGCT

ATAAAACAAGCTCGTATTTTATCTCCGTTACCTTTT-----------CTTAAT------A

ATGAGAAACAATTTGAGAGAACCGAATCCCTCCCTAGA-ATTACTGGTACTAGAACCATA

AAGAAATAG---------------------------------------------------

----------------------------------------------------ATGTCACG

TTC------------------------------------------------------ACT

TAAAAAAAATCCTTTTGTAGCTAATAATTTATTATTAAAAATTCATAAGCTTAACACG--

----------------------AGGGAGGAAAAAGAAATTATAGTAACTTGGTCCCGGGC

ATCTACCATTATACCCACTATGATTGGTCATACAATTGCTATTCATAATGGAAAAGAGCA

TTTACCTGTTTATGTAACAGATCGTATGGTAGGTCACAAATTGGGAGAATTTGCACCTAC

TCTTACTTTCCAGGG------ACATGCGAGAAA---------------------------

------------------------------------CGATAGTAAATTTCGTCGTTAAAT

GATT-------------------------------TTGAAATCTTT---TCTACTAGGTA

A---------TCTATTATCCTTATGCA--------------TGAAGATAAGAAATTCGGT

CGTTGTGGTCGGACTCTATTATGGATTTATTACC------ACATTCTCTATAGGGCCC--

--TCTTATC--------TCTTCCTTCTCCGAGCTCGGGTTATGGAAGAAGGAACCGAGAA

GGAGGTATCAGCAACAACTGGTTTTATTATGGGACAGTTCATGATGCTCATATCGATCTA

TTATACGCCTCTGCATCTAGCATTGGGTAGACCTCATACAATAACTGTCCTAGTTCTACC

CTATCTTTT-------GTTTCAT------TTCTTCTGGAACAATCACAAACACTTTTTTT

--CTTATAGAT---------CTAC---TACCAGAAATTCAATG---------CGTAATCT

CAGCA--------------------TTCAATGTGTA------------------------

------TTCCTGAATAATCTCATTTTTCAATTATTCAACCATTTCATTTTACCAAGTTCA

ACGTTAGCCAGATTAGTCAACATTTATATGTTTCGATGCAACAACAAGATGTTATTTGTA

ACAAGTAGTTTTGTTGGTTGGTTAATCGGTCACATTTTATTCATGAAATGGGTTGGA---

------------TTGGTATTATTCTGGATACGGCAAAATCATTCTATTAGATCGAATA--

-------AGTACATTAAGTACCTTGTGTCAGAATTGAGAAAGTCTATGGCTCG-------

--AATCTTGAGTATT-CTCTTGTTTATCACCTGTGTCTA--CTATTTAGGCAGAATACCA

TCGCCTATTTTCACT---------------------------------------------

------------------------AAGAAACTG------AAAGAAACCTCAGAAACA---

------------------------------------------------------------

----------------------------------------GGGGAAACTGAGGA------

---------------------------------AGAAACAGATG------TAGAAATAGA

AAGAA------------------------------CTTCCGAAACG------AAGGGGAC

T--------------GAACAG---GAAAAAGAGGGATC--AACCG---------------

-AAGAAGACCCTTCCCTTTTTTGTT------CGGAAGAAAA------------GG-----

--GGGATCCGGAC---AAA---------------ATA-----------------------

----------------------GATGAAAGAGAAG--------G---------AGTGAAT

---------GGAAA----------AGAAAAAACAAA------------------------

-----------------------------------------GGATCACTTC---------

---------------AACCTCAAAGAAAG--------------ATGGTAT-----AACAA

TAAGACTGT------ATACCGGGATCAGGAAGATAATACTGCATATGGAGATGGGAATCA

GGAAGA------TT--------GGGAGTTGAAAGCACTTAAA----------------CT

TAAA--------GAAGAGAAAAAATTA---------------TTCTGGT-----------

TTGAAAAACCCTTTAT---------------------TACTTTTCTTTTCGA--------

-----------------CTATCAACGAT--GGAATCGACCATTTCGATATATAGAAAA--

--------------------------TGATCAA----------------------TTTGC

------AAA---TACTGTAAGAAATGAAATGTCCCAA-----------------------

---------------------------------TATTTTTTTAACACATGTCCAAG----

---TGATGGAAAACGAG------------------TAATCTCTTTCAC------------

--------------ATATCCTCCCAGTTTGTCGGTTTTTGGG---------GAAATGAT-

-ACAAAAA----------------------------AAAATGTCTTTA------------

TGCGGTATGAAAGAACTATATC-----CCAAAG----------------ACAAAAATCTG

TATAA------TCATTGGG----------------TTTCTACA--------AATGAACAA

AAAAGGGATAAGGTA-AGCAAGGAGTTCATAAATCGAA------------TAGAAGCTCT

AT---------------ACGGGGGAGATATTGTT---------CTCCAAAAAAGG-----

---------GTCAGATTATGC----------------AATGATCGGGATGAAAAA--GAA

TGCTTACCCAAAATGTCTGATCC--CTTTTTAAACGGATCATACCGTGGAACAATAAAAG

AATTGTATTC------------------------------AAATTCAATTAT-------A

AACGA---------TCCAATCACTTC---------------G------------ACATA-

--------------------------TAATTCCGGAGAAAAATTTTGGATAAATAGAATC

CACAGTCTCCT-------------------------------------------------

------------------------------------------------------------

------------------------------------------------------------

------------------------------------------------------------

------------------------------------------------------------

----------------------------------TTTTTG--------------------

-----------------------------------------TGATCTAAATGATCAAG--

C---AATTAGGAAT-----------AAATCTAG------TGAAA-------T------T-

-----CAAGAAATAAAGAAAAA-------GATACCTCAA---------CGGTTATA----

-----------------CAGATTAA---CTACC---GA--------TTTG----------

--GA--------------------------------ACAAGACGA--GGGAGAAGA----

------------------------------------------------------------

------------TG---AGAG-------AGAATCTGTAGAGT---TTCCTGAAATTC---

-------------------------GTTCAAGAA------------------AAACTAAA

CA------------------TATGGTAATTTAT-------------------GCTGATAA

CGAGGAGA------------ATAC-------------------GAATAT------CTTT-

------------------------------------------------------------

------------------------------------------------------------

-----------------------------------ACTCATACTGGTGCCGGGACTACTT

ACGAT------------------------------------------------------C

CAGAAAATGATCAA---------------ATGGAA------GAGGTGGATGTGATACGTT

ACACACAGAAACCTGATTTTCGTCGGGATCTAATCAAAGGATCGATGCGCGCTCAACGAC

GGAAAACAGTTA---CTTGGGAAA-----------TGCTTC---AAATAAATTTGCATTC

CCC------CCTATTTTTGGAT----------AGAATAGACAAAAACCCTTTTTT-----

-TTA---------TTTTGATA---T---------CTCCAGAATCATAAACCT------AA

TTTTTAGGGATTGGACAAAGAAAAAAC------CTGAATT---------TCAAACTTCC-

-----------AACTTTAAAG---------------------------------------

-------------AGGA-------------AACAAAAAAAGAGG----------------

-----------------ATGA------------------GATAGAAGAAACTTGGGAGAC

TATTGCATTTGGTCAAGTAA-------------TAAGAGGTTTTCTGTTAGTAACCCAAT

CTATTCTTAGAAAATACATCGTATTACCTTCATTGATAATAGCTAAGAATCTCGTGCGTA

TGTTATTATTTCAACTCCCCGAGTGGTATGAGGATTTCAAGGAATGGAGTAGAGAAATGC

ATATTAAATGCACCTATCATGGTGTTCAATTATCAGAAAGAAAATTTCCGAAGAATTGGT

TAAAAGCCGGTATTCAGATAAAGATTCTATTTCCTTTCTATTTGAAACCTTGGCGCAAAT

------CTA--AACTACGATCC------CATCATATAGATCATCCGATG---AAAAAAAG

AAAAAAACAAAATTCTT--------GTTTTTTAACAGTCTGGGGAATGGAAACTGACCTA

CCCTTTGGT---CCTGCCCGAAAGG----GACCTTCC-----TTTTTTGAGCCTATTCAT

AAAGAACTTGA------------AAAAAAGT------------TTAAAAAAGGAAAAAAG

AAAT------GGTTTTTATTTGTAAGAA--------------------------------

----------------TTTTCAAAGAAAAAAA---------A---ATATGGGTTA-----

--------------------------------------TCAAAAGAGTTCTAT-----TT

ATAAAAG------GAGTAATGAAAGAACTT------------------------------

---------------------ACAAAAGCAAATCCCGTTTTCTTATTCGGACTAAA----

-----GAGAGTCTAT---------GATGGAAGTGAAAATAGG-----AAAGATTCCATAA

G------------TAATAATAAGACAATTTCTGAATCACCTATTA------GA-----AA

ATCACCTATTAGAACTGGATCGATGGATTGGACAAATTCTTCACTGACAG----------

------------------AAAGAAAAA--------GAAAGGATCTGTCTGATAAGACAAC

CACAATCAGAGAT---------------------CAAA------TAGAAAGGATCAGGAT

CAC----AAG--------------AGACAAGAAAA------CAAATTTCCTAACTATA--

-------GATATGAATATCAGTCCTAATGAGACAA---------GTTATAGTGATAAAA-

-----GATCGGAATC-------ACAGAAA---------CCTATTTGGCAAGT--------

-ATCC---AAAA------------------------------------------------

----------------------------GAAGAAG---------------TACTCGATTA

CTCTATAAA---TGGCGCTA-----------------TTTTATGAAA-------------

---------AGTTTCATTGAAAGAATACACAGAAATG-----------------------

-------------------------------------TCTTTCTATGTATGATTAACC--

TCCCTAGGATCAATACA------CAACT---------------TTTTCTTGAATCAACAA

AAAAG-------------------ATTATAGATAAAT---ACATTTCC-----------A

ATGATGAAAAAAAT---------------CAAAG--------------------------

-------------------------TGGAATTGATGAAAAGAATAAAAAAAA--------

------GATCCACTTTATCTTTATTTCGACT-----------AGGAAGCTG---------

------------CTTTCCACTATCTCTAATAAGAA---------------------GACT

AATAATTCAAAGATTC---------------------------ATTG---------TTCG

TTGTCTCAAGCATATGTATTTTACAAATTATCGCAAAAACCAGGTATTAAGAAGTATCGC

TTGGGATCTTTATTTCAATAC------CGCAGAGCGTAT---TCTATTAAAGATAGAATA

AAG------------GACTATTTGGAAATAGAAAGAATATTTCATTCCGAATCAAAACAT

AAGAAACCGGGTAATTTCGGAATG-AACGAATGGAAAAACTGGTTAATAGGTAATTATCA

ATACAATTTTTC-TTATA--CTAGATGGTCTAGATTAGACCCGCAAAAATGGCGAAATAA

AGTAAATGAACAGTGTATGA------TAAAAA------ATAAGGATTCGAAAAAAACCGA

TTCATATTCATATGAGAAAGACCCATTAATTAATCACGAG----AGACATAT------TT

TGTATTC------AGCGGATTTATTGC---------------------------AGAATC

ATAAAAAAAAACT----TAGCAAACGTTACAGATATGATCTTTTATCATATAGATATATC

---------------AATTTTGGAAATA---------------------------ACTCG

AGTATTTATAGATCCC----------------------TATTACAAGTGAACGAA-----

------------------------------------------------------------

------GAGCAAAGGATTCTAATTCTATCTAGTCAT-AGT---CACATCATACATAAATA

TGAACAATTGA---ATTTAC---------CAGCTATTAGCGATTATCTAGAAGAAAGGTT

TATCGTTGATATGGAGAAAA------AGACAGA-----TAGAAA---ATATTT-TGATC-

TGAGAATCATCAAATTTT-G-----------GTCTAGAAGGAATACCA------ATACTG

------------ATATGGATCTCAGGATC---------------AATACTAACAAAAAGA

ATAATACTGGAACGAATTA--TTATCAAATGATTG-ATAAGAAA-----GATCTTATTTA

TC------TCACGAGTTATCAAGAAATTTACC------CTAGAAATAAAGAAA---ATAA

CTT------TTTTGATTGGATG---GGAATGAATGAAGAACTACT---------ATATCG

TCGCATAT-----------------CGAATCTGGCGCTTTGGTTT-------------TT

CCCAGAACTTGTACTACT------------------TAATGACGCATATAAGACTAAACC

TTGGACCATACCCATAAGATTGCTTCTTTTTAA------TG---------GAAAGAAAA-

-----------------------------AGATTACTGAAACTCAAAAAA----------

------TGAATGAAAATAA-------------------AAAAAGGGA------TCTTGGT

ATATTATCCAATCAA------AA--AAAATATCTGGAATTAGGGAC---------TCGAG

ACCGGGAAGA-------------------------GAAAAAATGGTGG---GATCAAG--

--AAGATC---T--------------------TAGACCAGATACTAAATA----CCAAGA

ATATTTTGGATCAGATGTAAAAAACCAAAAAGATGTTGAATTAGATGTTTGGTACAGAGA

AGGAAAGAGTAGGGAACAAGAGGA---------TTACA------CTGACATTAATAAAAG

TAGGAAAAAGAAGCAATC---------------CAAGAG-CAACAAGGAAGCAGAACTGG

ATTTACTCCTGAAA-----AGATAT----------TTACT--------TTTTC-------

---AATTCAGATG-------------GG---------ATGATTCTTTGAAG---------

-AAAAAAA----------TGATCAA-----------------------------------

-TAACATCAAATTATATAGTCTCCTGCTTAG-------ACTCATG--GATCC-------A

AAAAAAATTGTTA-------------------------------TATC----CTCTATTC

AAAGA-----------------GGAGAAATGTGGTTGGATATAATGCT------AAT---

---TCATAAGGATCTACCTCTTACAA----------------------------------

AATTGAAA--------AAAGGGGGAGT------ATTT-----------------------

------ACTAT------GGAACCGCTTCGTCTATCTAGAAAATGGAA-------------

-------------------------------------------------TGGACAATTTC

TTATGTATCAAACCCTAAGTATTTCATTG-------GTCCATAAGA--------------

-TTAAGCAGC------AAACCGATCGAAGATACCGAGAAACAAAAT--------------

-----ATATTGAT---GAGAATTTTTTCGATTTATTCATTCCACGGAACGGAAGGGTGTT

TGT---GAAT-GGAGACAAT---------------------------------AAGAATT

ATGATTTGT------------TTGTTCCTGAAAATATTTCAGCTCTGAGACGGCGTAGAG

AATTGAGAATTCTAAGTCGTCTCAATCCCGGAAAA----GGAA--------------ACA

TAAATATAATATTTTCCAATAGGAAGAAGATAC------AGAACTGTAAGC-----CATT

TTTGGATAG-----------GGGCAAGCATCT------TGAGACAGATAC------AAAT

------AAAGCCATTAAATTC------------------AAATTGTTTCTTTGGCCCAAC

CATCGATTAGAAGATTTAGCTTGCATGAATCGCTATTGGTTTGATACCAATAATGGTACT

CGTTTCAGTATGTCAAGGATACGTATGTATCC-ACGATTCGGAATTAGTTGA--------

-------------------------------------------------ATGAAAGGACA

TCAAC------------------------TCAA---------AT----------------

------------------------------------------------------------

-CCTGGATCTTCGAATTGAGAGAGATATTGAGAGAGATCAAGAATTCTCACTATTTCTTA

GATTCATGGACAAAATTGAATTTAGTGGGATCTTTCACTCACATTTTTTTCCACCAAGAA

CGTTTTATGAAACTCTTTGACCCCCGAATTTGGAGTATCCTACTTTCATGCGATTCGC--

----AGAGTTCAACAAGCAATCGATATTTCACGATCAAAGGTGTAGTACTGCTTGTAGTA

GCGGTCCTTATATATCGTATTAACAATCGA---AATATGGTCGAAAGAAAAAATCTCTAT

TTGATGAGGC---------------TTCTTCC---TATACCTATGAATTCCATTGGGCCC

AGAAAT------------GATACATTGGAAGAATCTTTTTTGTCTTCCAATATCAATAGG

TTGATTGTTTCGC------TCCTGTATCTTCCAAAAGGGAAAAAGATCTCTGAGAGTTGT

TTCATGGATCCGAAAGAGAGTACTTGGCTTCTCCCAATAACTA---------AAAAGTGT

ATCATGCCTGAATCTAACT------------GGGGTTCGCGGTGGTGGAGGAACCGGATC

GGAAAAAAGAGGGATTCTAGTTGTAAGATATCTAATGAAACAGTAGCTGGAATTGAGATC

TCATTCAAAGAGAAAGATATCAAATATCTGGAGTTTCTTTTTGTATCCTATACGGATACG

GATGATCCGATCCGCAAGGACCATGATTTGGAAT---TGTTTGATCGTCTTTCTCCGGGG

AAGAAGCAAAACATAATCAACTTGAATTCGGGACAGCTATTCGAAATCTTAGTGAAACAC

TTGATTTGTTATCTCATGTCTGCTTTTCGTGAAAAAAGACCAATTGAAGTGGAGGGTTTC

TTCAAACAACAAGGAGCTGAGGCAACTATTCAATCAAATGATATTGAGCATGTTCCCCG-

-----TC-----------------------------------------------------

------------------------------------------------------------

-TCTTCTCGGGAAACAAAAACAAGTGGGGTATTTCTTTGCAAAATTGCGCTCAATTTCAT

ATGTGGCAATTCCACCAGGATCTCTTCGTTAGTTGGGGGAAGAATCAGCACGAATCGGAT

TTTTTGAGGAACGTATCGAGAGAGAATTTGATTTGGTTAGGC---AGTATGTGGTTGGTA

AACAAGGATAGGTTTTTTAGCAAGGTACGGAATGTATCGTCAAATATTCAATACGATTCC

ACAAGATCTATTTTCGTTAAAGTAACGGATTCTAGCCAATTGAAAGGA---TCTTCTGAT

CAATCCAGAGATCATTTCAATTCCATTAGTAATGAGGATTCGGAATATCACACATTGATA

CATCAAACAG--------------------------------------------------

------------------------------------------------------------

------------------------------------------------------------

-------AAATTGAGCAAC---------TAAAAGAAAGATCGATTCTTTGGGATC---CT

TCCTTTCTTCAAACGGAACGAAC------AGAGATAGAATCAGATCGATTCCCGAAATGC

CTTTCTGGATATTTCTCAATGTCCCGG---------CTATTCACGGAACGTGAGAAGCAG

ATGAATAATCATCTGTTTCCG------GAAGAAATCGAAGAATTTATTGGGAATCCTACA

AGATCAATTCGTTCTTTTTTCTCTGACAGATGGTCA------GAACTTCATCTG---GGG

TCGAATGCTACTGAGAGGTCCACTAGAGATCAGAA------------ATTGTTGAAGAAA

CAACAAGATG---TTTCTTTTGTCCCTTCCAGGCGATCGGA------------AAATAAA

GAAATGGTTGATATATTCAAGATAATTACGTATT------TACAAAATACCGTCTCAATT

CATCCT-----ATTTCATCAGATCCGGGATGTGATATG---GTTCCGAAGGATGAACCGG

AT---ATGGACAGTTCCAATAAGATTTCATTCTTGAACAAAAATCCATTTTGTGATTTAT

TCCACCTATTCCATGGCCGGAACAAAGGTGGATACACGTTACACCACGA---TTTTGAAT

CAGAAGAGAGATTTCAAGAAAGGGCAGATCTATTCACTCTATCAATAACCGAGCCCGATC

TGATGTATCATAAGGGATTTGCCTTTTCTATTGAT---------------TCCTACGGAT

TGGATCAAAAAAAATTATTGA------ATGAGGTATTCAACTCCAGGGATGAATCGAAAA

AGAAATCTTTATTGGTTCTACCTCATATTTTTTATGAAGAGAATGAATCTTTTTATCGAA

GGATCAG-AACAAAATCGGTCCGGATCTCCTGCAGGAATGGTTTGGAAGATCCAA-----

-AAATAGTGGTATTTGCTAGCAACAACATAATGGAGGCAGTCAATCAATATAGATTGATC

CGAAATCTGATTCAAATCCAATATAGCACCTATGGGTACATAAGAAATGTATCGAAGAGA

TTCTTTTTAATGAATAGATCCGATCGCAACTTCGAATATGGAATTCAAAGGGAT---CAA

ATAGGAAATGAT----ACTCTGAATCATATAACTATAATGAAATATACGATCAACCAACA

TTTATCGAATTTGAAAAAGAGTCAGAAGAAATGGTTCGATCCTCTT----ATTTCTCGAA

CCGAGAGATCCATGAATCGGGATCCTAATGCATATAGATACAAATGGTCCAATGGGAGCA

AGAATTTCCAAGAACATTTGGAACATTTCGTTTCTGAACAGAAGAACCGTTTT-------

--CAAGTAGTGTTCGATC------GATTACG-----------------TATTAATCAACA

TTCGATTGATT-GGTCCGAGGTTATCGACAAACAAGATTTGTCTAAGTCACTTCGTTTCT

TT--TTGTCCAAGTCA--CTT------------------------CTCTTTTTGTC----

---------------------------------------TAAGTCACTTCCTTTTTTCTT

TGTGAGTATCGGGAATATCCCCATTCATAGGTCCGAGATCCACATCTATGAATTGAAAGG

TCCGAATGATCAA---CTCT---------------------GCAATCAGTTGTTAGAATC

AATAGGTGTTCAAATCGTTCATTTGAACAAATTGAAACCT------TTCTTATTAGATGA

TCATGATA--CTTCCCAAAGATCGAAATTATTGATCAATGGAGGAACAATAGCACGATTT

TGGTTCAAT-AAGATACCAA------AGTG-GATGATTGACTCATTCCATACTAGGAATA

ATCGCGGGAAATCCTTTGAGAACACGGATTCCTATTTCTCAATGATATCCCACGATCGAG

ACAATTGGCTGAATCCC-GTGAAACCCTTTCATAGGAGTTCATTGATATCTTCTTTTTAT

AAAGCAAATCAACTTCGATTCTTGAATAATCCACATCACTTCTGGTTCTATTGTAACAAA

AGATTCCCTTTTTATGC---GGAAAAGACCCGTATCAATAATTATGATCTTACATATGGA

CAATTCCTCAATATCTCGTTCATTCGCA-ACAAGATATTTTCTTTGTGCGTC------GG

TAAAAAAAAACATGTTTTTTTGGAGAGAGATACTATTTCACCTTCGCCAATTGAGTCACA

GGTATCTGACATATTCATACCTAACGATTTTC------CACAAAGTGGTGACGAAACG--

----------TATAACTT---------GTACAAATCTTTCCATTTTCCAACTCGATCCGA

TCCATTCGTTCGT------AGAGCTATTTACTCGATCGCAGACATTTCTGGAACACCTCT

GACAGAGGAACAAAGAGTCAATTTTGAAGGAACTTATTGTCAGCCTCTTTCAGATCTGAA

TCTATCTGATTCAGAAGGGAAGAACTTGCATCAGTATCTCA------GTTTCAATTCAAA

CATGGGTTTGATTCACACTCCATGTTCTGAGAAATATTTACCATCCGGAA----------

------------------------------------------------------------

----------------------AGAGGAAAAAACGGAATC--------------------

------TTTGTCTAAATA------------------------------------------

--------AATGCGTTGAGAAACGGCAGATGTATAGAACCTTTCAACGA---GATAGTGC

TTTTTCAAATATCTCAAAATGGAATCTGTTCCAAACATATATGCCATGGTTCCTTACTTC

GACAGGGTGCAAATATATAAATTTCATCCTTTTAGATACTTTTTCAGACCCATTGCCGAT

ACTAAGTAGCAGTCACAAATTTGTATCCATTTTTCATGATATTATGCA------------

TGGATCGG------CATGGTCAATTCCTCAGAAAAAATTGCGGGCGATTCTTCCACAAT-

----------GGAATCTGATAAGTGA----------------------------GATTTC

GAGTAAATG------TTTACAGAA---TCTT--------CTTCTGTCCG-AAGAAATGAT

TCATCGAAATAATGAGTCACCCGTTCCATTGATATGGACACATCTGAGATCACCAA--AT

CCTCGAGAGTTCTTCTATTCAAGCCTTTTCCTTCTTCTTGTTGCTGGATATCTCGTTCGT

ACACATCTTCTCTTTGTTTCCCGAGTCTCTAGTGAGTTACATACAGAGTTAGAAAAGATC

AA----ATCTTTGAT---------GATTCCATCATAC-ATGATGG---------------

---------------------------AGTTG----------------------------

-------------------CGAAAACTTCTGGATAGTTATCCTCCACCTGAACTGAAT--

------------------------------------------------------------

----TCTTT--CTGGTTAAAGAATCTCTTTCTAGTTGCTCTGG-----------------

--------AACAATTAGGA-------------------------GATTCTCCGGAAGAAA

TACGGGGTTCTGATTCTGGCGGCAACATGCTATTGGGTGGCGGT----------------

----CCCGCTTATGGGGTCAAATCAATACGTTCTAAGAAGAAAGATTTGAATATCAATCT

CATCGATATCATCGATCTCATAAGTATCATACCAAATCCCGTCAATCCAATCACTTTTT-

-------------CGATAAATACGAGACGTTTAAGTCGTA--------------------

------------------------------------------------------------

CGAG------------------------------TAAAGAGATCTA------TTCATTGA

TAAGAAAAAGAAAAAA--CGTGAACAGCGATTGGATTGATGGGAAAAT------------

-------------------------AGAATCCTGGGTCGCGAACAGTGATTTGA------

---------------TTGAT----------------------------------------

-----GATGAAGAAAGAGAATTCTTGGTTCAGTTCTCCGCCTTAACGACAGAAAAAAGGA

TTGATCAAATTCTATGGAGTCTGACTCATAGTGATCCTTTCTCAAAGAATGACTCTGGTT

ATCAAATGATTGAACAACCGGGATCAATTTACTTACGATACTTAGTTGACATTCATAAAA

AGTATCTAATGAATTCTGAGTTCAATAGATCCTGTTTAGCAGAAAGACGAATATTCCTTG

CTCATTATCAGACAATCACTTATTC------ACAAACCTCGTGTGGGGCTAATAGTTCTC

ATTTCCCAT---CTCATGGAAAACCCTTTTCGCTCCGCTTAGACCTATCCCCTTCTAGGG

GTATTTTAGTGATAGGTTCTATAGGAACTGGACGATCCTATTTGGTCAAATACCTAGCGA

CAAACTCCTACCTTCCTTCCATTACGGTATCTCCGAACAAGTTCCTGGATGACAAGCCTA

AAGGTTATCTTATTGATGATATTGATGATAGTGACGATATCGATCTTGATGATAGTAATA

GTATTGATGATAGTGACGATGTCGATATTGGTGATAGTGATGATATCGATATTGATGATG

ACCTTGATGC------GGAGCTGCTAACTATGACGAATGTGCTAACTATGTAT---ATGA

CGCCGAA---AATGGACC------GGTTTGATATCACCCCTCAATTAGAATTAGCAAAAG

CAATGTCTCCTTGCATAATATGGATTCCAAACATT----------CATGATCTGT-----

------------------------------------------------------------

---------------ATGTGAATGA---------------------------GTCGAATT

ACTTATCCC-----------TCGGTCTATTAGTGAACCATCTCTCCA---------GAG-

------ATTGTGAAAGATGTTCCACT------AGAAATATTCTTGTTATTGCTTCGACTC

ATATTCCCCAAAAAGTGGATCCCGCTCTAATAGCTCCGAATAAATCAAATACATGCATTA

AGATACGAAGGCTTCTTATTCCACAACAACGAAAGCACTTTTTCATTCTTTCATATACTA

GGGGATTCCACTTGGAAAAGAAGATGTTCCATACTAACGGATTCGGGTCCATAACCATGG

GTTCCAATGCACGAGATCTTGTAGCACTTACCAATGAGGCCCTATCAATTAGTATTACAC

AGAAGAAATCAATTATAGACACTAATACAATTAGATCAGCTCTTCATAGACAAACTTGGG

ATTTGCGATCC---------CAGG---TAAGATCCGTTC---AGGATCATGGGATCCTTT

TCTATCAGATAGGAAGGTCCTTTGCACAAAATGTACTTCTAAGTAATTGCTCCATAGATC

CTATATCTACCTATATGAAGAAGAAATCATGTAAGGAAGGGGATTCTTATTTGTACAAAT

GGTACTTCGAACTT---------GGAACGAGCATAAAGAAATTAACGACACTTCTTTATC

TTTTGAGTTGTTCTGCCGGATCGGTCGCTCAAGATCTTTGGT------CTCCACCCGGAC

CCGATGAAAAAAATTGGATCACTTCTTATGGATTCGTTGAGAATGATTCTGATCTAGTTC

ATGGCCTA---------------------TTAGAAGGC------GCTCTGGTGGGATCCT

CAC------GGACAGAAAAAGATTGCAGTCAGTT---T------------------GATA

ATGATCGAGTG---ACATTGCTTCTTCGGTCCGAACCAAGGAATCCGTTAGATATGATGC

AAAATGGATCTTGTTCTATCGTTGATCAGAGATTTCTATATGAAAAATACGAATCGGAGT

TTGAAGAAGGGGAAGGAGAAGGAACCCTCGACCCGCAACAG------------------A

TAGAGGAGGATTTATTCAATCACATAGTTTGGGCTCCTAGAATATGGCGC---------C

CTTGTGGCAATCTATTTGATTGT------ATCGAAAGGCCCAATGAATCGAGATTTCCCT

ATCGGTCCAGGTCATTTCGGGGCAAGCAGATCATTTCTCATAAAG---------------

------------------------------------------------------------

------------AGGATGAGCTTCAAGAGA----------------------ATGATTTG

GAGTTCTTGCAGAGT----------------GGAA----CCATGCAGTACCAGACACGAG

ATAGATC------------CTCCAA---AGAACAAGGGT------TTTTTCAAATAAGCC

AATTCATTTGGGACC------CCGCAGATCCATTCTTTTTCCTATTCAAAGATCAGCCCT

TTGTCTCTG---------TGTTTGCACGTCGAGAATTCT------TTGCAGATGAAGAGA

TGTCAAAGGGGCTTATTAC-----------------------------------------

----------TTCCC---------------------------------------------

------------------------------------------------------------

------------------------------------------------------------

------------------------------------------------------------

------------AAACACATTCTCCTACATCTATATATGAACGCTGGTTGATCAAGAATA

CGCAAGAAAAG------CACTTCGAATTGTTGATTCATCGCC---AGAGATGGCTTAGC-

-----ACCAATAGTTCATTATCTAATGGATCTTTCCGTTCTAATACGCCATCCGAGAGTT

ATCAGTATTTATCAAATCTGTTCCTATC---TAACGGAACGCTATTGGATCAAATGGCAA

AGACATTGTTGAGAAAGAGATGGCTTTTCCCGGATGAAATGAAACATTTGATTCATGTAA

CAGGAGAAAGATTTCCCATTCC----TTAG------------------------------

--------ATGCCTAGATCTCGGATAAATGGCAATTTTATCGATAAGACCTCTTCAATTG

TAGCCAATATCTTATTACGGATCATTCCGACGACTTCAGGAGAAAAAGAGGCATTTACCT

ATTACAGAGATGGTGCGATTTGAT----T---------------CTTTTTTTACTTACCA

CTC-------------------------CCATCCTTAC--GAGAAA----------TTCC

G------GATGG----AA-AGAAA-AAATTGTT-GAAA----GAAACCCACGCTTGGTGA

AGGTGAAGT-----TCGTAGATAGAACAATACCTTCTGTCGTGTATCCACGATTGATGCA

GCCTCAGATGCTTGAAT----TGTTGATTCTAGTATTGAGCGAAAGGTTACACCTATA--

----GGT----TCTCT-ATTG-----CGGGTCAATCCTACTGTA-C----TAGTACCAAT

AGGC-GGCATAG----GGGA-AGAAGCACTACACC------T-----AGAAATCAACAAC

ATAAAAACTTTGTTATA---------AATTATCCCTTTTGCTCTATTGGGATTGGGACTA

ACAA-------------GAATGGTTGGGACAACAAACATCCATCTCGTTCGTACTTTGGA

TACCCGTAT-----AACCATCGAAGATCGTTGAAGTGTCTAATTC--CT-GGAAATATGG

AGCGTTGAGGACAAAGAAATTGTTGGGGGAAGCTACCATTTTTATCT----AGGACCAAG

ACGCTTGGT--ATTAAGACAT----------TCAGAAAGGGCCTCTTGCAGGAAGGCTGG

CTAGAGATTTCTTGTAAAAA-CACCAGCCCCTGTCAGTTTATAACA--TAATGAGAATAT

TTTAATCTTT----TTTGATTCCATA-------------------------GATTATTCC

TCTTATTAC---TATGCACAGAAGGGGAGGAGCCGTATGAGATGGAAATCTCACGTACGG

TTCTGGAACGGAGATCCTTTGA-ATGGAAT------GAACGACCGTAACGGATGTCAGCT

CAATCTGAAGGAAATTATGCGGAAGCTTTACAGAATTATTATGAAGCTACGCGACCAGAA

ATTGATCCCTATGATCGAAGTTATATACTCTATAATATAGGACTTATCCACACAAGCAAC

GGAGAACATACGAAGGCTTTGGAATATTATTTTCGGGCACTAGAACGAAATCCATTCTTA

CCACAAGCTTCTAATAATATGGCCGTGATCTGTCATTACGTGCGACTATCTCTACT----

--ATAGAATAGAAAGAAGAATAGAAAGAGAATAGAATAATAATAATAAAGAATAAGAAAA

AGAAGAATAATAAAAAAGTAAAG-------AAGGAAAGAA-AGAGAAAATCCGCTAGTA-

-----------------AAT-ACTAGAAAGAGAAACAAACAAAAGAAAACAAAAAAGGCC

TTCTA-CATATACATCGTGCAAA--GGAACGGTTTT----TATCAGCTGTAGCAAAGAAA

GAGA------CTTCATAGGAGCC-GGGATATGA--AGAAATGGATATGGCCTATATACTG

ATTTCTATGGAT-----AAAA--GATCTAATTGATAGAGGAAGCACCGTAAAGATCAATG

ATTGAGGTT---TT-GGGCCCATACAA-T-----AAGACCTGCTTACT----TATGTCAA

GA-TATGAGATA--TAAAGTTAGG----AATCAACTTATGTAATAGAGTTGATCCAC---

----TAAGGTGCTGAGCAGCGGTGTAGCATCAGATCCCAAAGAT-AGTAAGTCCTTT-AT

TAT-----------CGAG---------------AAAGGTATTTTTC-AAAGATTCTATAA

TGAATT--------------TCTATACGA---AC---------GAAAATGAGATAGTTAC

CT-TTCAGAAA-------ATTCTAACGATAGGGGGAGATGGAGATATCTATGCTTCAGCT

TCATTCTTCTGAAGGTGGGAGAAGAGATAAAACTGA-TTATTTGATTGATGGAAA-TTTG

AGTTTGAAAATC-ATGTAATTAAC-----CTCTTCTGGTTA-TTTGGTTAATTCCGG--A

AAATGGGATGGAT------TTACGAGAAATCTCATTCAG-TTA----G-ATACAGTAGAT

T-----AAGACGGAAC----GCCAAAAGAGTTGACTGCTGAGCCGTATGAGGTAGGAAAC

TCTCAAGTACGGTTCTAAGGGAAGGAATTGATCC-------ACCTATCCCGACCGGGGAG

AACAGGCCATTCGACAGGGAGATTCTGAAATCGCAGAGACTTGGTCCGATCAAGCAGCTG

AGTATTGGAAACAAGCTATAGCGCTTACTCCAGGTAATTATATTGAAGCACAGAATTGGT

TGAAGATCACGAGGCGCTTCGAA------TAAATGAACTGGCGATCAGAACGTATATGGA

TAGAACTTATAACAGGTTCTCGAAAAACAAGTAATTTATGTTGGGCCTGTATACTTTTTT

TAGGTTCACTAGGGTTCTTATTAGTTGGAACTTCCAGTTATCTTGGCAGGAATCTGATAT

CCTTATTTCCGTCTCAGCAAATCCTTTTTTTTCCACAAGGGATTGTGATGTCTTTCTATG

GTATCGCAGGTCTGTTCATTAGCTCCTATTTGTGGTGCACAATTCTGTGGAATGTAGGTA

GTGGTTATGATAGATTTGATAGAAAAGAAGGAATAGTGTGTATTTTTCGTTGGGGATTTC

CTGGAAGAAATCGTCGTATCTTCTTTCGATTCCTTATGAGAGATATTCGGTCAATCAGAA

TGGAAGTGAAAGAAGGTATTTATCCT---CGTCGTGTCCTTTCTATTGAAATAAGAAGCC

AGGGATCCATTCCCTTGACTCGTACTGATGAAAA---TTTCACTCCACGAGAAATTGAGC

AAAAAGCTGCTGAATTGGCCTATTTCTTGCGCGTACCAATTGAAG------TATTTCGAA

CCAAGGAATGGATACTTTCTCGGCATGGGGTAGGAAACCCAAGAATCCTTTTTAATACAA

CTGATCTTTCTTCGGAACAACTGCTCATTCGATCAAAACATGTTAGTGTTAGATCCTATT

TCCGCTCCCTTCTGTTCCCCGTCTGCGGCTAG

>Bambusa

------------------------------------------------------------

------------------------------------------------------------
[truncated: 2,398,126 more chars]
